# Enantioselective annulation of enals with 2-naphthols by triazolium salts derived from l-phenylalanine†
†Electronic supplementary information (ESI) available: CCDC 1051429 and 1051473. For ESI and crystallographic data in CIF or other electronic format see DOI: 10.1039/c5sc00731c


**DOI:** 10.1039/c5sc00731c

**Published:** 2015-04-30

**Authors:** Guo-Tai Li, Qing Gu, Shu-Li You

**Affiliations:** a School of Pharmacy , East China University of Science and Technology , 130 Mei-Long Road , Shanghai 200237 , China . Email: slyou@sioc.ac.cn; b State Key Laboratory of Organometallic Chemistry , Shanghai Institute of Organic Chemistry , Chinese Academy of Sciences , 345 Lingling Lu , Shanghai 200032 , China; c Collaborative Innovation Center of Chemical Science and Engineering , Tianjin , China

## Abstract

A series of chiral triazolium salts have been synthesized from methyl l-phenylalaninate hydrochloride. The NHCs derived from this class of novel triazolium salts were found to be highly efficient catalysts in the annulation reaction of enals and 2-naphthols. These reactions proceeded with high chemoselectivity and wide substrate scope affording enantioenriched β-arylsplitomicins in good yields with up to 96% ee.

## Abstract 2

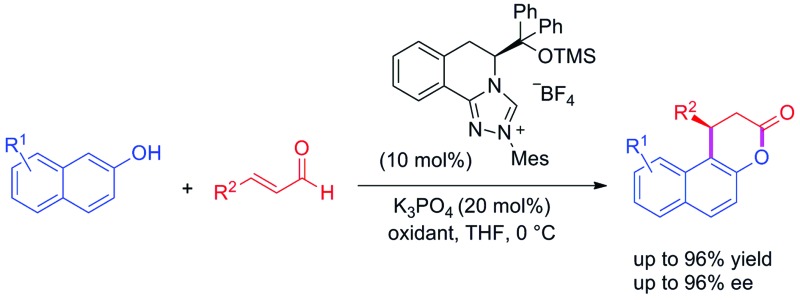
The annulation reaction between enals and 2-naphthols catalyzed by a novel NHC affords enantioenriched β-arylsplitomicins in good yields and enantioselectivity.

## 

The naphthopyran-3-one core is a characteristic structural motif existing in a variety of biologically active natural products and pharmaceuticals (Fig. 1).^1^ Specifically, β-arylsplitomicin and its analogues have recently been extensively investigated and they were found to exhibit highly interesting biological activities such as recombinant SIRT2 inhibition, antiproliferative properties and tubulin hyperacetylation in MCF7 breast cancer cells.1c–e,g Moreover, they are also important synthetic intermediates in both organic synthesis and medicinal chemistry. Consequently, various approaches to access these β-arylsplitomicin scaffolds have been established. Although the synthesis of racemic β-arylsplitomicin derivatives has been extensively carried out,^2^ limited progress on the synthesis of enantioenriched β-arylsplitomicins has been reported.1g,^3^ In 2008, Jung and coworkers reported a cyclization of β-naphthol with propiolic acid, followed by a chiral rhodium-catalyzed conjugated addition of arylboronic acids to benzo[*f*]chromen-3-one, delivering the β-arylsplitomicin in excellent enantioselectivity but with poor yield (18%).1*g* Lately, Zhang and Feng employed a chiral bifunctional thiourea–tertiary amine-catalyzed annulation between β-naphthols and akylidene Meldrum's acids to afford β-arylsplitomicins in good yields but with moderate enantioselectivity.3*a*

**Fig. 1:**
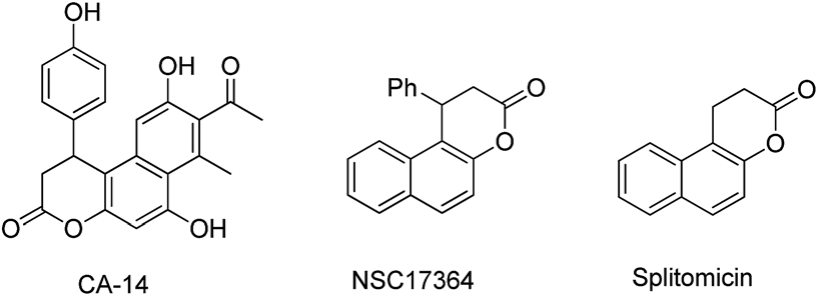
Selected bioactive compounds and natural products containing the naphthopyran-3-one core.

Recently, N-heterocyclic carbene (NHC)^4^-catalyzed reactions involving α,β-unsaturated acyl azolium intermediates generated from enals,3b,^5^ ynals,^6^ α-bromoenals,^7^ α,β-unsaturated acyl fluorides,^8^ α,β-unsaturated esters,8a,^9^ and α,β-unsaturated carboxylic acids^10^ have received great attention and enjoyed rapid development. In 2009, Bode and coworkers reported an elegant work on the NHC-catalyzed enantioselective Claisen rearrangement of ynals and 2-naphthols, affording β-phenylsplitomicin in 79% yield with 68% ee.3b,c Later, the asymmetric annulation of 2-bromoenals and β-naphthols was also realized by Biju and coworkers to deliver β-phenylsplitomicin in moderate yield and enantioselectivity.3*d* Despite these pioneering studies, highly enantioselective synthesis of β-arylsplitomicins by NHC catalysis remains to be an unsolved project (Fig. 2). In particular, the chemoselectivity of annulation over ester byproduct formation is responsible for the low yield of the desired splitomicin product. As part of our continuous interest in NHC catalysis,5d,^11^ we envisioned that the introduction of novel chiral triazolium salts might provide a solution for this highly challenging but interesting reaction. In this paper, we report the synthesis of novel NHCs derived from l-phenylalanine and their excellent performance in the annulation reaction of enals with 2-naphthols.^5^

**Fig. 2:**
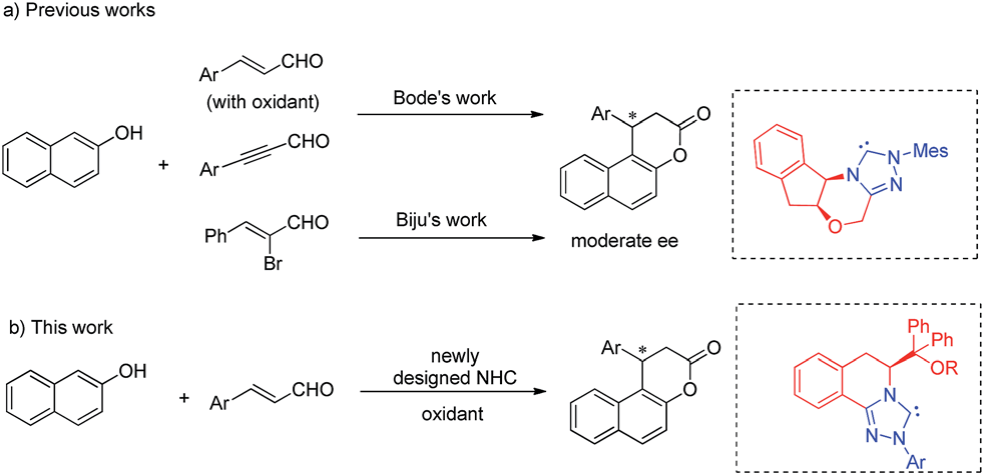
NHC-catalyzed annulation reactions of β-naphthol.

Our study began with the preparation of a series of triazolium salts from the commercially available methyl l-phenylalaninate hydrochloride (Scheme 1). The synthesis commenced with the transformation of l-phenylalaninate hydrochloride to the lactam.^12^ The addition of a Grignard reagent to the ester group in lactam **1** and protection of the hydroxyl group by TMSOTf or TBSOTf afforded the corresponding silyl ethers.^13^ Several homologous triazolium salts **4a–4g** were then prepared by following the procedures developed by Rovis and coworkers.^14^ Desilylation of **4a–4b** under acidic reflux conditions gave triazolium salts **5a–5b** bearing a free hydroxyl group.^15^ Furthermore, the structure of **4a** was confirmed by an X-ray crystallographic analysis as shown in Scheme 1.

**Scheme 1:**
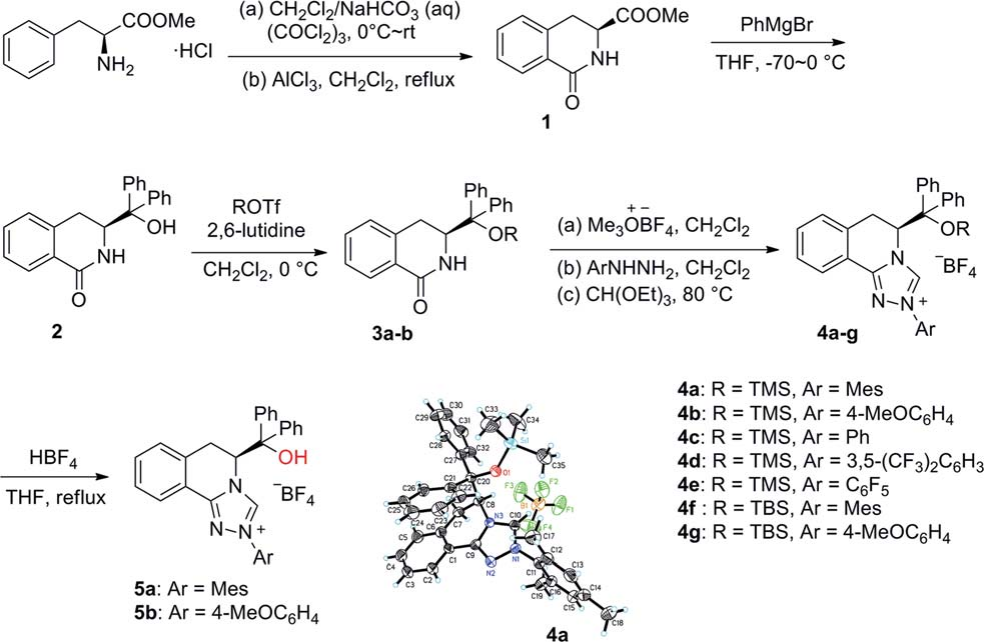
Synthesis of chiral triazolium salts derived from l-phenylalanine and the X-ray crystal structure of **4a**.

With these triazolium salts in hand, we began to test their catalytic activity in the annulation reaction between 3-methylnaphthalen-2-ol (**6a**) and (*E*)-3-(4-methoxyphenyl)acrylaldehyde (**7a**). To our delight, in the presence of 10 mol% *N*-Mes substituted triazolium salt **4a**, 20 mol% DBU and 1 equivalent of quinone **8** as oxidant in THF, the reaction proceeded smoothly to afford the corresponding β-arylsplitomicin in 68% yield and 78% ee, along with 24% yield of byproduct ester **10a** (Table 1, entry 3). Interestingly, the enantioselectivity obtained by **4a** is much higher than those obtained by previously reported NHC precursors **A** and **B** (Table 1, entries 1–2). The ee value of the product could be further increased to 84% ee when 1 equivalent of **7a** was used (Table 1, entry 5). However when **7a** was used in excess, it was difficult to isolate **9a** from the reaction mixture due to the similar *R*_f_ value between **7a** and **9a**. Other NHC precursors with different substituents were then investigated. As summarized in Table 1, NHC precursors **4b** and **4c**, bearing 4-MeOC_6_H_4_ and C_6_H_5_ groups on the N atom, respectively, gave moderate enantioselectivity and low conversions (Table 1, entries 7–8). In the presence of NHC precursors either bearing an electron-withdrawing group such as 3,5-(CF_3_)_2_C_6_H_3_ (**4d**) and C_6_F_5_ (**4e**) or a TBS-protected hydroxyl group (**4f**, **4g**), this annulation reaction became sluggish and only gave trace amounts of the desired product (Table 1, entries 9–12). Notably, NHC precursor **5a** bearing a free OH group gave comparable results (Table 1, entry 5 *vs.* entry 13).^15^

**Table 1:** Screening NHC precursors^*a*^

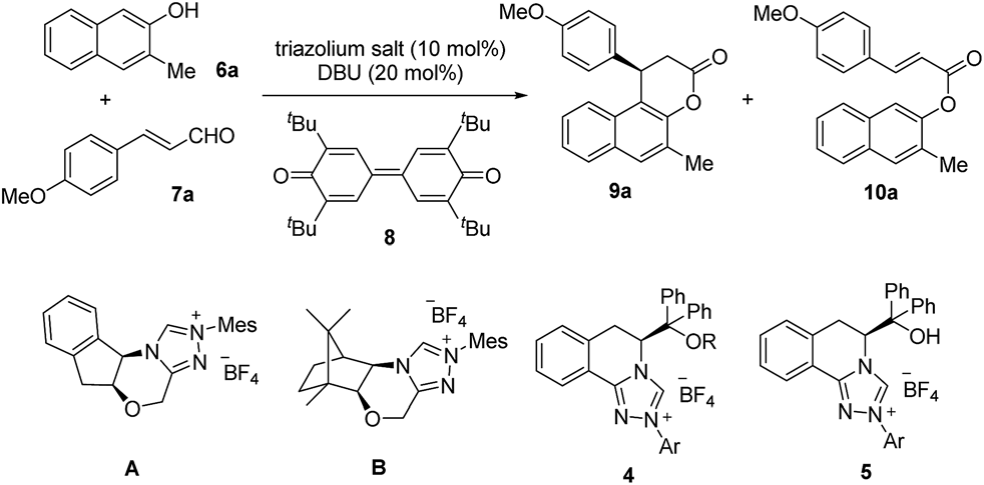						
Entry	Triazolium salt	**6a**/**7a**	Time (h)	Yield^*b*^ (%)	ee^*c*^ (%)	**9a**/**10a**^*d*^
1	**A**	3	4	74	–24	—
2	**B**	3	15	58	–24	—
3	**4a**	3	2	68	78	—
4	**4a**	2	2	68	81	—
5	**4a**	1	2	61	84	1/0.40
6	**4a**	0.5	2	—	85	—
7	**4b**	1	24	34	53	—
8	**4c**	1	48	8	51	—
9	**4d**	1	30	<5	—	—
10	**4e**	1	30	<5	—	—
11	**4f**	1	30	24 (30)^*e*^	75	1/1.27^*f*^
12	**4g**	1	48	7	42	—
13	**5a**	1	2.5	62	84	1/0.42
14	**5b**	1	24	30	54	—

^*a*^Reaction conditions: **7a** (0.2 mmol), **8** (0.2 mmol), triazolium salt (0.02 mmol), DBU (0.04 mmol) in THF (2.0 mL) at rt.

^*b*^Isolated yield for **9a**.

^*c*^Determined by HPLC.

^*d*^Determined by ^1^H NMR of the crude reaction mixture.

^*e*^Isolated yield for **10a**.

^*f*^Determined after isolation.

With **4a** as the NHC precursor, further optimization of the reaction conditions was carried out. The results are summarized in Table 2. Various solvents such as THF, toluene, CH_2_Cl_2_, ether and dioxane were tolerated well, providing the desired product in moderate to good yields and enantioselectivity. The reaction in THF gave the highest ee (Table 2, entry 1, 61% yield, 84% ee), although toluene gave a higher yield (Table 2, entry 2, 83% yield, 74% ee). Lowering the reaction temperature to 0 °C led to an increased yield and ee (Table 2, entry 8, 72% yield, 85% ee).

**Table 2:** Screening solvents and bases^*a*^

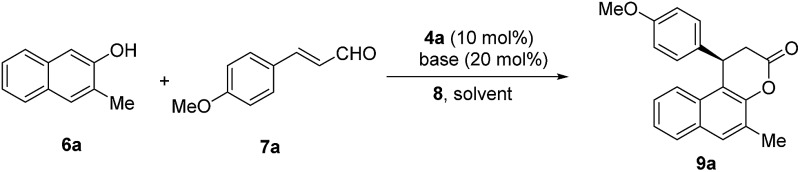							
Entry	Solvent	Base	*T* (°C)	Time (h)	Yield^*b*^ (%)	ee^*c*^ (%)	**9a**/**10a**^*d*^
1	THF	DBU	rt	3	61	84	1/0.40
2	Toluene	DBU	rt	18	83	74	1/0.16
3	Et_2_O	DBU	rt	3	64	78	1/0.42
4	DCM	DBU	rt	48	76	65	1/0.19
5	Dioxane	DBU	rt	16	57	80	1/0.62
6	PhOMe	DBU	rt	12	75	73	1/0.13^*e*^
7	CH_3_CN	DBU	rt	48	43	53	1/0.30^*e*^
8	THF	DBU	0	23	72	85	1/0.32
9	Cyclohexane	DBU	rt	24	39	81	1/0.27^*e*^
10	THF	KOAc	0	72	72	84	1/0.27
11	THF	Cs_2_CO_3_	0	2	67	86	1/0.31
12	THF	Cs_2_CO_3_	–10	8	73	86	1/0.20
13	THF	K_2_CO_3_	0	8	72	85	1/0.28
14	THF	K_3_PO_4_	0	8	72	87	1/0.23
15	THF	KO^*t*^Bu	0	1.5	72	80	1/0.38
16	THF	KHCO_3_	0	72	61	83	1/0.28
17	THF	KHMDS	0	1.5	73	81	1/0.36

^*a*^Reaction conditions: **6a** (0.2 mmol), **7a** (0.2 mmol), **8** (0.2 mmol), **4a** (0.02 mmol), base (0.04 mmol) in solvent (2.0 mL).

^*b*^Isolated yield for **9a**.

^*c*^Determined by HPLC.

^*d*^Determined by ^1^H NMR of the crude reaction mixture.

^*e*^Determined after isolation.

Several bases including DBU, KOAc, Cs_2_CO_3_, K_3_PO_4_, KHCO_3_ and KHMDS were further evaluated, and most of them gave the desired product in good yields and enantioselectivity (Table 2, entries 10–17, 61–73% yields, 80–87% ee). Among them, K_3_PO_4_ gave the best result (Table 2, entry 14, 72% yield, 87% ee). Other NHC precursors synthesized by utilizing different Grignard reagents were evaluated in this annulation reaction; unfortunately no better results were obtained compared with **4a** (see the ESI† for details). Under the optimized conditions (10 mol% of **4a** and 20 mol% of K_3_PO_4_ in THF at 0 °C), the reactions of various 2-naphthols with α,β-unsaturated aldehydes were tested to investigate the generality of the reaction. The results are summarized in Table 3. Firstly, the effect of the substituents on the naphthol ring was investigated. Various 3-substituted 2-naphthols (OMe, OBn, Bn, 4-CF_3_C_6_H_4_ and OCH_2_CHCH_2_) were all tolerated well, and their corresponding annulation products were obtained in 46–90% yields and 84–90% ee (Table 3, entries 1–7). When 3-Br, 3-Ph and 3-PhCONH substituted 2-naphthols were used, relatively longer time was required to obtain the satisfactory results (Table 3, entries 8–10; 73–84% yields, 76–95% ee). When the 6-COOMe 2-naphthol was used, the annulation product was obtained in 86% yield and 83% ee (Table 3, entry 11), and the 7-OMe 2-naphthol gave excellent enantioselectivity with moderate yield (Table 3, entry 12; 62% yield, 91% ee). Next a wide range of substituted cinnamaldehydes bearing either an electron-donating or electron-withdrawing group were further tested. In all cases, the annulation proceeded smoothly to afford their corresponding products in good yields and enantioselectivity (Table 3, entries 15–23; 82–96% yields, 71–96% ee). Notably, (*E*)-3-(furan-2-yl)acrylaldehyde and (*E*)-but-2-enal were also suitable substrates, affording annulation products in 91% yield, 85% ee and 65% yield, 75% ee respectively (Table 3, entries 24–25).

**Table 3:** Substrate scope^*a*^

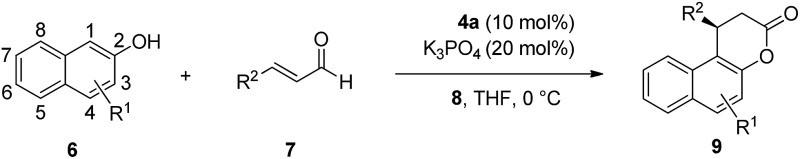						
Entry	R^1^	R^2^	**9**	Time (h)	Yield^*b*^ (%)	ee^*c*^ (%)
1	3-Me	4-MeOC_6_H_4_	**9a**	12	73	87
2	H	4-MeOC_6_H_4_	**9b**	11	46	88
3	3-OMe	4-MeOC_6_H_4_	**9c**	18	75	88
4	3-OBn	4-MeOC_6_H_4_	**9d**	4	79	90
5	3-OCH_2_CHCH_2_	4-MeOC_6_H_4_	**9e**	13	76	88
6	3-Bn	4-MeOC_6_H_4_	**9f**	5	90	84
7	3-(4-CF_3_C_6_H_4_)	4-MeOC_6_H_4_	**9g**	5.5	87	88
8	3-Ph	4-MeOC_6_H_4_	**9h**	24	84	95
9	3-Br	4-MeOC_6_H_4_	**9i**	24	73	82
10	3-PhCONH	4-MeOC_6_H_4_	**9j**	5 days	73	76
11	6-COOMe	4-MeOC_6_H_4_	**9k**	12	86	83
12	7-OMe	4-MeOC_6_H_4_	**9l**	12	62	91
13	H	Ph	**9m**	36	60	85
14	3-Me	Ph	**9n**	24	65	79
15	3-Ph	Ph	**9o**	8.5	82	91
16	3-Ph	2-MeC_6_H_4_	**9p**	36	89	96
17	3-Ph	2-MeOC_6_H_4_	**9q**	30	85	92
18	3-Ph	4-MeC_6_H_4_	**9r**	6	92	92
19	3-Ph	4-Me_2_NC_6_H_4_	**9s**	36	91	92
20	3-Ph	4-FC_6_H_4_	**9t**	1.5	94	86
21	3-Ph	4-ClC_6_H_4_	**9u**	2	95	85
22	3-Ph	4-BrC_6_H_4_	**9v**	6	96	85
23	3-Me	4-MeCO_2_C_6_H_4_	**9w**	5.5	82	71
24	3-Ph	2-Furyl	**9x**	3	91	85
25^*d*^	3-Ph	Me	**9y**	5 days	65	75

^*a*^Reaction conditions: **6** (0.2 mmol), **7** (0.2 mmol), **8** (0.2 mmol), **4a** (0.02 mmol), K_3_PO_4_ (0.04 mmol) in THF (2.0 mL) at 0 °C unless noted otherwise.

^*b*^Isolated yield for **9**.

^*c*^Determined by HPLC.

^*d*^At rt.

In order to determine the absolute configuration of the products, a crystal of enantiopure **9h** was obtained and X-ray crystallographic analysis determined its configuration as *R*.

To our great delight, when an ynal or 2-bromoenal was used, this annulation reaction occurred without external oxidant to give comparable results by running the reaction at room temperature (eqn (1) and (2)).1
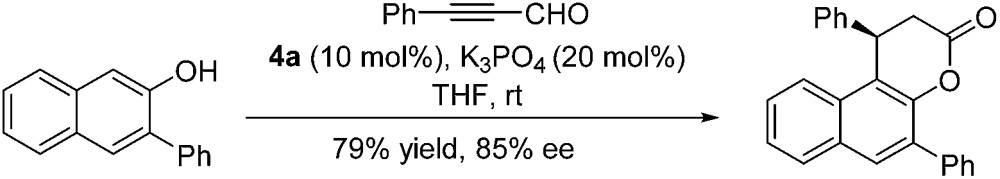

2
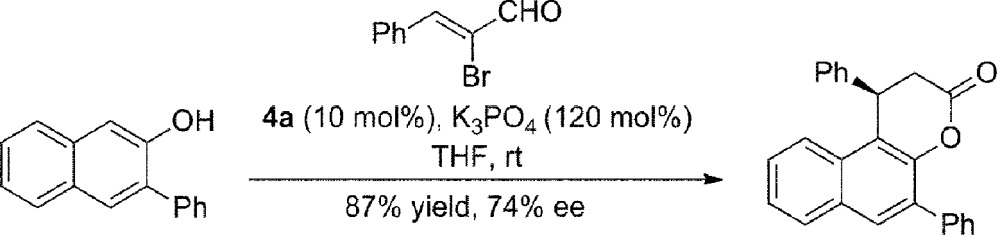



In order to shed light on the reaction mechanism,^16^ compound **10o** was synthesized and subjected to the identical reaction conditions. Product **9o** was isolated in 22% yield with 48% ee after 7 days at room temperature, along with **6h** in 44% yield (eqn (3)). In addition, real time monitoring of the reaction of **6a** with **7a** by ^1^H NMR showed that there was little change in the ratio of product **9a** to byproduct **10a** during the progress of the reaction, which suggested that an α,β-unsaturated acyl azolium intermediate is directly generated from the enal with the NHC and oxidant rather than from byproduct **10a** (see the ESI† for details).3
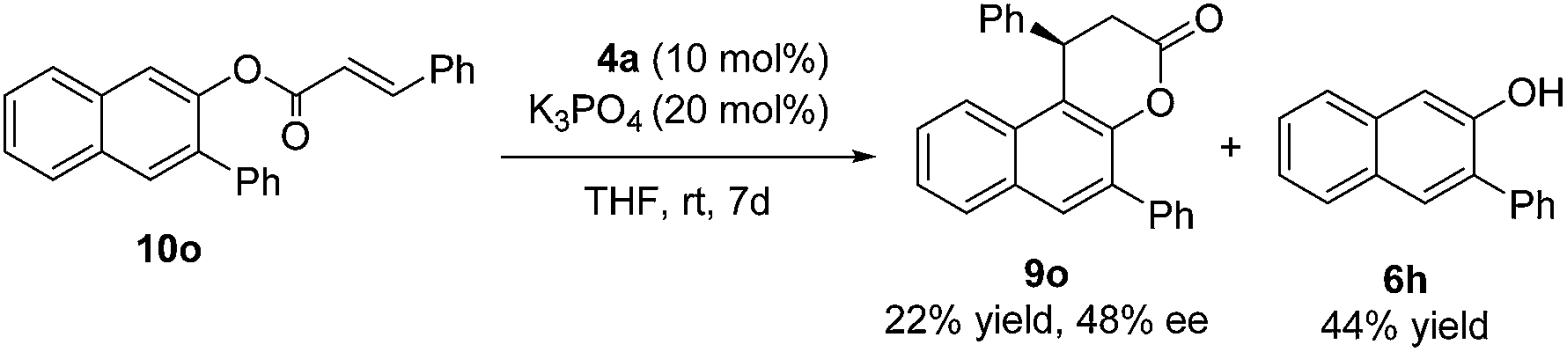



A gram-scale reaction between **6h** and **7a** was carried out to give the desired product in 85% yield and 93% ee without any loss of either yield or enantioselectivity, which further demonstrated the practicality of this methodology. Highly enantioenriched products obtained here can undergo diverse transformations. For example, the reduction of **9h** by DIBAL–H gave hemiacetal **11** in 96% yield and 92% ee. Ring opening of **9h** by dimethylamine or LiAlH_4_ led to amide **12** or diol **13** respectively in high yield without loss of enantiomeric purity (Scheme 2).

**Scheme 2:**
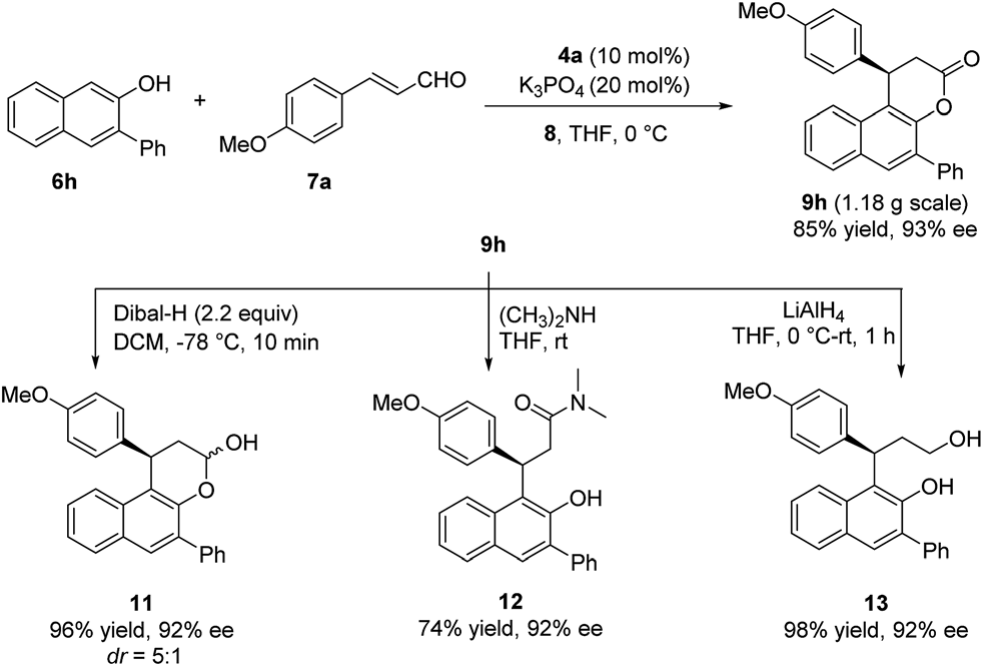
Gram-scale synthesis and transformations of the product.

## Conclusions

We have synthesized a series of novel chiral triazolium salts derived from l-phenylalanine. The NHC derived from **4a** is found to be a highly efficient catalyst for annulation of enals with 2-naphthols under oxidative conditions. Structurally diverse β-arylsplitomicins were formed in good yields with up to 96% ee. Understanding the excellent performance of these novel triazolium salts and further exploration of their application in asymmetric reactions are currently underway in our lab.

## Supplementary Material

Supplementary information

Crystal structure data
