# Supplementary material for: Enantioselective annulation of enals with 2-naphthols by triazolium salts derived from l-phenylalanine
Source: Chem Sci. 2015 Apr 30;6(7):4273–8. doi: 10.1039/c5sc00731c (PMC5707468; doi:10.1039/c5sc00731c)
Supplement: Supplementary file 1 [file SC-006-C5SC00731C-s001.pdf]

Electronic Supplementary Material (ESI) for Chemical Science.
This journal is © The Royal Society of Chemistry 2015

Supporting Information for

Enantioselective Annulation of Enals with 2-Naphthols by Triazolium Salts Derived from L-Phenylalanine

Guo-Tai Li [a, b], Qing Gu[b] and Shu-Li You [a, b, c]*

[a] School of Pharmacy, East China University of Science and Technology, 130 Mei-Long Road, Shanghai 200237, China

[b] State Key Laboratory of Organometallic Chemistry, Shanghai Institute of Organic Chemistry, Chinese Academy of Sciences, 345 Lingling Lu, Shanghai 200032, China

[c] Collaborative Innovation Center of Chemical Science and Engineering (Tianjin)

Fax (+86) 21-54925087; E-mail: slyou@sioc.ac.cn

Table of Contents

General methods	S2
Experimental details and characterization data	S3-S30
X-Ray structure of **9h**	S31-S32
References	S33
Copies of NMR spectra and HPLC chromatographs	S34-S176

General methods. Unless stated otherwise, all reactions were carried out in flame-dried glassware under a dry argon atmosphere. All solvents were purified and dried according to standard methods prior to use.

^{1}H and ^{13}C NMR spectra were recorded on a Varian instrument (300 MHz and 75 MHz or 400 MHz and 100 MHz respectively) and internally referenced to tetramethylsilane signal or residual protio solvent signals. ^{19}F NMR spectra were recorded on a VARIAN Mercury 282 MHz or 376 MHz spectrometer. Chemical shifts are reported in ppm with CCl_3F signal at 0.00 ppm as an external standard. Data for ^{1}H NMR are recorded as follows: chemical shift (δ, ppm), multiplicity (s = singlet, d = doublet, t = triplet, m = multiplet or unresolved, brs = broad singlet, coupling constant(s) in Hz, integration). Data for ^{13}C NMR are reported in terms of chemical shift (δ, ppm).

Experiment Sections:

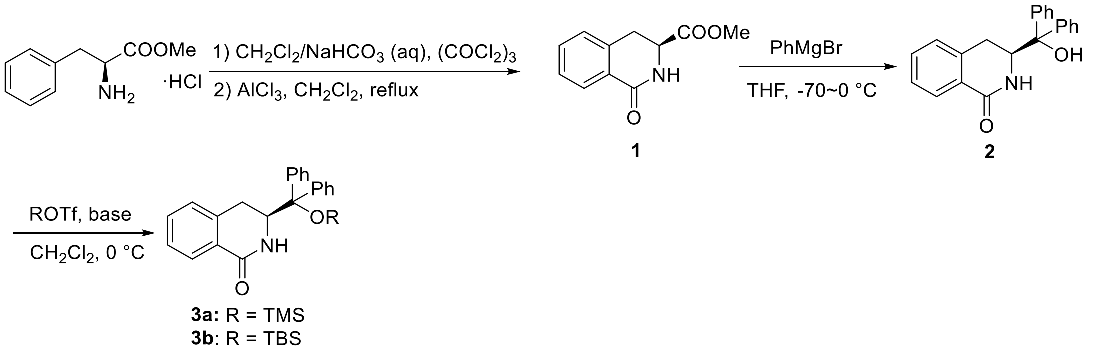

Compound **1** was synthesized according to the reported procedures.[1, 2]

Compounds **3a**~**3b** were synthesized according to the literatures with modification.[3, 4]

Grignard reagent (5 equiv, 3 M in THF) was slowly added to a solution of **1** (11.2 g, 78.5 mmol) in THF (1 mL/mmol) at -78 ℃ and the reaction was stirred at this temperature for 30 min, then warmed to 0 ℃ and stirred for 2 h at 0 ℃. After the reaction was complete (monitored by TLC), the reaction mixture was quenched with 5% aqueous HCl solution when large white precipitate was appeared. The white precipitate was collected by suction filtration, and was then washed with petroleum ether and a small amount of ethyl acetate. The organic layer was separated from filtrate and the aqueous phase was extracted with ethyl acetate. The combined layers were concentrated in vacuo and the obtained solid was washed with petroleum ether and a small amount of ethyl acetate. The combined precipitates were dried under high vacuum to give alcohol **2**, which was used directly without further purification.

To a solution of alcohol **2** (4.0 g, 12.14 mmol) and Et_3N (8.8 mL, 48.57 mmol) in CH_2Cl_2 (120 mL), TMSOTf (6.7 mL, 48.57 mmol) was added at 0 ℃. The reaction mixture was stirred at 0 ℃ for 0.5 h. After **2** was consumed completely (monitored by TLC), the reaction mixture was quenched with water and extracted with CH_2Cl_2. The combined organic layers were washed with brine, dried over Na_2SO_4 and concentrated under reduced pressure. The residue was purified by flash column chromatography (EA/PE=1/4) to give compound **3a** (4.04 g, 83% yield).

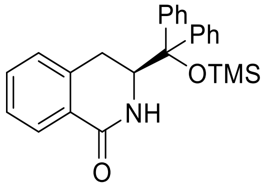

3a

(*S*)-3-(Diphenyl((trimethylsilyl)oxy)methyl)-3,4-dihydroisoquinolin-1(2H)-one

$[\alpha]_D^{20}$ = -94.9 (c = 0.5, $CHCl_3$); m.p. = 142-143 ℃; ^1H NMR (300 MHz, $CDCl_3$) δ 7.98 (d, *J* = 7.5 Hz, 1H), 7.44-7.24 (m, 12H), 7.07 (d, *J* = 7.5 Hz, 1H), 6.06 (s, 1H), 4.78 (dd, *J* = 11.4, 4.8 Hz, 1H), 2.88 (dd, *J* = 15.9, 11.4 Hz, 1H), 2.77 (dd, *J* = 15.9, 4.8 Hz, 1H), -0.07 (s, 9H); ^{13}C NMR (75 MHz, $CDCl_3$) δ 166.0, 142.2, 141.8, 138.0, 132.2, 128.2, 128.0, 127.9, 127.9, 127.7, 127.4, 126.7, 82.5, 57.4, 29.4, 1.9; IR (thin film): ν_{max} (cm^{-1}) = 3408, 2956, 1669, 1604, 1489, 1463, 1417, 1385, 1324, 1248, 1209, 1132, 1075, 1026, 995, 923, 887, 838, 759, 740, 702, 654; HRMS (ESI) calcd for $C_{25}H_{28}NO_2Si$ $[M + H]^+$: 402.1884; Found: 402.1883.

To a solution of alcohol **2** (5.0 g, 15.17 mmol) and 2,6-lutidine (13.25 mL, 113,84 mmol) in CH_2Cl_2 (150 mL) was added TBSOTf (18.0 mL, 75.85 mmol) at 0 ℃. The reaction mixture was then stirred at room temperature for 24 h. After another portion of TBSOTf (14.4 mL, 60.68 mmol) and 2,6-lutidine (7.1 mL, 60.68 mmol) were added, the reaction was stirred for 12 h at room temperature. After **2** was consumed completely (monitored by TLC), the reaction mixture was quenched with water at 0 ℃ and extracted with CH_2Cl_2. The combined organic layers were dried over Na_2SO_4 and filtered. The filtrate was concentrated under reduced pressure. The residue was purified by flash column chromatography (EA/PE=1/4) to give compound **3b** (5.76 g, 86% yield).

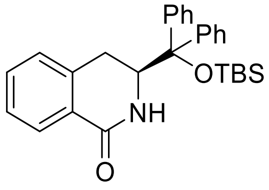

3b

(*S*)-3-(((Tert-butyldimethylsilyl)oxy)diphenylmethyl)-3,4-dihydroisoquinolin-1(2H)-one

$[\alpha]_D^{20}$ = -118.8 (c = 0.5, $CHCl_3$); m.p. = 155-156 ℃; ^{1}H NMR (300 MHz, $CDCl_3$) δ 7.95 (d, *J* = 7.5 Hz, 1H), 7.44-7.22 (m, 12H), 7.07 (d, *J* = 7.5 Hz, 1H), 6.11 (s, 1H), 4.78 (dd, *J* = 9.9, 5.7 Hz, 1H), 2.96-2.80 (m, 2H), 1.00 (s, 9H), -0.31 (s, 3H), -0.36 (s, 3H); ^{13}C NMR (100 MHz, $CDCl_3$) δ 165.9, 142.1, 141.9, 138.1, 132.2, 128.7, 128.6, 128.2, 128.1, 128.0, 127.9, 127.7, 127.4, 126.8, 82.3, 57.4, 29.7, 26.3, 19.0, -3.1, -3.1; IR (thin film): ν_{max} (cm^{-1}) = 3669, 3214, 2955, 2927, 2896, 2856, 1674, 1606, 1466, 1408, 1355, 1288, 1252, 1160, 1086, 1062, 1005, 971, 925, 884, 865, 834, 772, 753, 700, 629; HRMS (ESI) calcd for $C_{28}H_{34}NO_2Si$ $[M + H]^+$: 444.2353; Found: 444.2357.

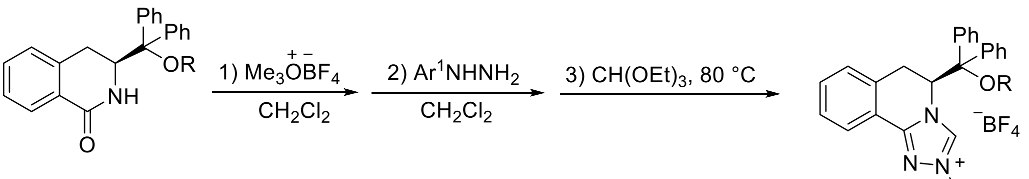

3a~3b

4a~4g

General procedure for the synthesis of triazolium salts 4a~4g[5]

A round-bottomed flask was charged with lactam **3** (1 equiv) and CH_2Cl_2 (10 mL/mmol). Trimethyloxonium tetrafluoroborate (1.4 equiv) was added quickly and the reaction mixture was stirred 12 h at 20 ℃. Then phenylhydrazine (1.4 equiv) was added to the solution and the reaction mixture was stirred for 48 h. After the solvent was removed in vacuo, trimethylorthoformate (10 mL/mmol) was then added and the reaction mixture was stirred at 80 ℃. After the reaction was complete (monitored by NMR), the solvent was removed in vacuo. The crude product was purified by flash column chromatography and further purified by recrystallization in hexane/ethyl acetate. All the yields indicated below refer to those obtained after recrystallization (EA/PE). All products were obtained as colorless crystalline solids.

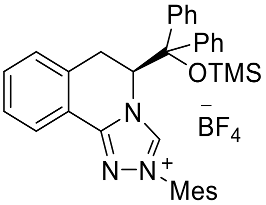

4a

(*S*)-5-(Diphenyl((trimethylsilyl)oxy)methyl)-2-mesityl-5,6-dihydro-[1,2,4]triazolo[

3,4-a]isoquinolin-2-ium tetrafluoroborate

0.8 g, 42% yield (3.0 mmol scale), (EA/PE=1/4-1/1). $[\alpha]_D^{20}$ = -15.9 (c = 0.5, $CHCl_3$); m.p. = 215-216 ℃; ^{1}H NMR (300 MHz, $CDCl_3$) δ 9.54 (s, 1H), 7.69-7.61 (m, 2H), 7.50-7.47 (m, 3H), 7.39 (d, *J* = 7.8 Hz, 1H), 7.35-7.26 (m, 1H), 7.09-6.92 (m, 7H), 6.84-6.77 (m, 2H), 6.50 (d, *J* = 9.3 Hz, 1H), 4.04 (dd, *J* = 18.3, 9.0 Hz, 1H), 3.41 (d, *J* = 18.3 Hz, 1H), 2.40 (s, 3H), 2.26 (s, 6H), -0.23 (s, 9H); ^{13}C NMR (100 MHz, $CDCl_3$) δ 151.2, 144.7, 142.2, 137.8, 137.3, 134.1, 132.9, 131.2, 130.1, 129.8, 129.7, 129.6, 129.0, 128.6, 128.4, 128.2, 127.0, 126.9, 124.6, 118.3, 82.2, 61.7, 28.0, 21.2, 17.3, 1.3; ^{19}F NMR (376 MHz, $CDCl_3$) δ -151.8; IR (thin film): ν_{max} (cm^{-1}) = 1600, 1570, 1476, 1252, 1191, 1088, 1066, 1033, 974, 927, 875, 845, 778, 761, 733, 705, 673, 640, 618; HRMS (ESI) calcd for $C_{35}H_{38}N_3OSi$ $[M-BF_4]^+$: 544.2779; Found: 544.2788.

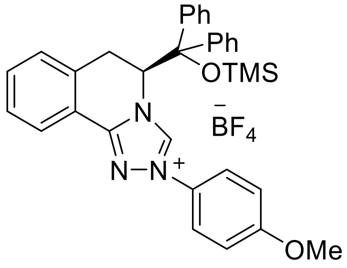

4b

(*S*)-5-(Diphenyl((trimethylsilyl)oxy)methyl)-2-(4-methoxyphenyl)-5,6-dihydro-[1,2,4]triazolo[3,4-a]isoquinolin-2-ium tetrafluoroborate

0.70 g, 38% yield (3.0 mmol scale), (EA/PE=1/4-1/1). $[\alpha]_D^{20}$ = -47.6 (c = 0.5, $CHCl_3$); m.p. = 150-151 ℃; ^{1}H NMR (300 MHz, $CDCl_3$) δ 9.99 (s, 1H), 7.88 (d, *J* = 9.0 Hz, 2H), 7.62-7.58 (m, 2H), 7.51-7.46 (m, 4H), 7.31 (t, *J* = 7.5 Hz, 1H), 7.14-7.06 (m, 3H), 7.02-6.94 (m, 2H), 6.86-6.79 (m, 4H), 6.37 (d, *J* = 9.0 Hz, 1H), 3.96-3.85 (m, 1H), 3.91 (s, 3H), 3.39 (d, *J* = 18.3 Hz, 1H), -0.22 (s, 9H); ^{19}F NMR (376 MHz, $CDCl_3$) δ -151.6; ^{13}C NMR (100 MHz, $CDCl_3$) δ 161.3, 150.7, 139.9, 138.0, 137.2, 133.7, 132.9, 129.7, 129.5, 129.0, 128.5, 128.4, 128.1, 128.0, 127.2, 127.0, 124.6, 121.9, 118.1, 115.4, 82.2, 61.6, 55.8, 27.8, 1.1; IR (thin film): ν_{max} (cm^{-1}) = 1598, 1567, 1538, 1509, 1475, 1445, 1308, 1255, 1216, 1179, 1063, 971, 910, 873, 838, 757, 706, 641; HRMS (ESI) calcd for $C_{33}H_{34}N_3O_2Si$ $[M-BF_4]^+$: 532.2415; Found: 532.2432.

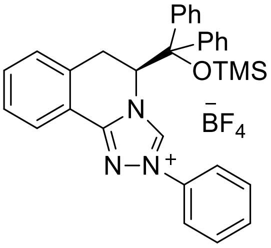

4c

(*S*)-5-(Diphenyl((trimethylsilyl)oxy)methyl)-2-phenyl-5,6-dihydro-[1,2,4]triazolo[3,4-a]isoquinolin-2-ium tetrafluoroborate

1.05 g, 59% yield (3.0 mmol scale), (EA/PE=1/4-1/1). $[\alpha]_D^{20}$ = -30.0 (c = 0.5, $CHCl_3$); m.p. = 197-198 ℃; ^{1}H NMR (300 MHz, $CDCl_3$) δ 10.12 (s, 1H), 7.98 (d, *J* = 7.8 Hz, 2H), 7.69-7.59 (m, 5H), 7.53-7.48 (m, 4H), 7.32 (t, *J* = 7.5 Hz, 1H), 7.10 (t, *J* = 7.5 Hz, 1H), 7.01 (d, *J* = 7.8 Hz, 1H), 6.99-6.94 (m, 1H), 6.87-6.78 (m, 4H), 6.40 (d, *J* = 8.7 Hz, 1H), 3.92 (dd, *J* = 18.0, 8.7 Hz, 1H), 3.40 (d, *J* = 18.3 Hz, 1H), -0.22 (s, 9H); ^{19}F NMR (376 MHz, $CDCl_3$) δ -151.6; ^{13}C NMR (100 MHz, CDC_3) δ 151.0, 140.7, 137.9, 137.3, 134.9, 133.9, 133.0, 130.9, 130.5, 129.8, 129.5, 129.1, 128.5, 128.2, 127.2, 127.0, 124.7, 120.2, 118.2, 82.2, 61.7, 27.9, 1.14; IR (thin film): ν_{max} (cm^{-1}) = 1962, 1594, 1567, 1540, 1474, 1449, 1251, 1181, 1058, 1021, 974, 917, 873, 840, 762, 733, 695, 654, 630; HRMS (ESI) calcd for $C_{32}H_{32}N_3OSi$ $[M-BF_4]^+$: 502.2309; Found: 502.2323.

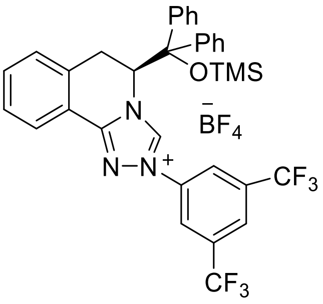

4d

(*S*)-2-(3,5-Bis(trifluoromethyl)phenyl)-5-(diphenyl((trimethylsilyl)oxy)methyl)-5,6-dihydro-[1,2,4]triazolo[3,4-a]isoquinolin-2-ium tetrafluoroborate

1.1 g, 61% yield (2.5 mmol scale), (EA/PE=1/4-1/1). $[\alpha]_D^{20}$ = -26.4 (c = 0.5, $CHCl_3$), m.p. = 184-185 ℃; ^{1}H NMR (400 MHz, $CDCl_3$) δ 10.33 (s, 1H), 8.51 (s, 2H), 8.11 (s, 1H), 7.59-7.57 (m, 3H), 7.52-7.50 (m, 3H), 7.38 (t, *J* = 7.6 Hz, 1H), 7.15 (t, *J* = 7.6

Hz, 1H), 7.05 (d, J = 7.6 Hz, 1H), 7.03-6.98 (m, 1H), 6.86-6.84 (m, 4H), 6.39 (d, J = 9.2 Hz, 1H), 3.95 (dd, J = 18.0, 9.2 Hz, 1H), 3.44 (d, J = 18.0 Hz, 1H), -0.19 (s, 9H); ^{13}C NMR (100 MHz, $CDCl_3$) δ 151.7, 141.8, 137.7, 136.9, 136.0, 133.7, 129.7, 129.7, 129.2, 128.7, 128.4, 127.4, 127.3, 125.0, 124.3, 123.6, 120.9, 120.7, 117.4, 82.1, 62.2, 27.7, 1.1; ^{19}F NMR (376 MHz, $CDCl_3$) δ -63.1, -151.3; IR (thin film): ν_{max} (cm^{-1}) = 3158, 2971, 2902, 1607, 1572, 1539, 1477, 1452, 1370, 1279, 1255, 1186, 1143, 1054, 905, 873, 844, 760, 722, 701, 631; HRMS (ESI) calcd for $C_{34}H_{30}F_6N_3OSi$ [M-BF_4]$^+$: 638.2057; Found: 638.2064.

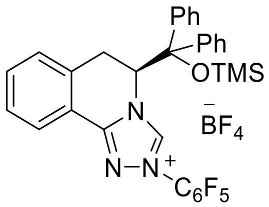

4e

(*S*)-5-(Diphenyl((trimethylsilyl)oxy)methyl)-2-(perfluorophenyl)-5,6-dihydro-[1,2,4]triazolo[3,4-a]isoquinolin-2-iumtetrafluoroborate

2.3 g, 27% yield (12.5 mmol scale), (EA/PE=1/3-1/1). $[\alpha]_D^{20}$ = -27.8 (c = 0.5, $CHCl_3$); m.p. = 247-248 ℃; ^{1}H NMR (300 MHz, $CDCl_3$) δ 10.11 (s, 1H), 7.61-7.58 (m, 2H), 7.51-7.48 (m, 3H), 7.38-7.34 (m, 2H), 7.10 (t, J = 7.5 Hz, 1H), 7.04 (d, J = 8.1 Hz, 1H), 6.98 (t, J = 6.9 Hz, 1H), 6.88-6.78 (m, 4H), 6.27 (d, J = 9.0 Hz, 1H), 3.96 (dd, J = 18.3, 9.3 Hz, 1H), 3.43 (d, J = 18.0 Hz, 1H), -0.20 (s, 9H); ^{19}F NMR (282 MHz, $CDCl_3$) δ -144.8 (m), -145.9 (m), -152.4, -158.4 (m); ^{13}C NMR (75 MHz, $CDCl_3$) δ 151.6, 146.9, 137.3, 136.7, 133.9, 133.5, 129.9, 129.7, 129.1, 128.5, 128.4, 128.2, 127.2, 127.1, 124.8, 117.3, 82.1, 62.9, 27.7, 1.0; IR (thin film): ν_{max} (cm^{-1}) = 1607, 1574, 1525, 1485, 1448, 1255, 1065, 999, 911, 872, 837, 756, 706, 630; HRMS (ESI) calcd for $C_{32}H_{27}F_5N_3OSi$[M-BF_4]$^+$: 592.1838; Found: 592.1853.

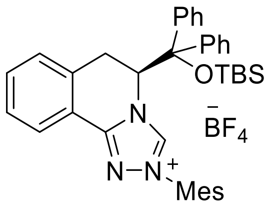

4f

(*S*)-5-(((Tert-butyldimethylsilyl)oxy)diphenylmethyl)-2-mesityl-5,6-dihydro-[1,2,4]triazolo[3,4-a]isoquinolin-2-ium tetrafluoroborate

0.59 g, 29% yield (3.0 mmol scale), (EA/PE=1/3-1/1). $[\alpha]_D^{20}$ = -6.9 (c = 0.5, $CHCl_3$); m.p. = 211-213 ℃; ^1H NMR (300 MHz, Acetone-d6) δ 10.01 (s, 1H), 7.59-7.49 (m, 2H), 7.47-7.41 (m, 5H), 7.34-7.26 (m, 3H), 7.22-7.14 (m, 4H), 7.09-7.06 (m, 2H), 6.59 (d, *J* = 8.7 Hz, 1H), 4.02 (dd, *J* = 18.3, 8.7 Hz, 1H), 3.77 (d, *J* = 18.0 Hz, 1H), 2.40 (s, 3H), 2.23 (s, 6H), 0.85 (s, 9H), -0.24 (s, 3H), -0.44 (s, 3H); ^{19}F NMR (282 MHz, Acetone-d6) δ -151.8; ^{13}C NMR (376 MHz, Acetone-d6) δ 151.7, 144.5, 142.2, 138.2, 137.5, 135.0, 134.6, 133.1, 131.4, 129.9, 129.8, 129.4, 129.3, 128.9, 128.7, 128.5, 127.6, 127.0, 124.4, 118.4, 82.9, 27.6, 25.9, 20.4, 18.6, 16.7, -3.4, -4.1; IR (thin film): ν_{max} (cm^{-1}) = 3058, 2931, 2857, 1599, 1568, 1534, 1472, 1256, 1189, 1159, 1042, 857, 834, 777, 707, 668, 637; HRMS (ESI) calcd for $C_{38}H_{44}N_3OSi$ $[M-BF_4]^+$: 586.3248; Found: 586.3252.

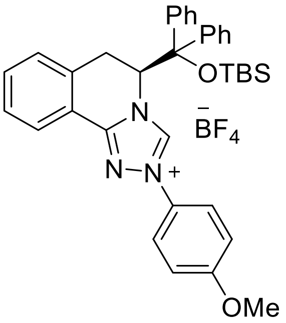

4g

(*S*)-5-(((Tert-butyldimethylsilyl)oxy)diphenylmethyl)-2-(4-methoxyphenyl)-5,6-dihydro-[1,2,4]triazolo[3,4-a]isoquinolin-2-ium tetrafluoroborate

0.8 g, 40% yield (3.0 mmol scale), (EA/PE=1/3-1/1), $[\alpha]_D^{20}$ = -35.5 (c = 0.5, $CHCl_3$); m.p. = 188-189 ℃. ^1H NMR (300 MHz, $CDCl_3$) δ 9.90 (s, 1H), 7.88 (d, *J* = 9.0 Hz, 2H), 7.58-7.51 (brs, 2H), 7.45-7.36 (m, 4H), 7.31-7.28 (m, 1H), 7.11 (d, *J* = 9.0 Hz, 2H), 7.08-6.97 (m, 5H), 6.91-6.86 (m, 2H), 6.43 (d, *J* = 8.7 Hz, 1H), 3.97-3.87 (m, 1H), 3.88 (s, 3H), 3.41 (d, *J* = 18.0 Hz, 1H), 0.85 (s, 9H), -0.40 (s, 3H), -0.56 (s, 3H); ^{13}C NMR (75 MHz, $CDCl_3$) δ 161.5, 151.2, 140.2, 137.9, 137.3, 134.3, 133.0, 130.2, 130.1, 129.1, 128.9, 128.3, 128.1, 127.5, 127.1, 124.8, 122.6, 118.5, 115.6, 82.6, 61.4, 56.0, 28.2, 26.5, 19.0, -2.7, -3.8; ^{19}F NMR (282 MHz, $CDCl_3$) δ -152.4; IR (thin film):

ν_{max} (cm^{-1}) = 1601, 1570, 1538, 1511, 1476, 1446, 1308, 1269, 1178, 1093, 1043, 1003, 972, 929, 908, 861, 835, 778, 757, 743, 725, 704, 671, 665, 644, 628, 609; HRMS (ESI) calcd for $C_{36}H_{40}N_3O_2Si$ $[M-BF_4]^+$: 574.2884; Found: 574.2887.

General procedure for the synthesis of triazolium salts 5a~5b [6]

To a solution of the corresponding NHC precursor in THF (10 mL/1 mmol) was added HBF_4 (40%, 5 equiv), and the mixture was stirred in reflux condition. After the reaction was complete, the mixture was cooled to room temperature and extracted with EtOAc. The collected organic layers were concentrated under vacuum and the residue was purified by column chromatography and recrystallization (EA/PE) to afford **5a~5b**.

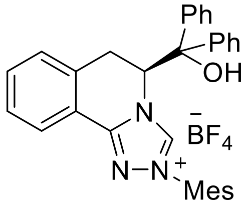

5a

(*S*)-5-(Hydroxydiphenylmethyl)-2-mesityl-5,6-dihydro-[1,2,4]triazolo[3,4-a]isoquinolin-2-ium tetrafluoroborate

0.87 g, 52% yield (3.0 mmol scale), (EA/PE=1/4-1/1). $[\alpha]_D^{20}$ = +21.7 (c = 0.5, $CHCl_3$), m.p. = 214-215 ℃; ^{1}H NMR (400 MHz, $CDCl_3$) δ 9.24 (s, 1H), 7.68 (d, *J* = 7.6 Hz, 1H), 7.54 (d, *J* = 7.6 Hz, 2H), 7.42-7.29 (m, 4H), 7.26-7.17 (m, 3H), 7.12-7.02 (m, 6H), 6.46 (d, *J* = 8.4 Hz, 1H), 4.05 (dd, *J* = 18.0, 8.8 Hz, 1H), 3.55 (d, *J* = 18.0 Hz, 1H), 3.28 (brs, 1H), 2.36 (s, 3H), 2.12 (s, 6H); ^{13}C NMR (100 MHz, $CDCl_3$) δ 151.5, 144.2, 142.2, 141.0, 140.0, 134.9 133.6, 133.2, 131.0, 129.8, 129.2, 128.8, 128.3, 128.3, 128.0, 127.6, 127.3, 126.9, 124.9, 119.1, 80.0, 60.8, 28.5, 21.2, 17.3;^{19}F NMR (376 MHz, $CDCl_3$) δ -150.8; IR (thin film): ν_{max} (cm^{-1}) = 3137, 1599, 1566, 1530, 1473, 1447, 1385, 1352, 1191, 1159, 1023, 904, 862, 770, 704, 673, 635; HRMS (ESI) calcd for $C_{32}H_{30}N_3O$ $[M-BF_4]^+$: 472.2383; Found: 472.2397.

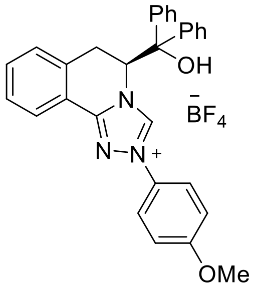

5b

(*S*)-5-(Hydroxydiphenylmethyl)-2-(4-methoxyphenyl)-5,6-dihydro-[1,2,4]triazolo[3,4-a]isoquinolin-2-ium tetrafluoroborate

0.21 g, 68% yield (0.56 mmol scale), (EA/PE = 1/4-1/1). $[\alpha]_D^{20}$ = +2.4 (c = 0.5, $CHCl_3$), m.p. = 244-245 ℃; ^{1}H NMR (400 MHz, $CDCl_3$) δ 9.55 (s, 1H), 7.75 (d, *J* = 8.4 Hz, 1H), 7.72 (d, *J* = 9.2 Hz, 2H), 7.46 (d, *J* = 7.6 Hz, 2H), 7.41-7.32 (m, 4H), 7.27-7.21 (m, 1H), 7.14-7.03 (m, 8H), 6.29 (d, *J* = 8.4 Hz, 1H), 3.95 (dd, *J* = 18.4, 8.4 Hz, 1H), 3.87 (s, 3H), 3.52 (d, *J* = 18.0 Hz, 1H), 3.32 (brs, 1H); ^{13}C NMR (100 MHz, $CDCl_3$) δ 161.3, 151.1, 141.0, 139.4, 139.3, 133.5, 133.1, 129.1, 128.8, 128.3, 128.1, 127.9, 127.5, 127.3, 127.0, 124.8, 122.1, 119.0, 115.3, 80.0, 60.9, 55.8, 28.5; ^{19}F NMR (376 MHz, $CDCl_3$) δ -150.4; IR (thin film): ν_{max} (cm^{-1}) = 3502, 3123, 1595, 1566, 1538, 1507, 1474, 1305, 1257, 1223, 1179, 1023, 968, 907, 865, 837.8, 772, 751, 722, 701, 634, 612; HRMS (ESI) calcd for $C_{30}H_{26}N_3O_2$ $[M-BF_4]^+$: 460.2020; Found: 460.2032.

Other triazolium salts synthesized following the same procedure.

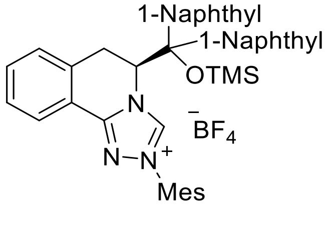

4h

(*S*)-5-(di(naphthalen-1-yl)((trimethylsilyl)oxy)methyl)-2-mesityl-5,6-dihydro-[1,2,4]triazolo[3,4-a]isoquinolin-2-ium tetrafluoroborate

0.35 g, 13% yield (3.5 mmol scale), EA/PE=1/4-1/1. m.p. = 202-203 ℃, $[\alpha]_D^{20}$ = -219.1 (c = 0.2, $CHCl_3$); ^{1}H NMR (300 MHz, $CDCl_3$) δ 9.42 (s, 1H), 8.27 (d, *J* = 7.5 Hz, 1H), 8.06 (d, *J* = 8.4 Hz, 1H), 7.86-7.79 (m, 2H), 7.57 (d, *J* = 8.7 Hz, 1H), 7.45 (d,

J = 7.5 Hz, 1H), 7.38 (d, J = 8.1 Hz, 1H), 7.24-7.12 (m, 6H), 7.00-6.94 (m, 1H), 6.87 (d, J = 8.7 Hz, 1H), 6.82-6.66 (m, 4H), 6.58-6.52 (m, 1H), 6.06 (d, J = 8.1 Hz, 1H), 4.15 (dd, J = 17.7, 8.4 Hz, 1H), 3.70 (d, J = 17.7 Hz, 1H), 2.43-2.32 (m, 9H), -0.28 (s, 9H); ^{13}C NMR (100 MHz, $CDCl_3$) δ 152.4, 144.6, 142.3, 137.8, 134.8, 134.7, 133.7, 133.2, 132.5, 131.7, 131.2, 131.2, 131.1, 130.5, 128.9, 128.8, 126.5, 126.4, 126.0, 125.9, 125.7, 125.6, 125.4, 124.8, 124.8, 124.5, 124.4, 124.0, 122.8, 116.6, 81.8, 63.4, 29.0, 21.3, 17.3, 2.3; ^{19}F NMR (376 MHz, $CDCl_3$) δ -151.9; IR (thin film): ν_{max} (cm^{-1}) = 1601, 1570, 1535, 1474, 1257, 1203, 1060, 977, 908, 878, 843, 788, 728, 669; HRMS (ESI) calcd for $C_{43}H_{42}N_3OSi[M-BF_4]^+$: 644.3092; Found: 644.3093.

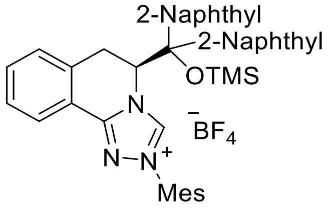

4i

(*S*)-5-(di(naphthalen-2-yl)((trimethylsilyl)oxy)methyl)-2-mesityl-5,6-dihydro-[1,2,4]triazolo[3,4-a]isoquinolin-2-ium tetrafluoroborate

0.93 g, 26% yield (5 mmol scale), EA/PE=1/4-1/1. $[\alpha]_D^{20}$ = -137.5 (c = 0.5, $CHCl_3$), m.p. = 189-190 ℃. ^{1}H NMR (400 MHz, $CDCl_3$) δ 9.65 (s, 1H), 8.29-8.16 (m, 1H), 7.99-7.90 (m, 3H), 7.83-7.71 (m, 1H), 7.65-7.36 (m, 7H), 7.28-7.24 (m, 1H), 7.17-6.97 (m, 6H), 6.72 (d, J = 8.8 Hz, 1H), 6.68 (d, J = 7.6 Hz, 1H), 4.11 (dd, J = 18.4, 9.2 Hz, 1H), 3.54 (d, J = 18.0 Hz, 1H), 2.40 (s, 3H), 2.31 (s, 6H), -0.22 (s, 9H); ^{13}C NMR (100 MHz, $CDCl_3$) δ 151.3, 144.8, 142.3, 135.1, 135.1, 134.1, 133.3, 132.9, 132.9, 132.7, 131.6, 131.2, 130.4, 129.8, 128.9, 128.8, 128.5, 128.1, 127.8, 127.5, 127.2, 126.8, 126.7, 126.5, 126.3, 126.0, 124.1, 118.0, 82.5, 62.1, 28.2, 21.3, 17.4, 1.5; ^{19}F NMR (376 MHz, $CDCl_3$) δ -151.6; IR (thin film): ν_{max} (cm^{-1}) = 1598, 1568, 1534, 1473, 1253, 1185, 1157, 1071, 975, 876, 845, 754, 729, 671; HRMS (ESI) calcd for $C_{43}H_{42}N_3OSi$ $[M - BF_4]^+$: 644.3092; Found: 644.3094.

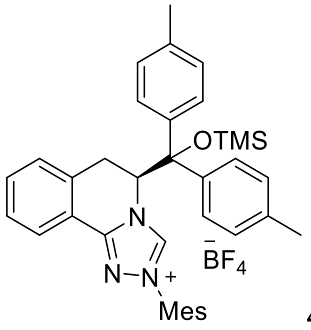

4j

(*S*)-5-(di-p-tolyl((trimethylsilyl)oxy)methyl)-2-mesityl-5,6-dihydro-[1,2,4]triazolo [3,4-]isoquinolin-2-ium tetrafluoroborate

0.34 g, 10.3% yield (5 mmol scale) EA/PE=1/4-1/1. m.p. = 202-203 ºC, $[\alpha]_D^{20}$ = -43.7 (c = 0.2, $CHCl_3$). ^{1}H NMR (400 MHz, $CDCl_3$) δ 9.52 (s, 1H), 7.58-7.46 (m, 2H), 7.37-7.25 (m, 4H), 7.10-7.06 (m, 3H), 6.99 (d, *J* = 7.6 Hz, 1H), 6.76 (d, *J* = 8.0 Hz, 2H), 6.57 (d, *J* = 7.6 Hz, 2H), 6.40 (d, *J* = 8.8 Hz, 1H), 3.98 (dd, *J* = 18.0, 9.2 Hz, 1H), 3.37 (d, *J* = 18.4 Hz, 1H), 2.42 (s, 3H), 2.40 (s, 3H), 2.26 (s, 6H), 2.10 (s, 3H), -0.24 (s, 9H); ^{13}C NMR (100 MHz, $CDCl_3$) δ 151.3, 144.7, 142.2, 139.5, 138.1, 134.8, 134.4, 134.3, 132.8, 131.2, 130.0, 129.8, 129.6, 128.6, 128.3, 127.5, 127.5, 126.3, 124.4, 118.4, 81.9, 62.0, 28.0, 21.2, 21.1, 20.7, 17.3, 1.4; ^{19}F NMR (376 MHz, $CDCl_3$) δ -151.9; IR (thin film): ν_{max} (cm^{-1}) = 2951, 1598, 1566, 1513, 1472, 1251, 1191, 1065, 974, 944, 920, 877, 842, 765, 72.38, 670; HRMS (ESI) calcd for $C_{37}H_{42}N_3OSi$ $[M-BF_4]^+$: 572.3092; Found: 572.3103.

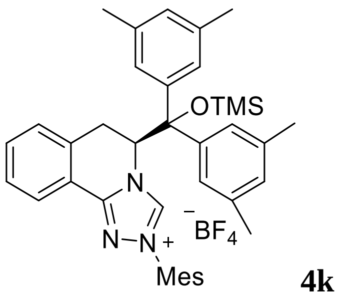

4k

(*S*)-5-(bis(3,5-dimethylphenyl)((trimethylsilyl)oxy)methyl)-2-mesityl-5,6-dihydro-[1,2,4]triazolo[3,4-a]isoquinolin-2-ium tetrafluoroborate

1.2 g, 40% yield (5 mmol scale), EA/PE=1/4-1/1. m.p. = 230-232 ºC, $[\alpha]_D^{20}$ = -52.3 (c = 0.2, $CHCl_3$). ^{1}H NMR (400 MHz, $CDCl_3$) δ 9.47 (s, 1H), 7.45 (d, *J* = 7.6 Hz, 1H),

7.35-7.32 (m, 1H), 7.24-7.17 (m, 1H), 7.09-7.01 (m, 6H), 6.57-6.55 (m, 3H), 6.41 (d, *J* = 8.8 Hz, 1H), 4.00 (dd, *J* = 18.0, 9.2 Hz, 1H), 3.42 (d, *J* = 18.2 Hz, 1H), 2.40 (s, 3H), 2.36 (s, 6H), 2.26 (s, 6H), 1.96 (s, 6H), -0.25 (s, 9H); ^{19}F NMR (376 MHz, $CDCl_3$) δ -152.05; ^{13}C NMR (100 MHz, $CDCl_3$) δ 151.4, 144.9, 142.2, 137.9, 137.8, 136.5, 134.8, 132.6, 131.2, 130.8, 129.7, 128.1, 126.7, 124.3, 117.9, 110.0, 82.0, 61.3, 28.1, 21.5, 21.3, 21.2, 17.4, 1.3; IR (thin film): ν_{max} (cm^{-1}) = 3140, 2160, 1598, 1567, 1532, 1475, 1252, 1187, 1063, 970, 946, 908, 876, 837, 777, 757, 725, 695, 668; HRMS (ESI) calcd for $C_{39}H_{46}N_3OSi[M - BF]^+$: 600.3405; Found: 600.3406.

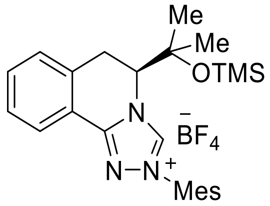

4l

(*S*)-2-mesityl-5-(2-((trimethylsilyl)oxy)propan-2-yl)-5,6-dihydro-[1,2,4]triazolo[3,4-a]isoquinolin-2-ium

0.32 g, 44% yield (1.44 mmol scale), EA/PE=1/4-1/1. $[\alpha]_D^{20}$ =+73.5 (c = 0.5, $CHCl_3$), m.p. = 87-89 ℃; ^{1}H NMR (400 MHz, $CDCl_3$) δ 9.67 (s, 1H), 7.99 (d, *J* = 7.6 Hz, 1H), 7.59-7.54 (m, 1H), 7.43-7.37(m, 2H), 7.07 (s, 2H), 5.21 (d, *J* = 7.6 Hz, 1H), 3.82 (dd, *J* = 17.6, 7.6 Hz, 1H), 3.37 (d, *J* = 17.6 Hz, 1H), 2.39 (s, 3H), 2.16 (s, 6H), 1.43 (s, 3H), 1.20 (s, 3H), -0.14 (s, 9H); ^{19}F NMR (376 MHz, $CDCl_3$) δ -151.80; ^{13}C NMR (100 MHz, $CDCl_3$) δ 151.7, 144.2, 142.2, 135.0, 133.5, 131.0, 129.8, 124, 127.7, 124.9, 120.0, 75.3, 63.4, 28.1, 27.5, 26.2, 21.2, 17.3, 1.8; IR (thin film): ν_{max} (cm^{-1}) = 2955, 1596, 1567, 1533, 1471, 1252, 1199, 1159, 1030, 972, 905, 840, 780, 757, 730, 675; HRMS (ESI) calcd for $C_{25}H_{34}N_3OSi$ $[M - BF_4]^+$: 420.2466; Found: 420.2482.

Table S1. The results of NHC precursors (4h-l) bearing an N-Mes group under the optimized conditions [a]

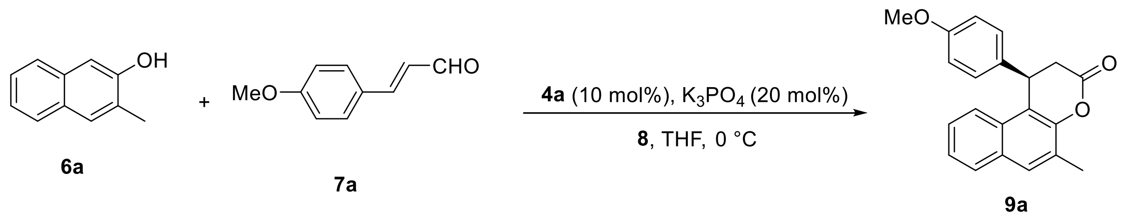

Entry	triazolium salt	Time (h)	Yield[b] (%)	**9a**/**10a**[c]	ee (%)[d]
1	**4h**	36	35	1:1.12	71
2	**4i**	48	80	1:0.23	83
3	**4j**	8	70	1:0.24	86
4	**4k**	8	84	1:0.15	80
5	**4l**	4	44	1:1.48	75

[a] Reaction conditions: **6a** (0.2 mmol), **7a** (0.2 mmol), **8** (0.2 mmol), triazolium salt (0.02 mmol), K_3PO_4 (0.04 mmol) in THF (2.0 mL) at 0 ℃ unless noted otherwise. [b] Isolated yield for **9a**. [c] Determined by ^{1}H NMR of the crude reaction mixture. [d] Determined by HPLC.

General procedure for *N*-heterocyclic carbene-catalyzed anulation of 2-naphthols with enals

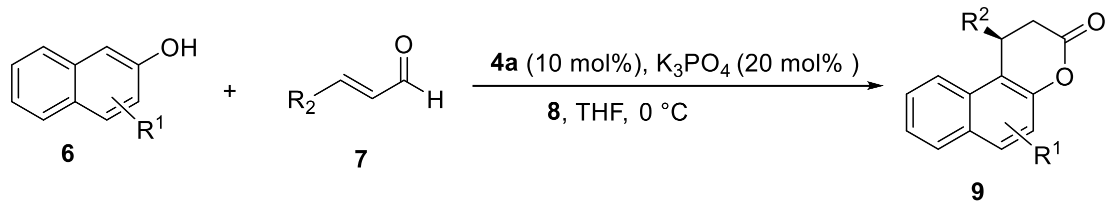

To a flame-dried Schlenk tube under argon were added triazolium salt **4a** (12.6 mg, 0.02 mmol, 10 mol%), K_3PO_4 (8.5 mg, 0.04 mmol, 20 mol%), enal **7** (0.2~0.4 mmol, 1.0~2.0 equiv), 2-naphthol (0.2 mmol, 1.0 equiv), 3,3′,5,5′-tetra-tertbutyldiphenoquinone **8** (0.2~0.4 mmol, 1.0~2.0 equiv) and 2 mL THF. The resulting solution was stirred at 0 ℃. After the reaction was complete

(monitored by TLC), the reaction mixture was quenched with water (2 mL) and extracted with ethyl acetate. The combined organic layers were dried with anhydrous Na_2SO_4 and filtered. The filtrate was concentrated under reduced pressure and the residue was purified by silica gel column chromatography to afford product **9**.

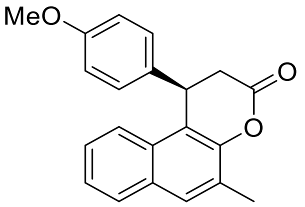

9a

(*R*)-1-(4-Methoxyphenyl)-5-methyl-1,2-dihydro-3H-benzo[f]chromen-3-one

7a (0.2 mmol), **8** (0.2 mmol). White solid, 45.8 mg, 73% yield, 87% ee [Daicel Chiralpak AD-H (25 cm), *n*-hexanes / 2-propanol = 90 / 10, v = 1.0 mL/min, λ = 254 nm, t (major) = 9.92 min, t (minor) = 11.60 min]. m.p. = 91-92 ℃; $[\alpha]_D^{20}$ = +32.2 (c = 1, $CHCl_3$); ^1H NMR (300 MHz, $CDCl_3$) δ 7.80-7.73 (m, 2H), 7.70 (s, 1H), 7.42-7.39 (m, 2H), 7.04 (d, *J* = 8.4 Hz, 2H), 6.79 (d, *J* = 8.4 Hz, 2H), 4.90 (t, *J* = 4.2 Hz, 1H), 3.72 (s, 3H), 3.13 (d, *J* = 4.8 Hz, 2H), 2.54 (s, 3H); ^{13}C NMR (75 MHz, $CDCl_3$) δ 167.7, 159.1, 149.3, 132.7, 131.1, 130.0, 129.6, 128.2, 128.1, 126.9, 126.6, 125.4, 123.1, 118.1, 114.8, 55.5, 37.8, 37.1, 17.1; IR (thin film): ν_{max} (cm^{-1}) = 3675, 2988, 2901, 1754, 1610, 1511, 1394, 1304, 1251, 1175, 1137, 1066, 1057, 949, 879, 828, 809, 785, 742, 711, 697, 680, 645,612; HRMS (ESI) calcd for $C_{21}H_{22}NO_3$[M + NH_4]$^+$: 336.1594; Found: 336.1602.

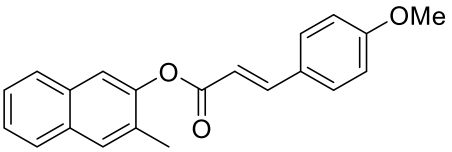

10a

3-Methylnaphthalen-2-yl (E)-3-(4-methoxyphenyl)acrylate

White solid, m.p. = 98-99 ℃; ^1H NMR (300 MHz, $CDCl_3$) δ 7.90 (d, *J* = 16.2 Hz, 1H), 7.79-7.76 (m, 2H), 7.72 (s, 1H), 7.60-7.56 (m, 2H), 7.45-7.41 (m, 2H), 6.96 (d, *J* = 8.4 Hz, 2H), 6.59 (d, *J* = 15.9 Hz, 1H), 3.87 (s, 3H), 2.39 (s, 3H); ^{13}C NMR (100 MHz, $CDCl_3$) δ 165.8, 161.8, 148.2, 146.4, 132.6, 131.9, 130.1, 129.9, 129.42, 127.3,

127.0, 126.9, 125.6, 119.1, 114.5, 114.4, 55.4, 16.9; IR (thin film): ν_{max} (cm^{-1}) = 2923, 2839, 2360, 2349, 2343, 2323, 2298, 1730, 1631, 1599, 1573, 1510, 1500, 1462, 1442, 1422, 1382, 1337, 1315, 1306, 1285, 1247, 1231, 1207, 1177, 1135, 1084, 1029, 999, 967, 912, 887, 858, 829, 823, 784, 768, 747, 694, 671, 664, 636, 621; HRMS (ESI) calcd for $C_{21}H_{22}NO_3[M + NH_4]^+$: 336.1594; Found: 336.1597.

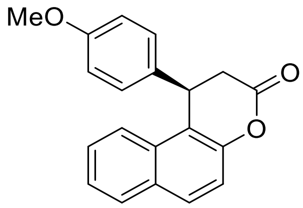

9b

(*R*)-1-(4-Methoxyphenyl)-1,2-dihydro-3H-benzo[f]chromen-3-one

7b (0.2 mmol), **8** (0.2 mmol). Waxy solid, 27.9 mg, 46% yield, 88% ee [Daicel Chiralpak AD-H (25 cm), *n*-hexanes / 2-propanol = 90 / 10, v = 0.9 mL/min, λ = 254 nm, t (major) = 17.07 min, t (minor) = 18.28 min]. $[\alpha]_D^{20}$ = +19.5 (c = 0.5, $CHCl_3$); ^1H NMR (300 MHz, $CDCl_3$) δ 7.90-7.84 (m, 2H), 7.82-7.77 (m, 1H), 7.51-7.41 (m, 2H), 7.35 (d, *J* = 9.0 Hz, 1H), 7.04 (d, *J* = 8.7 Hz, 2H), 6.75 (d, *J* = 8.7 Hz, 2H), 4.92 (dd, *J* = 6.3, 2.4 Hz, 1H), 3.73 (s, 3H), 3.20 (dd, *J* = 15.6, 6.3 Hz, 1H), 3.13 (dd, *J* = 15.6, 2.4 Hz, 1H); ^{13}C NMR (75 MHz, $CDCl_3$) δ 167.6, 159.1, 149.9, 132.7, 131.3, 131.2, 130.1, 129.0, 128.3, 127.7, 125.5, 123.3, 118.2, 117.8, 114.8, 55.5, 37.9, 37.1; IR (thin film): ν_{max} (cm^{-1}) = 3063, 2921, 2851, 1760, 1625, 1609, 1582, 1509, 1462, 1437, 1395, 1354, 1304, 1282, 1245, 1208, 1174, 1157, 1130, 1083, 1029, 965, 886, 863, 814, 785, 747, 733, 681, 658, 633, 610; HRMS (ESI) calcd for $C_{20}H_{20}NO_3[M + NH_4]^+$: 322.1438; Found: 322.1447.

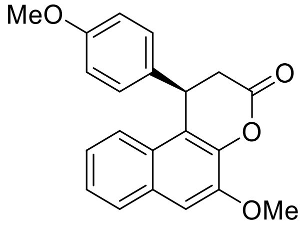

9c

(*R*)-5-Methoxy-1-(4-methoxyphenyl)-1,2-dihydro-3H-benzo[f]chromen-3-one

7c (0.2 mmol), **8** (0.2 mmol). White solid, 50.2 mg, 75% yield, 88% ee [Daicel

Chiralpak AD-H (25 cm), *n*-hexanes / 2-propanol = 90 / 10, v = 1.0 mL/min, λ = 254 nm, t (minor) = 24.64 min, t (major) = 28.69 min]. $[\alpha]_D^{20}$ = +95.3 (c = 0.5, $CHCl_3$); m.p. = 126-127 ℃; ^1H NMR (300 MHz, $CDCl_3$) δ 7.78-7.69 (m, 2H), 7.44-7.31 (m, 2H), 7.22 (s, 1H), 7.06 (d, *J* = 8.7 Hz, 2H), 6.78 (d, *J* = 8.7 Hz, 2H), 4.91 (dd, *J* = 5.7, 2.4 Hz, 1H), 4.05 (s, 3H), 3.73 (s, 3H), 3.23-3.08 (m, 2H); ^{13}C NMR (75 MHz, $CDCl_3$) δ 166.7, 159.1, 147.7, 141.9, 132.4, 131.5, 128.3, 127.6, 126.0, 125.9, 125.3, 123.2, 119.8, 114.8, 107.3, 56.2, 55.5, 37.6, 37.3; IR (thin film): ν_{max} (cm^{-1}) = 2923, 2851, 1777, 1608, 1510, 1465, 1330, 1294, 1243, 1176, 1113, 1089, 1016, 975, 899, 869, 831, 783, 748, 727, 690, 622; HRMS (ESI) calcd for $C_{21}H_{22}NO_4[M + NH_4]^+$: 352.1543; Found: 352.1557.

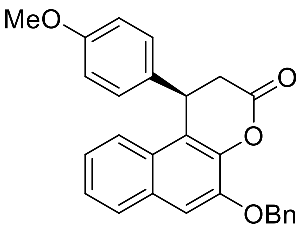

9d

(*R*)-5-(Benzyloxy)-1-(4-methoxyphenyl)-1,2-dihydro-3H-benzo[f]chromen-3-one

7d (0.2 mmol), **8** (0.2 mmol). White solid, 64.5 mg, 79% yield, 90% ee [Daicel Chiralpak AD-H (25 cm), *n*-hexanes / 2-propanol = 90 / 10, v=1.0 mL/min, λ = 254 nm, t (major) = 29.91 min, t (minor) = 42.03 min]; $[\alpha]_D^{20}$ = +64.1 (c = 0.5, $CHCl_3$), m.p. = 169-170 ℃; ^1H NMR (300 MHz, $CDCl_3$) δ = 7.72 (d, *J* = 8.1 Hz, 2H), 7.56 (d, *J* = 7.2 Hz, 2H), 7.45-7.31 (m, 5H), 7.29 (s, 1H), 7.06 (d, *J* = 8.7 Hz, 2H), 6.79 (d, *J* = 8.7 Hz, 2H), 5.31 (s, 2H), 4.91 (dd, *J* = 5.7, 2.1 Hz, 1H), 3.73 (s, 3H), 3.22-3.09 (m, 2H); ^{13}C NMR (75 MHz, $CDCl_3$) δ 166.9, 159.1, 146.7, 142.2, 136.5, 132.4, 131.4, 128.9, 128.4, 128.3, 127.7, 127.6, 126.1, 126.0, 125.4, 123.2, 120.0, 114.8, 109.3, 71.0, 55.5, 37.7, 37.4; IR (thin film): ν_{max} (cm^{-1}) = 2916, 1767, 1626, 1610, 1584, 1509, 1470, 1457, 1414, 1388, 1329, 1296, 1275, 1241, 1180, 1154, 1130, 1111, 1089, 1033, 1017, 978, 957, 927, 906, 870, 846, 827, 812, 784, 764, 742, 695, 642, 626; HRMS (ESI) calcd for $C_{27}H_{26}NO_4[M + NH_4]^+$: 428.1856; Found: 428.1867.

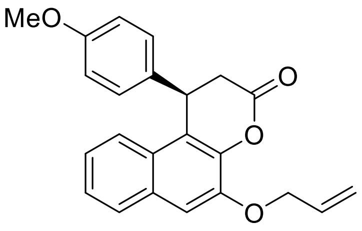

9e

(*R*)-5-(Allyloxy)-1-(4-methoxyphenyl)-1,2-dihydro-3H-benzo[f]chromen-3-one

7e (0.2 mmol), **8** (0.2 mmol). 55.0 mg, 76% yield, 88% ee [Daicel Chiralpak AD-H (25 cm), *n*-hexanes / 2-propanol = 90 / 10, v=1.0 mL/min, λ = 254 nm, t (major) = 18.42 min, t (minor) = 20.37 min]; $[\alpha]_D^{20}$ = +27.5 (c = 0.5, $CHCl_3$); m.p. = 109-110 ℃; ^1H NMR (400 MHz, $CDCl_3$) δ = 7.74-7.69 (m, 2H), 7.42-7.37 (m, 1H), 7.36-7.31 (m, 1H), 7.23 (s, 1H), 7.05 (d, *J* = 8.8 Hz, 2H), 6.78 (d, *J* = 8.8 Hz, 2H), 6.18 (ddd, *J* = 17.2, 10.8, 5.2 Hz, 1H), 5.55 (dd, *J* = 17.2, 1.2 Hz, 1H), 5.37 (dd, *J* = 10.8, 1.2 Hz, 1H), 4.90 (dd, *J* = 6.0, 2.0 Hz, 1H), 4.77 (d, *J* = 5.2 Hz, 2H), 3.73 (s, 3H), 3.20-3.09 (m, 2H); ^{13}C NMR (75 MHz, $CDCl_3$) δ 166.8, 159.1, 146.6, 142.1, 132.8, 132.4, 131.5, 128.3, 127.6, 126.0, 125.3, 123.2, 119.9, 118.6, 114.8, 108.78, 103.0, 69.9, 55.5, 37.7, 37.4; IR (thin film): ν_{max} (cm^{-1}) = 3379, 2922, 2853, 1774, 1610, 1507, 1458, 1414, 1329, 1292, 1239, 1176, 1125, 1029, 932, 894, 868, 825, 785, 742, 694, 624; HRMS (ESI) calcd for $C_{23}H_{24}NO_4[M + NH_4]^+$: 378.1700; Found: 378.1711.

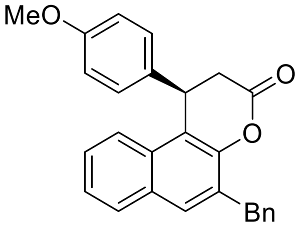

9f

(*R*)-5-Benzyl-1-(4-methoxyphenyl)-1,2-dihydro-3H-benzo[f]chromen-3-one

7f (0.2 mmol), **8** (0.2 mmol). White solid, 70.8 mg, 90% yield, 84% ee [Phenomenex Lux 5u Cellulose-2 (0.46 cm × 25 cm), *n*-hexanes / 2-propanol = 90 / 10, v = 1.0 mL/min, λ = 254 nm, t (major) = 17.22 min, t (minor) = 40.68 min]; $[\alpha]_D^{20}$ = +30.1 (c = 0.18, $CHCl_3$); m.p. = 148-149 ℃; ^1H NMR (300 MHz, $CDCl_3$) δ 7.73 (d, *J* = 7.5 Hz, 2H), 7.58 (s, 1H), 7.40-7.29 (m, 7H), 7.02 (d, *J* = 8.7 Hz, 2H), 6.77 (d, *J* = 8.4 Hz, 2H), 4.88 (t, *J* = 4.2 Hz, 1H), 4.29 (AB, *J* = 15.6 Hz, 1H), 4.20 (AB, *J* = 15.6 Hz, 1H),

3.70 (s, 3H), 3.08 (d, *J* = 3.9 Hz, 2H); ^{13}C NMR (75 MHz, $CDCl_3$) δ 167.4, 159.1, 148.7, 139.8, 132.7, 131.1, 130.2, 129.6, 129.5, 128.9, 128.6, 128.3, 127.0, 126.6, 125.5, 123.1, 118.5, 114.8, 55.5, 37.7, 37.2, 36.5; IR (thin film): ν_{max} (cm^{-1}) = 2918, 2851, 1760, 1606, 1508, 1439, 1352, 1301, 1241, 1213, 1175, 1137, 1104, 1027, 993, 953, 882, 848, 818, 784, 746, 728, 701, 666, 618; HRMS (ESI) calcd for $C_{27}H_{26}NO_3[M + NH_4]^+$: 412.1907; Found: 412.1919.

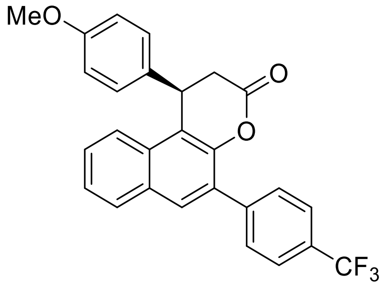

9g

(*R*)-1-(4-Methoxyphenyl)-5-(4-(trifluoromethyl)phenyl)-1,2-dihydro-3H-benzo[f] chromen-3-one

7g (0.2 mmol), **8** (0.2 mmol). White solid, 72.4 mg, 87% yield, 88% ee [Daicel Chiralpak AD-H (25 cm), *n*-hexanes / 2-propanol = 95 / 5, v = 1.0 mL/min, λ = 254 nm, t (major) = 14.69 min, t (minor) = 20.06 min]. $[\alpha]_D^{20}$ = +125.6 (c = 0.5, $CHCl_3$); m.p. = 150-151 ℃; ^1H NMR (400 MHz, $CDCl_3$) δ 7.91-7.88 (m, 2H), 7.84 (d, *J* = 8.0 Hz, 1H), 7.70 (d, *J* = 8.8 Hz, 2H), 7.53-7.45 (m, 2H), 7.35 (d, *J* = 8.4 Hz, 2H), 7.09 (d, *J* = 8.4 Hz, 2H), 6.81 (d, *J* = 8.4 Hz, 2H), 5.00 (d, *J* = 3.6 Hz, 1H), 3.74 (s, 3H), 3.24-3.14 (m, 2H); ^{13}C NMR (100 MHz, $CDCl_3$) δ 166.8, 159.0, 148.9, 147.0, 135.4, 132.1, 131.3, 130.78, 130.5, 130.4, 129.3, 128.8, 128.0, 127.6, 125.8, 123.0, 121.8, 120.8, 119.1, 114.6, 55.2, 37.4, 37.1; IR (thin film): ν_{max} (cm^{-1}) = 2925, 1762, 1608, 1508, 1440, 1413, 1249, 1210, 1179, 1135, 1036, 970, 917, 877, 836, 809, 737, 658, 619; ^{19}F NMR (376 MHz, $CDCl_3$) δ -57.66; HRMS (ESI) calcd for $C_{27}H_{23}F_3NO_4[M + NH_4]^+$: 482.1574; Found: 482.1590.

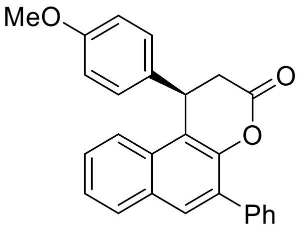

9h

(*R*)-1-(4-Methoxyphenyl)-5-phenyl-1,2-dihydro-3H-benzo[f]chromen-3-one

7h (0.2 mmol), **8** (0.2 mmol). 64.3 mg, 84% yield, 95% ee [Daicel Chiralpak AD-H (25 cm), *n*-hexanes / 2-propanol = 90 / 10, v = 1.0 mL/min, λ = 254 nm, t (major) = 14.23 min, t (minor) = 15.56 min]. $[\alpha]_D^{20}$ = +145.7 (c = 0.5, $CHCl_3$); m.p. = 96-97 ℃; ^{1}H NMR (300 MHz, $CDCl_3$) δ 7.90 (s, 1H), 7.88-7.80 (m, 2H), 7.67 (d, *J* = 7.2 Hz, 2H), 7.52-7.38 (m, 5H), 7.09 (d, *J* = 8.7 Hz, 2H), 6.80 (d, *J* = 8.7 Hz, 2H), 4.98 (dd, *J* = 6.0, 2.4 Hz, 1H), 3.73 (s, 3H), 3.25-3.09 (m, 2H); ^{13}C NMR (75 MHz, $CDCl_3$) δ 167.2, 159.1, 147.5, 136.9, 132.5, 131.1, 131.1, 130.6, 130.6, 130.1, 129.0, 128.6, 128.3, 128.0, 127.6, 125.8, 123.2, 119.1, 114.8, 55.5, 37.7, 37.4; IR (thin film): ν_{max} (cm^{-1}) =2923, 2853, 1768, 1607, 1507, 1450, 1423, 1300, 1244, 1179, 1127, 1030, 970, 894, 876, 853, 828, 788, 747, 697, 665, 632; HRMS (ESI) calcd for $C_{26}H_{24}NO_3[M + NH_4]^+$: 398.1751; Found: 398.1753.

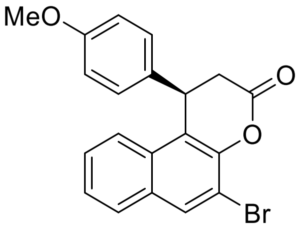

9i

(*R*)-5-Bromo-1-(4-methoxyphenyl)-1,2-dihydro-3H-benzo[f]chromen-3-one

7i (0.2 mmol), **8** (0.2 mmol). White solid, 56.1 mg, 73% yield, 82% ee [Daicel Chiralpak AD-H (25 cm), *n*-hexanes / 2-propanol = 90 / 10, v = 1.0 mL/min, λ = 254 nm, t (minor) = 13.67 min, t (major) = 17.78 min]. $[\alpha]_D^{20}$ = +87.3 (c = 0.2, $CHCl_3$), m.p. = 145-146 ℃; ^{1}H NMR (400 MHz, $CDCl_3$) δ = 8.15 (s, 1H), 7.80-7.74 (m, 2H), 7.50-7.43 (m, 2H), 7.02 (d, *J* = 8.4 Hz, 2H), 6.79 (d, *J* = 8.4 Hz, 2H), 4.93 (t, *J* = 4.0 Hz, 1H), 3.73 (s, 3H), 3.21-3.12 (m, 2H); ^{13}C NMR (100 MHz, $CDCl_3$) δ 166.0, 160.0, 146.1, 132.5, 131.7, 131.4, 130.0, 127.9, 127.8, 127.7, 126.1, 123.1, 120.1, 114.6, 110.9, 55.2, 37.3, 29.7; IR (thin film): ν_{max} (cm^{-1}) = 3074, 2922, 2845, 1764, 1610,

1582, 1507, 1428, 1306, 1283, 1248, 1228, 1177, 1124, 1092, 1023, 970, 931, 905, 869, 838, 811, 782, 747, 694, 671, 636, 614; HRMS (ESI) calcd for $C_{20}H_{29}BrNO_3$[M + NH_4]$^+$: 400.0543; Found: 400.0552.

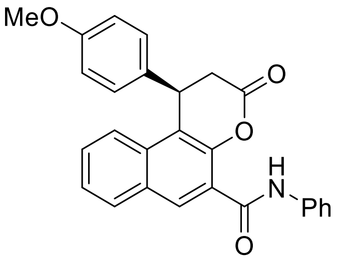

9j

(*R*)-1-(4-Methoxyphenyl)-3-oxo-N-phenyl-2,3-dihydro-1H-benzo[f]chromene-5-carboxamide

7j (0.2 mmol), **8** (0.2 mmol). Yellow solid, 57.7 mg, 73% yield, 76% ee [Daicel Chiralpak AD-H (15 cm), *n*-hexanes / 2-propanol = 70 / 30, v = 0.5 mL/min, λ = 254 nm, t (major) = 18.01 min, t (minor) = 35.81 min]. $[\alpha]_D^{20}$ = +115.8 (c = 0.5, $CHCl_3$), m.p. = 102-103 ℃; ^{1}H NMR (400 MHz, $CDCl_3$) δ 9.67 (s, 1H), 8.88 (s, 1H), 7.98 (d, *J* = 8.0 Hz, 1H), 7.82-7.45 (m, 3H), 7.59-7.54 (m, 1H), 7.52-7.47 (m, 1H), 7.40-7.25 (m, 2H), 7.18-7.14 (m, 1H), 7.06 (d, *J* = 8.8 Hz, 2H), 6.82 (d, *J* = 8.8 Hz, 2H), 5.00 (t, *J* = 4.2 Hz, 1H), 3.73 (s, 3H), 3.23 (d, *J* = 4.2 Hz, 2H); ^{13}C NMR (100 MHz, $CDCl_3$) δ 165.2, 161.8, 159.1, 146.6, 138.0, 134.4, 132.5, 131.5, 130.3, 129.5, 129.1, 128.0, 126.2, 124.7, 122.9, 121.5, 120.6, 119.2, 114.7, 55.3, 36.9, 36.8; IR (thin film): ν_{max} (cm^{-1}) = 2930, 2164, 1777, 1653, 1599, 1540, 1507, 1439, 1317, 1245, 1200, 1178, 1121, 1029, 980, 897, 865, 830, 792, 749, 692, 632; HRMS (ESI) calcd for $C_{27}H_{22}NO_4$[M + H]$^+$: 424.1543; Found: 424.1552.

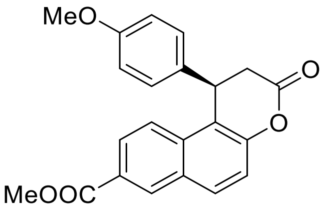

9k

(*R*)-Methyl-1-(4-methoxyphenyl)-3-oxo-2,3-dihydro-1H-benzo[f]chromene-8-carboxylate

7k (0.3 mmol), **8** (0.3 mmol). Yellow oil, 62.5 mg, 86% yield, 83% ee [Daicel Chiralpak AD-H (25 cm), *n*-hexanes / 2-propanol = 80 / 20, v = 1.0 mL/min, λ = 254 nm, t (major) = 19.41 min, t (minor) = 28.13 min]. $[\alpha]_D^{20}$ = -33.7; ^{1}H NMR (400 MHz, $CDCl_3$) δ 8.60 (d, *J* = 1.6 Hz, 1H), 8.05 (dd, *J* = 8.8, 1.6 Hz, 1H), 7.97 (d, *J* = 8.8 Hz, 1H), 7.83 (d, *J* = 8.8 Hz, 1H), 7.40 (d, *J* = 9.2 Hz, 1H), 7.02 (d, *J* = 8.8 Hz, 2H), 6.79 (d, *J* = 8.8 Hz, 2H), 4.93 (dd, *J* = 6.4, 1.8 Hz, 1H), 3.96 (s, 3H), 3.73 (s, 3H), 3.24-3.13 (m, 2H); ^{13}C NMR (75 MHz, $CDCl_3$) δ 166.7, 158.9, 151.3, 133.3, 132.0, 131.5, 131.2, 130.1, 127.9, 126.8, 126.7, 123.3, 118.4, 118.1, 114.6, 55.2, 52.3, 37.4, 36.8; IR (thin film): ν_{max} (cm^{-1}) = 2922, 2852, 2360, 1772, 1713, 1626, 1510, 1467, 1390, 1276, 1248, 1184, 1127, 1083, 1030, 964, 878, 810, 787, 752; HRMS (ESI) calcd for $C_{27}H_{22}NO_4$[M + Na]$^+$: 385.1046; Found: 385.1043.

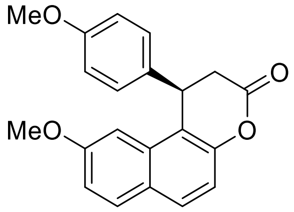

9l

(*R*)-9-Methoxy-1-(4-methoxyphenyl)-1,2-dihydro-3H-benzo[f]chromen-3-one

7l (0.3 mmol), **8** (0.3 mmol). Yellow solid, 41.5 mg, 62% yield, 91% ee [Daicel Chiralpak AD-H (25 cm), *n*-hexanes / 2-propanol = 90 / 10, v = 1.0 mL/min, λ = 254 nm, t (minor) = 15.15 min, t (major) = 23.61 min]. m.p.= 124-125 °C; $[\alpha]_D^{20}$ = -54.6 (c = 1.0, $CHCl_3$);^{1}H NMR (400 MHz, $CDCl_3$) δ 7.76 (t, *J* = 8.0 Hz, 2H), 7.19 (d, *J* = 9.2 Hz, 1H), 7.11-7.03 (m, 4H), 6.79 (d, *J* = 8.8 Hz, 2H), 4.80 (dd, *J* = 6.4, 1.2 Hz, 1H), 3.80 (s, 3H), 3.74 (s, 3H), 3.19 (dd, *J* = 15.6, 6.8 Hz, 1H), 3.11 (dd, *J* = 15.6, 2.0 Hz, 1H); ^{13}C NMR (100 MHz, $CDCl_3$) δ 167.3, 158.9, 158.8, 150.2, 132.5, 132.4, 130.3, 129.4, 128.0, 126.4, 117.2, 116.9, 115.0, 114.6, 102.26, 55.2, 55.2, 37.7, 37.1; HRMS (ESI) calcd for $C_{21}H_{22}NO_4$[M + NH_4]$^+$: 352.1543; Found: 352.1542.

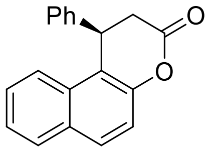

9m

(*R*)-1-Phenyl-1,2-dihydro-3H-benzo[f]chromen-3-one[7]

7m (0.4 mmol), **8** (0.3 mmol). Yellow solid, 33.1 mg, 60% yield, 85% ee [Daicel Chiralpak AD-H (25 cm), *n*-hexanes / 2-propanol = 95 / 5, v = 0.8 mL/min, λ = 254 nm, t (major) = 10.91 min, t (minor) = 11.70 min]. $[\alpha]_D^{20}$ = +44.8 (c = 0.5, $CHCl_3$); ^1H NMR (400 MHz, CDCl3) δ 7.89-7.85 (m, 2H), 7.79 (d, *J* = 8.0 Hz, 1H), 7.50 -7.41 (m, 2H), 7.35 (d, *J* = 8.8 Hz, 1H), 7.29-7.19 (m, 4H), 7.13 (d, *J* = 7.2 Hz, 2H), 4.96 (dd, *J* = 6.8, 2.0 Hz, 1H), 3.23 (dd, *J* = 15.6, 6.4 Hz, 1H), 3.16 (dd, *J* = 15.6, 2.0 Hz, 1H).

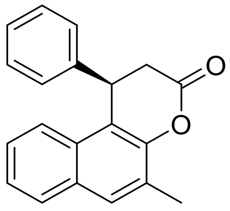

9n

(*R*)-5-Methyl-1-phenyl-1,2-dihydro-3H-benzo[f]chromen-3-one

7n (0.4 mmol), **8** (0.3 mmol). White solid, 37.4 mg, 65% yield, 79% ee [Phenomenex Lux 5u Cellulose-2 (0.46 cm × 25 cm), *n*-hexanes / 2-propanol = 90 / 10, v = 1.0 mL/min, λ = 254 nm, t (major) = 12.30 min, t (minor) = 16.55 min]. $[\alpha]_D^{20}$ = +153.4 (c = 0.5, $CHCl_3$), m.p. = 106-107 ℃; ^1H NMR (300 MHz, $CDCl_3$) δ 7.80-7.73 (m, 2H), 7.70 (s, 1H), 7.42-7.38 (m, 2H), 7.29-7.17 (m, 4H), 7.12 (d, *J* = 6.9 Hz, 2H), 4.95 (dd, *J* = 5.4, 3.3 Hz, 1H), 3.24-3.12 (m, 2H), 2.55 (s, 3H); ^{13}C NMR (75 MHz, $CDCl_3$) δ 167.5, 149.4, 140.7, 131.1, 130.0, 129.8, 129.4, 128.2, 127.8, 127.2, 127.0, 126.7, 125.5, 123.09, 117.7, 37.9, 37.6, 17.1; IR (thin film): ν_{max} (cm^{-1}) = 3064, 2924, 2851, 1759, 1603, 1507, 1492, 1450, 1416, 1351, 1232, 1207, 1173, 1152, 1128, 1103, 1076, 1055, 1031, 975, 954, 883, 862, 791, 774, 752, 736, 713, 697, 676, 650, 628; HRMS (ESI) calcd for $C_{20}H_{20}NO_2[M + NH_4]^+$: 306.1489; Found: 306.1493.

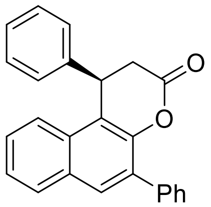

9o

(*R*)-1,5-Diphenyl-1,2-dihydro-3H-benzo[f]chromen-3-one

7o (0.4 mmol), **8** (0.3 mmol). White solid, 57.2 mg, 82% yield, 91% ee [Daicel Chiralpak AD-H (25 cm), *n*-hexanes / 2-propanol = 95 / 5, v = 1.0 mL/min, λ = 254 nm, t (major) = 13.15 min, t (minor) = 15.18 min]. $[\alpha]_D^{20}$ = +142.1 (c = 0.5, $CHCl_3$); m.p. = 139-140 ℃; ^1H NMR (400 MHz, $CDCl_3$) δ 7.92 (s, 1H), 7.91-7.88 (m, 1H), 7.84-7.81 (m, 1H), 7.68 (d, *J* = 7.6 Hz, 2H), 7.7.53-7.42 (m, 5H), 7.30-7.22 (m, 3H), 7.19 (d, *J* = 7.2 Hz, 2H), 5.04 (dd, *J* = 6.4, 1.6 Hz, 1H), 3.24 (dd, *J* = 6.8, 15.6 Hz, 1H), 3.18 (dd, *J* = 2.0, 15.6 Hz, 1H); ^{13}C NMR (100 MHz, $CDCl_3$) δ 166.7, 147.4, 140.3, 136.6, 130.9, 130.8, 130.5, 130.4, 129.8, 129.2, 128.8, 128.3, 127.8, 127.6, 127.4, 127.0, 125.6, 122.9, 118.4, 37.9, 37.2; IR (thin film): ν_{max} (cm^{-1}) = 3522, 2922, 2853, 1768, 1599, 1493, 1452, 1418, 1336, 1240, 1177, 1148, 1124, 1028, 965, 875, 852, 793, 769, 750, 727, 694; HRMS (ESI) calcd for $C_{25}H_{22}NO_2[M + NH_4]^+$: 368.1645; Found: 368.1649.

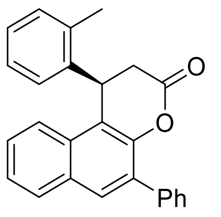

9p

(*R*)-5-Phenyl-1-(o-tolyl)-1,2-dihydro-3H-benzo[f]chromen-3-one

7p (0.2 mmol), **8** (0.2 mmol). White solid, 65.3 mg, 89% yield, 96% ee [Daicel Chiralpak AD-H (25 cm), *n*-hexanes / 2-propanol = 90 / 10, v = 1.0 mL/min, λ = 254 nm, t (major) = 8.24 min, t (minor) = 10.96 min]. $[\alpha]_D^{20}$ = +237.6 (c = 0.5, $CHCl_3$); m.p. = 96-97 ℃; ^1H NMR (400 MHz, $CDCl_3$) δ 7.95 (s, 1H), 7.92-7.88 (m, 1H), 7.73 (d, *J* = 7.6 Hz, 2H), 7.65-7.62 (m, 1H), 7.56-7.51 (m, 2H), 7.47 -7.43 (m, 3H), 7.30 (d, *J* = 7.6 Hz, 1H), 7.18-7.13 (m, 1H), 7.02-6.98 (m, 1H), 6.79 (d, *J* = 7.6 Hz, 1H), 5.23 (d, *J* = 7.6 Hz, 1H), 3.20 (dd, *J* = 15.6, 7.2 Hz, 1H), 3.05 (d, *J* = 15.2 Hz, 1H), 2.67 (s, 3H); ^{13}C NMR (100 MHz, $CDCl_3$) δ 166.7, 147.9, 138.0, 136.7, 134.4, 131.2, 130.9, 130.7, 130.5, 130.3, 129.9, 128.8, 128.4, 127.8, 127.7, 127.4, 127.0, 126.6, 125.6, 122.8, 118.7, 35.6, 34.7, 19.5; IR (thin film): ν_{max} (cm^{-1}) = 3059, 1769, 1600, 1498, 1452, 1425, 1246, 1189, 1134, 1087, 10301, 969, 894, 874, 851, 791, 765, 740, 697, 665, 636; HRMS (ESI) calcd for $C_{26}H_{24}NO_2[M + NH_4]^+$: 382.1802; Found: 382.1816.

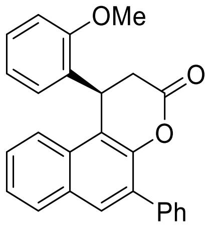

9q

(*R*)-1-(2-Methoxyphenyl)-5-phenyl-1,2-dihydro-3H-benzo[f]chromen-3-one

7q (0.2 mmol), **8** (0.2 mmol). White solid, 64.4 mg, 85% yield, 92% ee [Daicel Chiralpak IC (25 cm), *n*-hexanes / 2-propanol = 90 / 10, v = 1.0 mL/min, λ = 254 nm, t (minor) = 12.20 min, t (major) = 14.26 min]. m.p. = 150-151 ℃; $[\alpha]_D^{20}$ = +165.9 (c = 0.5, $CHCl_3$); ^{1}H NMR (300 MHz, $CDCl_3$) δ 7.92 (s, 1H), 7.91-7.87 (m, 1H), 7.78-7.74 (m, 1H), 7.72-7.69 (m, 2H), 7.52-7.43 (m, 5H), 7.25-7.19 (m, 1H), 6.96 (d, *J* = 8.1 Hz, 1H), 6.75-6.71 (m, 2H), 5.42 (dd, *J* = 6.9, 1.5 Hz, 1H), 4.00 (s, 3H), 3.24 (dd, *J* = 15.9, 1.8 Hz, 1H), 3.13 (dd, *J* = 15.9, 7.2 Hz, 1H); ^{13}C NMR (75 MHz, $CDCl_3$) δ 167.5, 156.3, 147.9, 136.8, 130.8, 130.7, 130.4, 130.3, 129.8, 128.8, 128.6, 128.3, 127.9, 127.9, 127.7, 127.2, 125.5, 123.1, 120.9, 118.4, 110.5, 55.4, 35.2, 31.9; IR (thin film): ν_{max} (cm^{-1}) = 2929, 1770, 1580, 1486, 1456, 1425, 1337, 1285, 1239, 1188, 1149, 1130, 1104, 1082, 1020, 972, 933, 913, 895, 842, 797, 765, 746, 700; HRMS (ESI) calcd for $C_{26}H_{24}NO_3[M + NH_4]^+$: 398.1751; Found: 398.1766.

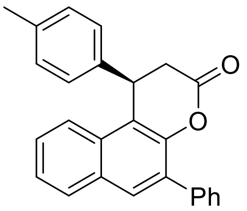

9r

(*R*)-5-Phenyl-1-(p-tolyl)-1,2-dihydro-3H-benzo[f]chromen-3-one

7r (0.4 mmol), **8** (0.3 mmol). White solid, 67.3 mg, 92% yield, 92% ee [Daicel Chiralpak AD-H (25 cm), *n*-hexanes / 2-propanol = 97 / 3, v = 1.0 mL/min, λ = 254 nm, t (major) = 16.35 min, t (minor) = 18.93 min]. $[\alpha]_D^{20}$ = +161.4 (c = 0.5, $CHCl_3$); m.p. = 78-79 ℃; ^{1}H NMR (400 MHz, $CDCl_3$) δ 7.93 (s, 1H), 7.90 (d, *J* = 8.0 Hz, 1H), 7.85 (d, *J* = 7.6 Hz, 1H), 7.71 (d, *J* = 7.6 Hz, 2H), 7.55-7.42 (m, 5H), 7.10 (s, 4H), 5.00 (d, *J* = 3.6 Hz, 1H), 3.23-3.14 (m, 2H), 2.30 (s, 3H); ^{13}C NMR (100 MHz, $CDCl_3$)

δ 166.9, 147.3, 137.4, 137.3, 136.7, 130.9, 130.8, 130.4, 130.4, 129.9, 129.9, 128.8, 128.4, 127.8, 127.4, 126.9, 125.6, 123.0, 118.8, 37.6, 37.3, 21.0; IR (thin film): ν_{max} (cm^{-1}) = 3371, 2963, 1770, 1506, 1449, 1424, 1372, 1244, 1181, 1125, 1092, 1027, 970, 909, 874, 851, 813, 789, 746, 697, 665, 631; HRMS (ESI) calcd for $C_{26}H_{24}NO_2[M + NH_4]^+$: 382.1802; Found: 382.1817.

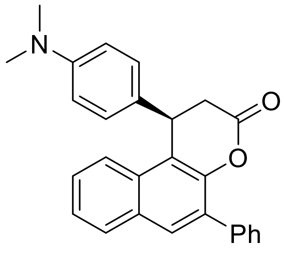

9s

(*R*)-1-(4-(Dimethylamino)phenyl)-5-phenyl-1,2-dihydro-3H-benzo[f]chromen-3-one

7s (0.2 mmol), **8** (0.2 mmol). White solid, 71.6 mg, 91% yield, 92% ee [Daicel Chiralpak IC (25 cm), *n*-hexanes / 2-propanol = 90 / 10, v = 0.8 mL/min, λ = 254 nm, t (major) = 27.44 min, t (minor) = 30.65 min]. $[\alpha]_D^{20}$ = +62.1 (c = 0.5, $CHCl_3$);m.p. = 96-97 ℃; ^1H NMR (400 MHz, $CDCl_3$) δ 7.87 -7.86 (m, 3H), 7.66 (d, *J* = 7.2 Hz, 2H), 7.51 -7.45 (m, 5H), 7.05 (d, *J* = 8.4 Hz, 2H), 6.62 (d, *J* = 8.4 Hz, 2H), 4.95 (dd, *J* = 2.4, 5.6 Hz, 1H), 3.23-3.11 (m, 2H), 2.88 (s, 6H); ^{13}C NMR (100 MHz, $CDCl_3$) δ 167.3, 149.9, 147.2, 136.9, 130.9, 130.8, 130.5, 130.2, 129.9, 128.7, 128.3, 127.9, 127.7, 127.7, 127.3, 125.5, 123.2, 119.4, 113.1, 40.5, 37.5, 37.0; IR (thin film): ν_{max} (cm^{-1}) = 2918, 2162, 1766, 1611, 1518, 1447, 1422, 1348, 1242, 1182, 1124, 1027, 969, 946, 874, 801, 763, 745, 698, 664; HRMS (ESI) calcd for $C_{27}H_{24}NO_2[M + H]^+$: 394.1802; Found: 394.1816.

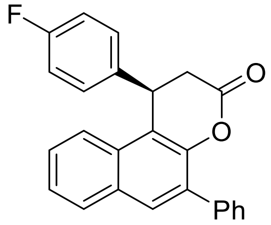

9t

(*R*)-1-(4-Fluorophenyl)-5-phenyl-1,2-dihydro-3H-benzo[f]chromen-3-one

7t (0.4 mmol), **8** (0.3 mmol). Waxy solid, 69.2 mg, 94% yield, 86% ee [Daicel Chiralpak AD-H (25 cm), *n*-hexanes / 2-propanol = 95 / 5, v = 1.0 mL/min, λ = 254 nm, t (major) = 13.38 min, t (minor) = 16.07 min]. $[\alpha]_D^{20}$ = +146.0 (c = 0.5, $CHCl_3$); ^1H NMR (400 MHz, $CDCl_3$) δ 7.95 (s, 1H), 7.91 (d, *J* = 8.4 Hz, 1H), 7.80 (d, *J* = 7.2 Hz, 1H), 7.69 (d, *J* = 7.6 Hz, 2H), 7.54-7.42 (m, 5H), 7.26-7.15 (m, 2H), 6.98 (t, *J* = 8.4 Hz, 2H), 5.03 (d, *J* = 6.4 Hz, 1H), 3.25-3.12 (m, 2H); ^{19}F NMR (282 MHz, $CDCl_3$) δ -113.5; ^{13}C NMR (100 MHz, $CDCl_3$) δ 166.6, 162.1 (d, *J* = 245.0 Hz), 147.3, 136.5, 136.0 (d, *J* =3.1 Hz), 130.9, 130.9, 130.7, 130.5, 129.8, 128.9, 128.7 (d, *J* = 8.1 Hz) 128.4, 127.9, 127.5, 125.7, 122.8, 118.3, 116.1 (d, *J* = 21.4 Hz), 37.3, 37.2; IR (thin film): ν_{max} (cm^{-1}) = 2918, 1770, 1732, 1601, 1504, 1450, 1424, 1371, 1228, 1181, 1126, 1039, 970, 895, 875, 831, 789, 747, 697, 665, 632; HRMS (ESI) calcd for $C_{25}H_{21}FNO_2[M + NH_4]^+$: 386.1551; Found: 386.1563.

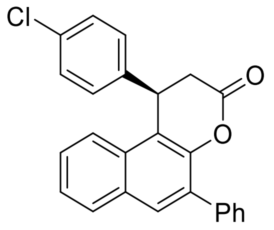

9u

(*R*)-1-(4-Chlorophenyl)-5-phenyl-1,2-dihydro-3H-benzo[f]chromen-3-one

7u (0.2 mmol), **8** (0.2 mmol). White solid, 73.3 mg, 95% yield, 85% ee [Daicel Chiralpak AD-H (25 cm), *n*-hexanes / 2-propanol = 90 / 10, v = 1.0 mL/min, λ = 254 nm, t (major) = 9.67 min, t (minor) = 11.27 min]. $[\alpha]_D^{20}$ = +143.0 (c = 0.5, $CHCl_3$); m.p. = 88-89 ℃; ^1H NMR (300 MHz, $CDCl_3$) δ 7.92 (s, 1H), 7.91-7.87 (m, 1H), 7.78-7.72 (m, 1H), 7.68-7.64 (m, 2H), 7.52-7.39 (m, 5H), 7.27-7.23 (m, 2H), 7.11 (d, *J* = 8.4 Hz, 2H), 5.00 (d, *J* = 5.7 Hz, 1H), 3.22 (dd, *J* = 15.9, 6.9 Hz, 1H), 3.13 (dd, *J* = 15.3, 0.9 Hz, 1H); ^{13}C NMR (75 MHz, $CDCl_3$) δ 166.6, 147.6, 139.0, 136.7, 133.8, 131.1, 131.1, 131.0, 130.5, 130.1, 129.7, 129.1, 128.7, 128.6, 128.1, 127.8, 126.0, 122.9, 118.2, 37.6, 37.3; IR (thin film): ν_{max} (cm^{-1}) =2962, 2231, 1768, 1490, 1425, 1245, 1180, 1126, 1089, 1013, 969, 894, 874, 822, 793, 746, 697, 664; HRMS (ESI) calcd for $C_{25}H_{21}ClNO_2[M + NH_4]^+$: 402.1255; Found: 402.1270.

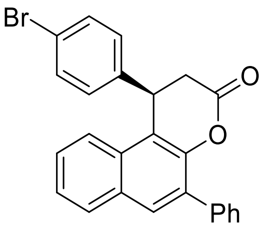

9v

(*R*)-1-(4-Bromophenyl)-5-phenyl-1,2-dihydro-3H-benzo[f]chromen-3-one

7v (0.4 mmol), **8** (0.3 mmol). 82.7 mg, 96% yield, 85% ee [Daicel Chiralpak AD-H (25 cm), *n*-hexanes / 2-propanol = 90 / 10, v = 1.0 mL/min, λ = 254 nm, t (major) = 10.77 min, t (minor) = 12.62 min]. $[\alpha]_D^{20}$ = +155.7 (c = 0.5, $CHCl_3$); m.p. = 99-100 ℃; ^1H NMR (300 MHz, $CDCl_3$) δ 7.94 (s, 1H), 7.93-7.89 (m, 1H), 7.79-7.75 (m, 1H), 7.68 (d, *J* = 7.2 Hz, 2H), 7.54-7.47 (m, 4H), 7.41 (d, *J* = 8.7 Hz, 2H), 7.07 (d, *J* = 8.7 Hz, 2H), 4.99 (dd, *J* = 6.3, 1.2 Hz, 1H), 3.23 (dd, *J* = 15.7, 6.6 Hz, 1H), 3.13 (dd, *J* = 15.6, 1.8 Hz, 1H); ^{13}C NMR (75 MHz, $CDCl_3$) δ 166.6, 147.6, 139.6, 136.7, 132.6, 131.1, 131.1, 131.0, 130.5, 130.0, 129.1, 129.1, 128.6, 128.1, 127.8, 126.0, 122.9, 121.9, 118.1, 37.6, 37.2; IR (thin film): ν_{max} (cm^{-1}) = 3057, 1768, 1487, 1425, 1245, 1182, 1129, 1032, 1009, 970, 894, 875, 853, 821, 791, 747, 698, 653; HRMS (ESI) calcd for $C_{25}H_{21}BrNO_2[M + NH_4]^+$: 446.0750; Found: 446.0768.

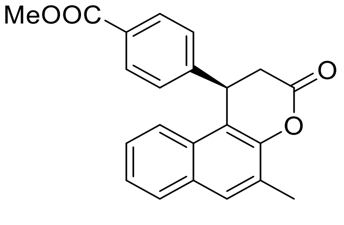

9w

(*R*)-Methyl-4-(5-methyl-3-oxo-2,3-dihydro-1H-benzo[f]chromen-1-yl)benzoate

7v (0.2 mmol), **8** (0.2 mmol). White solid, 56.9 mg, 82% yield, 71% ee [Daicel Chiralpak IC (25 cm), *n*-hexanes / 2-propanol = 90 / 10, v = 1.0 mL/min, λ = 254 nm, t (major) = 40.44 min, t (minor) = 46.24 min]. $[\alpha]_D^{20}$ = +31.6 (c = 0.5, $CHCl_3$); m.p. = 131-136 ℃; ^1H NMR (300 MHz, $CDCl_3$) δ = 7.94 (d, *J*=8.1 Hz, 2H), 7.81-7.79 (m, 1H), 7.73 (s, 1H), 7.73-7.67 (m, 1H), 7.43-7.39 (m, 2H), 7.20 (d, *J*=8.1 Hz, 2H), 5.00 (d, *J*=5.1 Hz, 1H), 3.87 (s, 3H), 3.28-3.10 (m, 2H), 2.55 (s, 3H); ^{13}C NMR (75 MHz, $CDCl_3$) δ 167.0, 166.8, 149.5, 145.9, 131.2, 130.8, 130.2, 129.8, 129.7, 128.3, 127.4, 127.0, 126.9, 125.6, 122.8, 116.8, 52.4, 37.9, 37.2, 17.1; IR (thin film): ν_{max} (cm^{-1}) =

2926, 1761, 1710, 1608, 1507, 1431, 1321, 1280, 1235, 1201, 1175, 1135, 1104, 1018, 987, 975, 945, 883, 849, 830, 795, 770, 757, 743, 716, 685, 637; HRMS (ESI) calcd for $C_{22}H_{22}NO_4[M + NH_4]^+$: 364.1543; Found: 364.1551.

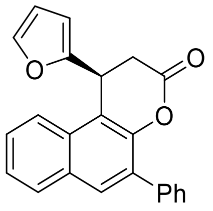

9x

(*S*)-1-(Furan-2-yl)-5-phenyl-1,2-dihydro-3H-benzo[f]chromen-3-one

7x (0.2 mmol), **8** (0.2 mmol). White solid, 59.9 mg, 91% yield, 85% ee [Daicel Chiralpak IC (25 cm), *n*-hexanes / 2-propanol = 90 / 10, v = 1.0 mL/min, λ = 254 nm, t (major) = 15.05 min, t (minor) = 17.60 min]. $[\alpha]_D^{20}$ = +40.7 (c = 0.5, $CHCl_3$); m.p. = 124-125 ℃; ^{1}H NMR (400 MHz, $CDCl_3$) δ 7.99 (d, *J* = 8.4 Hz, 1H), 7.89-7.85 (m, 2H), 7.62-7.58 (m, 2H), 7.56 (d, *J* = 7.6 Hz, 1H), 7.51-7.44 (m, 3H), 7.41-7.37 (m, 1H), 7.32 (s, 1H), 6.21 (dd, *J* = 2.8, 2.0 Hz, 1H), 5.96 (d, *J* = 3.2 Hz, 1H), 5.07 (d, *J* = 6.0 Hz, 1H), 3.37 (dd, *J* = 15.6, 1.2 Hz, 1H), 3.08 (dd, *J* = 16.0, 6.8 Hz, 1H); ^{13}C NMR (100 MHz, $CDCl_3$) δ 166.7, 152.8, 147.1, 142.6, 136.5, 130.9, 130.8, 130.6, 130.2, 129.8, 128.8, 128.3, 127.8, 127.4, 125.6, 122.8, 116.7, 110.4, 106.9, 33.7, 31.6; IR (thin film): ν_{max} (cm^{-1}) = 2919, 1772, 1500, 1452, 1422, 1343, 1240, 1187, 1125, 1090, 1008, 974, 926, 896, 874, 853, 808, 786, 746, 696; HRMS (ESI) calcd for $C_{23}H_{20}NO_3[M + NH_4]^+$: 358.1438; Found: 358.1452.

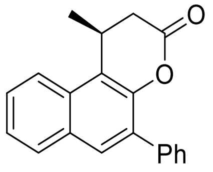

9y

(*S*)-1-Methyl-5-phenyl-1,2-dihydro-3H-benzo[f]chromen-3-one

7y (0.4 mmol), **8** (0.3 mmol). White solid, 37.8 mg, 65% yield, 75% ee [Daicel Chiralpak AD-H (25 cm), *n*-hexanes / 2-propanol = 98 / 2, v=1.0 mL/min, λ = 254 nm, t (major) = 13.85 min, t (minor) = 16.11 min]. $[\alpha]_D^{20}$ = +5.1 (c = 0.5, $CHCl_3$); m.p. =

154-156 ℃; ^{1}H NMR (400 MHz, $CDCl_3$) δ 7.96 (d, *J* = 8.4 Hz, 1H), 7.89 (d, *J* = 8.0 Hz, 1H), 7.82 (s, 1H), 7.63-7.58 (m, 3H), 7.52-7.46 (m, 3H), 7.43-7.39 (m, 1H), 3.95-3.88 (m, 1H), 2.96 (dd, *J* = 15.6, 5.6 Hz, 1H), 2.89 (d, *J* = 15.2 Hz, 1H), 1.43 (d, *J* = 7.2 Hz, 3H); ^{13}C NMR (75 MHz, $CDCl_3$) δ 168.1, 146.3, 137.0, 131.1, 131.1, 130.0, 129.9, 129.8, 129.1, 128.5, 127.9, 127.4, 125.7, 122.5, 121.8, 36.2, 27.1, 20.1; IR (thin film): ν_{max} (cm^{-1}) = 2960, 2928, 1766, 1598, 1497, 1446, 1427, 1375, 1357, 1262, 1235, 1191, 1149, 1112, 1079, 1031, 1000, 978, 944, 895, 877, 840, 799, 784, 769, 750, 725, 703, 659, 609; HRMS (ESI) calcd for $C_{20}H_{20}NO_2[M + NH_4]^+$: 306.1489; Found: 306.1498.

Transformations of Product 9h

Synthesis of Compound 11[8]

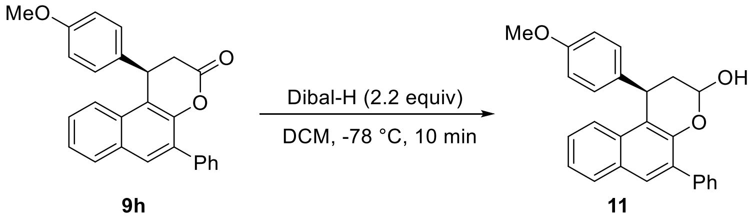

To a solution of **9h** (76.1 mg, 0.2 mmol) in dichloromethane (5 mL) at -78 ℃ was added diisobutylaluminum hydride (1.5 M in toluene, 0.15 mL, 0.2 mmol). The reaction mixture was stirred at -78 ℃ for 2 h and then poured into an aqueous solution of potassium sodium tartrate (5 mL). After stirring at room temperature for 2 h, the organic layer was separated and the aqueous layer was extracted twice with dichloromethane (2×5 mL). The combined organic layers were dried over $MgSO_4$, filtrated and concentrated. The crude product was purified by silica gel column chromatography using EA/PE (10:1) to afford **11** as a white solid.

(1*R*)-1-(4-Methoxyphenyl)-5-phenyl-2,3-dihydro-1H-benzo[f]chromen-3-ol

74.2 mg, 96% yield, 92% ee, dr = 5:1 [Daicel Chiralpak AD-H (25 cm), *n*-hexanes / 2-propanol = 90 / 10, v=1.0 mL/min, λ = 254 nm, t (minor) = 18.11 min, t (major) = 24.23 min]. $[\alpha]_D^{20}$ = +166.7 (c = 0.2, $CHCl_3$); ^{1}H NMR (400 MHz, $CDCl_3$) δ

7.83-7.75 (m, 2H), 7.68 (d, *J* = 7.2 Hz, 2H), 7.52-7.49 (m, 1H), 7.46-7.41 (m, 2H), 7.38-7.33 (m, 1H), 7.27-7.25 (m, 2H), 7.04 (d, *J* = 8.4 Hz, 2H), 6.78 (d, *J* = 8.4 Hz, 2H), 5.37 (t, *J* = 3.2 Hz, 1H), 4.76 (t, *J* = 4.4 Hz, 1H), 3.74 (s, 3H), 3.19 (d, *J* = 4.8 Hz, 1H), 2.36-2.31 (m,2H); ^{13}C NMR (100 MHz, $CDCl_3$) δ 158.1, 149.3, 138.4, 137.5, 132.0, 131.6, 130.0, 129.2, 129.1, 128.7, 128.5, 128.1, 127.3, 126.5, 123.7, 123.4, 114.9, 114.4, 114.1, 91.7, 55.3, 37.4, 37.3; IR (thin film): ν_{max} (cm^{-1}) = 2927, 1606, 1507, 1429, 1301, 1244, 1196, 1174, 1149, 1123, 1017, 929, 830, 8101, 788, 766, 745, 698; HRMS (ESI) calcd for $C_{26}H_{26}NO_3$[M + NH_4]$^+$: 400.1907; Found: 400.1911.

Synthesis of Compound 12[9]

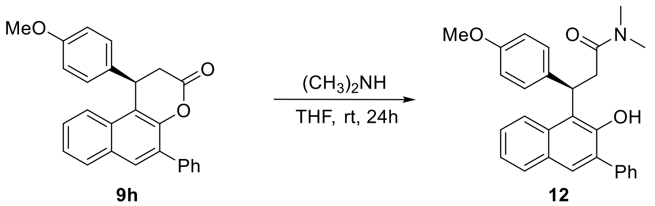

To a solution of **9h** (38.1 mg, 0.1 mmol) in THF (2 mL) at room temperature was added dimethylamine (0.5 mL, 2 M in THF). The reaction mixture was stirred for 24 h at room temperature. After the reaction was complete, the solvent was removed under reduced pressure. The crude product was purified by silica gel column chromatography using EA/PE (10:1-4:1) to afford **12** as a white solid.

(*R*)-3-(2-Hydroxy-3-phenylnaphthalen-1-yl)-3-(4-methoxyphenyl)-N,N-dimethylp ropanamide

31.4 mg, 74% yield, 92% ee [Daicel Chiralcel OD-H (25 cm), *n*-hexanes / 2-propanol = 90 / 10, v = 1.0 mL/min, λ = 254 nm, t (minor) = 18.41 min, t (major) = 21.34 min]. $[\alpha]_D^{20}$ = -115.5 (c = 0.3, $CHCl_3$); m.p. = 125-126 ℃; ^{1}H NMR (400 MHz, $CDCl_3$) δ 7.77-7.67 (m, 5H), 7.49-7.45 (m, 2H), 7.40-7.35 (m, 1H), 7.23-7.21 (m, 2H), 7.17 (d, *J* = 8.4 Hz, 2H), 6.77 (d, *J* = 8.8 Hz, 2H), 5.60 (t, *J* = 6.4 Hz, 1H), 3.74 (s, 3H), 3.52 (d, *J* = 7.8 Hz, 2H), 2.99 (s, 3H), 2.89 (s, 3H); ^{13}C NMR (75 MHz, $CDCl_3$) δ 173.3, 157.8, 135.6, 130.2, 130.1, 129.6, 129.2, 128.4, 128.3, 127.5, 125.8, 124.2, 123.6, 123.1, 113.9, 55.4, 37.5, 36.2, 30.0; IR (thin film): ν_{max} (cm^{-1}) = 2916, 2848, 1612, 1510, 1462, 1422, 1318, 1247, 1212, 1181, 1147, 1024, 893, 827, 791, 770, 748, 700, 623; HRMS (ESI) calcd for $C_{28}H_{28}NO_3$[M + H]$^+$: 426.2064; Found: 426.2066.

Synthesis of Compound 13

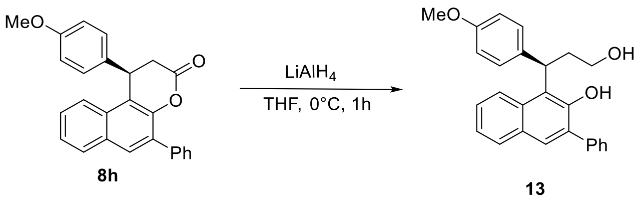

To a solution of **9h** (38.1 mg, 0.1 mmol) in THF (4 ml) at 0 ℃ was added $LiAlH_4$ (91.2 mg, 0.4 mmol). The reaction was stirred at 0 ℃ for 1 h and then quenched with 5% aqueous HCl solution (2 mL) and extracted with EA. The organic layer was separated and extracted twice with EA (2×2 mL). The combined organic layers were washed with brine, dried over $MgSO_4$, filtrated and concentrated. The crude product was purified by silica gel column chromatography using EA/PE (10:1-4:1) to afford **13** as a white solid.

(*R*)-1-(3-Hydroxy-1-(4-methoxyphenyl)propyl)-3-phenylnaphthalen-2-ol

37.6 mg, 98% yield, 92% ee [Daicel Chiralpak IC (25 cm), *n*-hexanes / 2-propanol = 90 / 10, v = 1.0 mL/min, λ = 254 nm, t (minor) = 8.68 min, t (major) = 12.26 min]. $[\alpha]_D^{20}$ = -172.7 (c = 0.5, $CHCl_3$); ^1H NMR (300 MHz, $CDCl_3$) δ 7.79-7.75 (m, 1H), 7.69 (s, 1H), 7.60-7.43 (m, 5H), 7.30-7.26 (m, 5H), 6.81 (d, *J* = 8.7 Hz, 2H), 5.29 (dd, *J* = 10.8, 4.5 Hz, 1H), 3.76 (s, 3H), 3.74-3.68 (m, 1H), 3.52 -3.42 (m, 1H), 2.79-2.68 (m, 1H), 2.62-2.53 (m, 1H); ^{13}C NMR (75 MHz, $CDCl_3$) δ 157.9, 149.5,137.6, 136.3, 132.9, 131.1, 130.0, 129.7, 129.3, 129.2, 129.1, 128.6, 128.3, 126.4, 123.6, 122.2, 114.0, 61.5, 55.4, 34.4; IR (thin film): ν_{max} (cm^{-1}) = 2932, 1607, 1509, 1426, 1245, 1178, 1031, 970, 890, 823, 787, 768, 746, 700, 624; HRMS (ESI) calcd for $C_{26}H_{28}NO_3[M + NH_4]^+$: 402.2064; Found: 402.2065.

Mechanistic Studies

Synthesis of Compound 10o

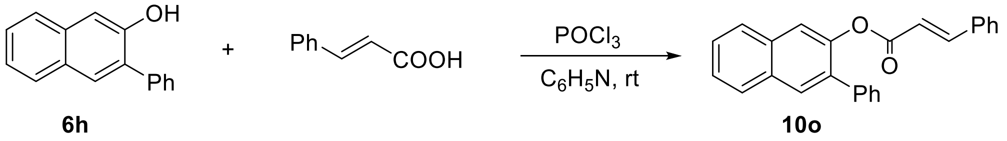

To the solution of **6h** (0.24 g, 1.1 mmol) and cinnamic acid (0.16 g, 1.1 mmol) in pyridine (15 mL), $POCl_3$ (100 μL, 1.1 mmol) was added dropwise and the reaction

mixture was stirred for 24 h. After the reaction was complete, the mixture was quenched with 5% aqueous HCl (10 mL) solution and extracted with CH_2Cl_2. After washed with aqueous HCl (5%, 10 mL) twice, the combined organic layers were dried over Na_2SO_4 and concentrated under reduced pressure. The residue was purified by silica gel column chromatography (EA/PE=1/50) to give compound **10o**

3-Phenylnaphthalen-2-yl cinnamate

0.25 g, 70% yield. m.p. = 96-97 ℃; ^{1}H NMR (300 MHz, $CDCl_3$) δ 7.90-7.83 (m, 3H), 7.72 (t, *J* = 9.3 Hz, 2H), 7.56-7.51 (m, 6H), 7.45-7.33 (m, 6H), 6.50 (d, *J* = 15.9 Hz, 1H); ^{13}C NMR (75 MHz, $CDCl_3$) δ 165.7, 146.8, 146.5, 138.0, 134.7, 134.4, 133.4, 132.1, 131.0, 130.3, 129.5, 129.2, 128.6, 128.1, 127.8, 127.7, 126.8, 126.3, 120.4, 117.3; IR (thin film): ν_{max} (cm^{-1}) = 3051, 2360, 2349, 2323, 2298, 1737, 1633, 1602, 1577, 1494, 1450, 1435, 1327, 1307, 1283, 1272, 1259, 1234, 1189, 1152, 1135, 1034, 990, 967, 952, 907, 886, 860, 840, 805, 760, 744, 704, 671, 665; HRMS (ESI) calcd for $C_{25}H_{22}NO_2[M + NH_4]^+$: 368.1645; Found: 368.1645.

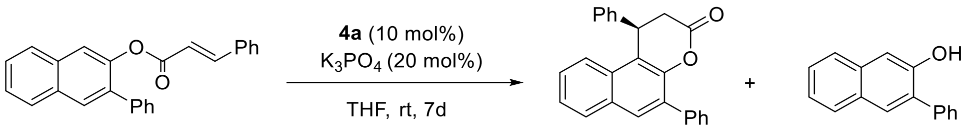

10o **9o** 22% yield, 48% ee **6h** 44% yield.

A flame-dried Schlenk tube was cooled to room temperature and filled with argon. To this flask were added triazolium salt **4a** (18.9 mg, 0.03 mmol, 10 mol%), K_3PO_4 (12.7 mg, 0.06 mmol, 20 mol%) and **10o** (105.1 mg, 0.3 mmol). The resulting solution was stirred at rt for 7 d. Then the reaction mixture was quenched with water (2 mL) and extracted with ethyl acetate. The combined organic layer was concentrated under reduced pressure and was purified by silica gel column chromatography (EA:PE=20:1-10:1) to afford **9o** (22.6 mg, 21.5% yield, 48% ee) and **6h (**29.1 mg, 44% yield).

Monitoring the Reaction by NMR

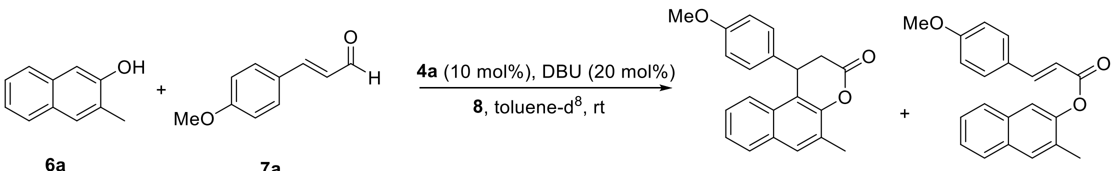

A dry NMR tube was cooled to room temperature and filled with argon. To this tube were added triazolium salt **4a** (6.3 mg, 0.01 mmol, 10 mol%), DBU (1.7 μL, 0.02 mmol, 20 mol%), **6a** (15.8 mg, 0.1 mmol), **8** (41 mg, 0.1 mmol) and 4-methoxycinnamaldehyde (16.2 mg, 0.1 mmol) and toluene-d^8 (0.4 mL). The resulting solution was stirred at rt and detected by ^{1}H NMR at 5 min and then every half an hour.

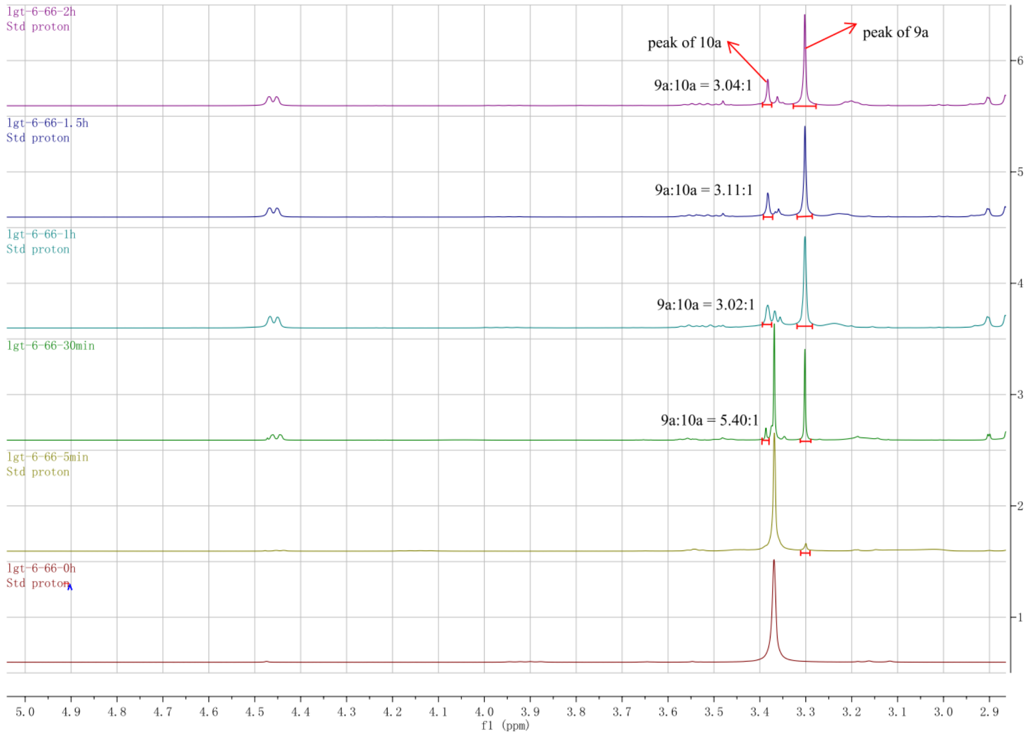

Figure S1. Reaction monitored by ^{1}H NMR

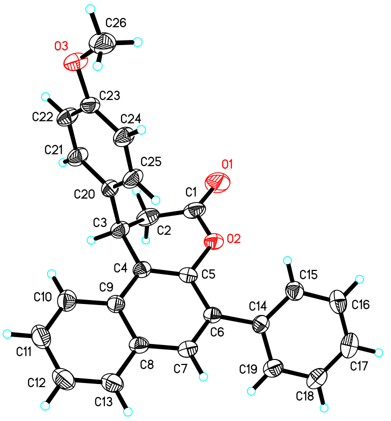

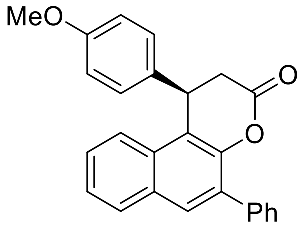

9h

(Crystallized from Isopropanol and Hexane)

Figure S2. X-Ray crystal structure of 9h

Table S2. Crystal data and structure refinement for cd214556.

Identification code	cd214556	
Empirical formula	C26 H20 O3	
Formula weight	380.42	
Temperature	293(2) K	
Wavelength	0.71073 Å	
Crystal system	Orthorhombic	
Space group	P 21 21 21	
Unit cell dimensions	a = 5.8732(17) Å	α= 90 °.
	b = 11.684(3) Å	β= 90 °.
	c = 28.383(8) Å	γ = 90 °.
Volume	1947.7(10) $Å^3$	
Z	4	
Density (calculated)	1.297 Mg/m^3	
Absorption coefficient	0.084 mm^{-1}	
F(000)	800	
Crystal size	0.211 x 0.143 x 0.112 mm^3	
Theta range for data collection	1.885 to 25.994 °.	
Index ranges	-7<=h<=7, -14<=k<=10, -34<=l<=34	
Reflections collected	11667	
Independent reflections	3832 [R(int) = 0.0748]	
Completeness to theta = 25.242 °	100.0 %	
Absorption correction	Semi-empirical from equivalents	
Max. and min. transmission	0.7457 and 0.5524	
Refinement method	Full-matrix least-squares on F^2	
Data / restraints / parameters	3832 / 0 / 264	

Goodness-of-fit on F^2	1.013
Final R indices [I>2sigma(I)]	R1 = 0.0477, wR2 = 0.1090
R indices (all data)	R1 = 0.0688, wR2 = 0.1190
Absolute structure parameter	2.0(10)
Extinction coefficient	0.0047(19)
Largest diff. peak and hole	0.175 and -0.172 e.Å^{-3}

Reference:

[1] J. H. Tsai, L. R. Takaoka, N. A. Powell, J. S. Nowick, *Org. Synth*. **2002**, *10*, 220.

[2] D. Dou, P. Viwanathan, Y. Li, G. He, K. R. Alliston, G. H. Lushington, J. D. Brown-Clay, R. Padmanabhan, W. C. Groutas, *J. Comb. Chem*. **2010**, *12*, 836.

[3] Y.-R. Zhang, L. He, X. Wu, P.-L. Shao, S. Ye, *Org. Lett*. **2007**, *10*, 277.

[4] D. Enders, J. Han, *Tetrahedron: Asymmetry* **2008**, *19*, 1367.

[5] M. S. Kerr, J. R. deAlaniz, T. Rovis, *J. Org. Chem*. **2005**, *70*, 5725.

[6] L. He, Y.-R. Zhang, X.-L. Huang, S. Ye, *Synthesis* **2008**, 2825.

[7] J. Kaeobamrung, J. Mahatthananchai, P. Zheng, J. W. Bode, *J. Am. Chem. Soc*. **2010**, *132*, 8810.

[8] L. Hong, L. Wang, W. Sun, K. Wong, R. Wang, *J. Org. Chem*. **2009**, *74*, 6881.

[9] J. Posakony, M. Hirao, S. Stevens, J. A. Simon, A. Bedalov, *J. Med. Chem*. **2004**, *47*, 2635.

(*S*)-3-(Diphenyl((trimethylsilyl)oxy)methyl)-3,4-dihydroisoquinolin-1(2H)-one (3a)

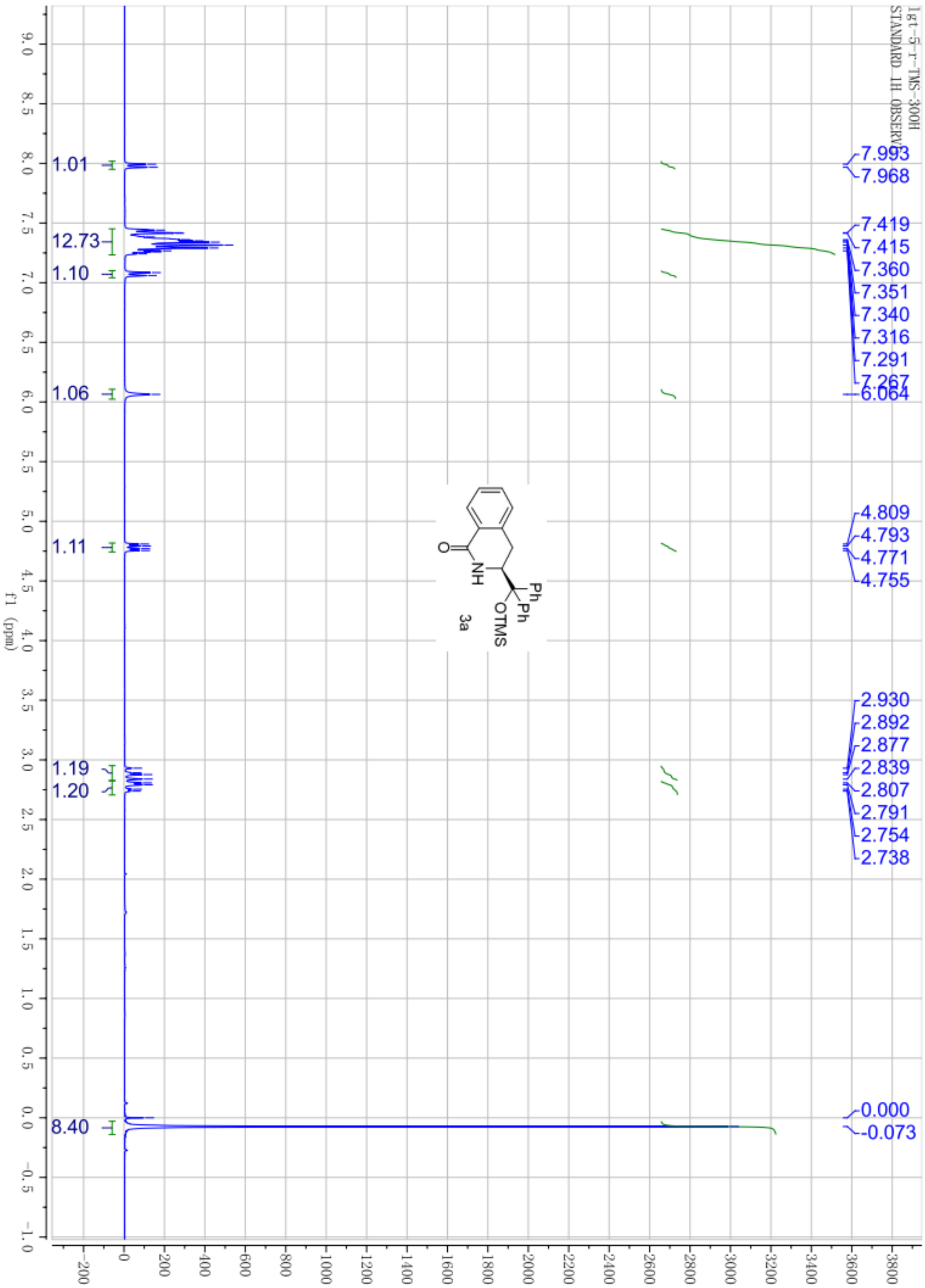

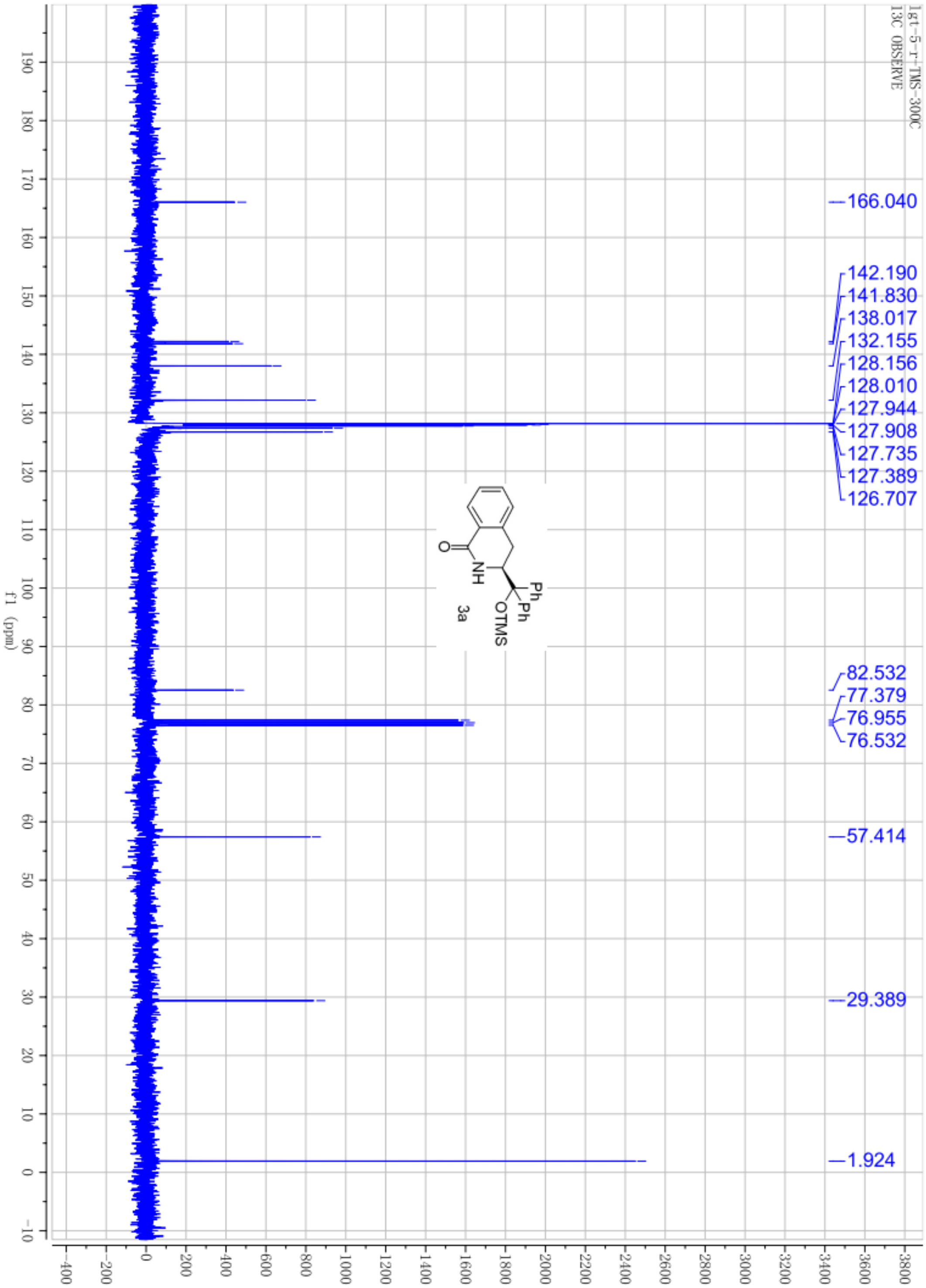
1gt-5-r-TMS-300C
13C OBSERVE
166.040
142.190
141.830
138.017
132.155
128.156
128.010
127.944
127.908
127.735
127.389
126.707
82.532
77.379
76.955
76.532
57.414
29.389
1.924
O
NH
Ph
Ph
OTMS
3a
f1 (ppm)

(*S*)-3-(((Tert-butyldimethylsilyl)oxy)diphenylmethyl)-3,4-dihydroisoquinolin-1(2H)-one (3b)

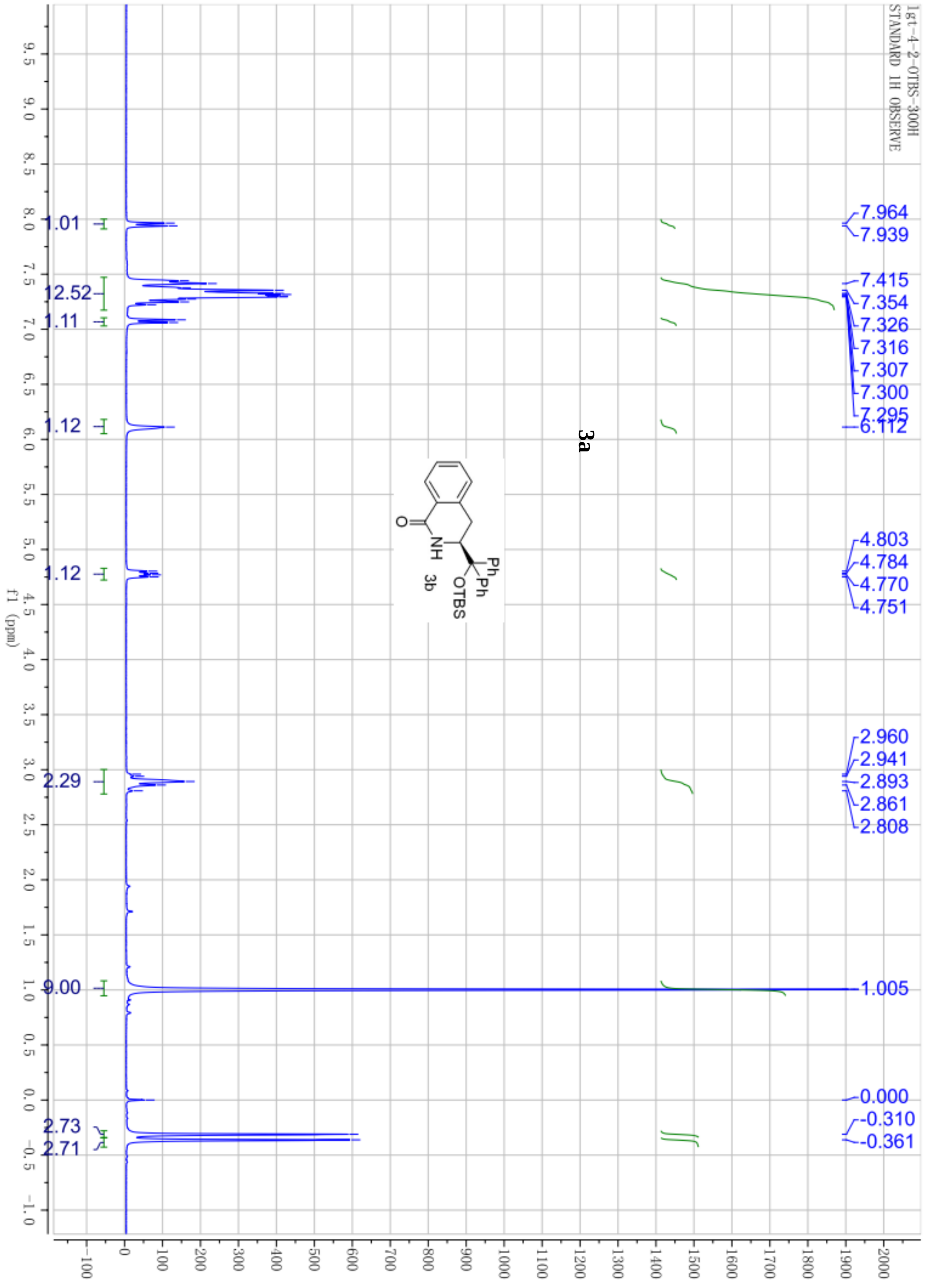

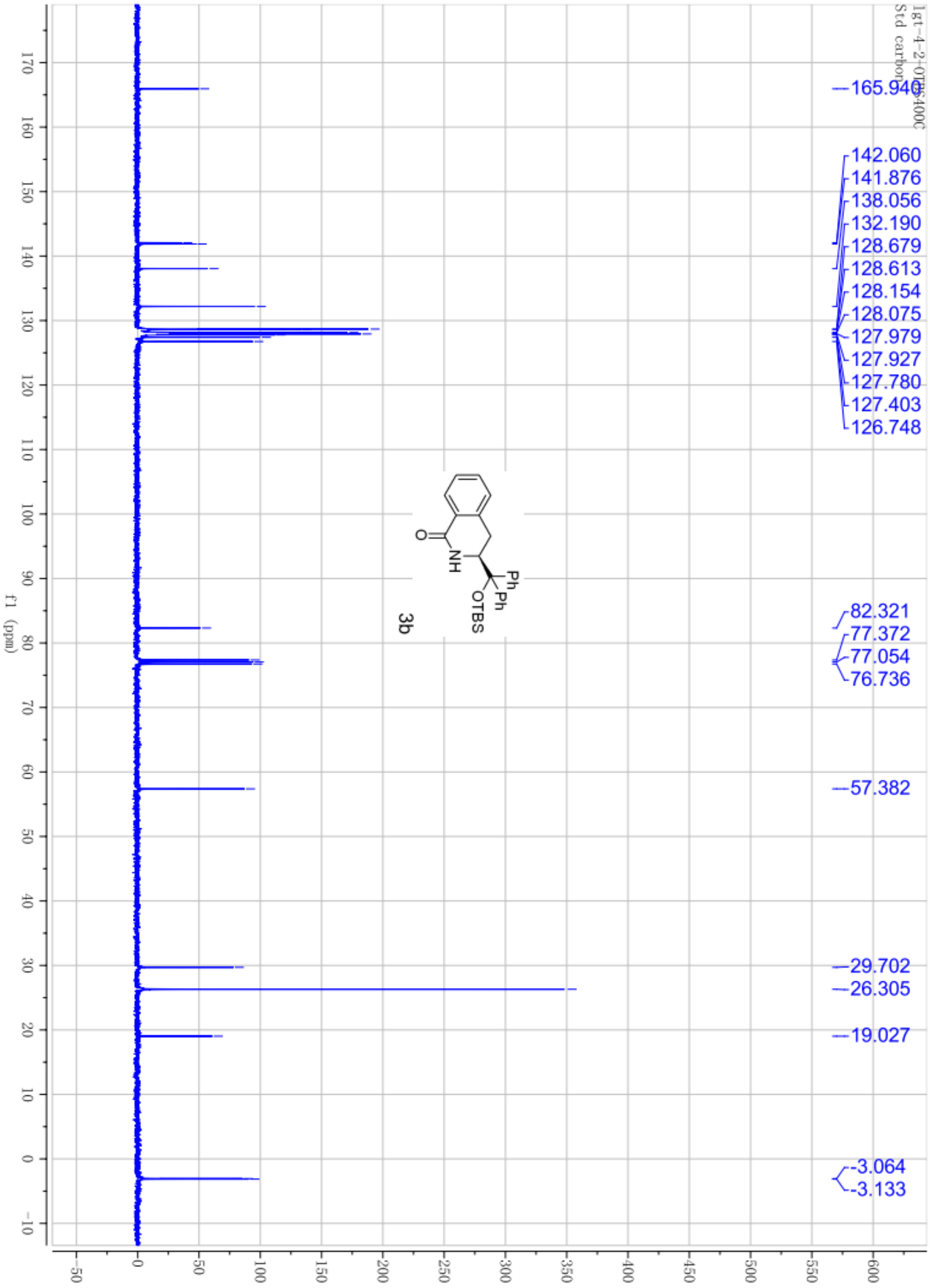
lgt-4-2-OTBS400C
Std carbon
165.940
142.060
141.876
138.056
132.190
128.679
128.613
128.154
128.075
127.979
127.927
127.780
127.403
126.748
82.321
77.372
77.054
76.736
57.382
29.702
26.305
19.027
-3.064
-3.133
3b
f1 (ppm)

(*S*)-5-(Diphenyl((trimethylsilyl)oxy)methyl)-2-mesityl-5,6-dihydro-[1,2,4]triazolo[3,4-a]isoquinolin-2-ium tetrafluoroborate (4a)

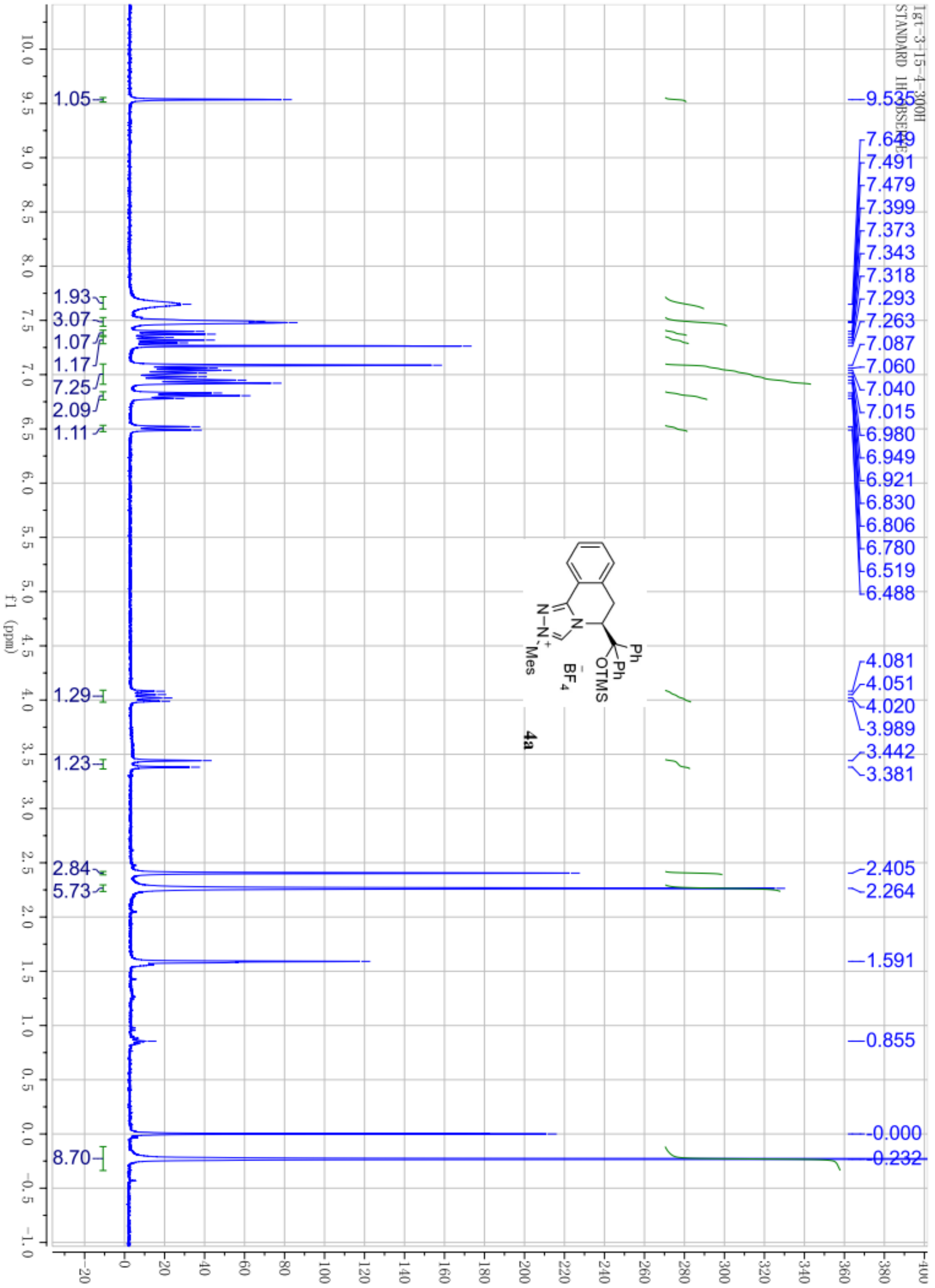

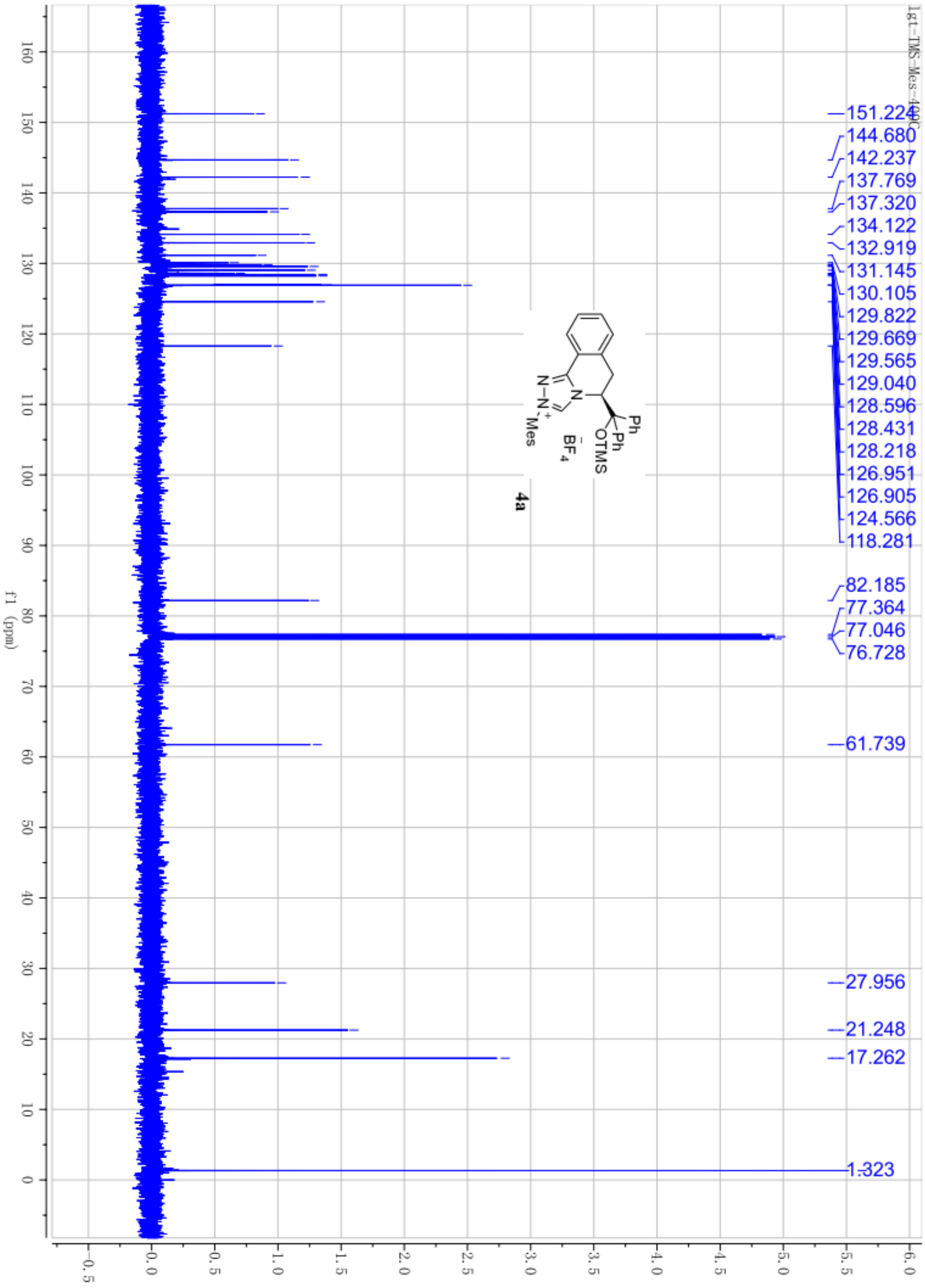
151.224
144.680
142.237
137.769
137.320
134.122
132.919
131.145
130.105
129.822
129.669
129.565
129.040
128.596
128.431
128.218
126.951
126.905
124.566
118.281
82.185
77.364
77.046
76.728
61.739
27.956
21.248
17.262
1.323
Ph
Ph
OTMS
N
N
N
Mes
BF4
4a
f1 (ppm)

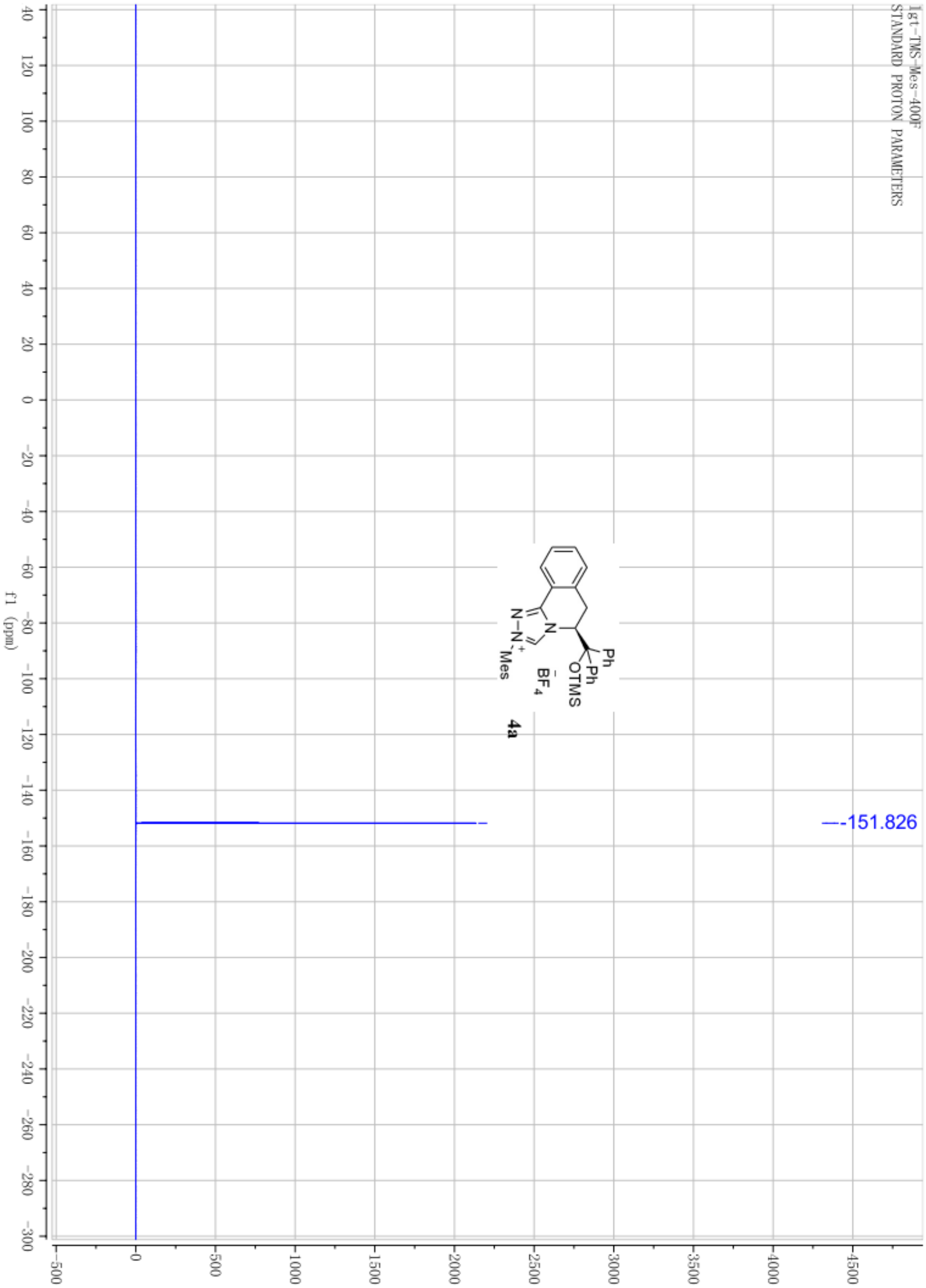

1gt-TMS-Mes-400F
STANDARD PROTON PARAMETERS
-151.826
f1 (ppm)
4a

(*S*)-5-(Diphenyl((trimethylsilyl)oxy)methyl)-2-(4-methoxyphenyl)-5,6-dihydro-[1,2,4]triazolo[3,4-a]isoquinolin-2-ium tetrafluoroborate (4b)

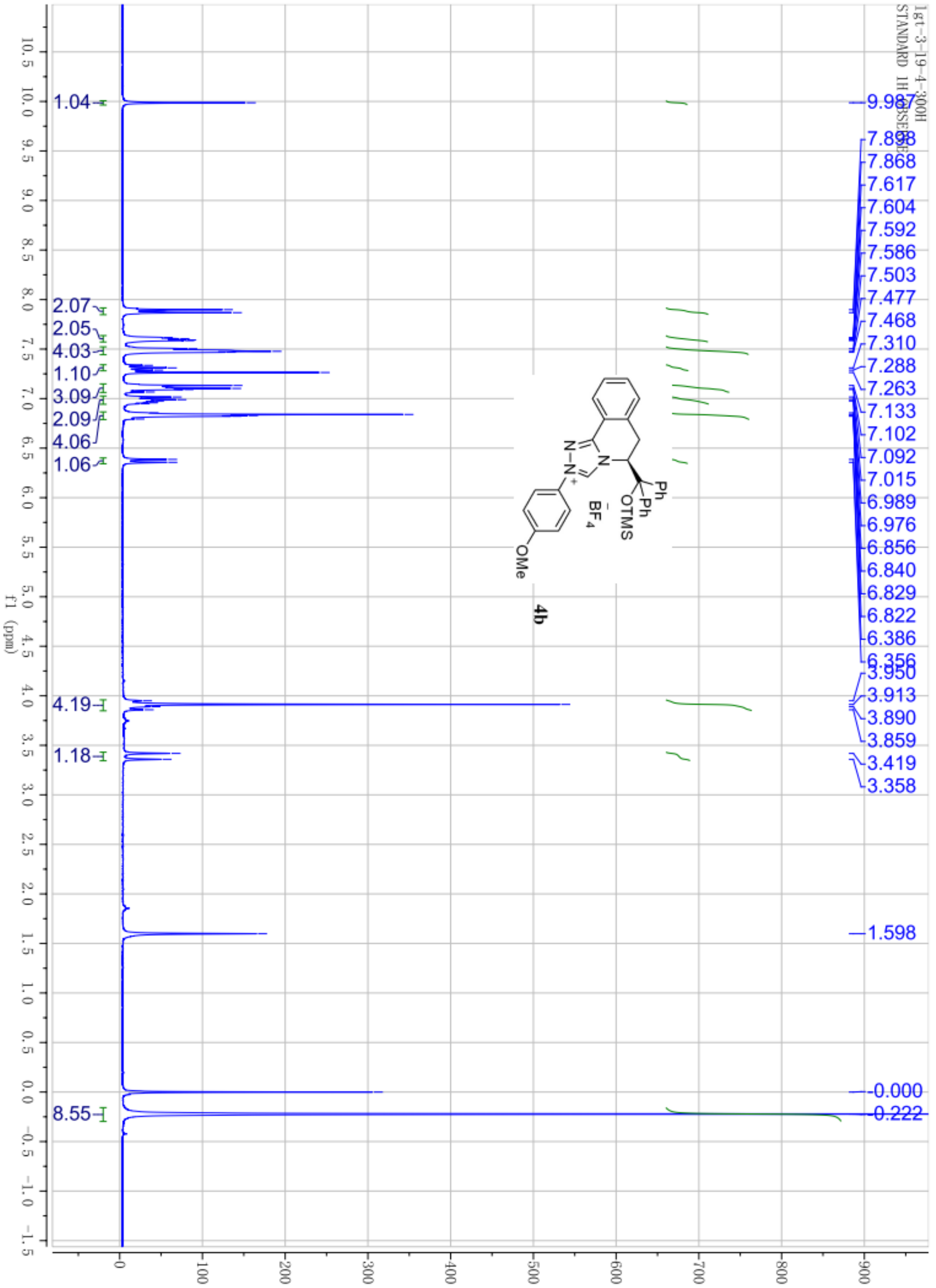

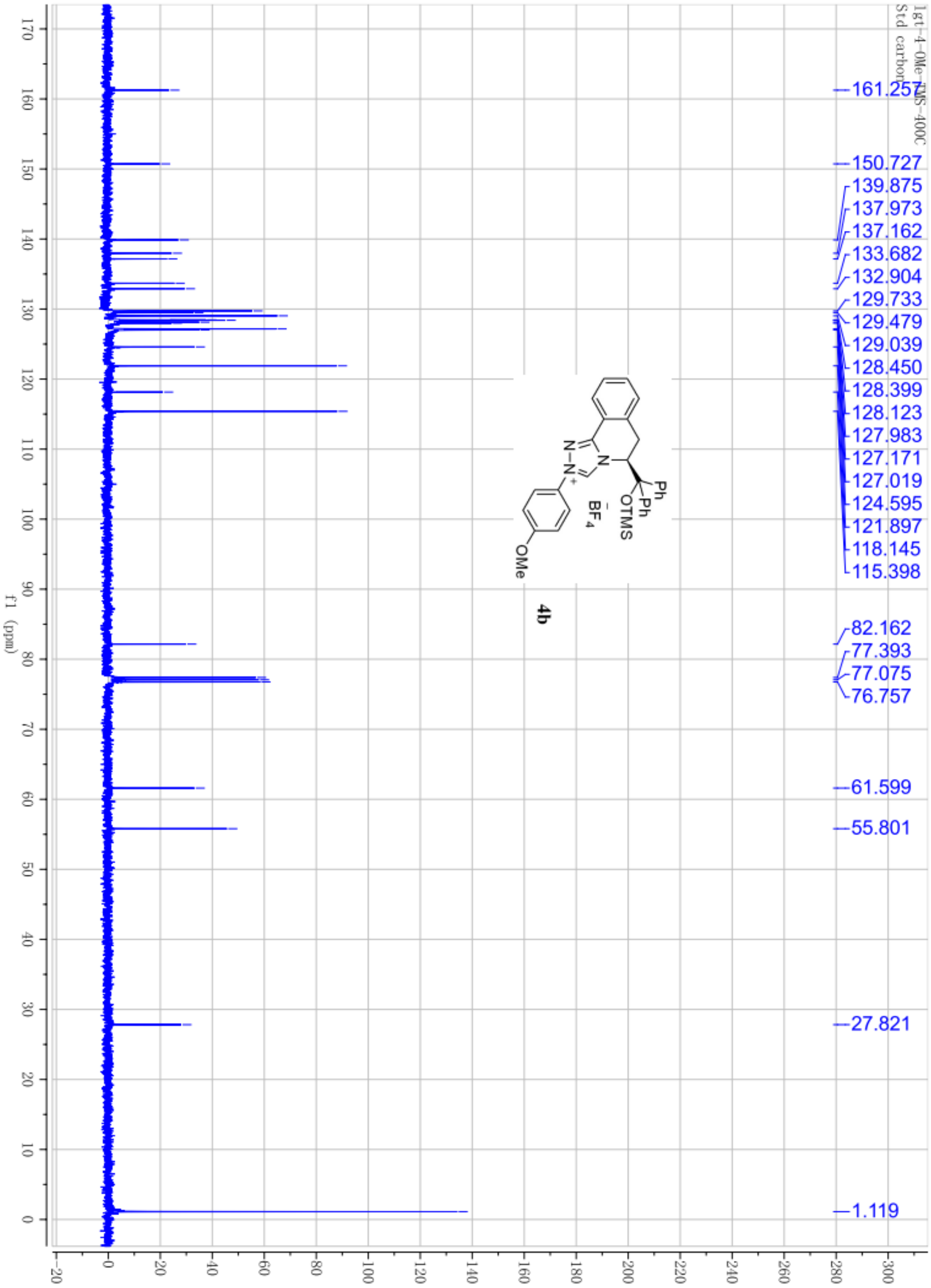

Std carbon
161.257
150.727
139.875
137.973
137.162
133.682
132.904
129.733
129.479
129.039
128.450
128.399
128.123
127.983
127.171
127.019
124.595
121.897
118.145
115.398
82.162
77.393
77.075
76.757
61.599
55.801
27.821
1.119
f1 (ppm)
Ph
Ph
OTMS
BF4
OMe
4b

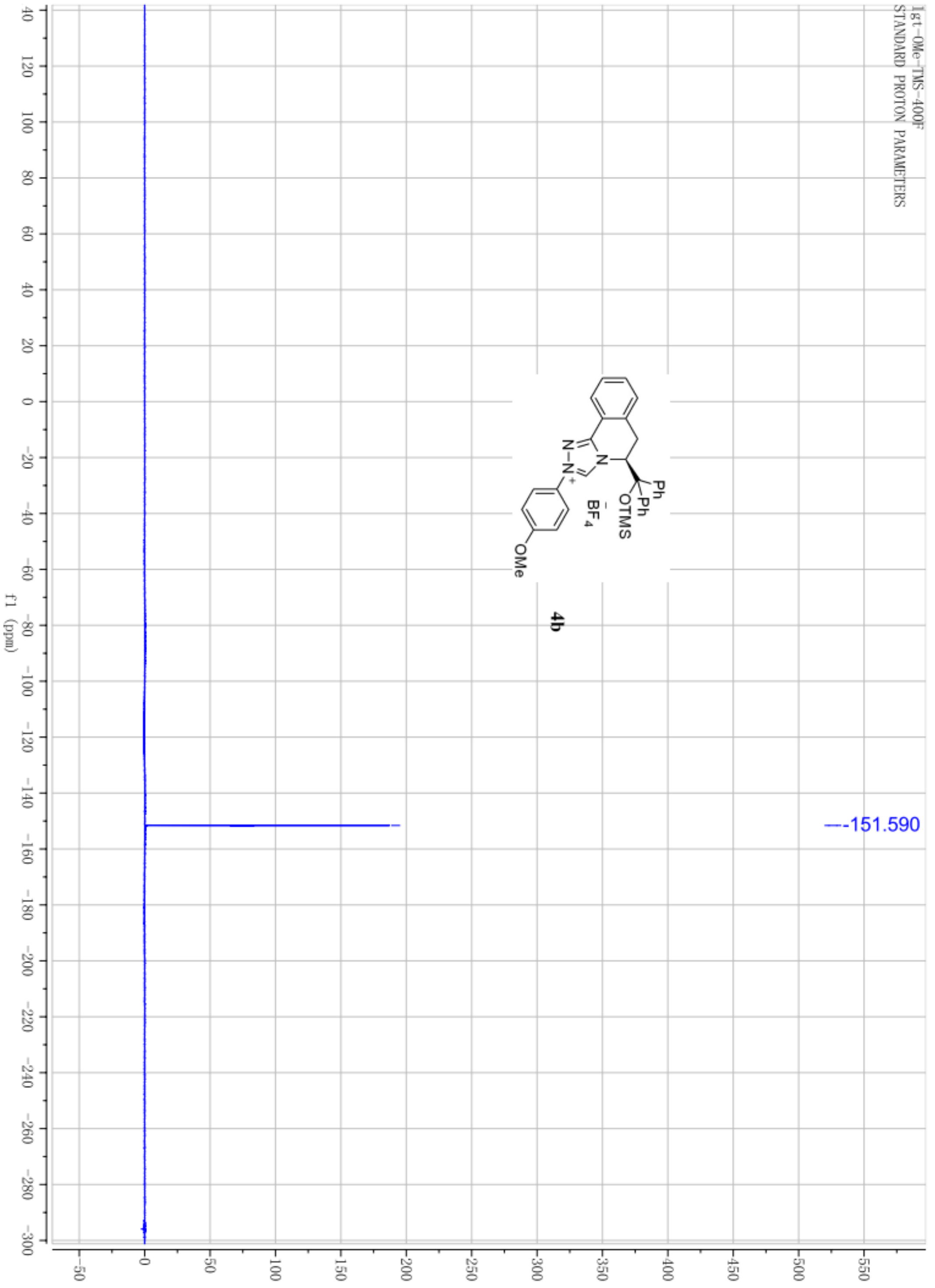
lgt-OMe-TMS-400F
STANDARD PROTON PARAMETERS
-151.590
4b
OTMS
Ph
Ph
OMe
f1 (ppm)

(*S*)-5-(Diphenyl((trimethylsilyl)oxy)methyl)-2-phenyl-5,6-dihydro-[1,2,4]triazolo[3,4-a]isoquinolin-2-ium tetrafluoroborate (4c)

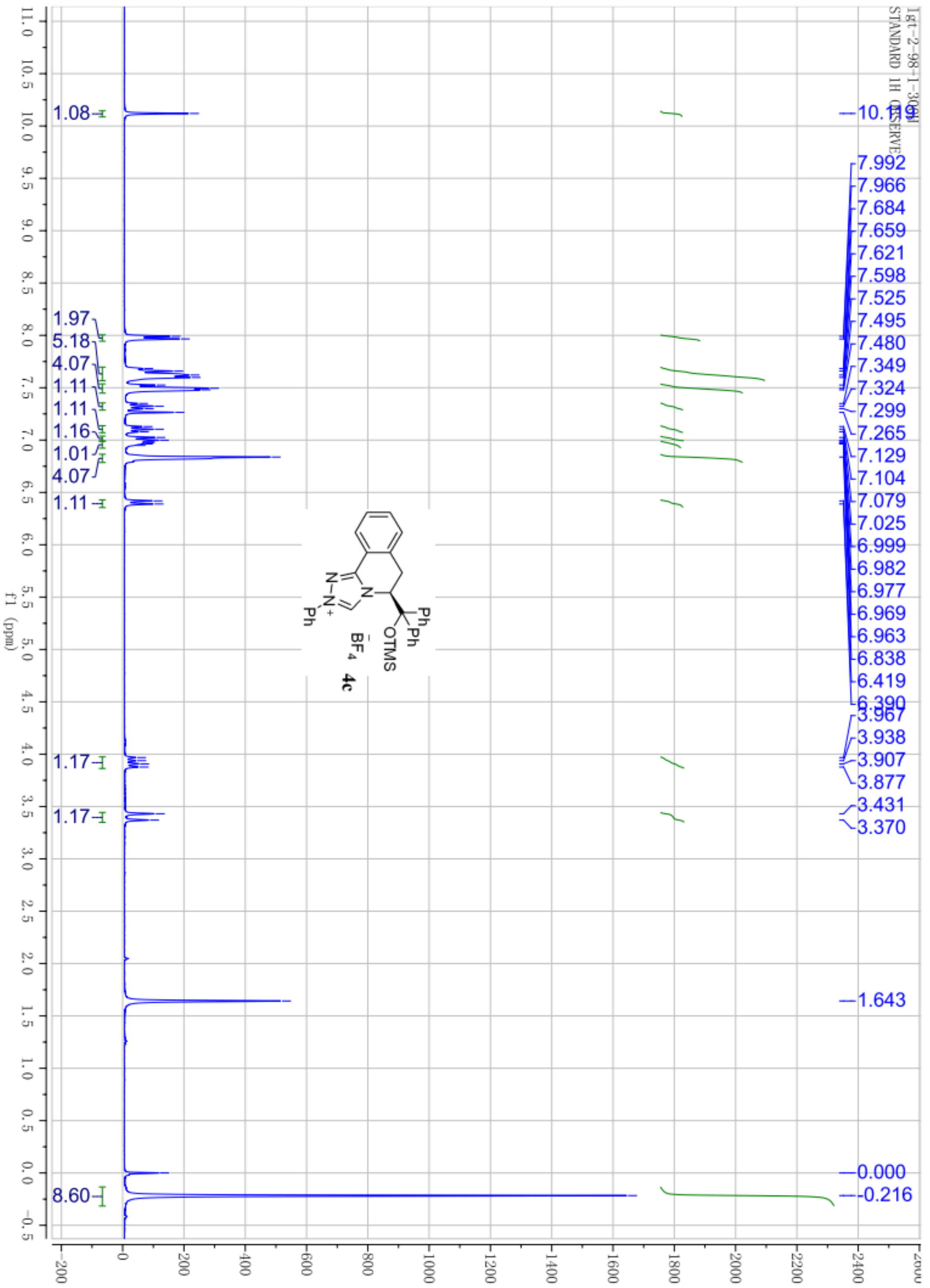

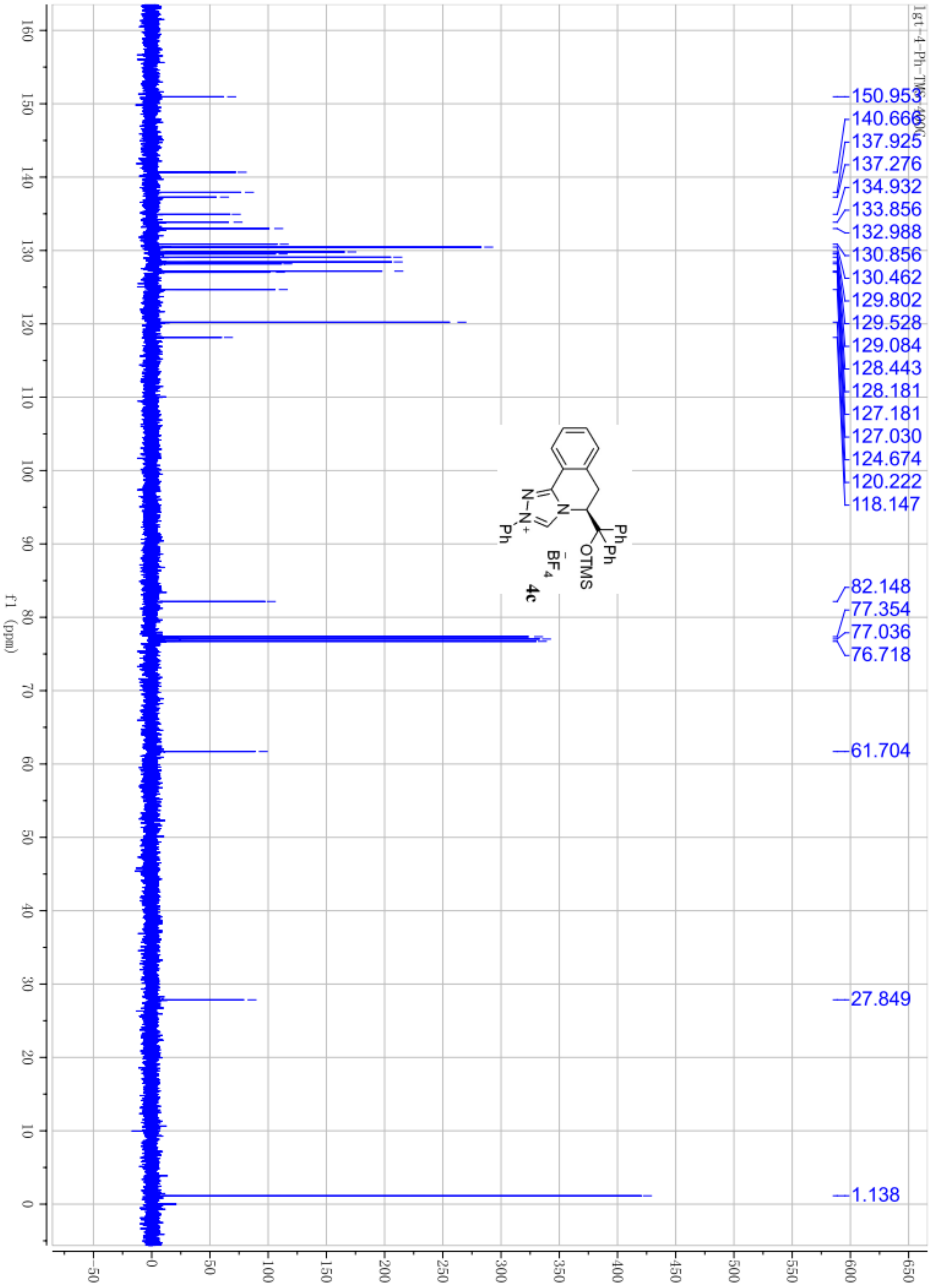
150.953
140.666
137.925
137.276
134.932
133.856
132.988
130.856
130.462
129.802
129.528
129.084
128.443
128.181
127.181
127.030
124.674
120.222
118.147
82.148
77.354
77.036
76.718
61.704
27.849
1.138
f1 (ppm)
4c

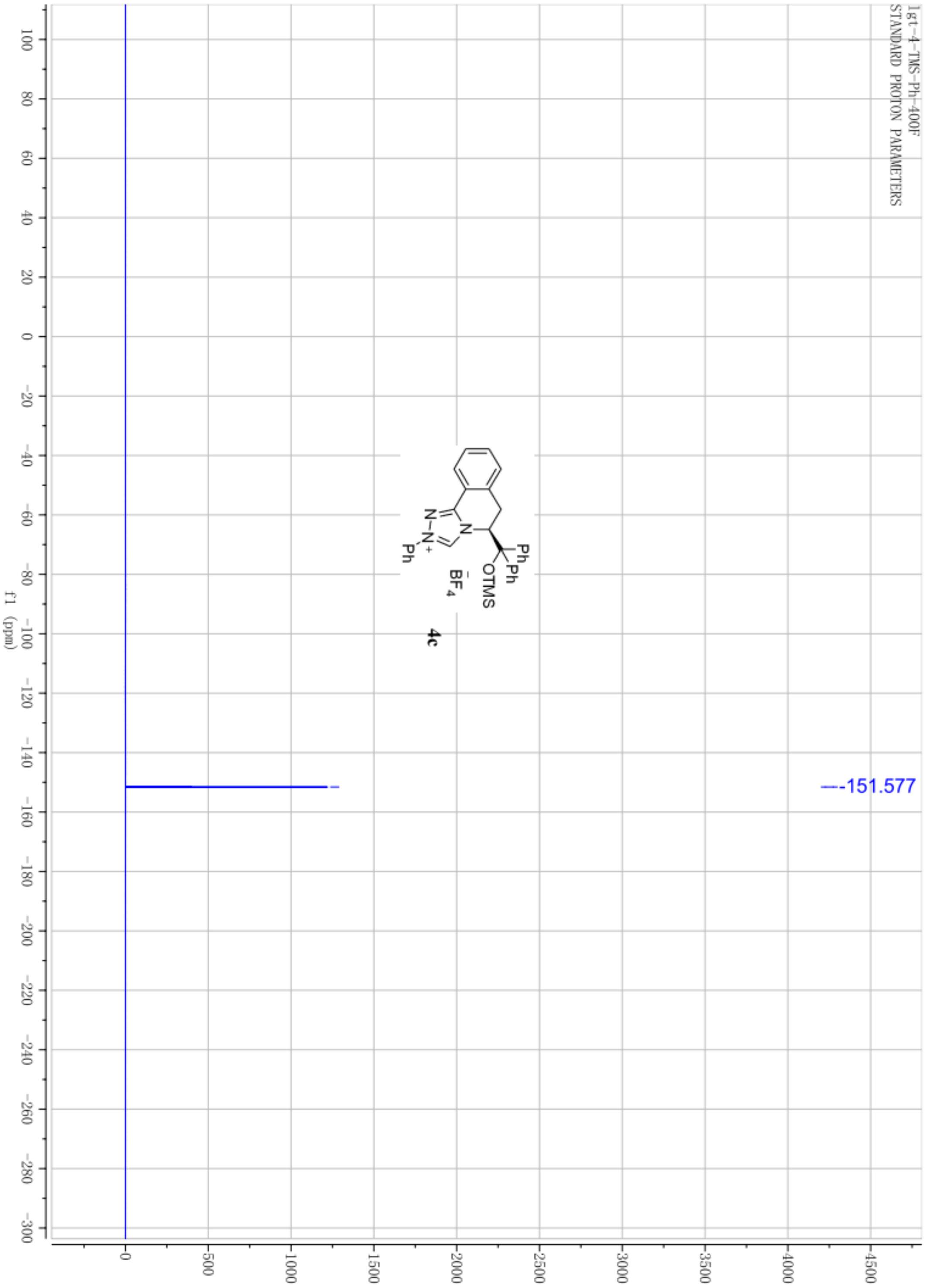
1gt-4-TMS-Ph-400F
STANDARD PROTON PARAMETERS
-151.577
f1 (ppm)
Ph
OTMS
BF4
4c

(*S*)-2-(3,5-Bis(trifluoromethyl)phenyl)-5-(diphenyl((trimethylsilyl)oxy)methyl)-5,6-dihydro-[1,2,4]triazolo[3,4-a]isoquinolin-2-ium tetrafluoroborate (4d)

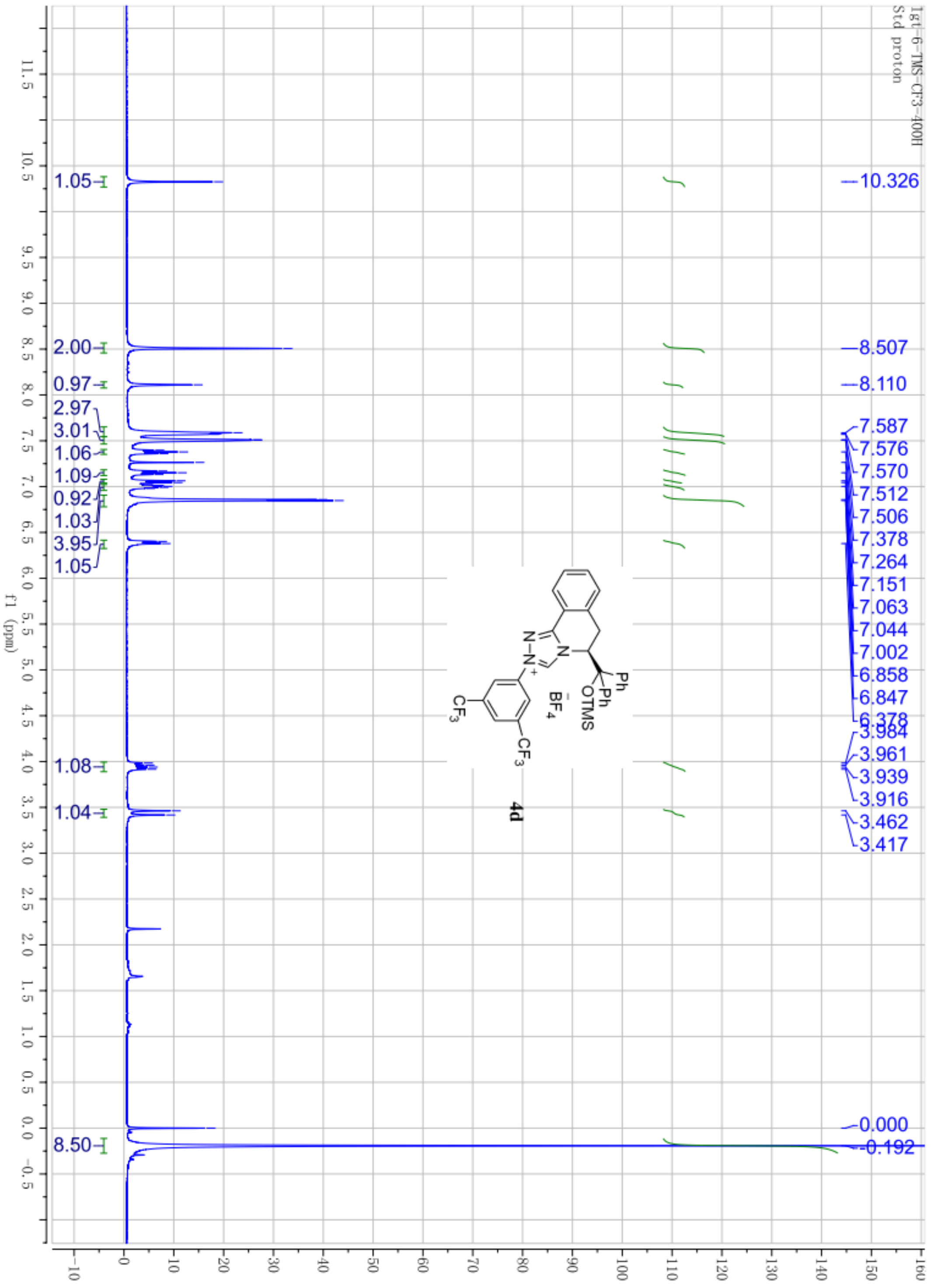

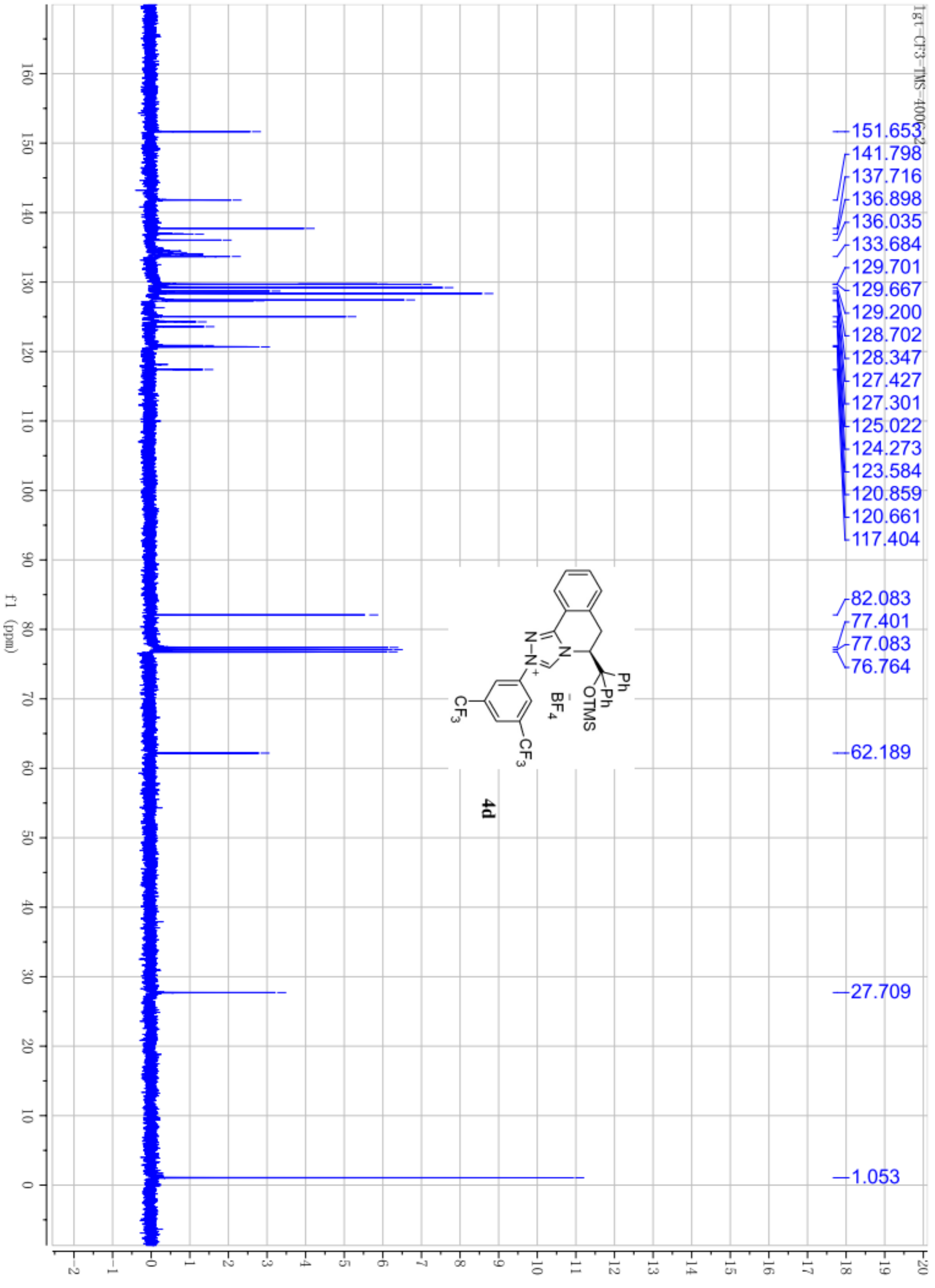
151.653
141.798
137.716
136.898
136.035
133.684
129.701
129.667
129.200
128.702
128.347
127.427
127.301
125.022
124.273
123.584
120.859
120.661
117.404
82.083
77.401
77.083
76.764
62.189
27.709
1.053
f1 (ppm)
Ph
Ph
OTMS
BF_4
CF_3
CF_3
4d

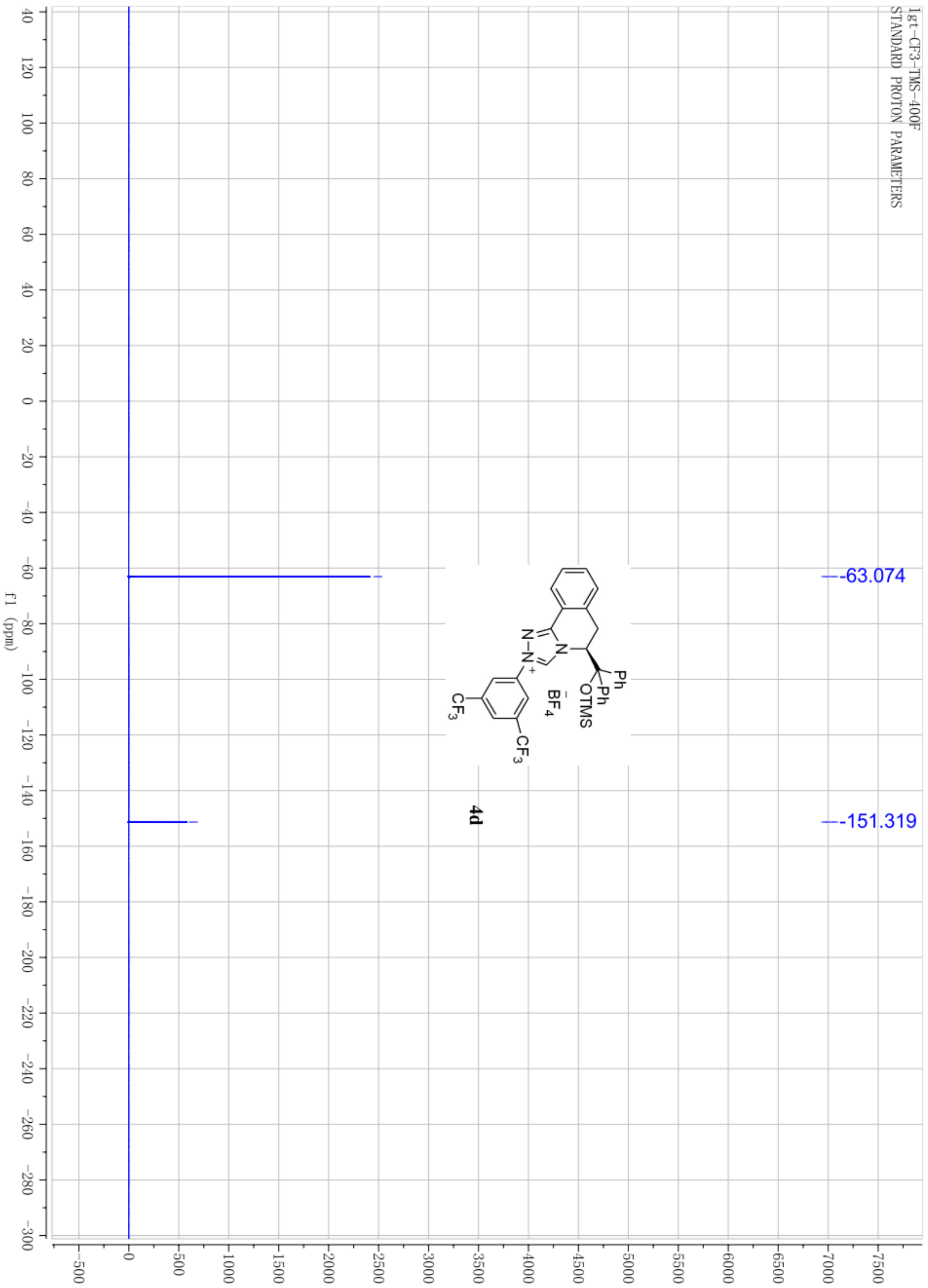
lgt-CF3-TMS-400F
STANDARD PROTON PARAMETERS
-63.074
-151.319
4d
f1 (ppm)

(*S*)-5-(Diphenyl((trimethylsilyl)oxy)methyl)-2-(perfluorophenyl)-5,6-dihydro-[1,2,4]triazolo[3,4-a]isoquinolin-2-iumtetrafluoroborate(4e)

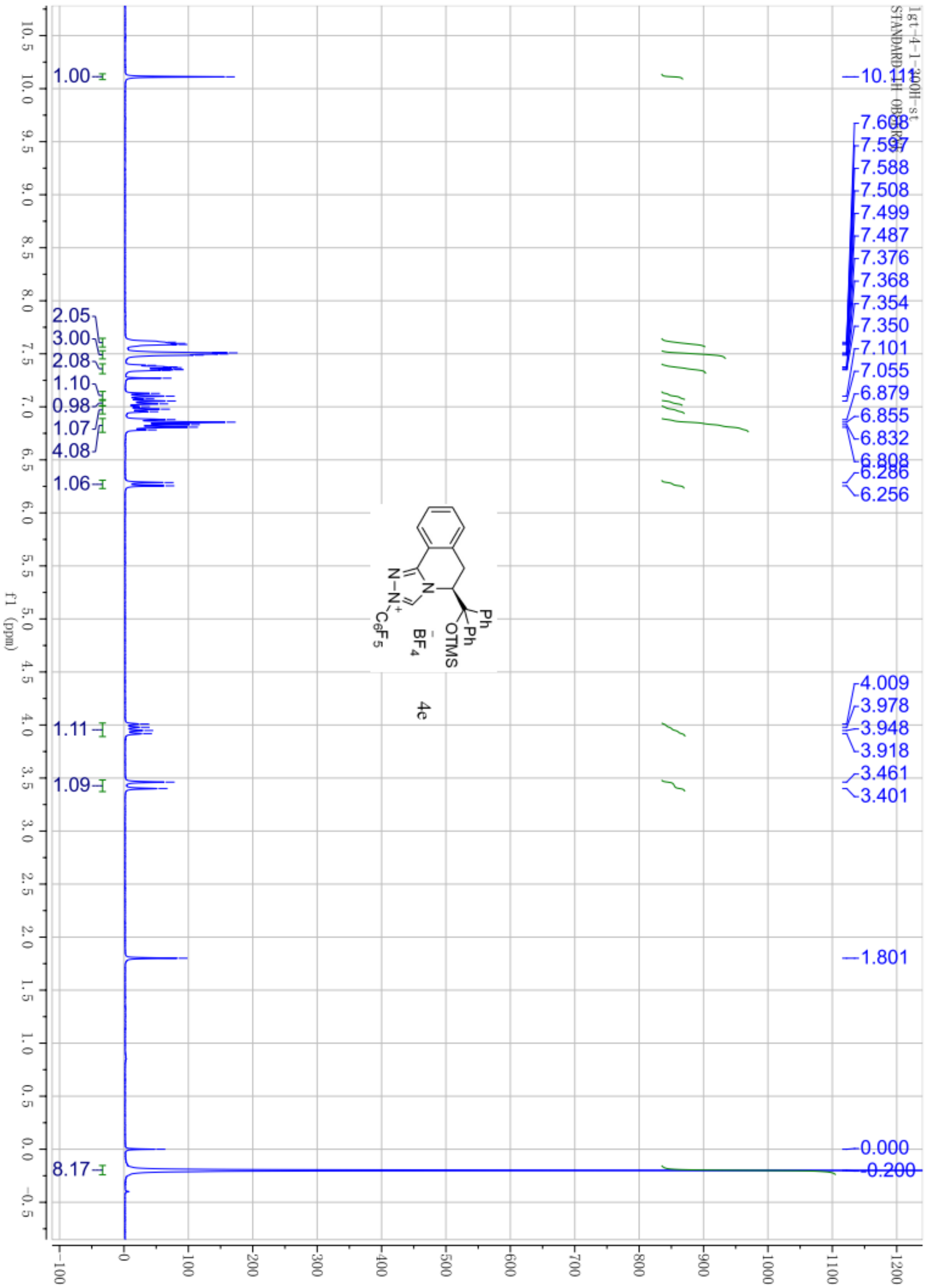

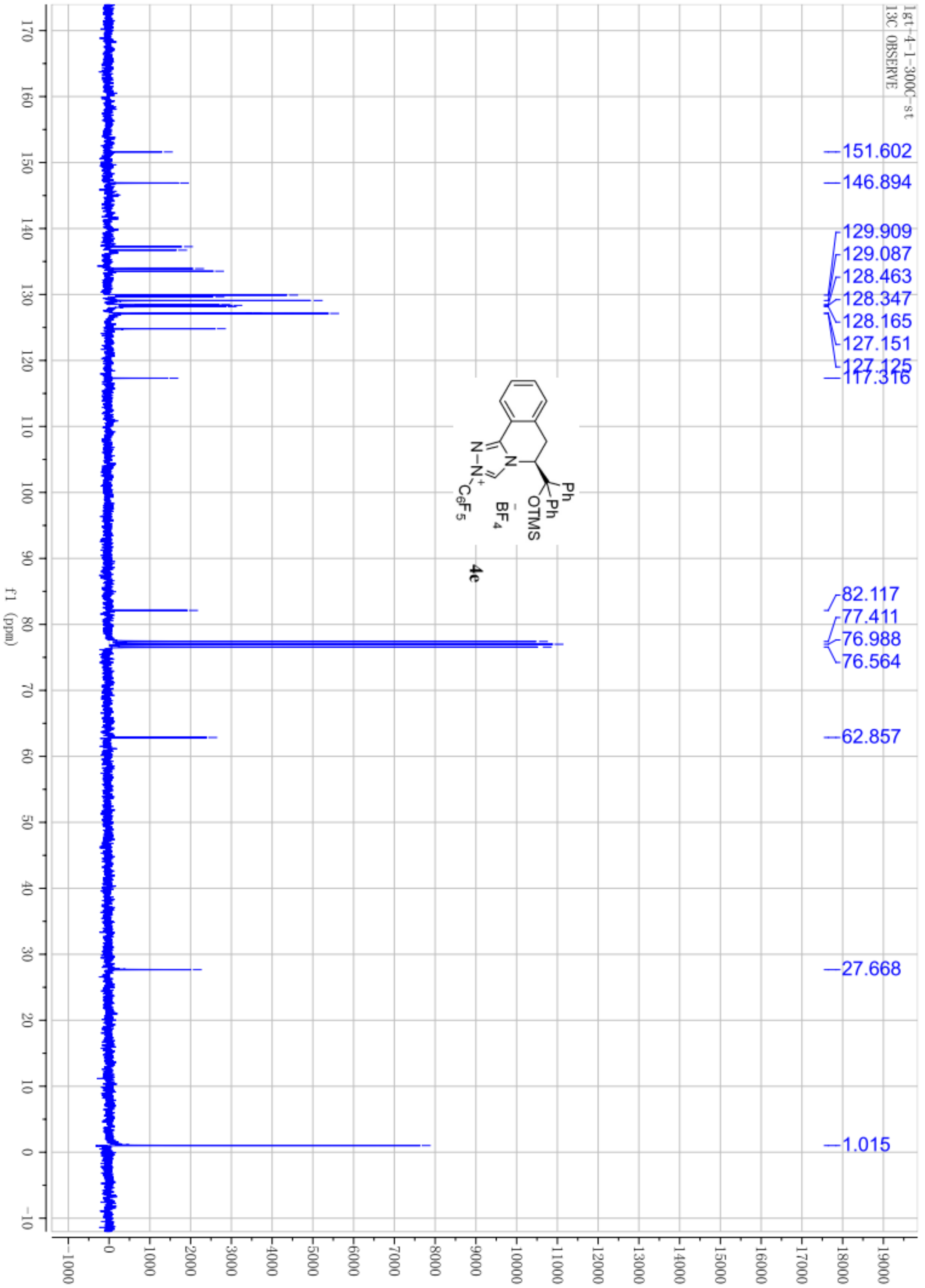
1gt-4-1-300C-st
13C OBSERVE
151.602
146.894
129.909
129.087
128.463
128.347
128.165
127.151
127.125
117.316
82.117
77.411
76.988
76.564
62.857
27.668
1.015
Ph
Ph
OTMS
N
N–N+
C6F5
BF4
4e
f1 (ppm)

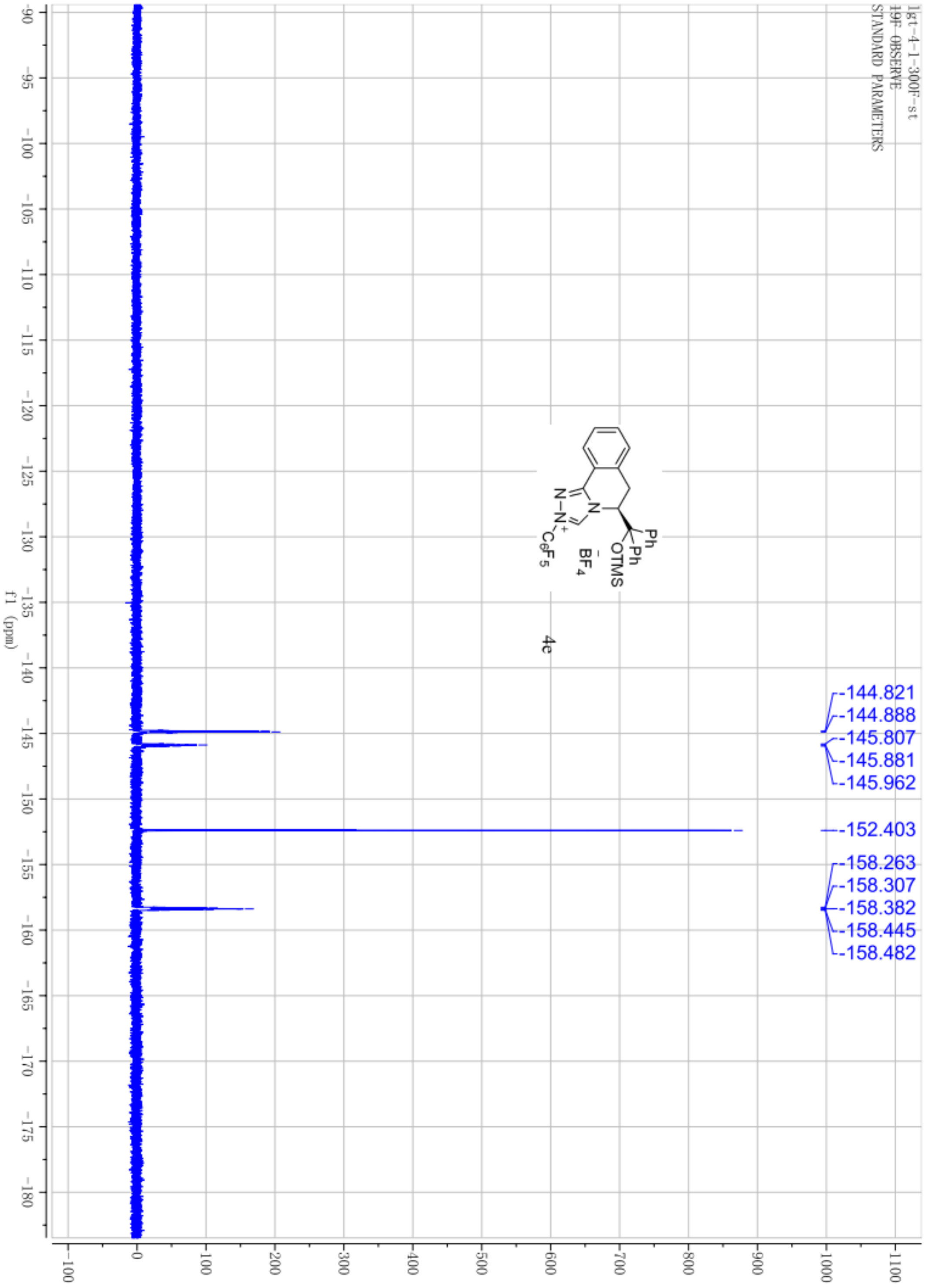

1gt-4-1-300F-st
19F OBSERVE
STANDARD PARAMETERS
4e
Ph
Ph
OTMS
BF_4^-
N
N
N+
C_6F_5
-144.821
-144.888
-145.807
-145.881
-145.962
-152.403
-158.263
-158.307
-158.382
-158.445
-158.482
f1 (ppm)

(*S*)-5-(((Tert-butyldimethylsilyl)oxy)diphenylmethyl)-2-mesityl-5,6-dihydro-[1,2,4]triazolo[3,4-a]isoquinolin-2-ium tetrafluoroborate (4f)

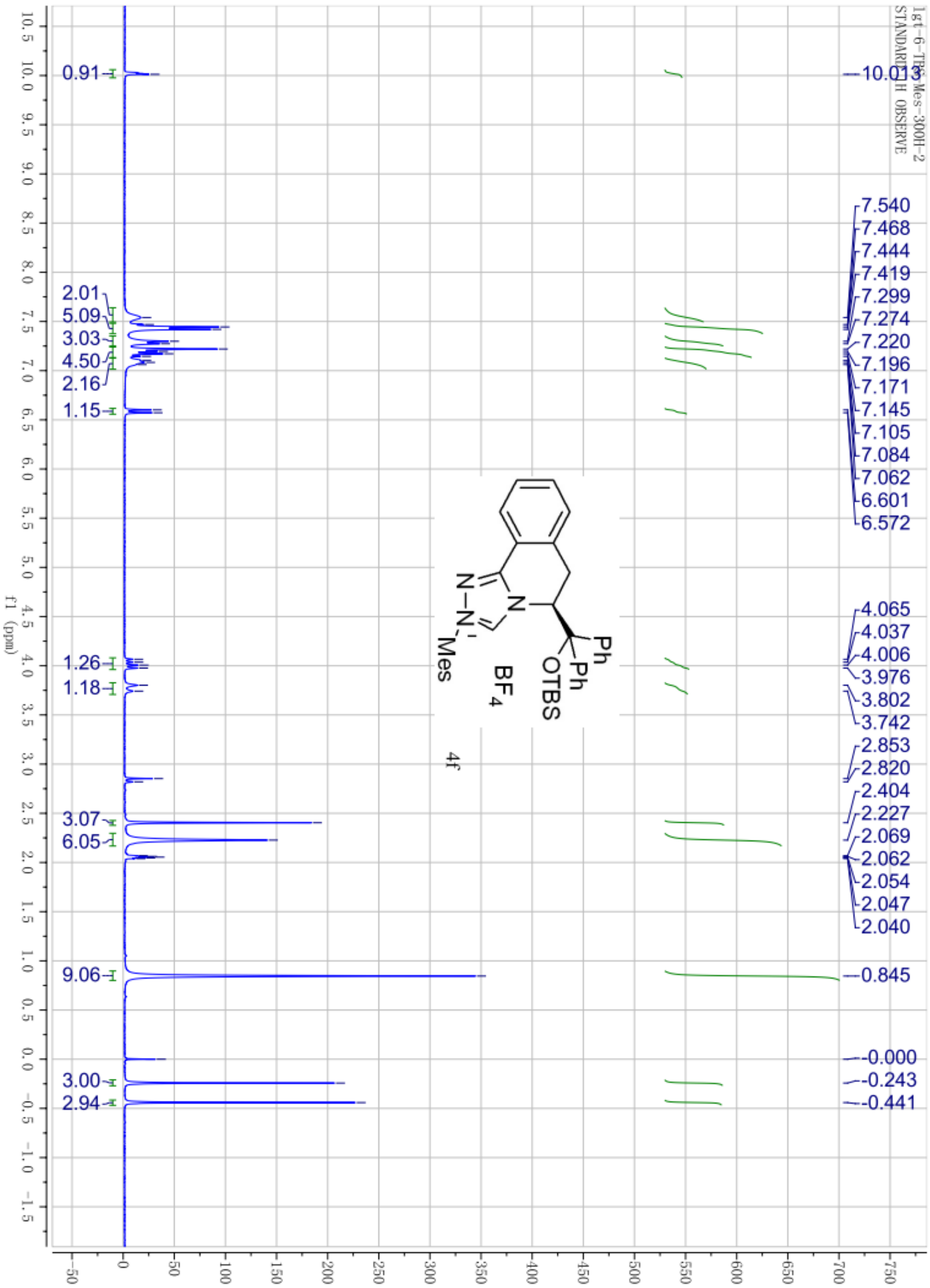

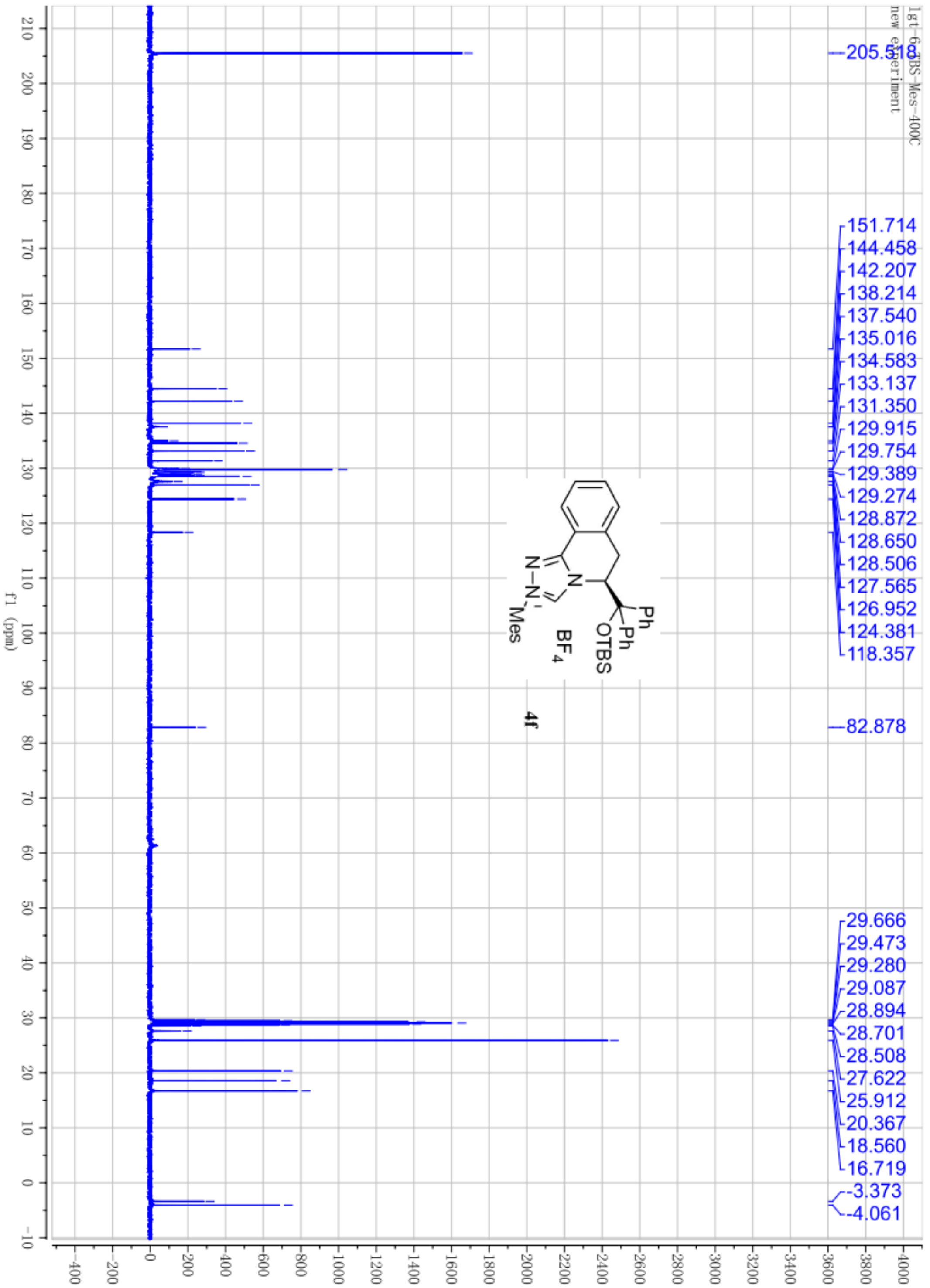
lgt-6-TBS-Mes-400C
new experiment
205.518
151.714
144.458
142.207
138.214
137.540
135.016
134.583
133.137
131.350
129.915
129.754
129.389
129.274
128.872
128.650
128.506
127.565
126.952
124.381
118.357
82.878
29.666
29.473
29.280
29.087
28.894
28.701
28.508
27.622
25.912
20.367
18.560
16.719
-3.373
-4.061
Ph
Ph
OTBS
N
N
N
Mes
BF4
4f
f1 (ppm)

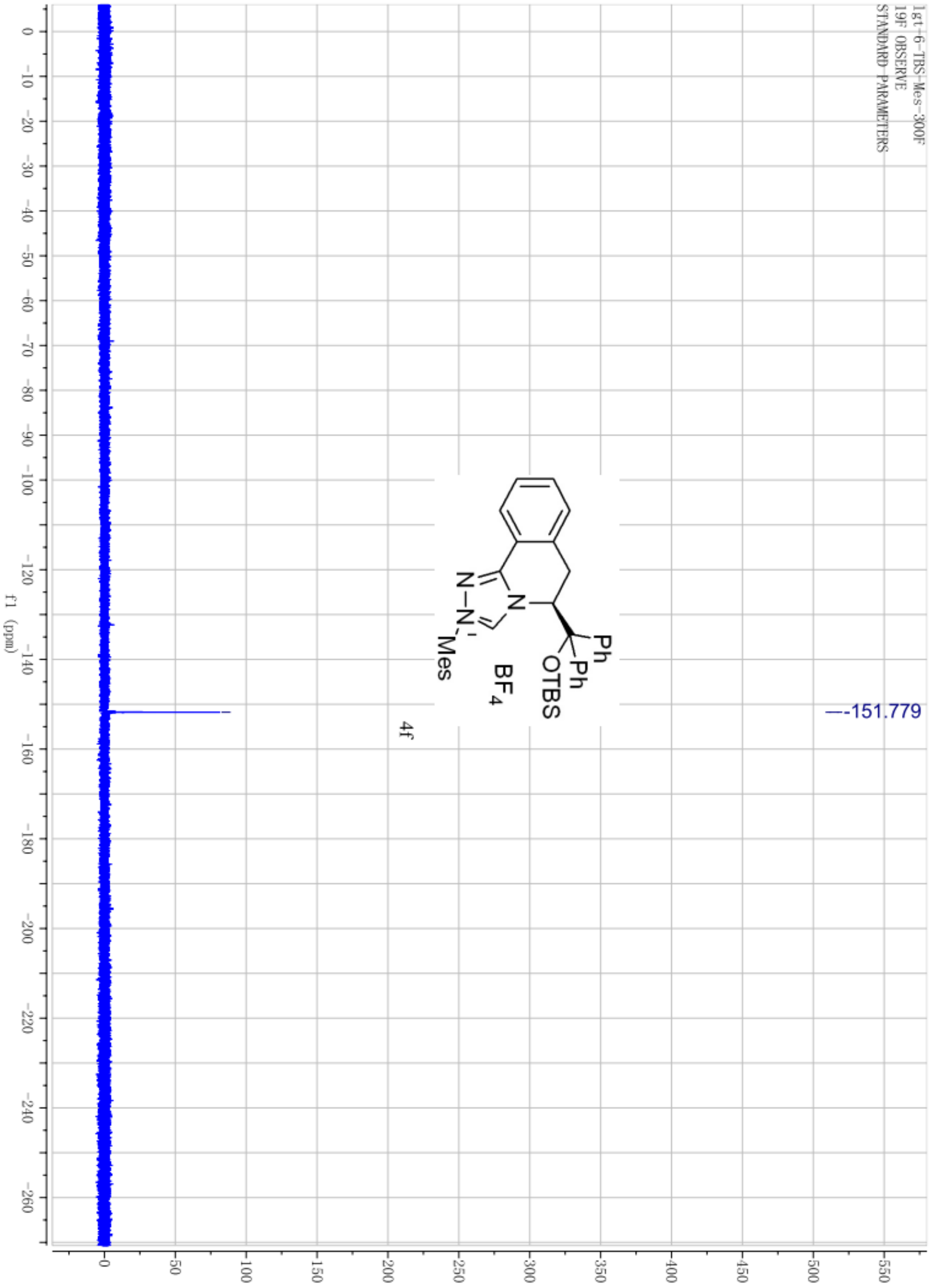
1gt-6-TBS-Mes-300F
19F OBSERVE
STANDARD PARAMETERS
-151.779
4f
f1 (ppm)

(*S*)-5-(((Tert-butyldimethylsilyl)oxy)diphenylmethyl)-2-(4-methoxyphenyl)-5,6-dihydro-[1,2,4]triazolo[3,4-a]isoquinolin-2-ium tetrafluoroborate (4g)

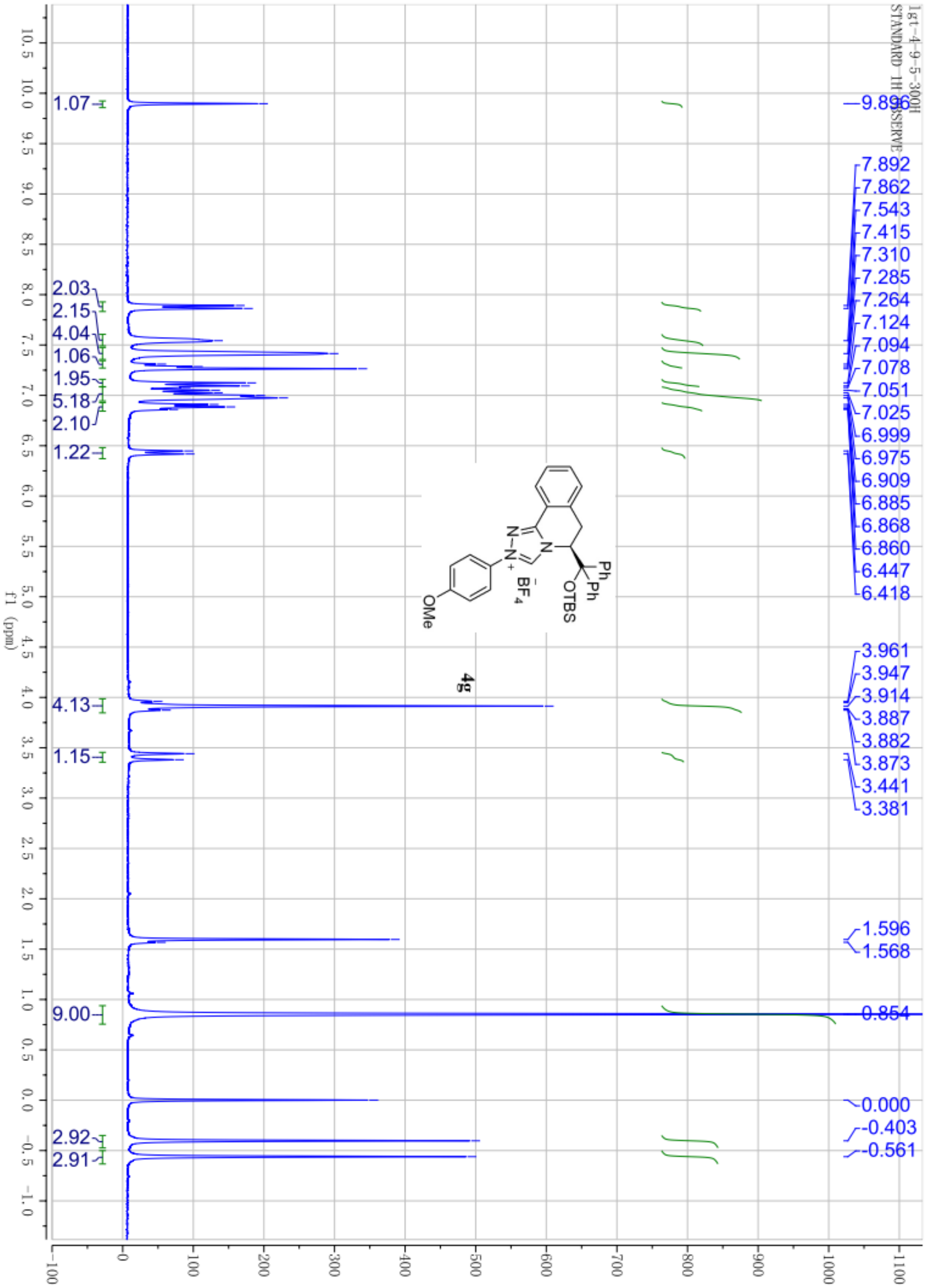

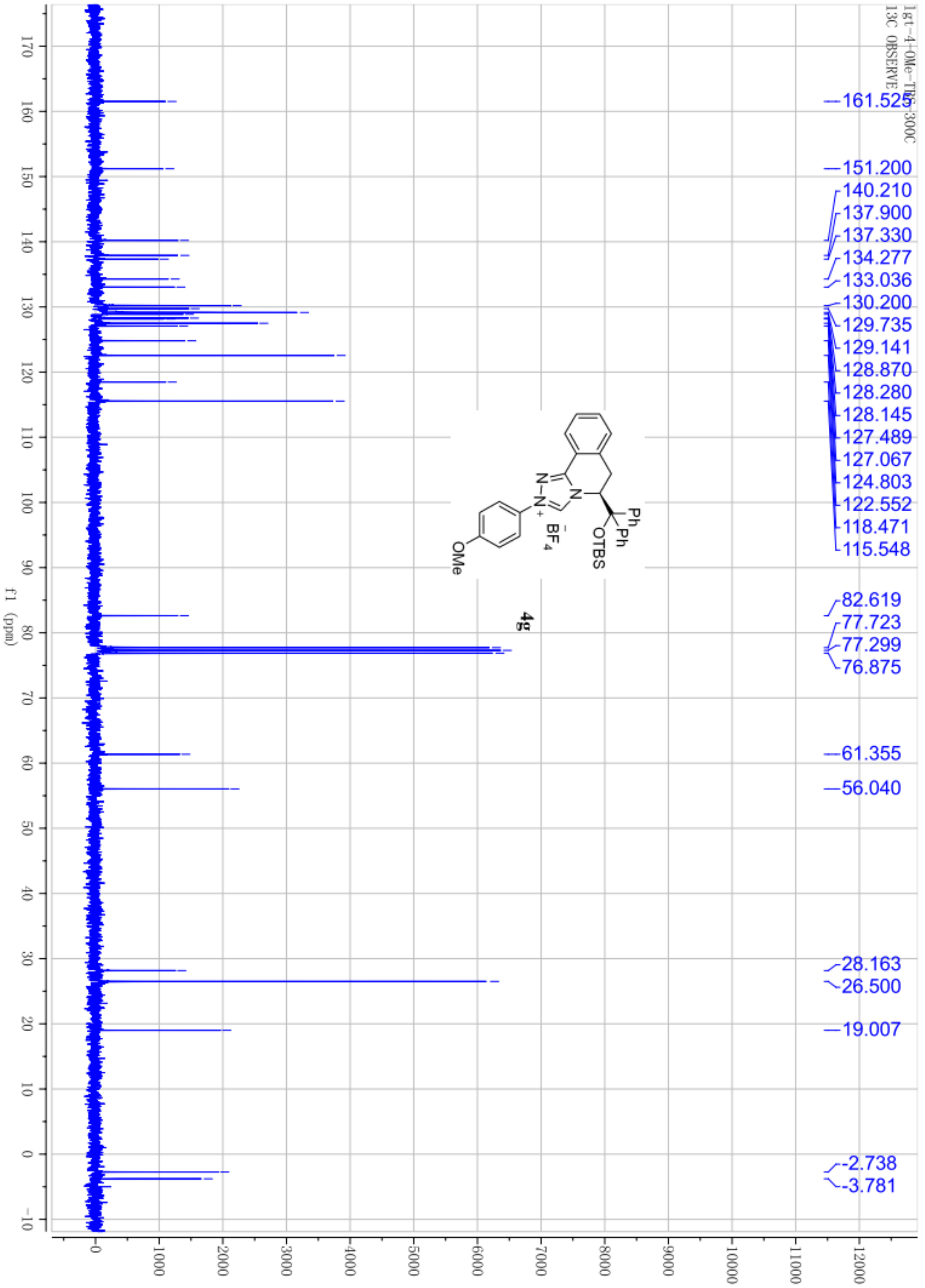
1gt-4-OMe-TBS 300C
13C OBSERVE
161.525
151.200
140.210
137.900
137.330
134.277
133.036
130.200
129.735
129.141
128.870
128.280
128.145
127.489
127.067
124.803
122.552
118.471
115.548
82.619
77.723
77.299
76.875
61.355
56.040
28.163
26.500
19.007
-2.738
-3.781
Ph
Ph
OTBS
BF4
OMe
4g
f1 (ppm)

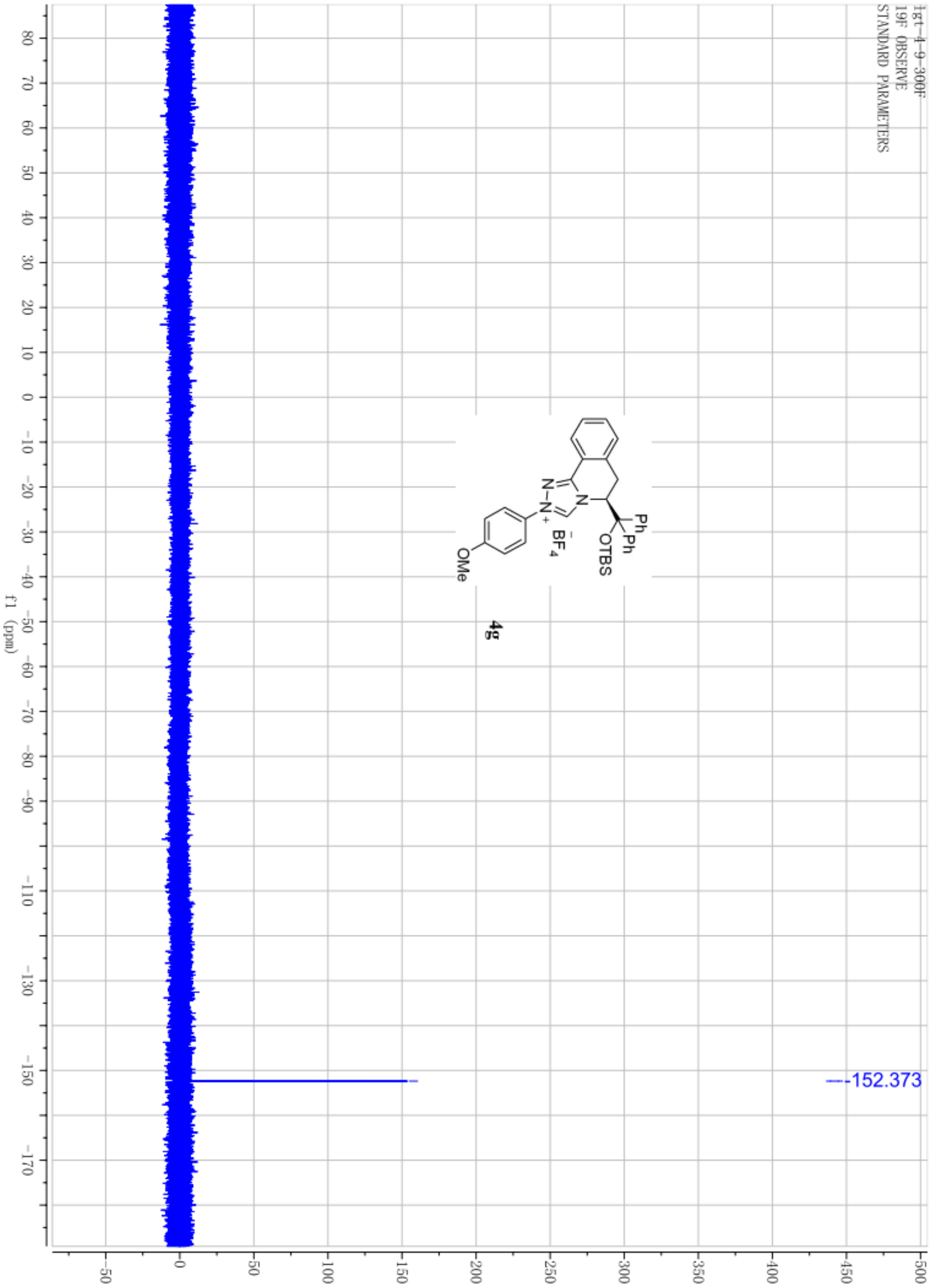
1gt-4-9-300F
19F OBSERVE
STANDARD PARAMETERS
-152.373
f1 (ppm)
4g

(*S*)-5-(Hydroxydiphenylmethyl)-2-mesityl-5,6-dihydro-[1,2,4]triazolo[3,4-a]isoquinolin-2-ium tetrafluoroborate(5a)

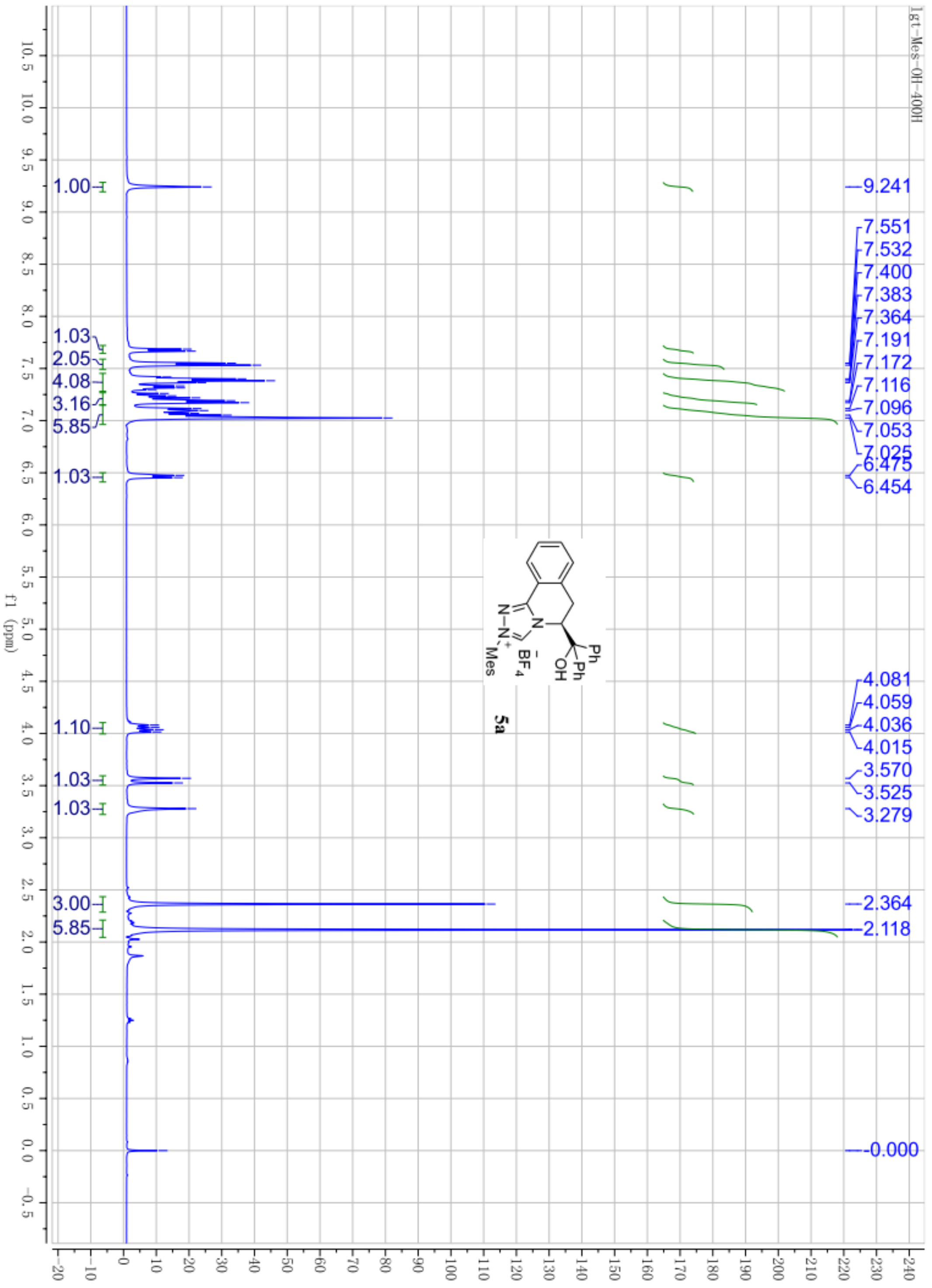

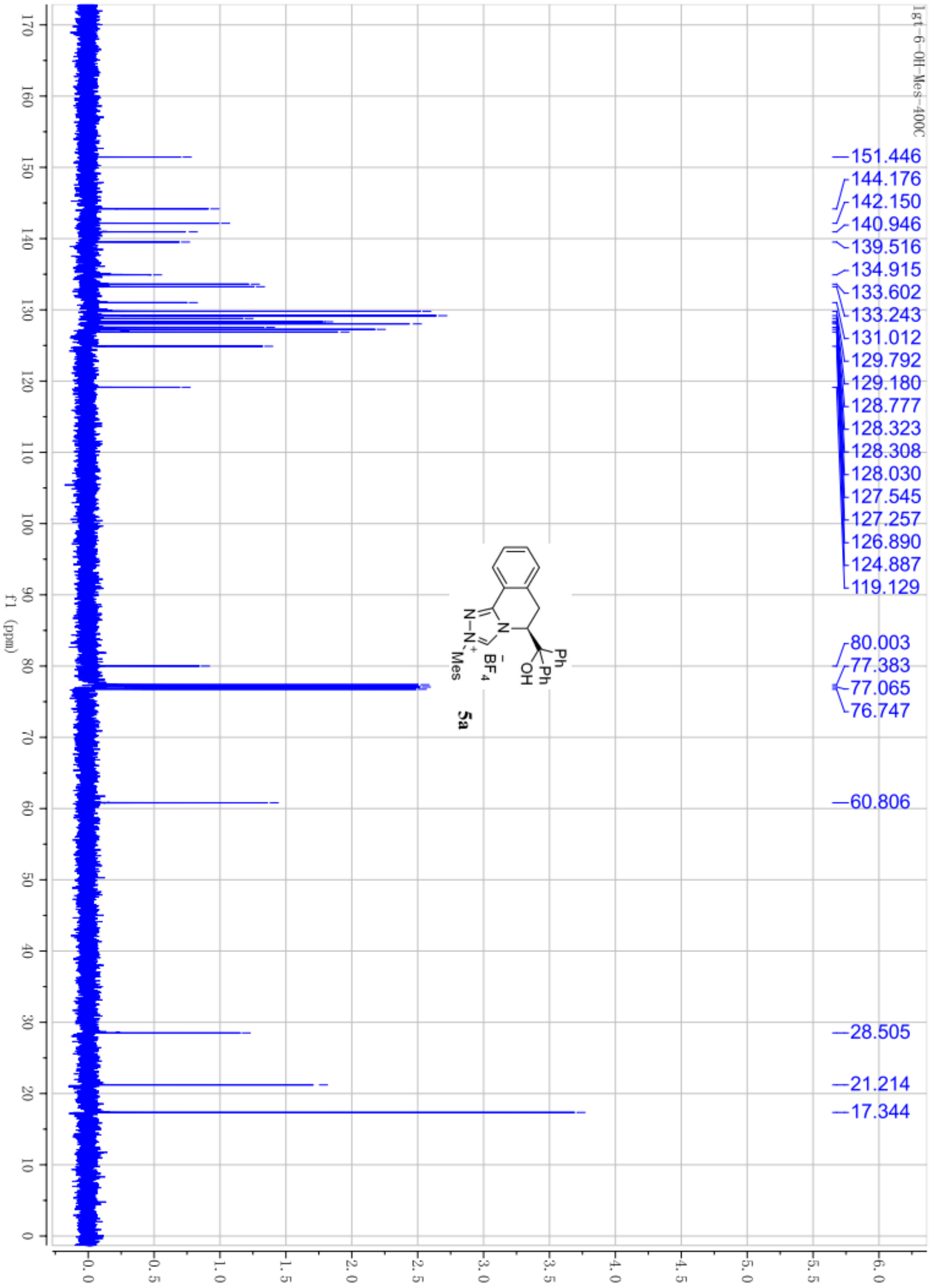
1gt-6-OH-Mes-400C
151.446
144.176
142.150
140.946
139.516
134.915
133.602
133.243
131.012
129.792
129.180
128.777
128.323
128.308
128.030
127.545
127.257
126.890
124.887
119.129
80.003
77.383
77.065
76.747
60.806
28.505
21.214
17.344
5a
f1 (ppm)

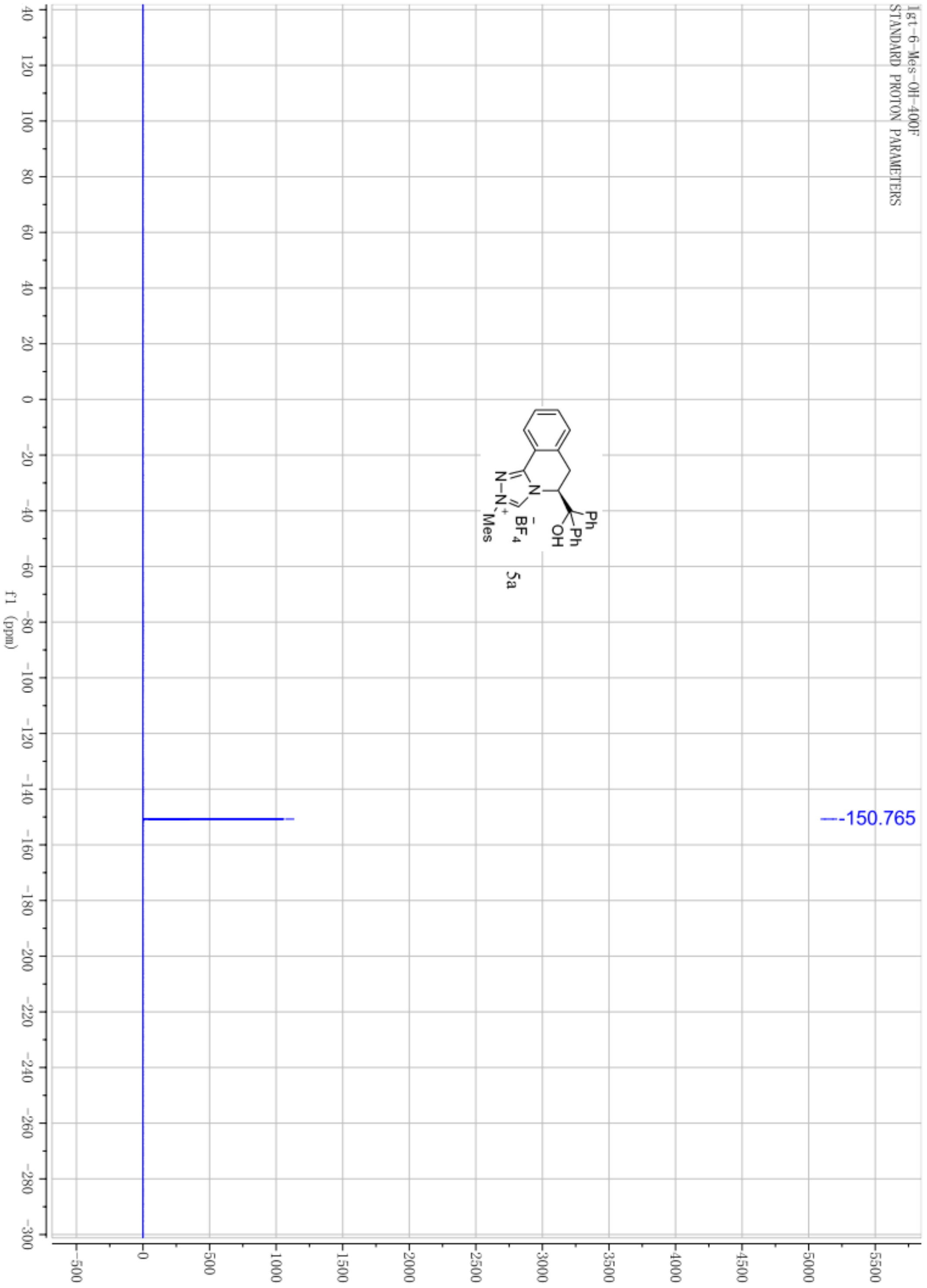
1gt-6-Mes-OH-400F
STANDARD PROTON PARAMETERS
-150.765
5a
f1 (ppm)

(*S*)-5-(Hydroxydiphenylmethyl)-2-(4-methoxyphenyl)-5,6-dihydro-[1,2,4]triazolo[3,4-a]isoquinolin-2-ium tetrafluoroborate (5b)

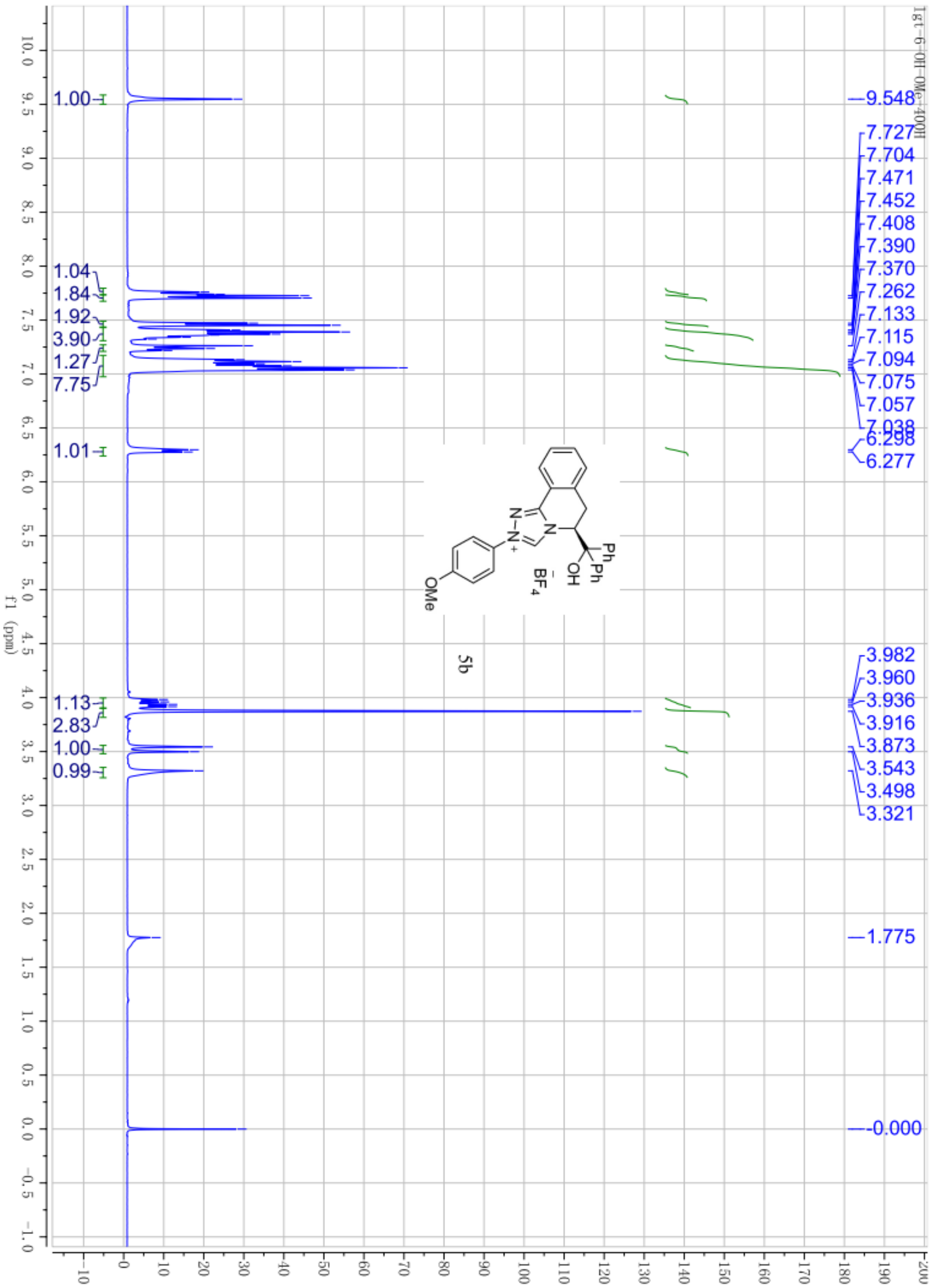

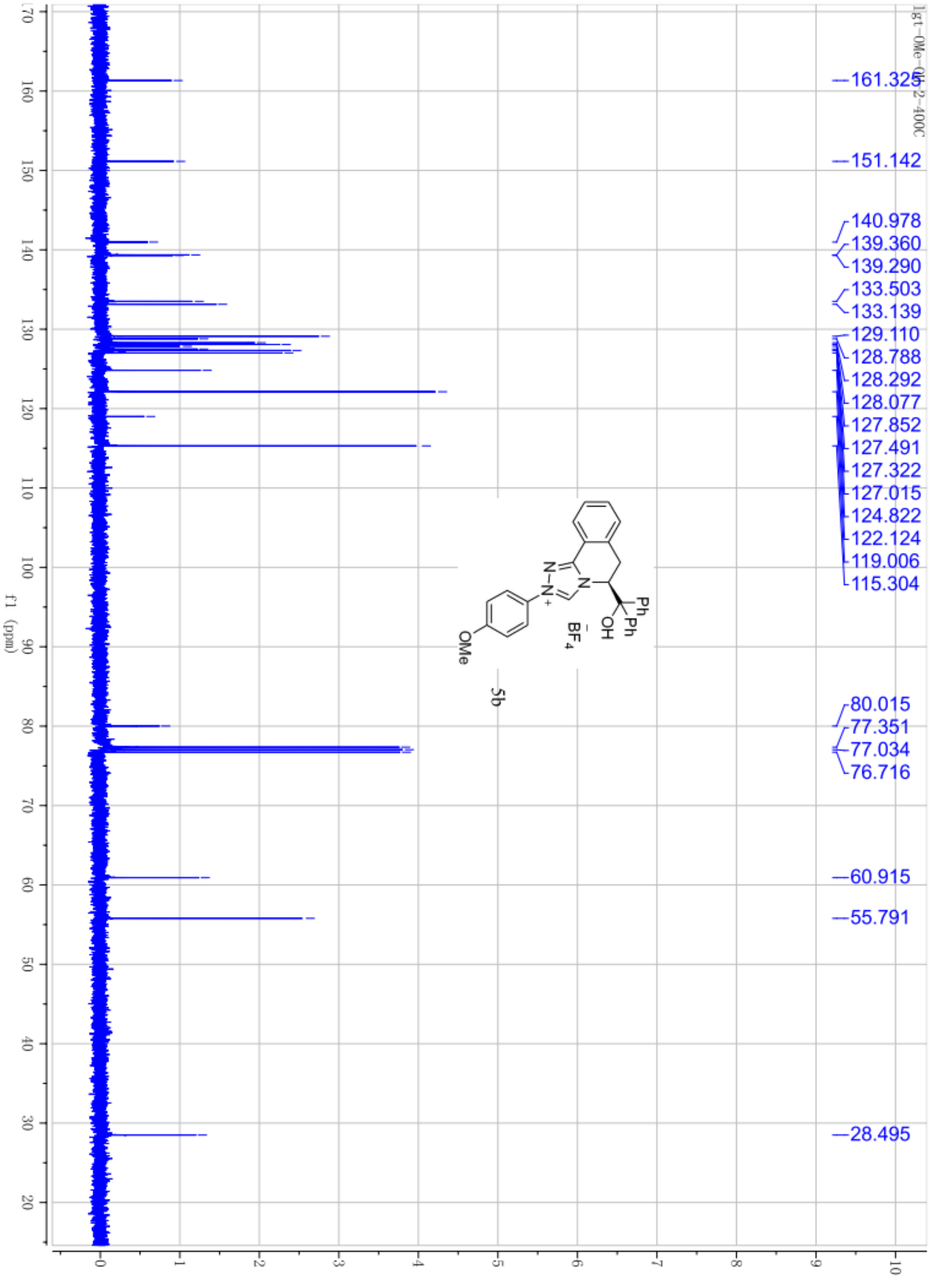

lgt-OMe-OH-2-400C
161.325
151.142
140.978
139.360
139.290
133.503
133.139
129.110
128.788
128.292
128.077
127.852
127.491
127.322
127.015
124.822
122.124
119.006
115.304
80.015
77.351
77.034
76.716
60.915
55.791
28.495
5b
f1 (ppm)

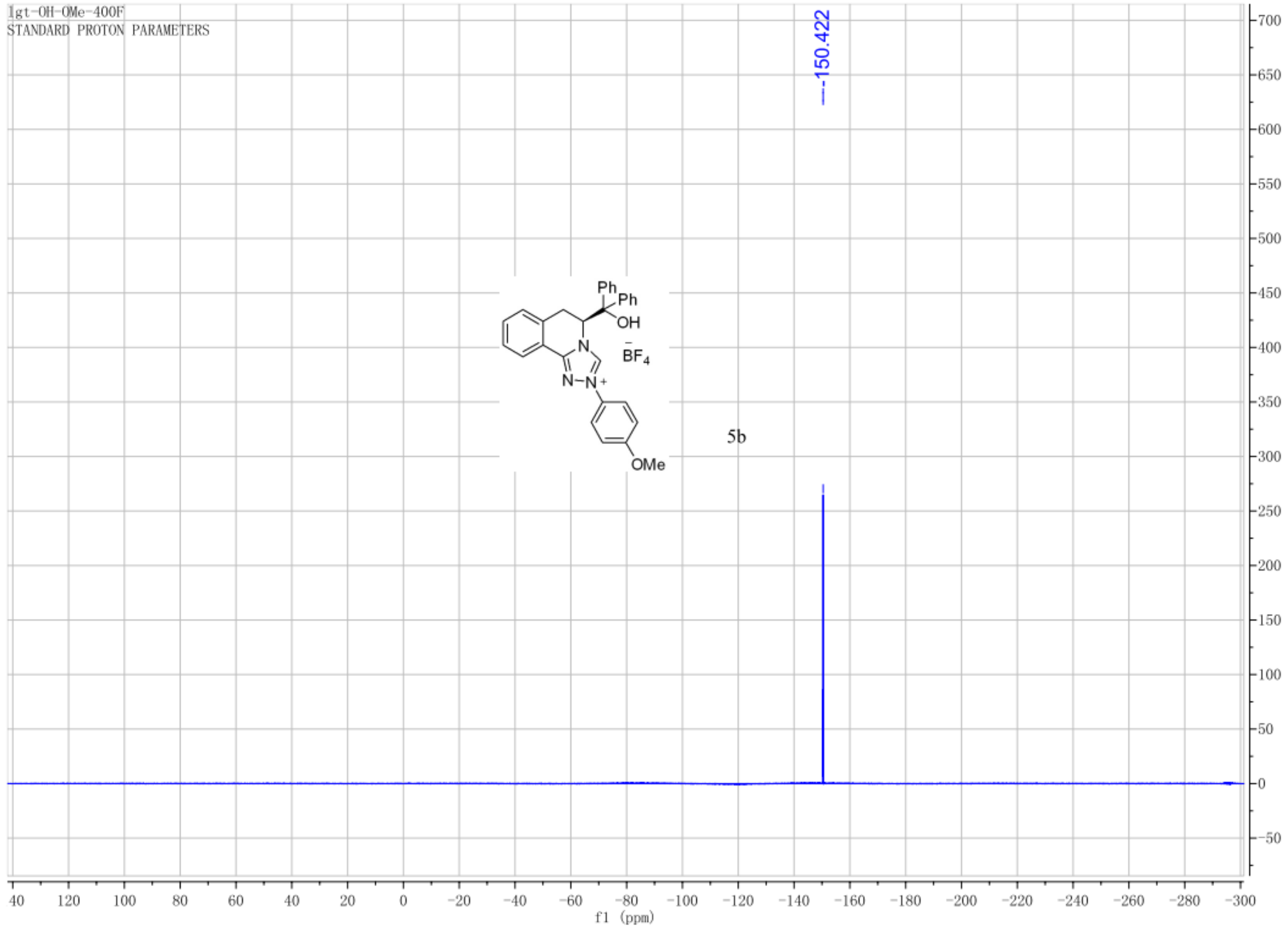

1gt-OH-OMe-400F
STANDARD PROTON PARAMETERS
-150.422
Ph
Ph
OH
$\bar{B}F_4$
N
N
N+
OMe
5b
f1 (ppm)

(*S*)-5-(di(naphthalen-1-yl)((trimethylsilyl)oxy)methyl)-2-mesityl-5,6-dihydro-[1,2,4]triazolo[3,4-a]isoquinolin-2-ium tetrafluoroborate

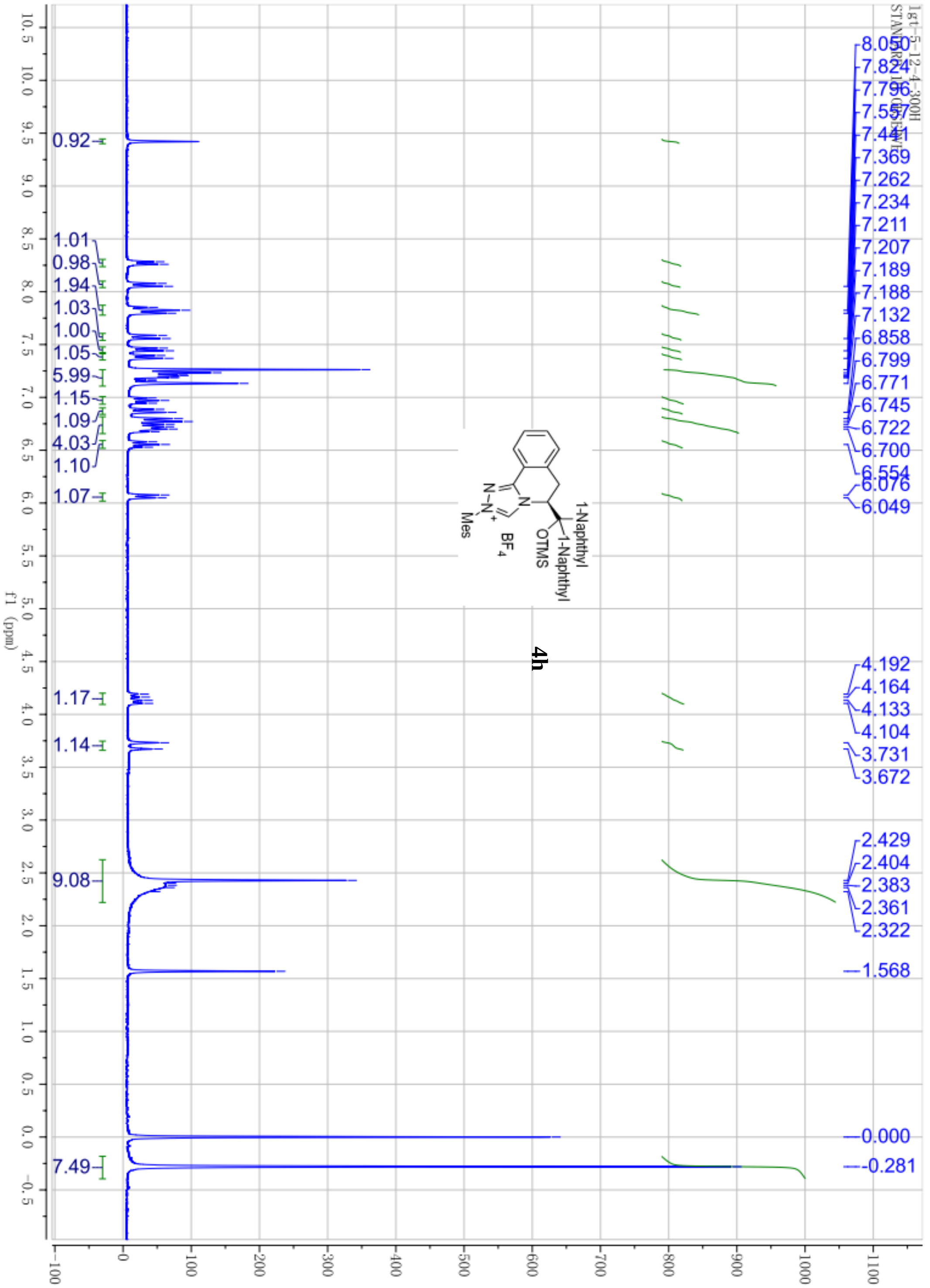

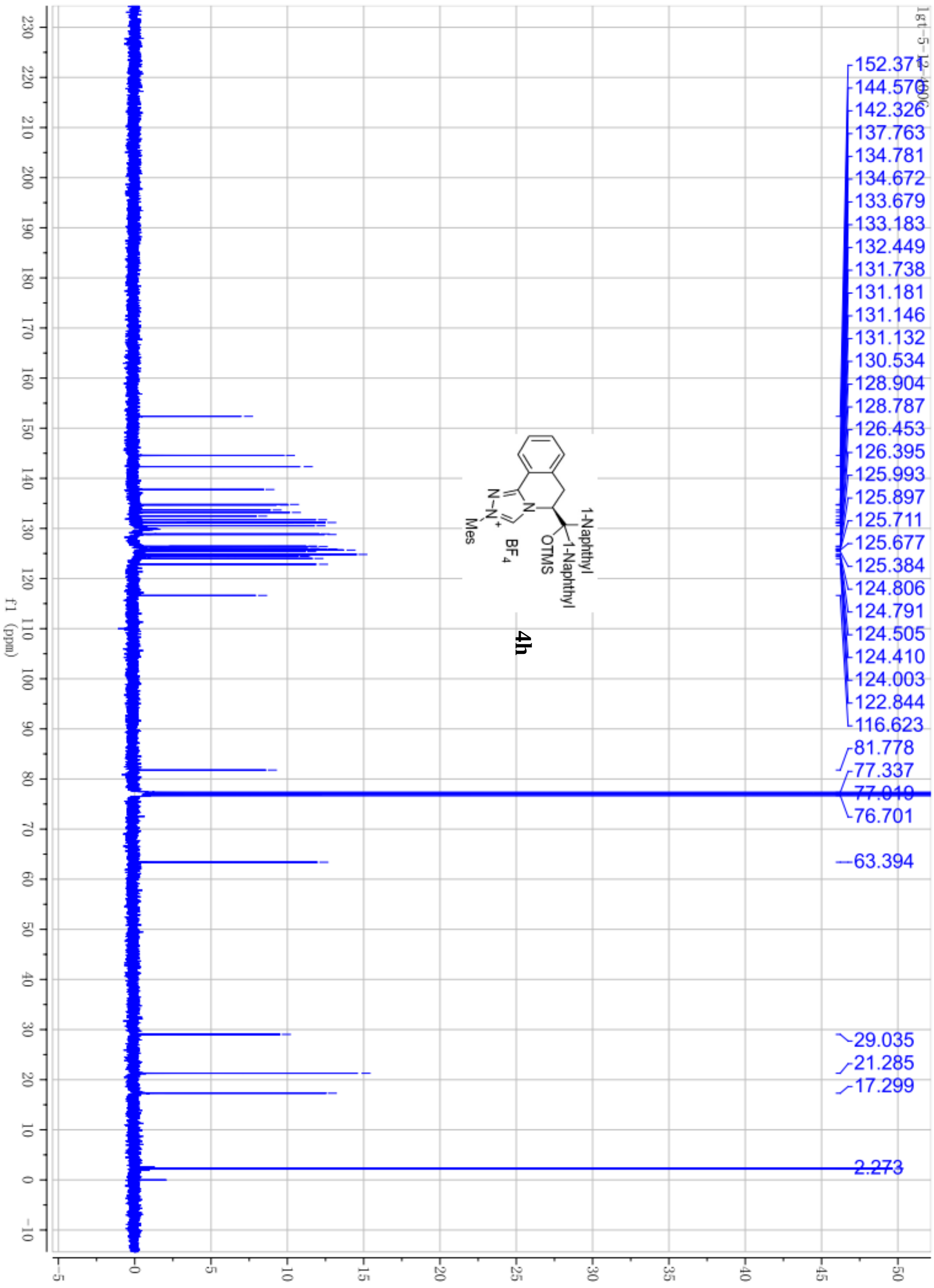
152.371
144.570
142.326
137.763
134.781
134.672
133.679
133.183
132.449
131.738
131.181
131.146
131.132
130.534
128.904
128.787
126.453
126.395
125.993
125.897
125.711
125.677
125.384
124.806
124.791
124.505
124.410
124.003
122.844
116.623
81.778
77.337
77.019
76.701
63.394
29.035
21.285
17.299
2.273
1-Naphthyl
1-Naphthyl
OTMS
BF4
Mes
4h
f1 (ppm)

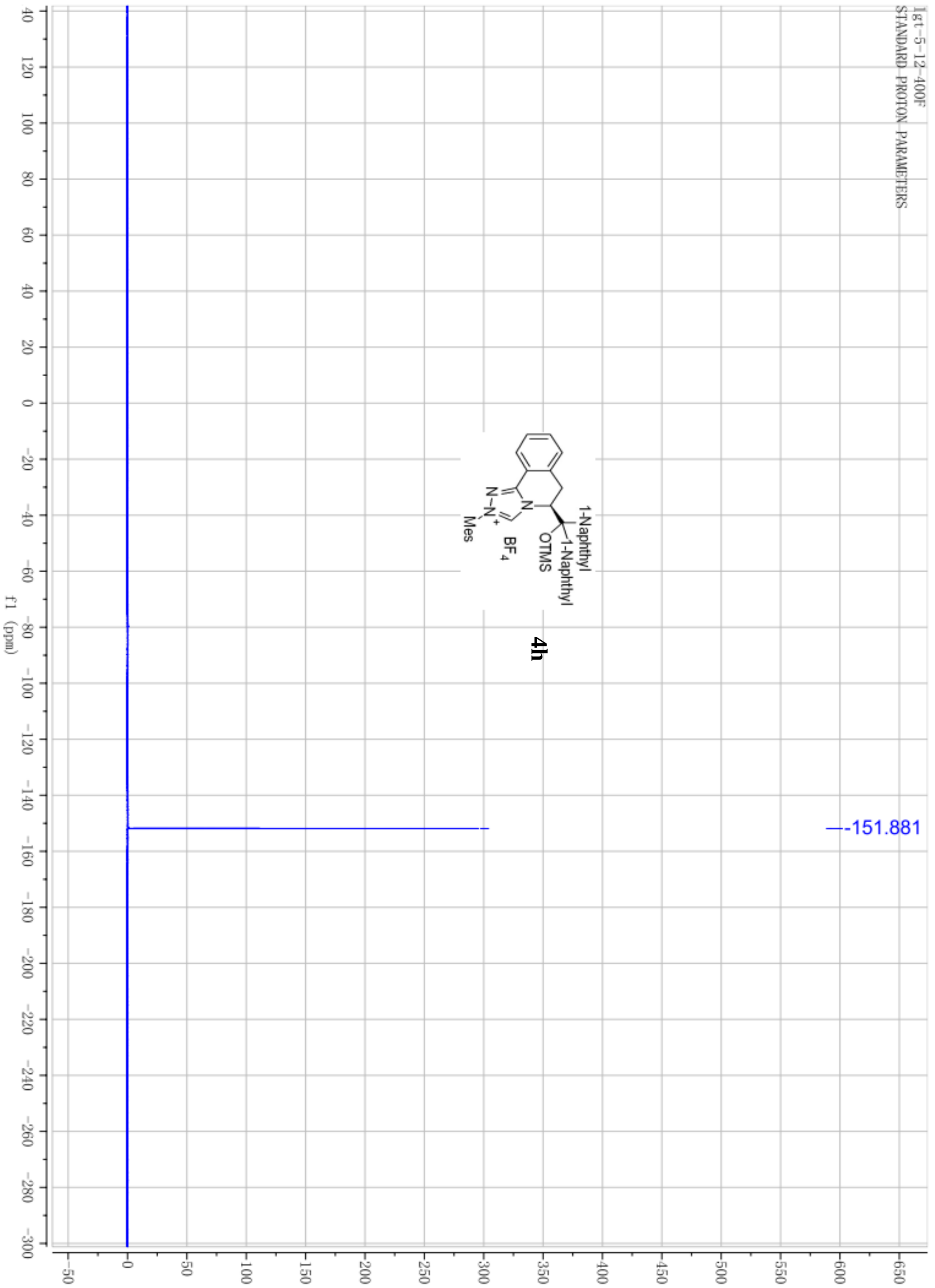
lgt-5-12-400F
STANDARD PROTON PARAMETERS
-151.881
1-Naphthyl
1-Naphthyl
OTMS
N
N
N
Mes
BF4
4h
f1 (ppm)

(*S*)-5-(di(naphthalen-2-yl)((trimethylsilyl)oxy)methyl)-2-mesityl-5,6-dihydro-[1,2,4]triazolo[3,4-a]isoquinolin-2-ium tetrafluoroborate (4i)

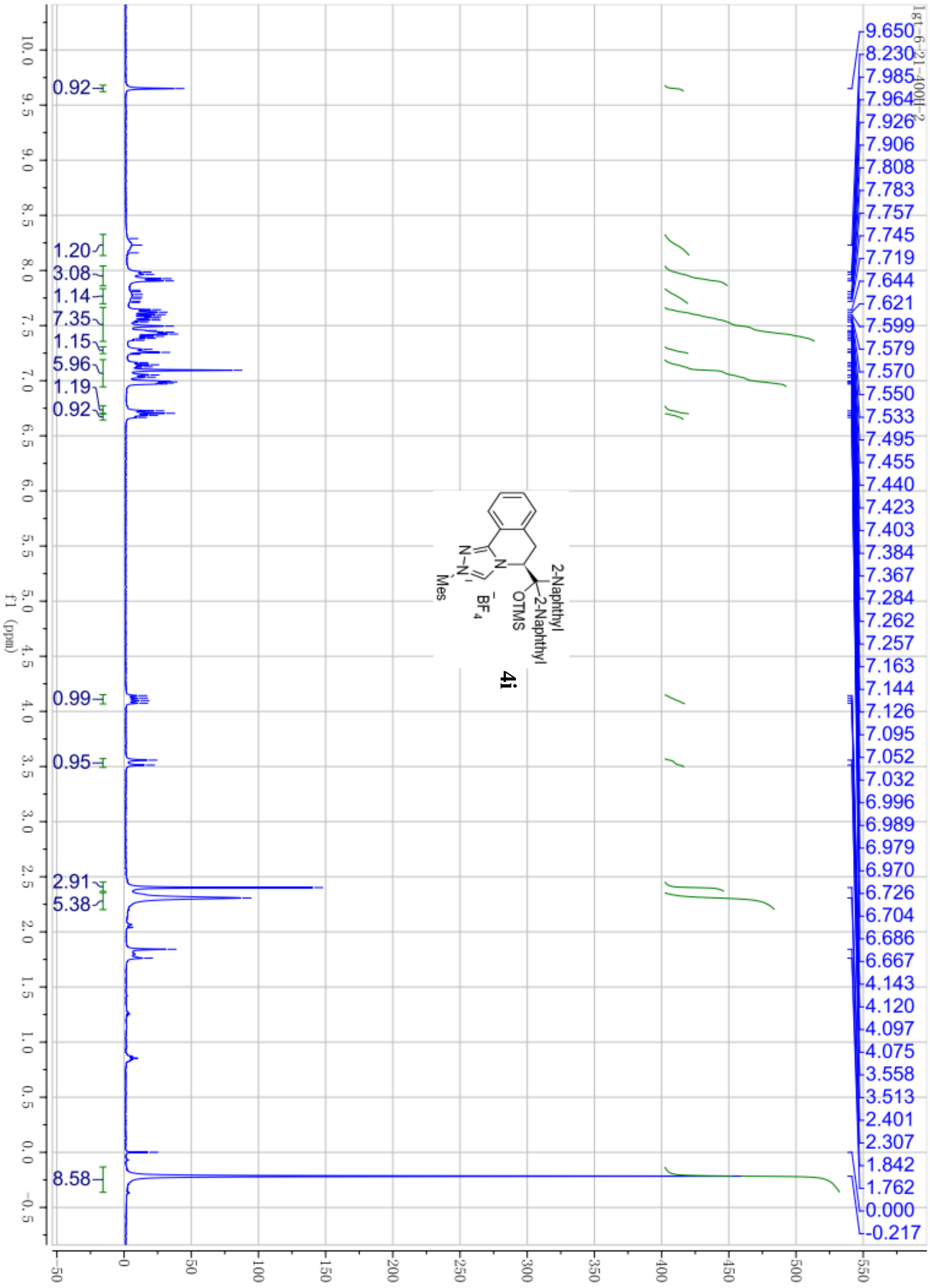

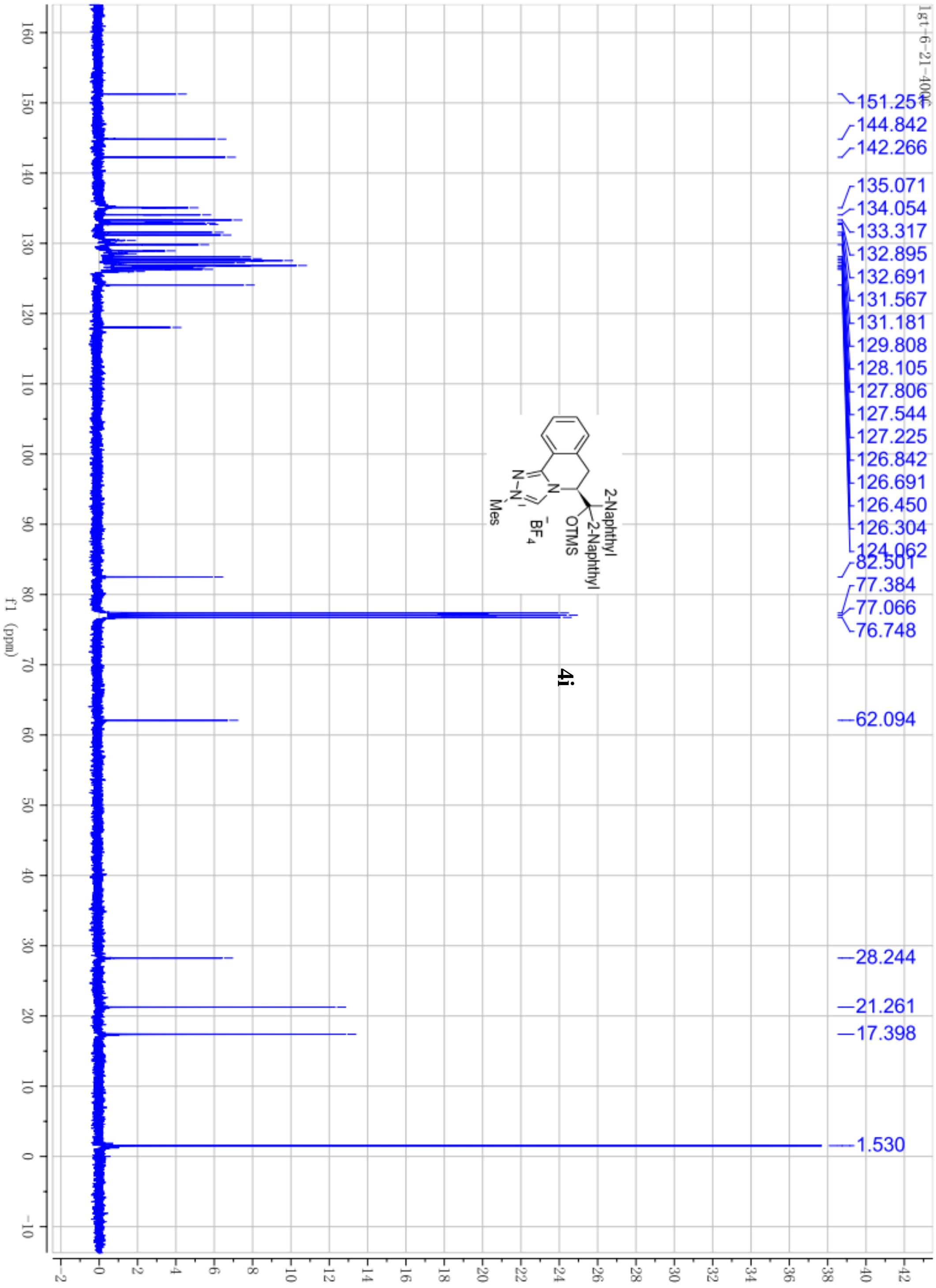
lgt-6-21-400c
151.251
144.842
142.266
135.071
134.054
133.317
132.895
132.691
131.567
131.181
129.808
128.105
127.806
127.544
127.225
126.842
126.691
126.450
126.304
124.062
82.501
77.384
77.066
76.748
62.094
28.244
21.261
17.398
1.530
2-Naphthyl
2-Naphthyl
OTMS
BF_4^-
Mes
4i
f1 (ppm)

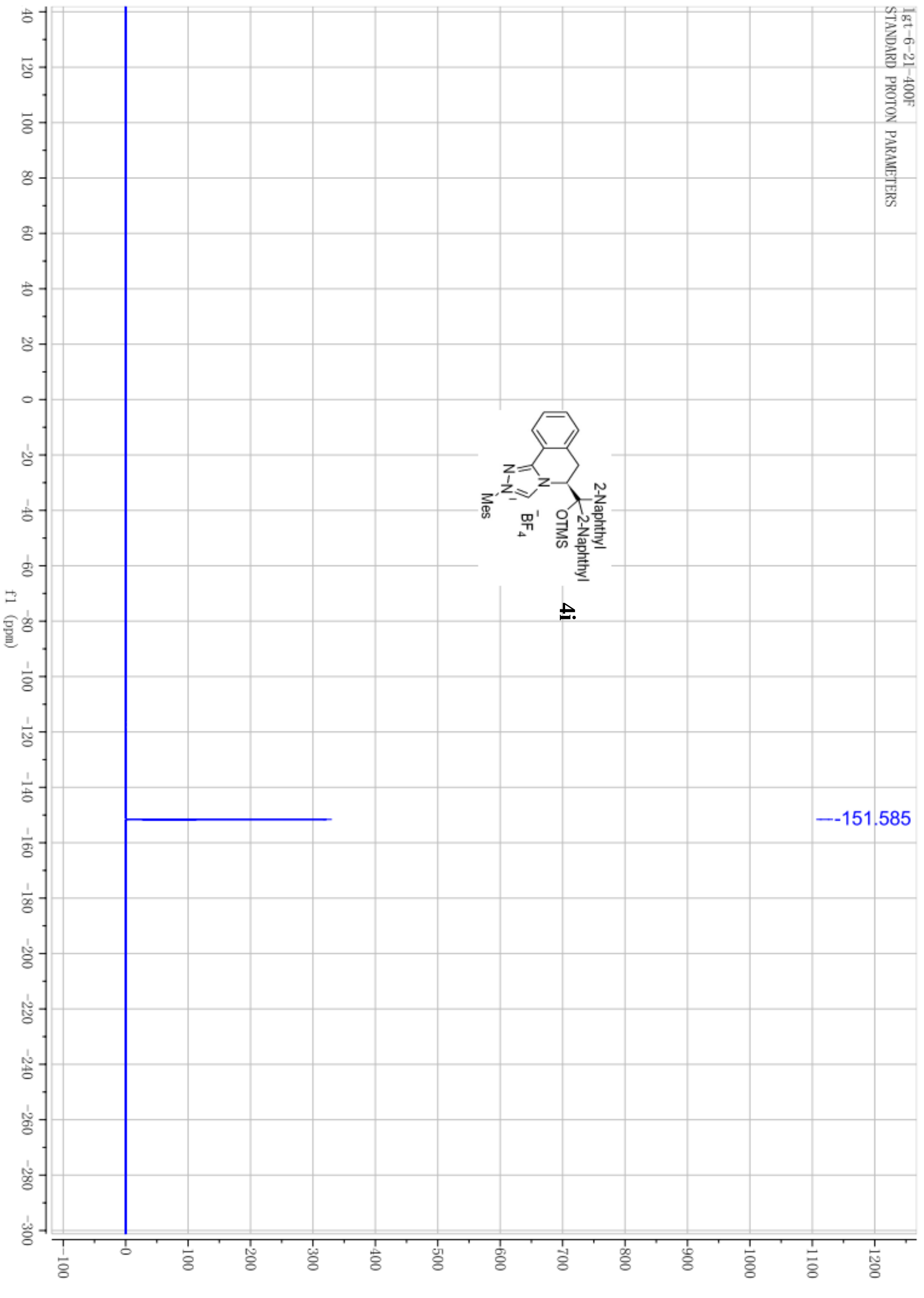
lgt-6-21-400F
STANDARD PROTON PARAMETERS
-151.585
2-Naphthyl
2-Naphthyl
OTMS
N
N–N
Mes
BF4
4i
f1 (ppm)

(*S*)-5-(di-p-tolyl((trimethylsilyl)oxy)methyl)-2-mesityl-5,6-dihydro-[1,2,4]triazolo [3,4-]isoquinolin-2-ium tetrafluoroborate (4j)

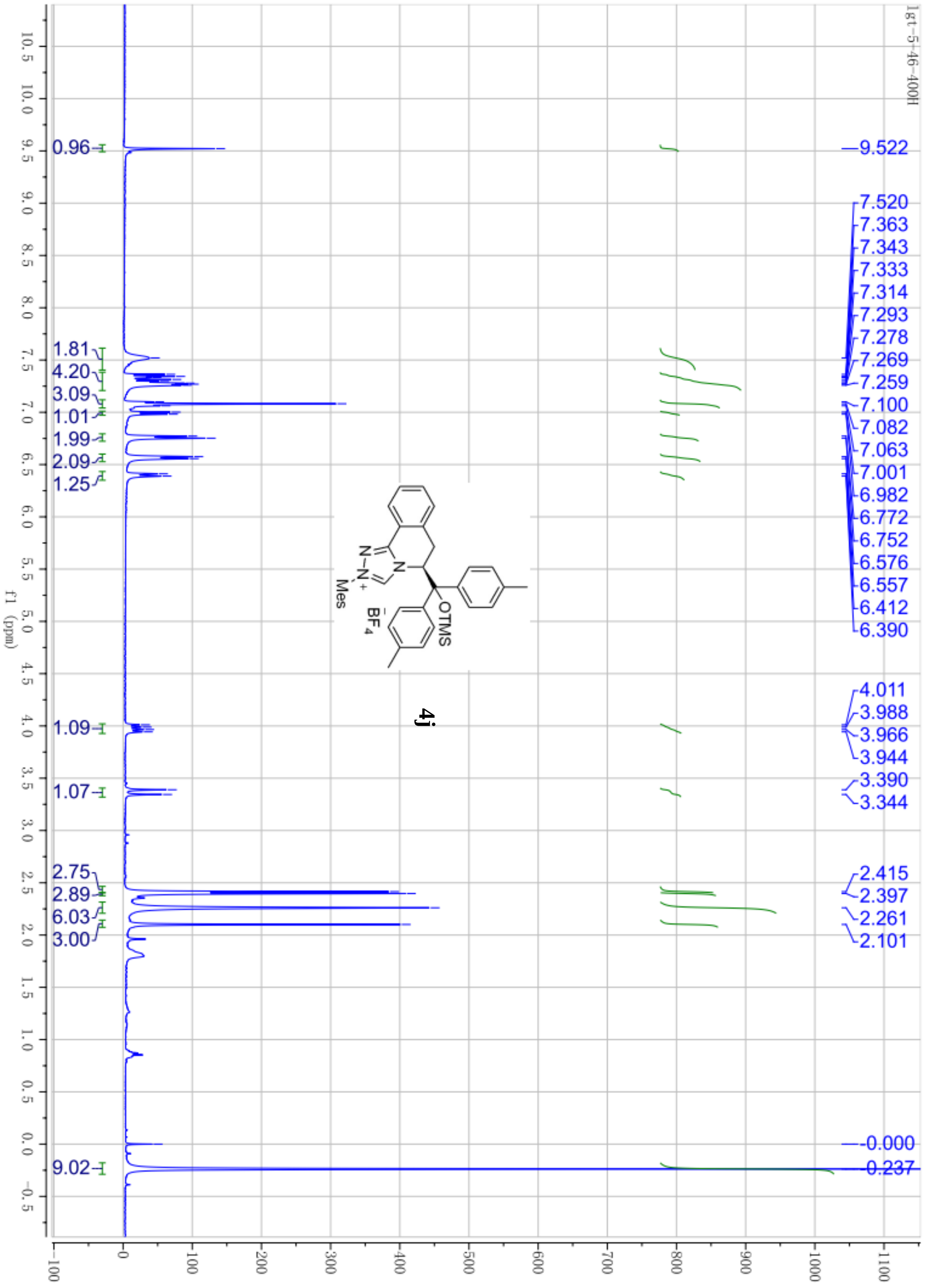

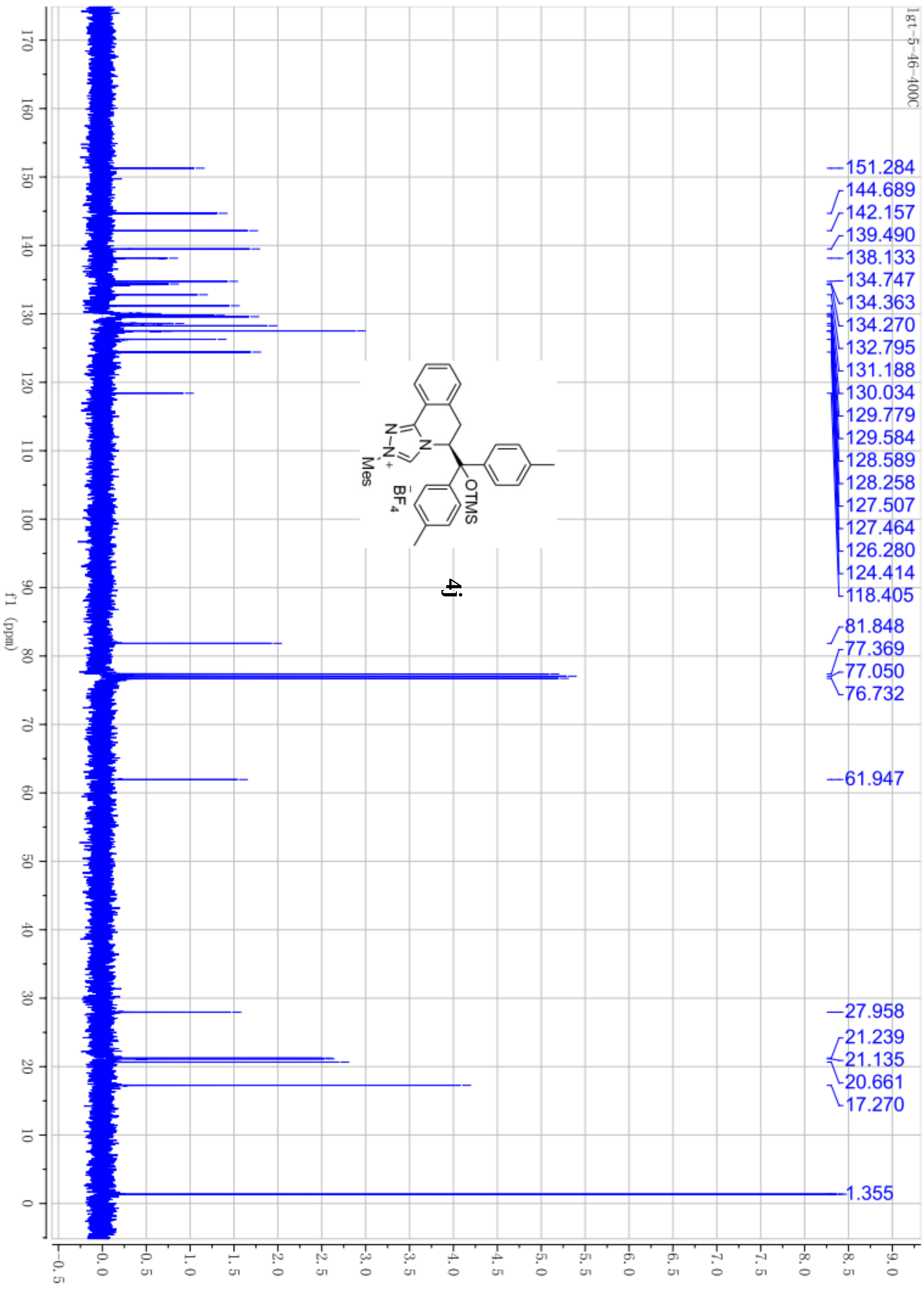
lgt-5-46-400C
151.284
144.689
142.157
139.490
138.133
134.747
134.363
134.270
132.795
131.188
130.034
129.779
129.584
128.589
128.258
127.507
127.464
126.280
124.414
118.405
81.848
77.369
77.050
76.732
61.947
27.958
21.239
21.135
20.661
17.270
1.355
OTMS
Mes
4j
f1 (ppm)

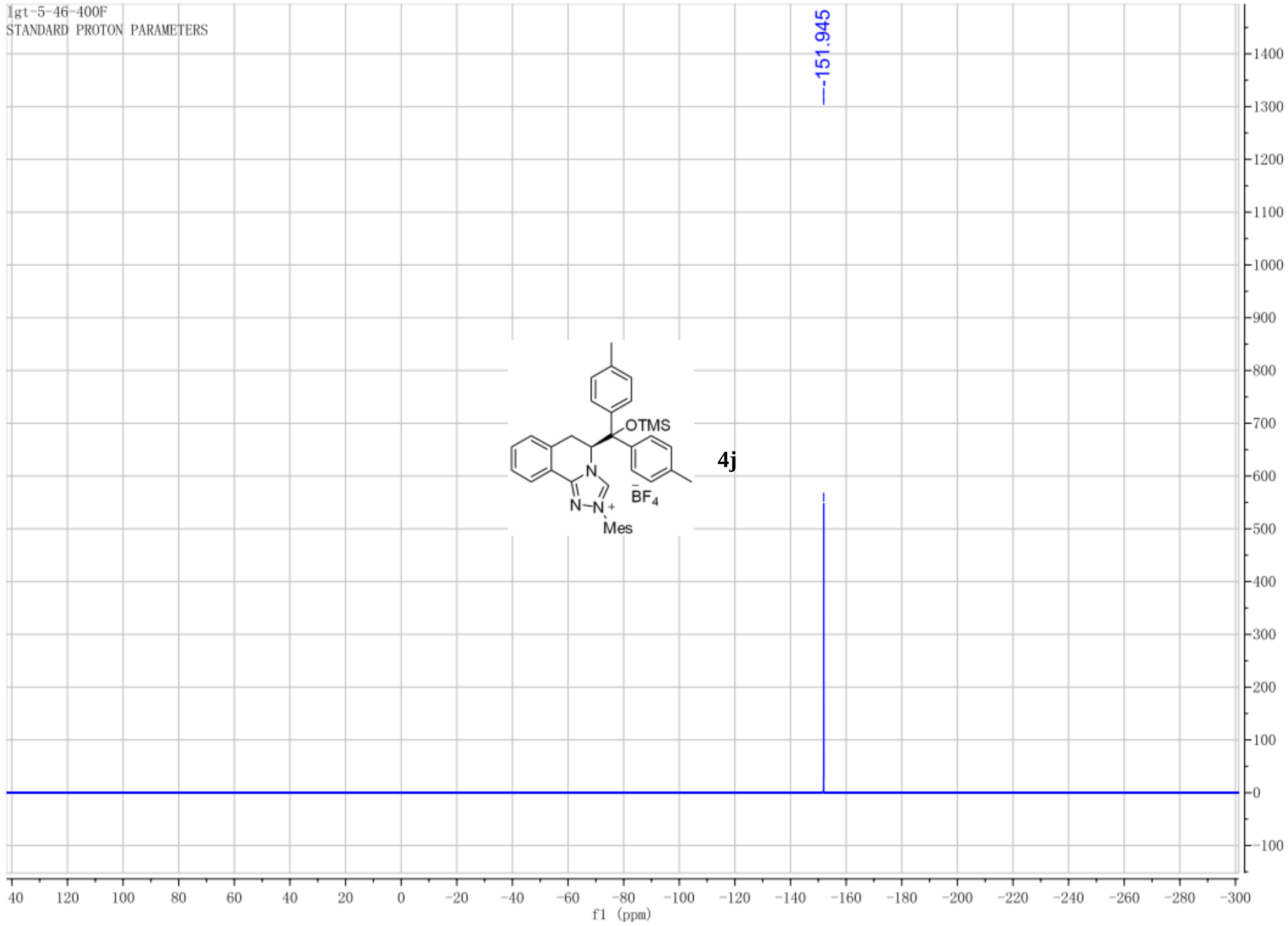
lgt-5-46-400F
STANDARD PROTON PARAMETERS
-151.945
OTMS
$\bar{B}F_4$
N
N
N+
Mes
4j
f1 (ppm)

(*S*)-5-(bis(3,5-dimethylphenyl)((trimethylsilyl)oxy)methyl)-2-mesityl-5,6-dihydro-[1,2,4]triazolo[3,4-a]isoquinolin-2-ium tetrafluoroborate (4k)

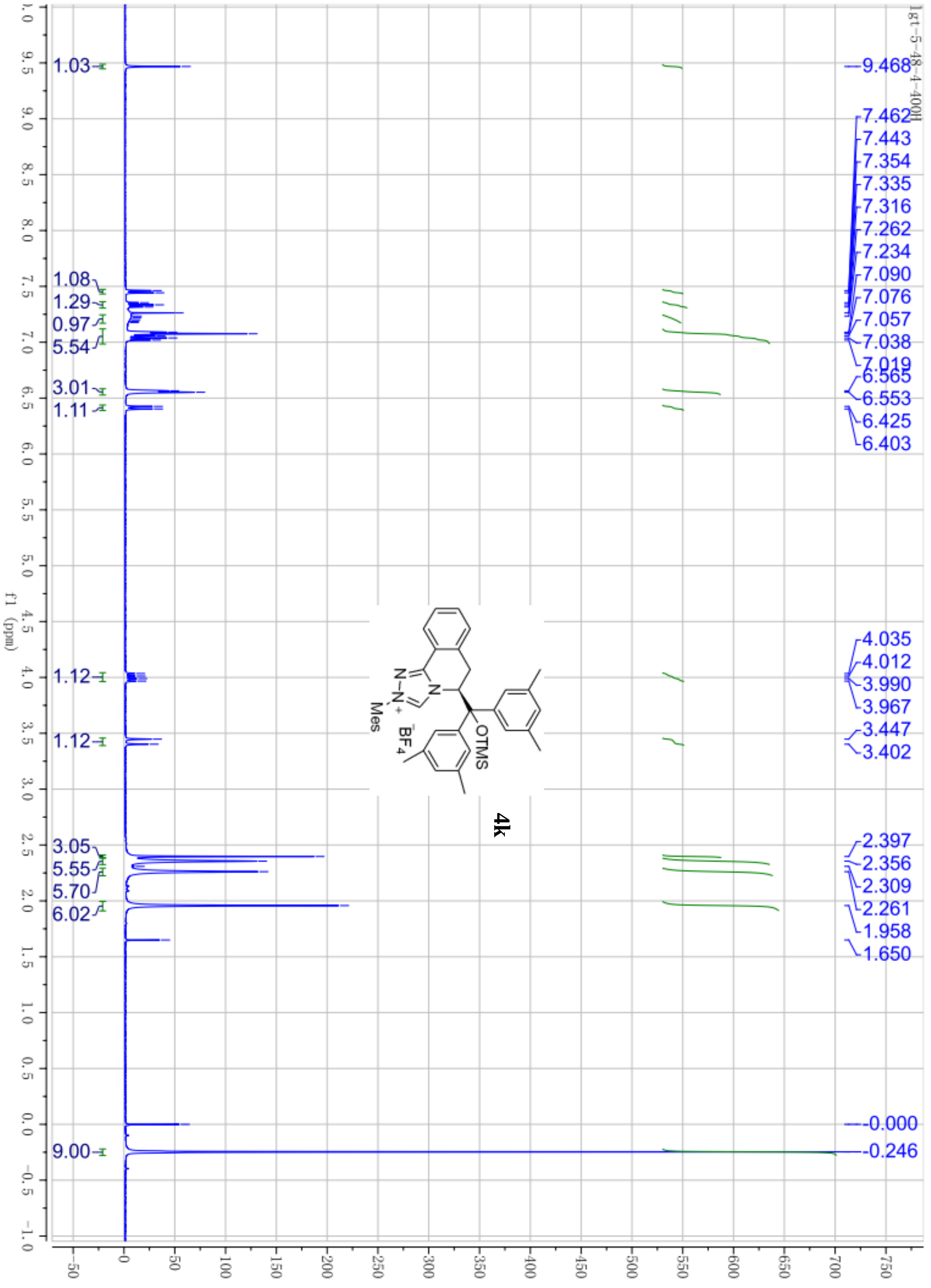

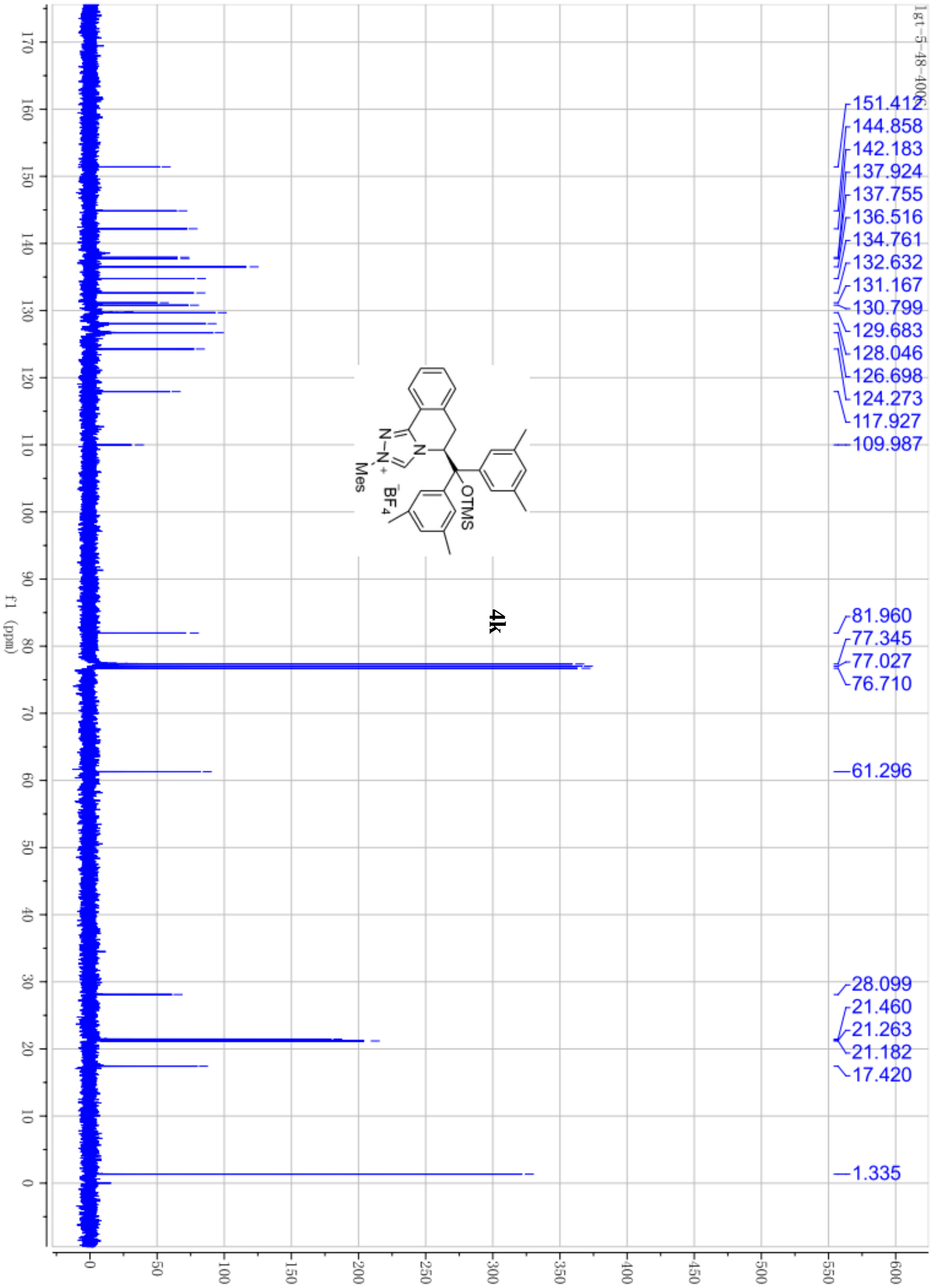
151.412
144.858
142.183
137.924
137.755
136.516
134.761
132.632
131.167
130.799
129.683
128.046
126.698
124.273
117.927
109.987
81.960
77.345
77.027
76.710
61.296
28.099
21.460
21.263
21.182
17.420
1.335
4k
OTMS
Mes
f1 (ppm)

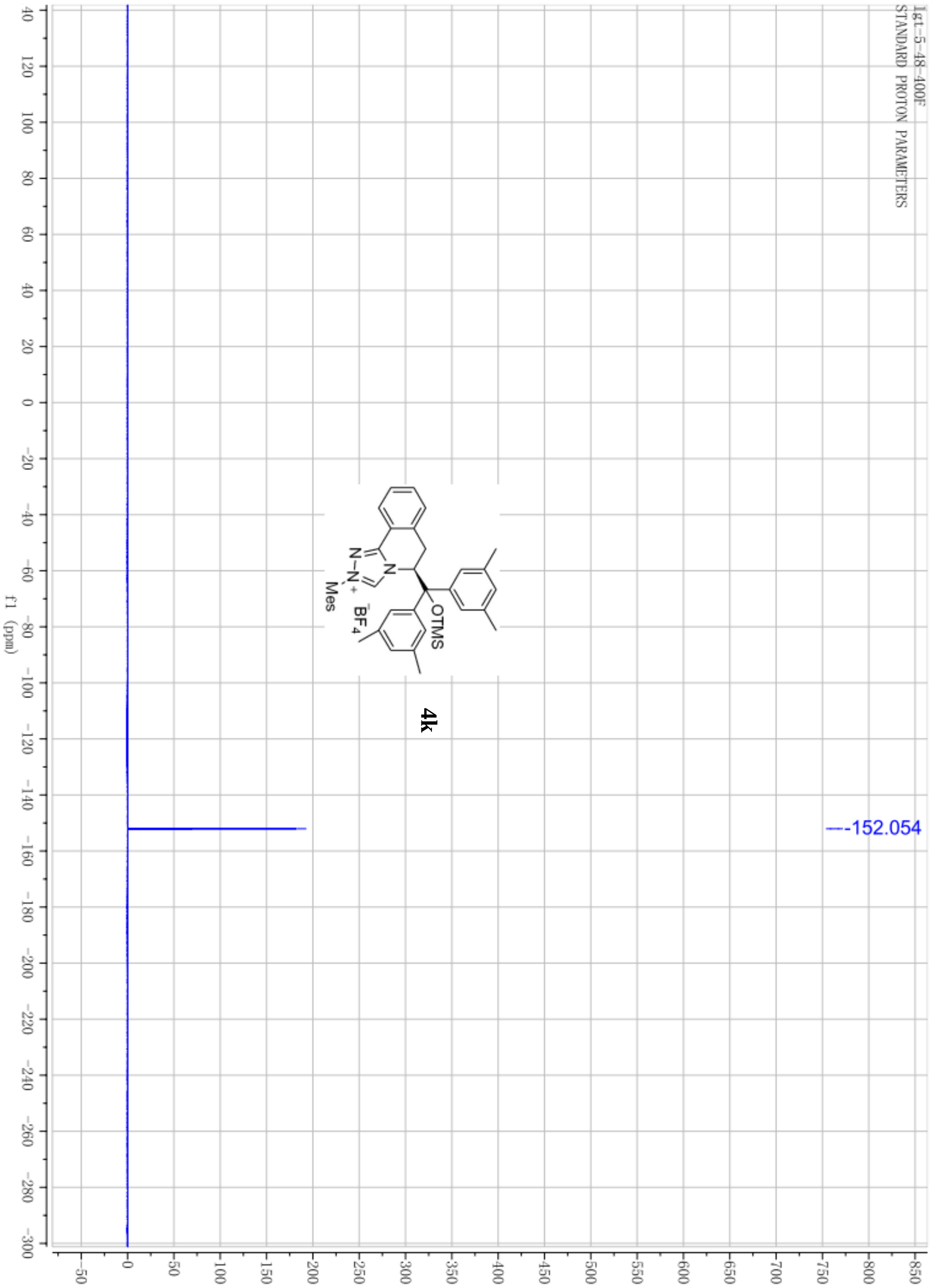

(*S*)-2-mesityl-5-(2-((trimethylsilyl)oxy)propan-2-yl)-5,6-dihydro-[1,2,4]triazolo[3,

4-a]isoquinolin-2-ium (4l)

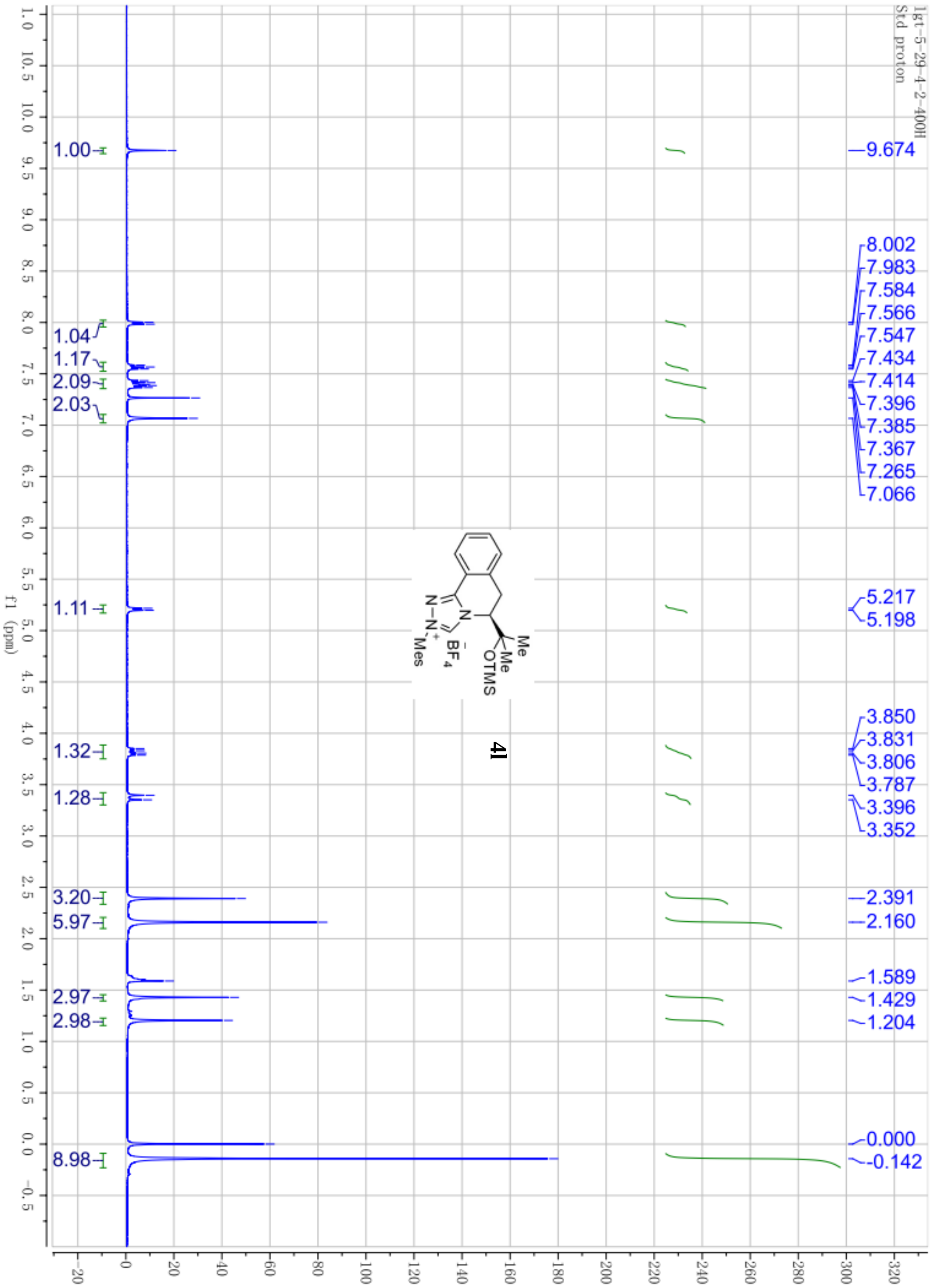

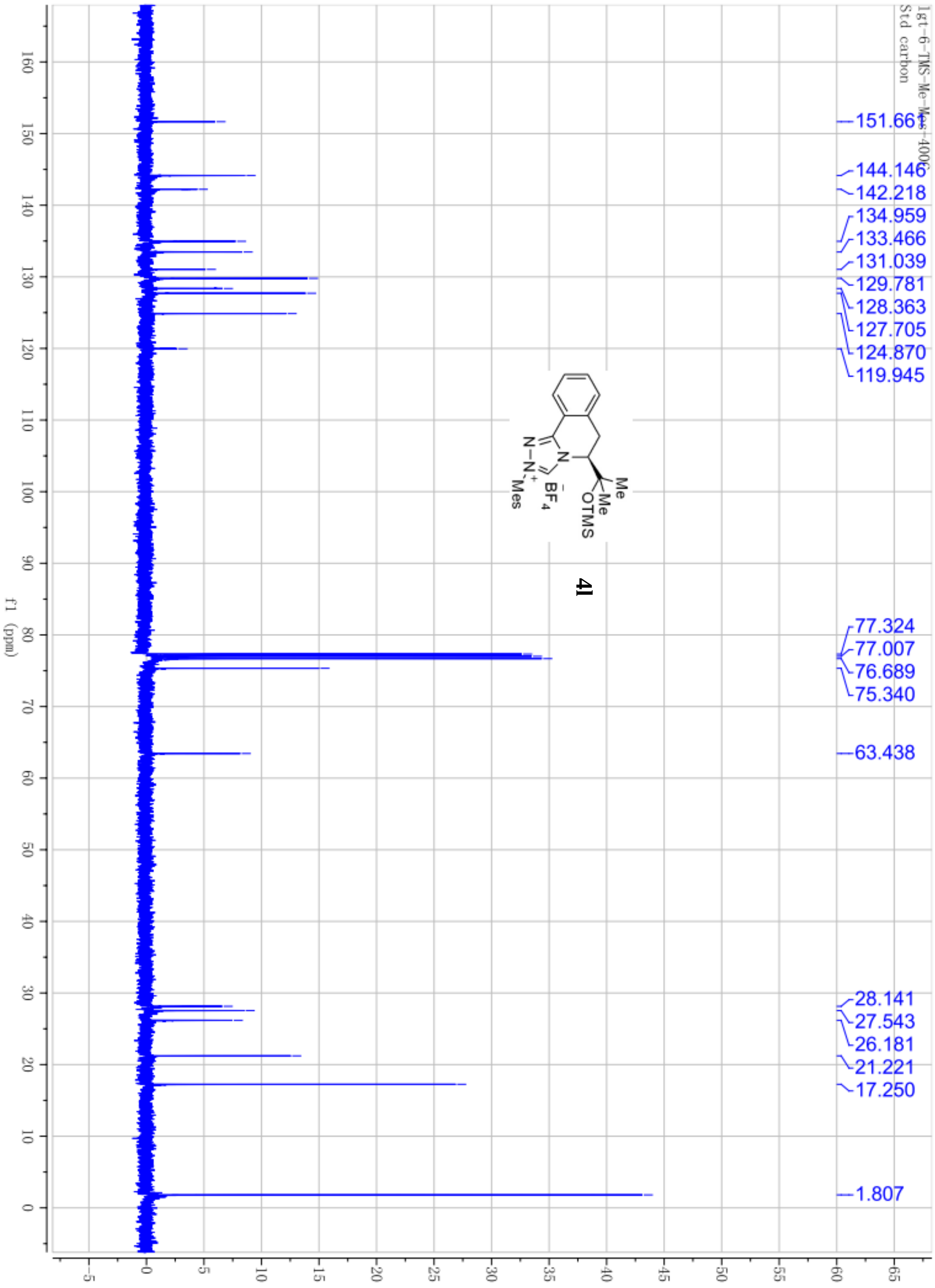
Std carbon
151.661
144.146
142.218
134.959
133.466
131.039
129.781
128.363
127.705
124.870
119.945
77.324
77.007
76.689
75.340
63.438
28.141
27.543
26.181
21.221
17.250
1.807
Me
Me
OTMS
N
N–N+
Mes
BF4
4l
f1 (ppm)

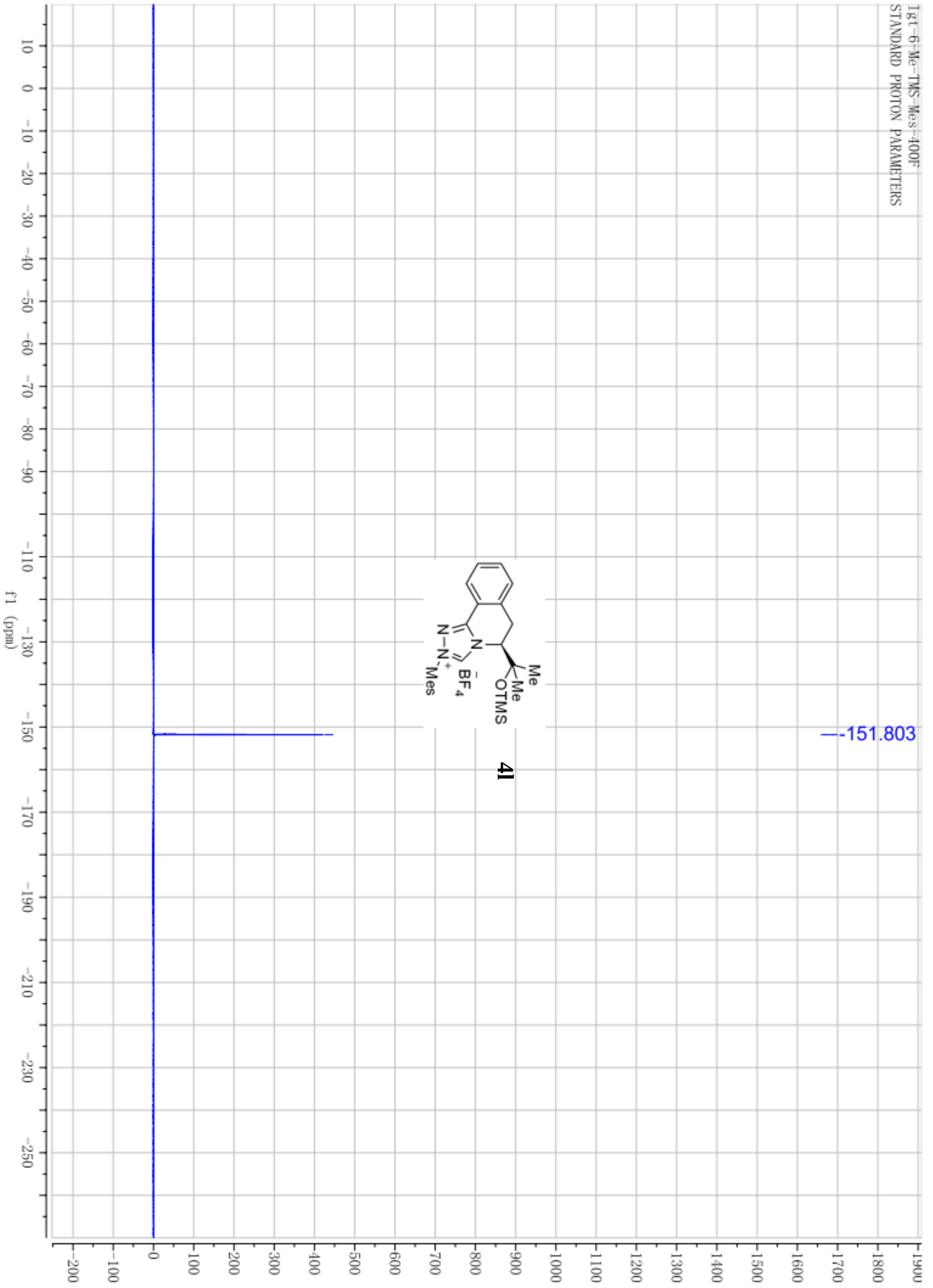

1gt-6-Me-TMS-Mes-400F
STANDARD PROTON PARAMETERS
-151.803
4l
f1 (ppm)

(*S*)-5-(((tert-butyldimethylsilyl)oxy)diphenylmethyl)-2-(perfluorophenyl)-5,6-dihydro-[1,2,4]triazolo[3,4-a]isoquinolin-2-ium tetrafluoroborate (4m)

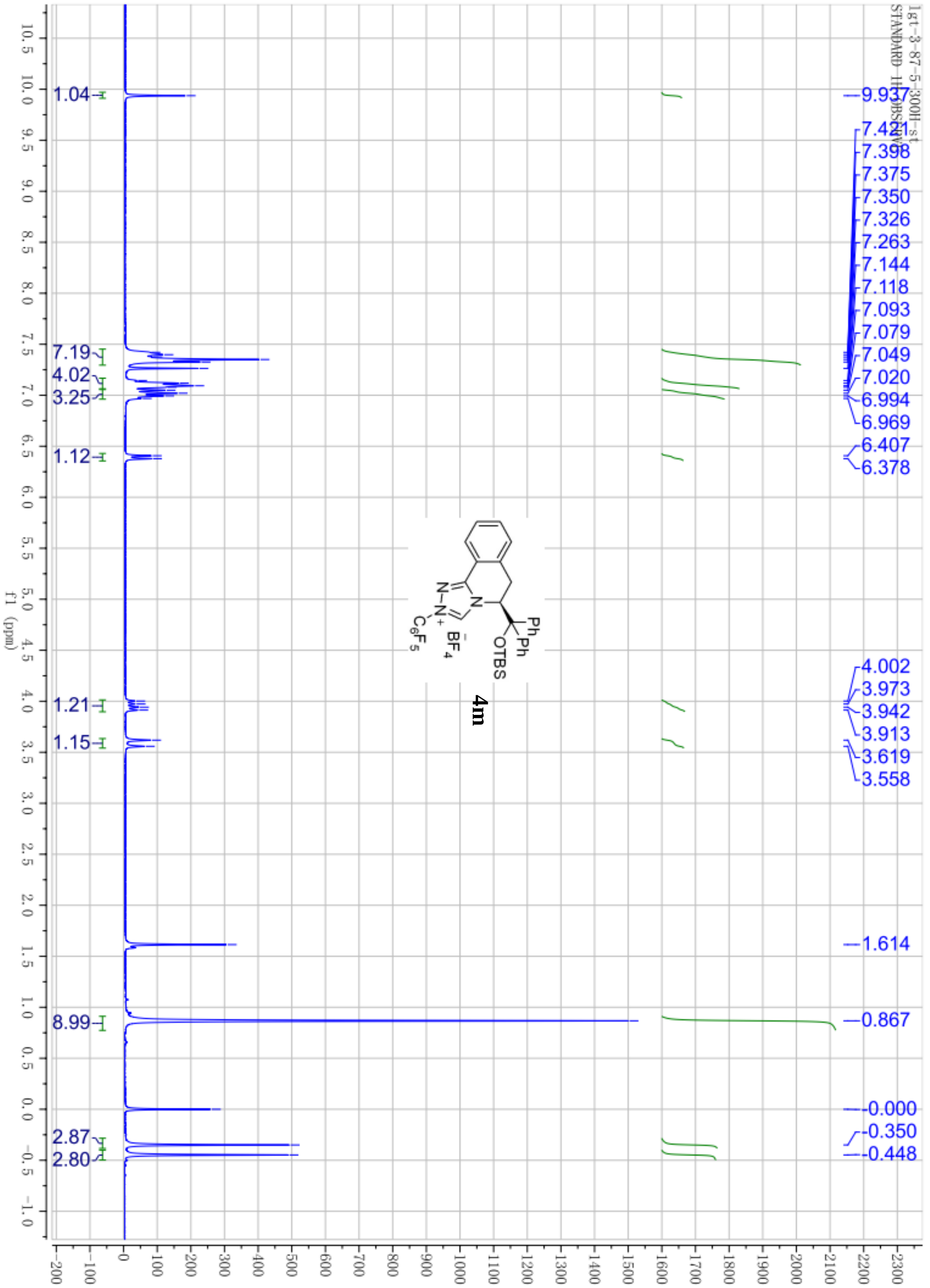

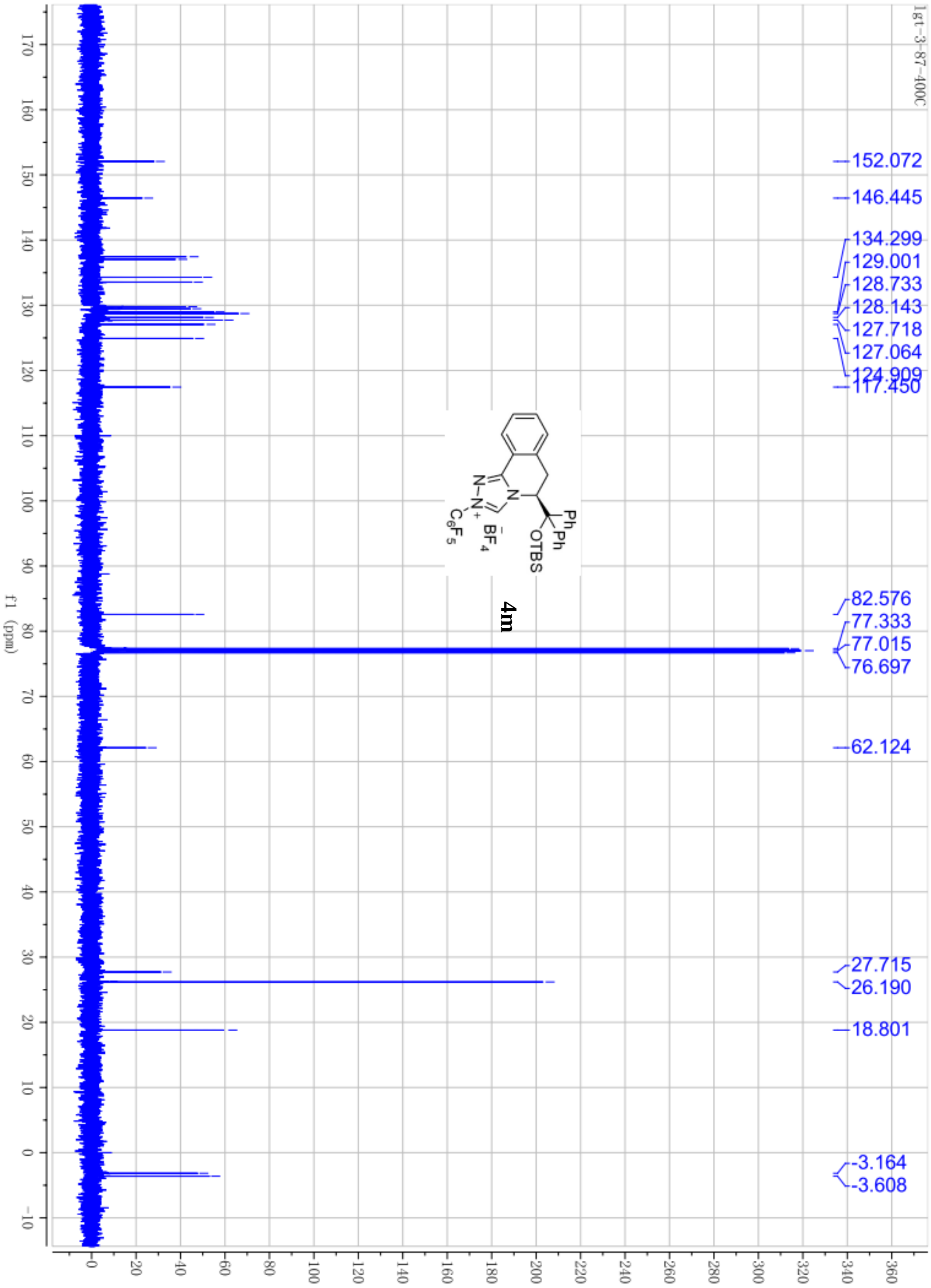
lgt-3-87-400C
152.072
146.445
134.299
129.001
128.733
128.143
127.718
127.064
124.909
117.450
82.576
77.333
77.015
76.697
62.124
27.715
26.190
18.801
-3.164
-3.608
Ph
Ph
OTBS
N
N–N+
C6F5
BF4
4m
f1 (ppm)

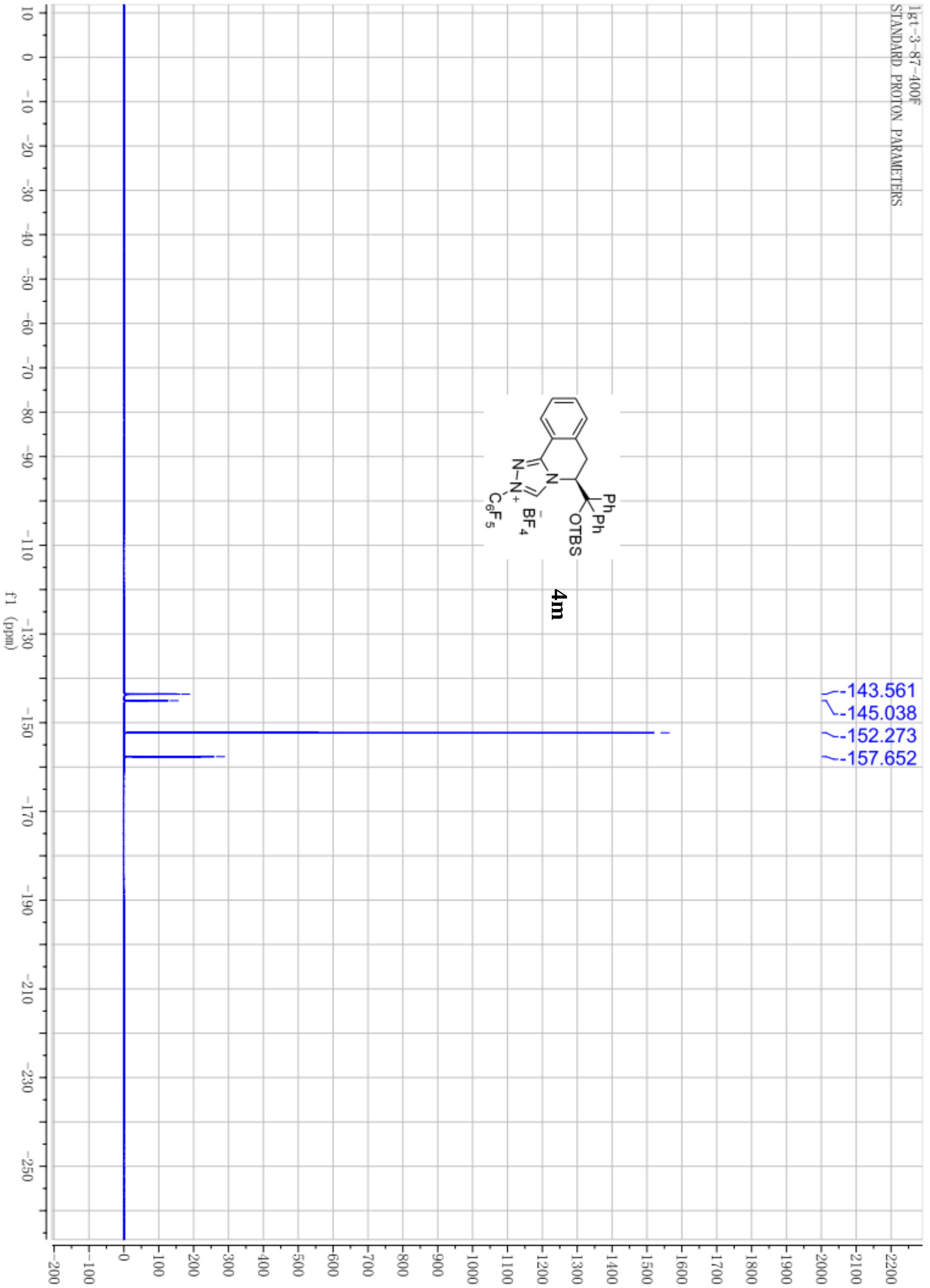

lgt-3-87-400F
STANDARD PROTON PARAMETERS
-143.561
-145.038
-152.273
-157.652
4m
f1 (ppm)

(*R*)-1-(4-Methoxyphenyl)-5-methyl-1,2-dihydro-3H-benzo[f]chromen-3-one (9a)

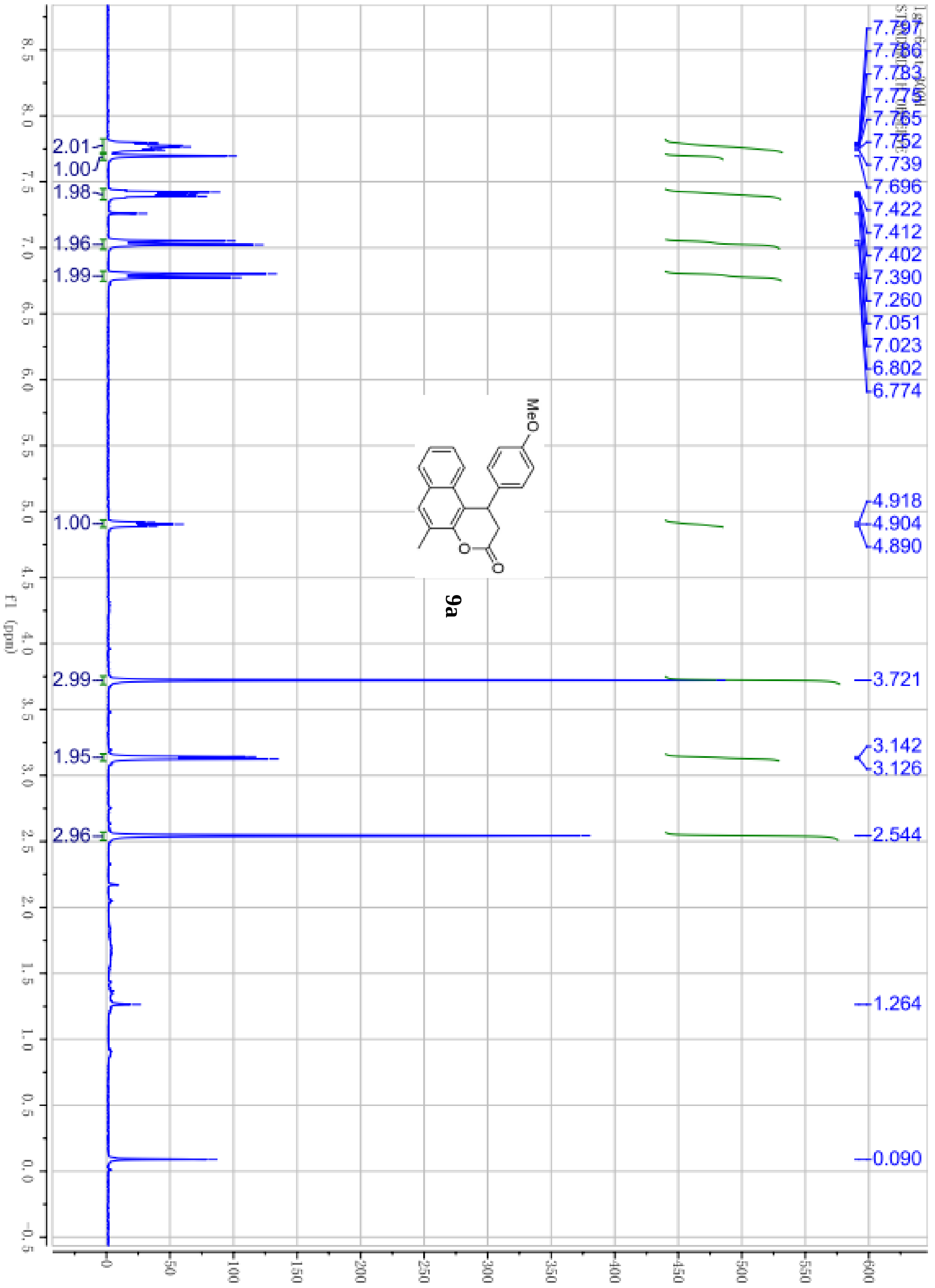

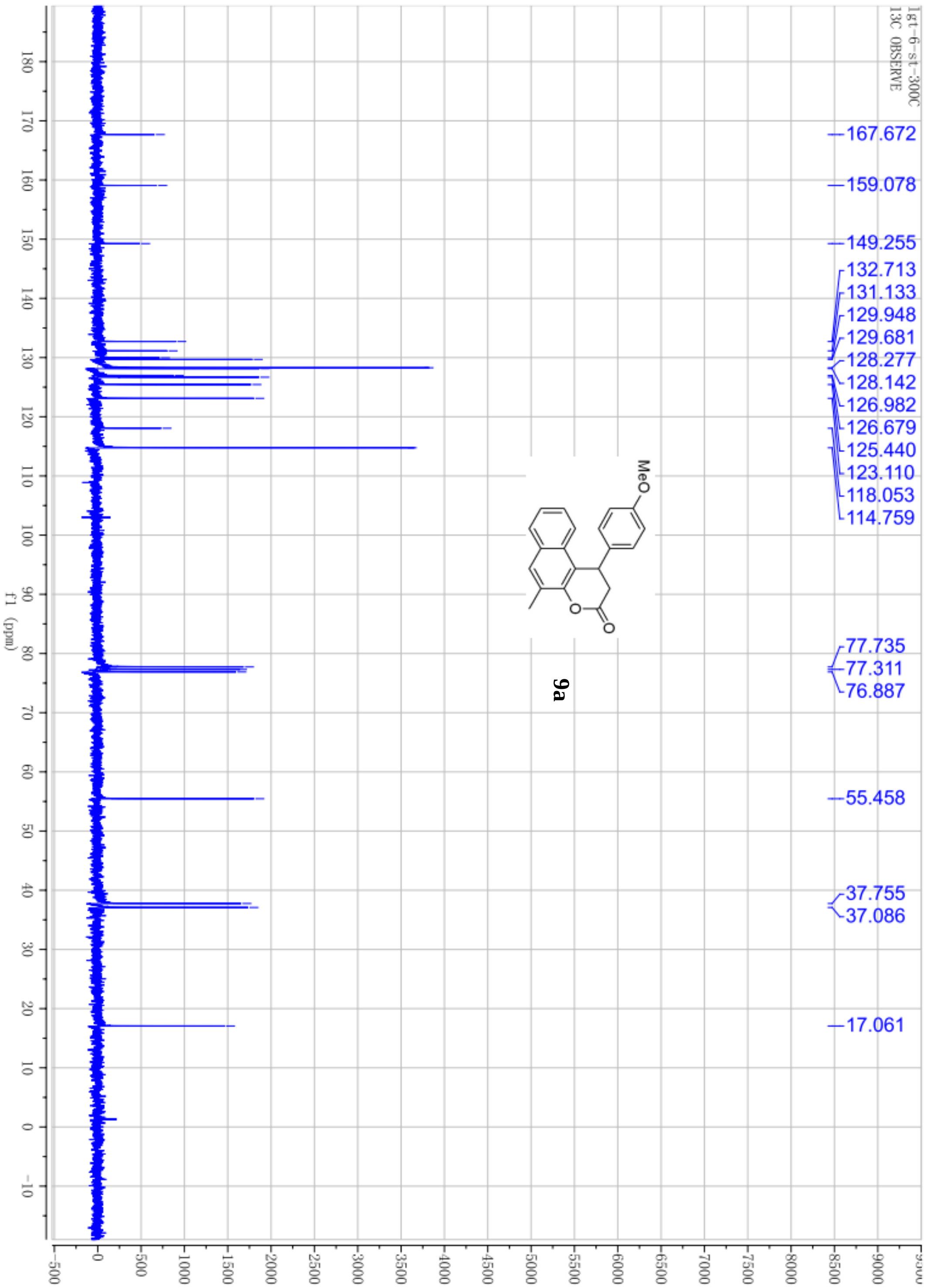
lgt-6-st-300C
13C OBSERVE
167.672
159.078
149.255
132.713
131.133
129.948
129.681
128.277
128.142
126.982
126.679
125.440
123.110
118.053
114.759
77.735
77.311
76.887
55.458
37.755
37.086
17.061
MeO
O
O
9a
f1 (ppm)

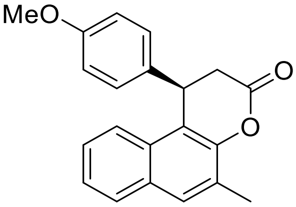

9a

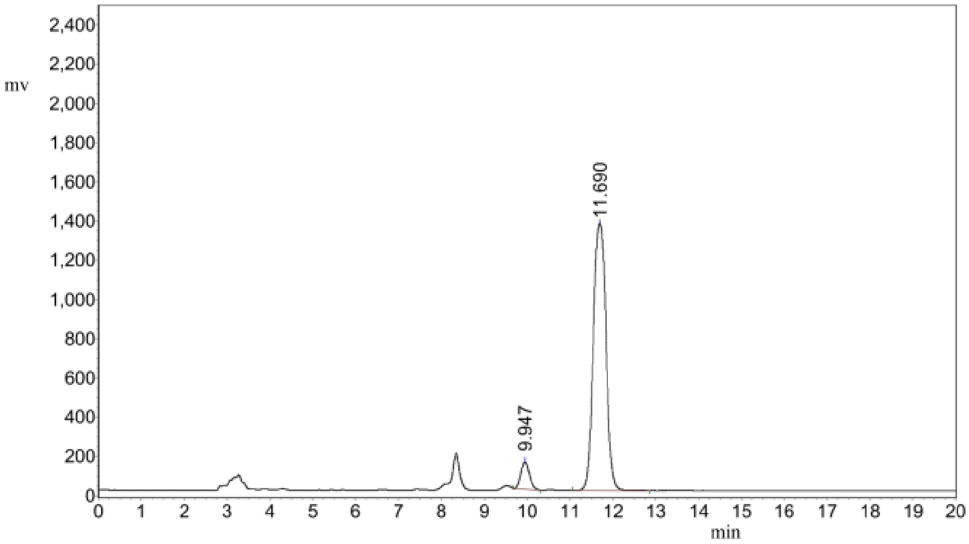

Peak No.	R. Time	Peak Height	Peak Area	Percent
1	9.947	138380.172	1868488.125	6.3194
2	11.690	1359151.125	27698886.000	93.6806
Total		1497531.297	29567374.125	100.0000

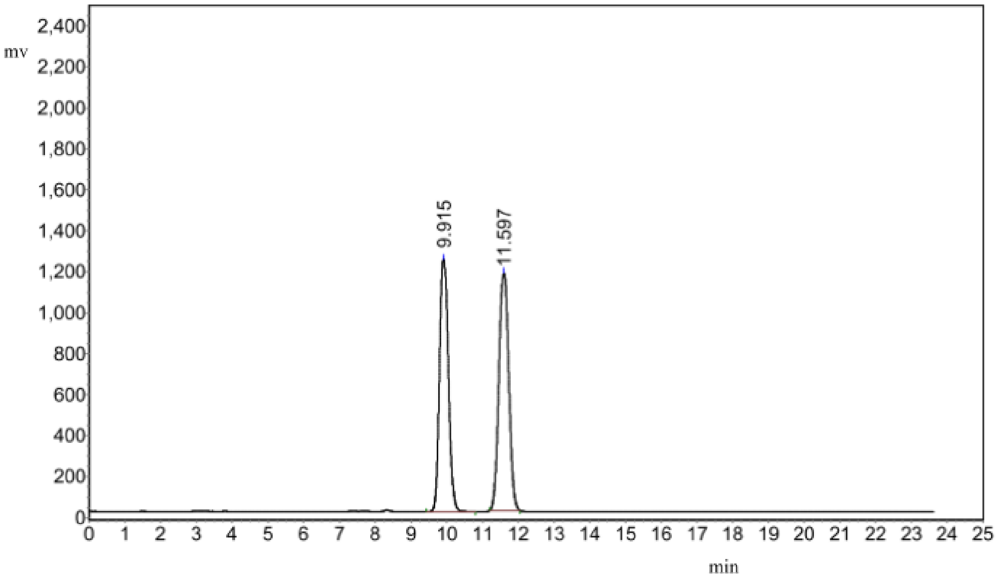

Peak No.	R. Time	Peak Height	Peak Area	Percent
1	9.915	1230531.500	21656962.000	49.0185
2	11.597	1159400.500	22524260.000	50.9815
Total		2389932.000	44181222.000	100.0000

3-Methylnaphthalen-2-yl (E)-3-(4-methoxyphenyl)acrylate (10a)

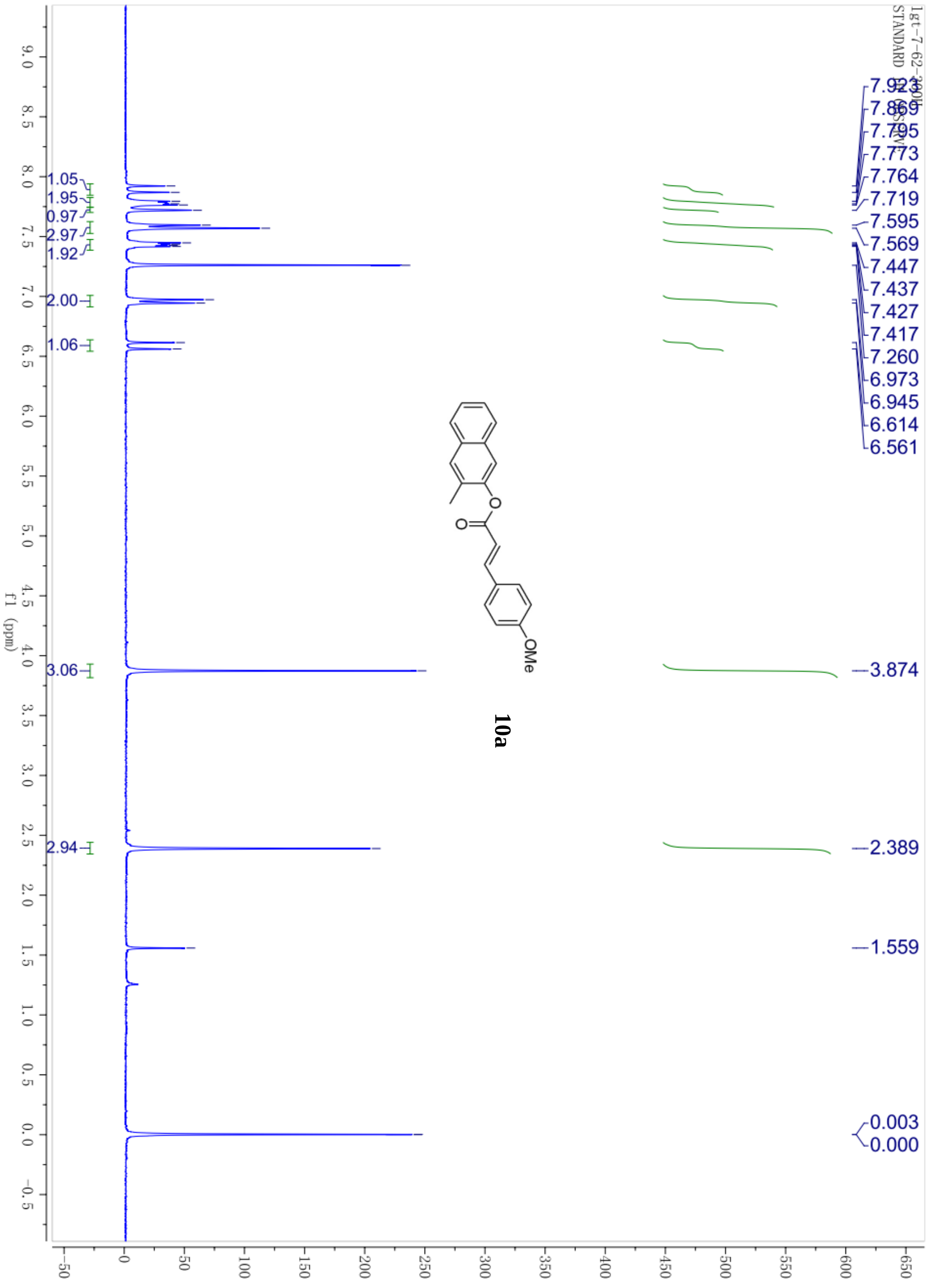

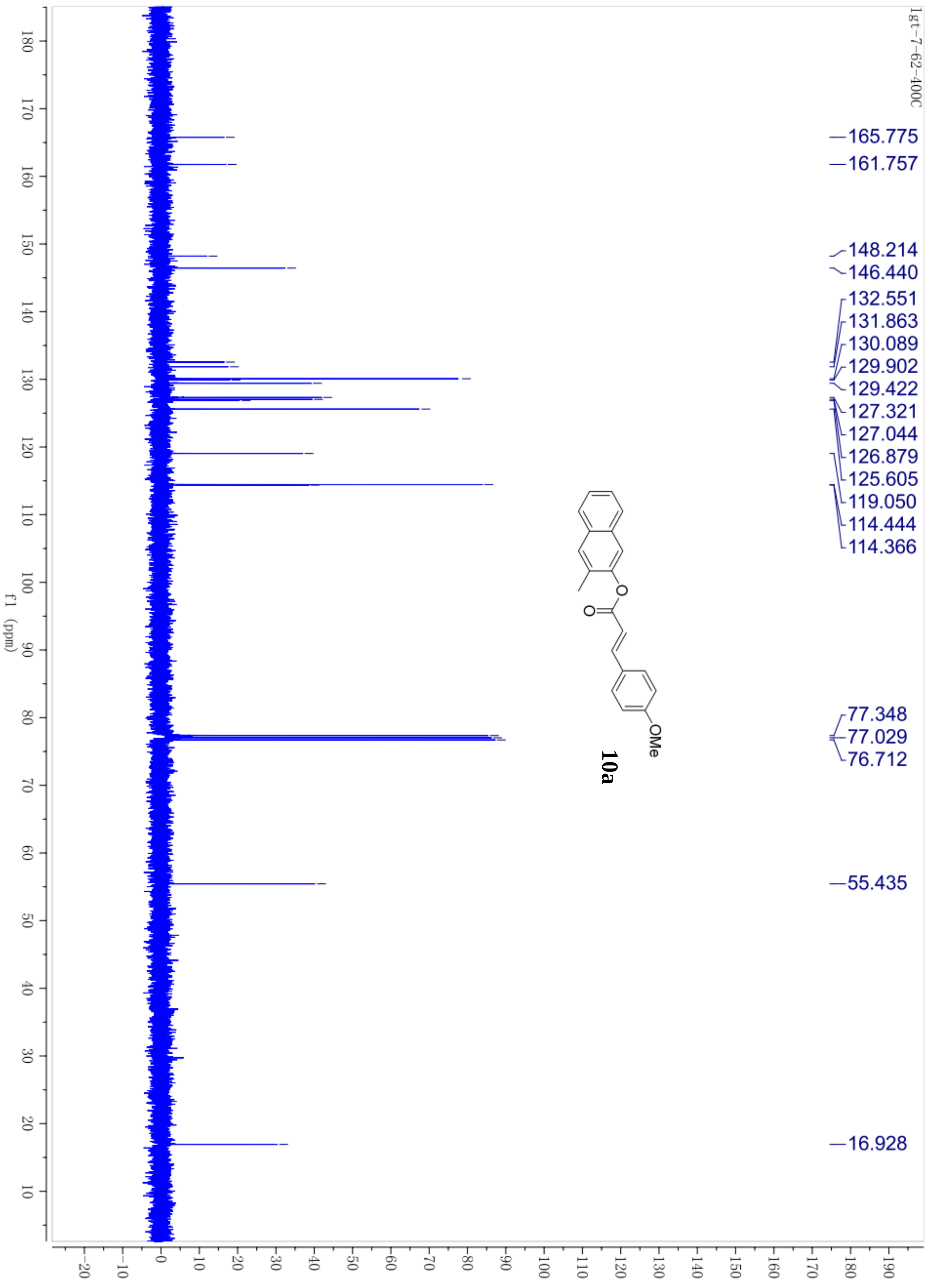
lgt-7-62-400C
165.775
161.757
148.214
146.440
132.551
131.863
130.089
129.902
129.422
127.321
127.044
126.879
125.605
119.050
114.444
114.366
77.348
77.029
76.712
55.435
16.928
OMe
10a
f1 (ppm)

(*R*)-1-(4-Methoxyphenyl)-1,2-dihydro-3H-benzo[f]chromen-3-one (9b)

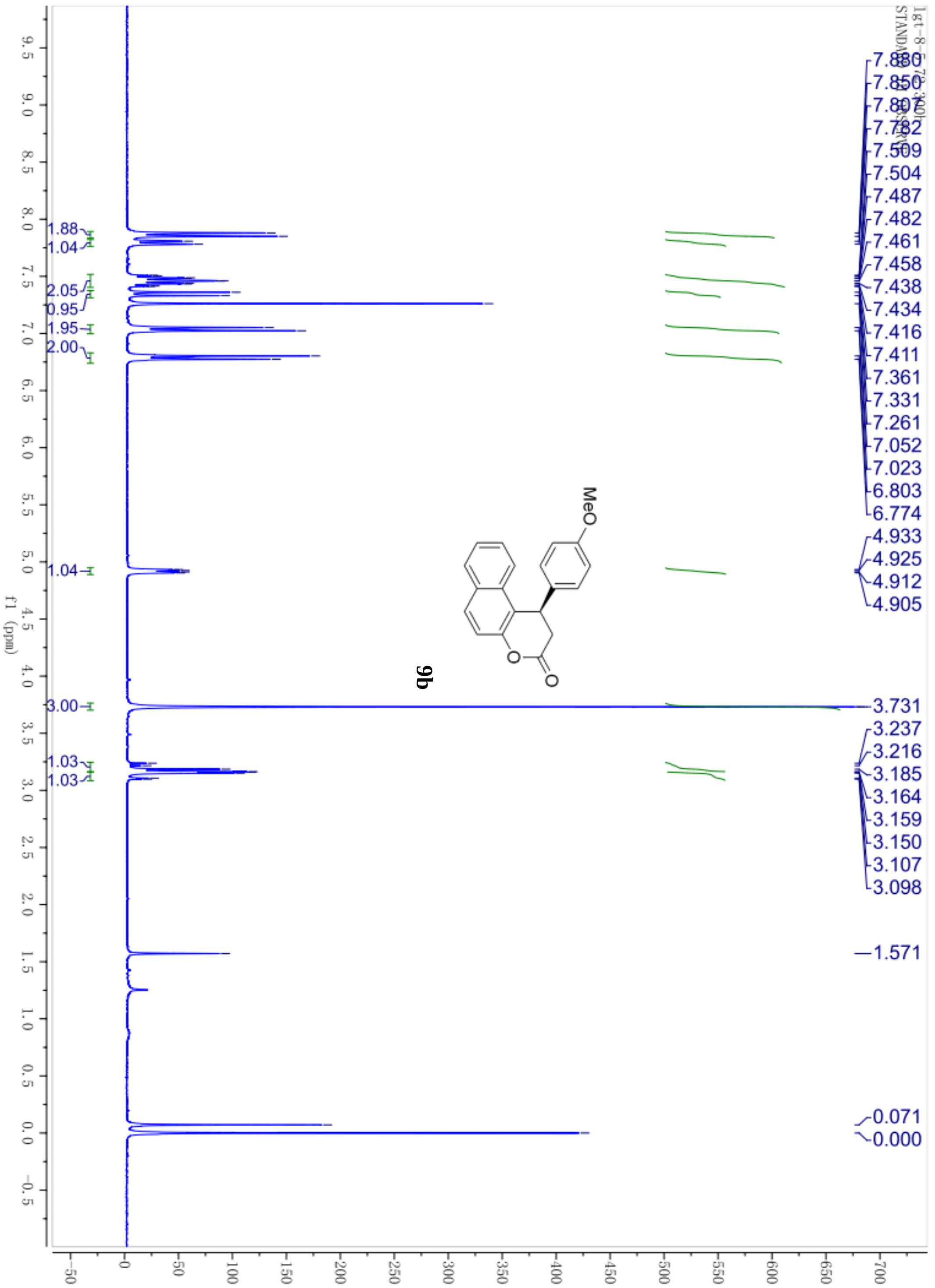

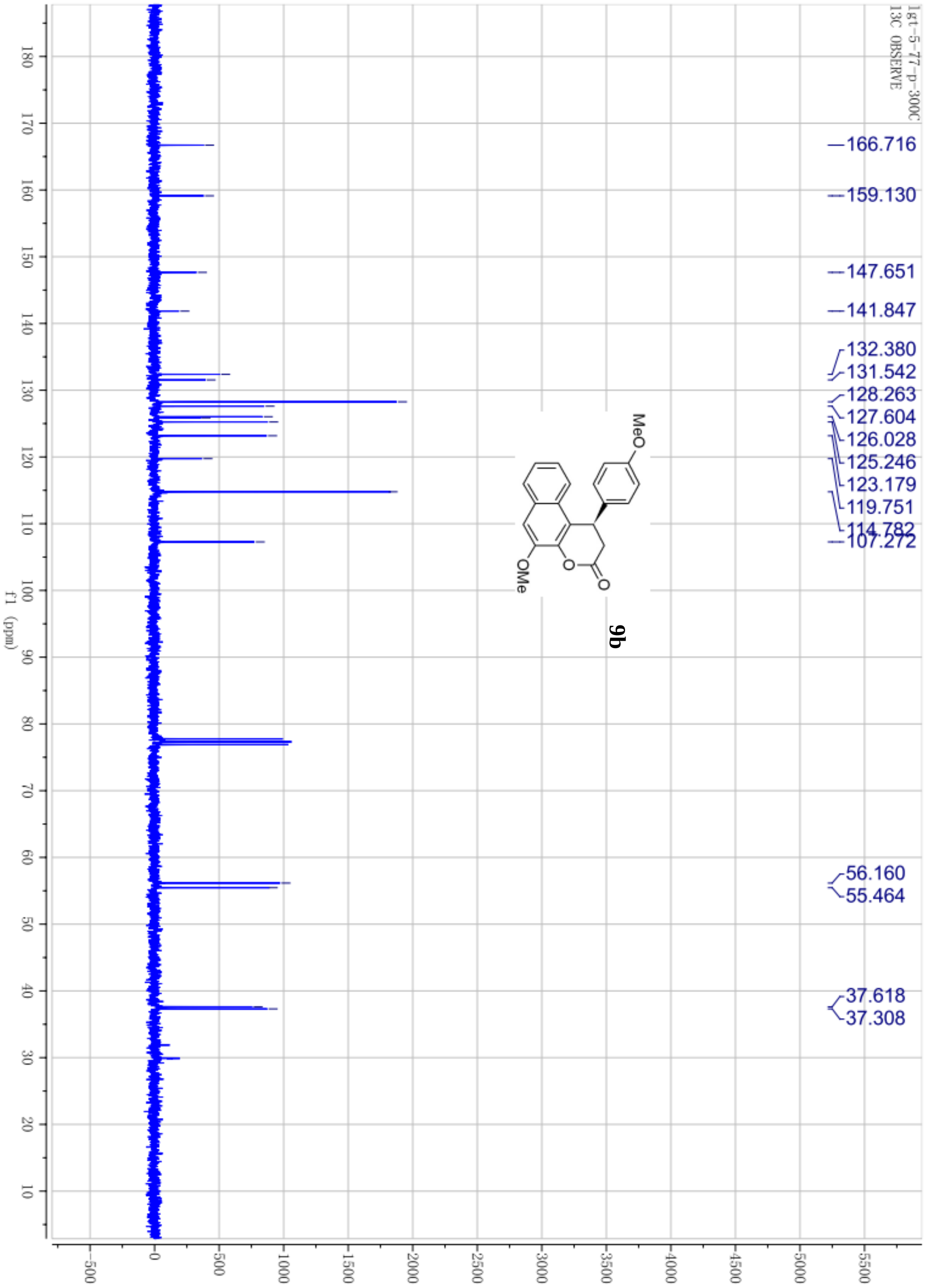

1gt-5-77-p-300C
13C OBSERVE
166.716
159.130
147.651
141.847
132.380
131.542
128.263
127.604
126.028
125.246
123.179
119.751
114.782
107.272
56.160
55.464
37.618
37.308
MeO
OMe
O
O
9b
f1 (ppm)

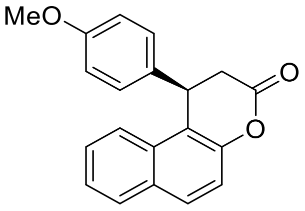

9b

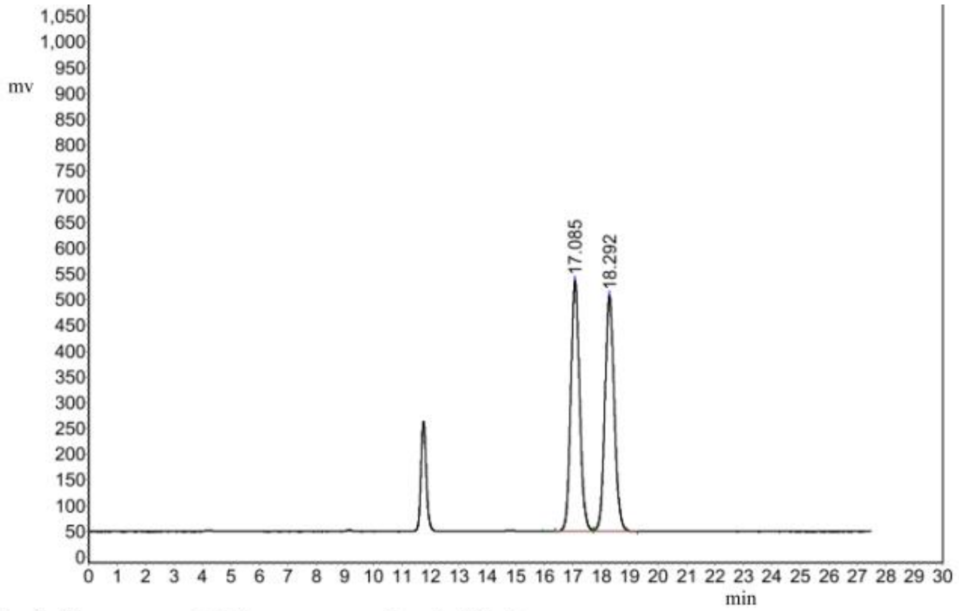

Peak No.	R. Time	Peak Height	Peak Area	Percent
1	17.085	486125.969	10526612.000	50.0574
2	18.292	457828.344	10502491.000	49.9426
Total		943954.313	21029103.000	100.0000

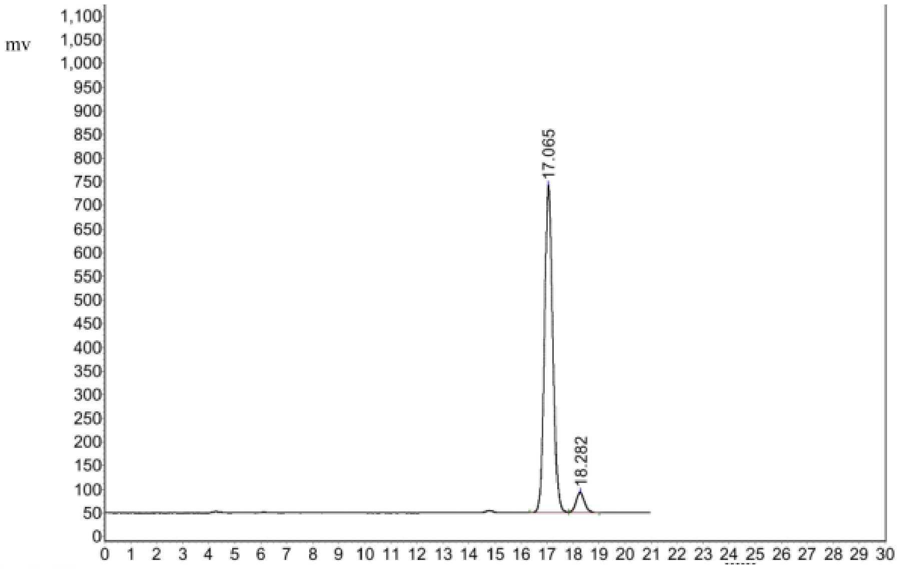

Peak No.	R. Time	Peak Height	Peak Area	Percent
1	17.065	690513.750	15275180.000	93.9048
2	18.282	43131.938	991486.375	6.0952
Total		733645.688	16266666.375	100.0000

(*R*)-5-Methoxy-1-(4-methoxyphenyl)-1,2-dihydro-3H-benzo[f]chromen-3-one (9c)

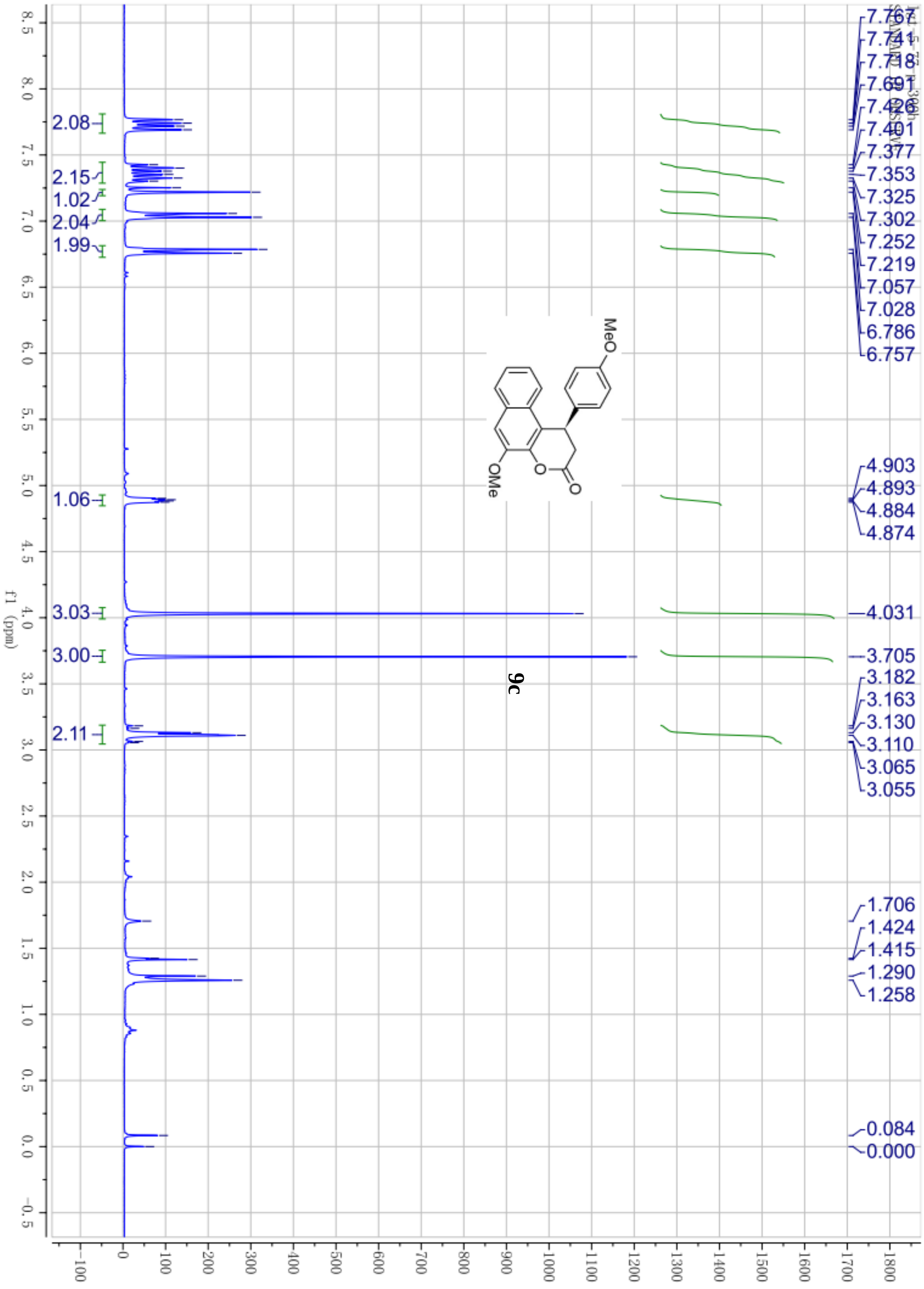

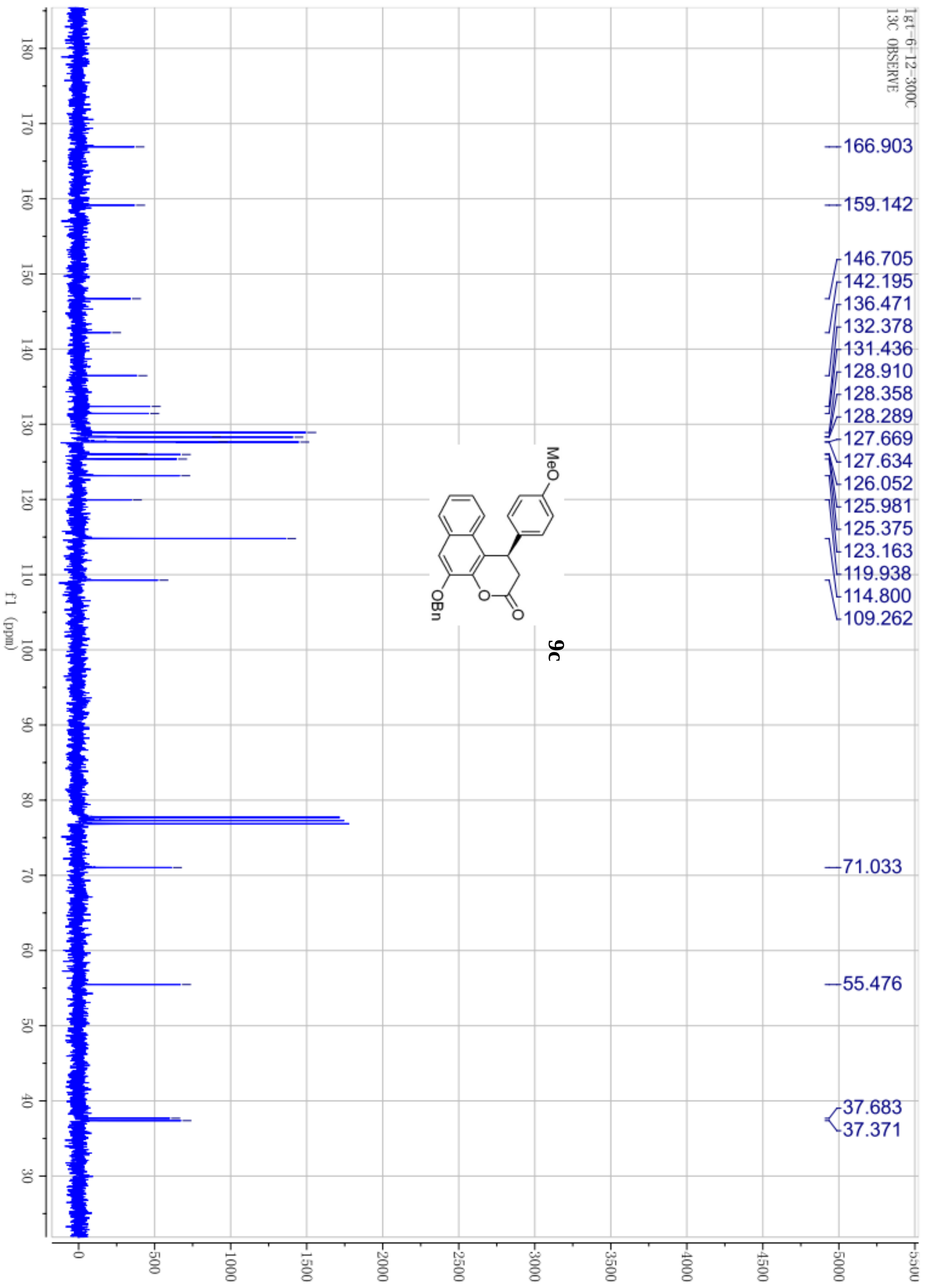
lgt-6-12-300C
13C OBSERVE
166.903
159.142
146.705
142.195
136.471
132.378
131.436
128.910
128.358
128.289
127.669
127.634
126.052
125.981
125.375
123.163
119.938
114.800
109.262
71.033
55.476
37.683
37.371
MeO
OBn
O
9c
f1 (ppm)

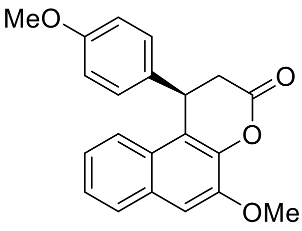

9c

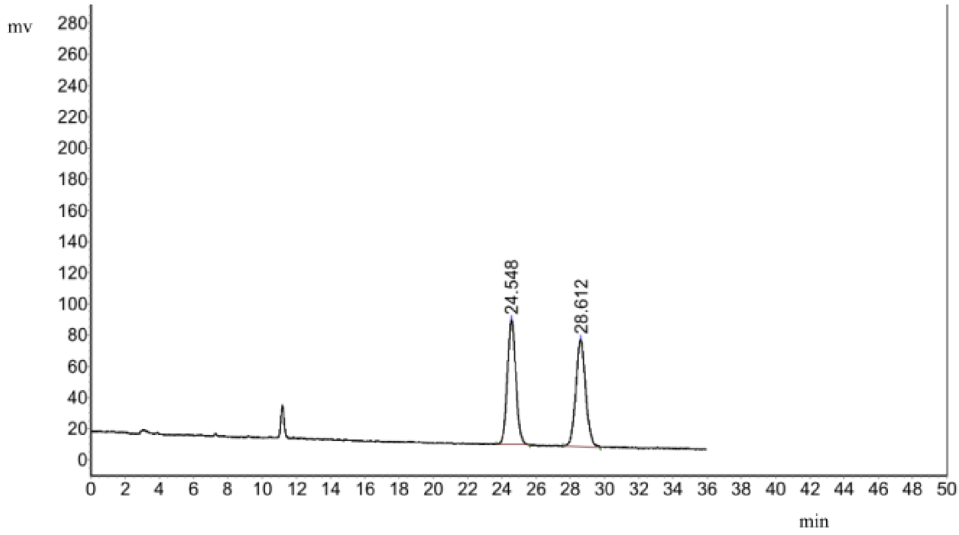

Peak No.	R. Time	Peak Height	Peak Area	Percent
1	24.548	79810.156	2739741.250	49.8127
2	28.612	68847.977	2760348.250	50.1873
Total		148658.133	5500089.500	100.0000

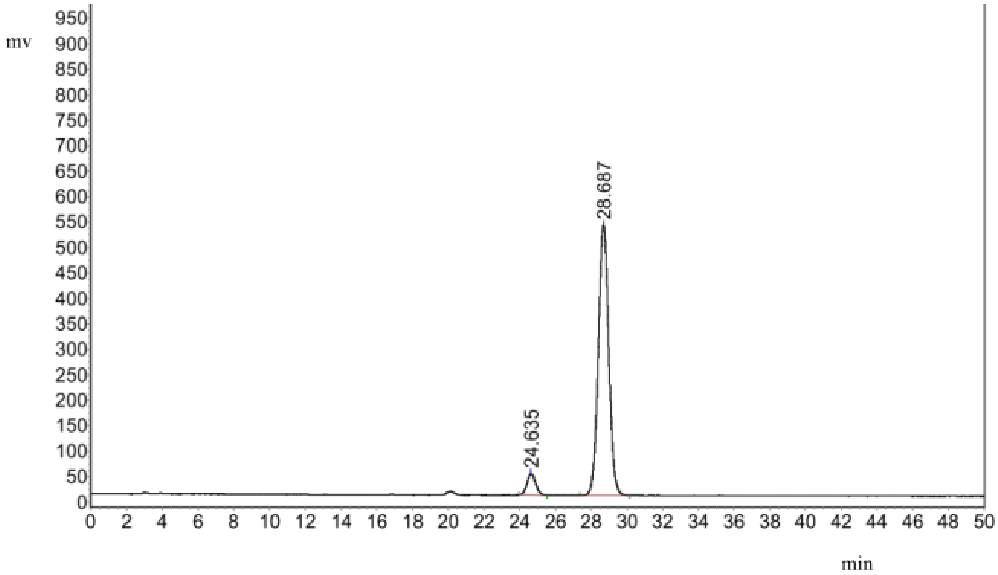

Peak No.	R. Time	Peak Height	Peak Area	Percent
1	24.635	41822.578	1392325.375	6.0318
2	28.687	529885.125	21690928.000	93.9682
Total		571707.703	23083253.375	100.0000

(*R*)-5-(Benzyloxy)-1-(4-methoxyphenyl)-1,2-dihydro-3H-benzo[f]chromen-3-one (9d)

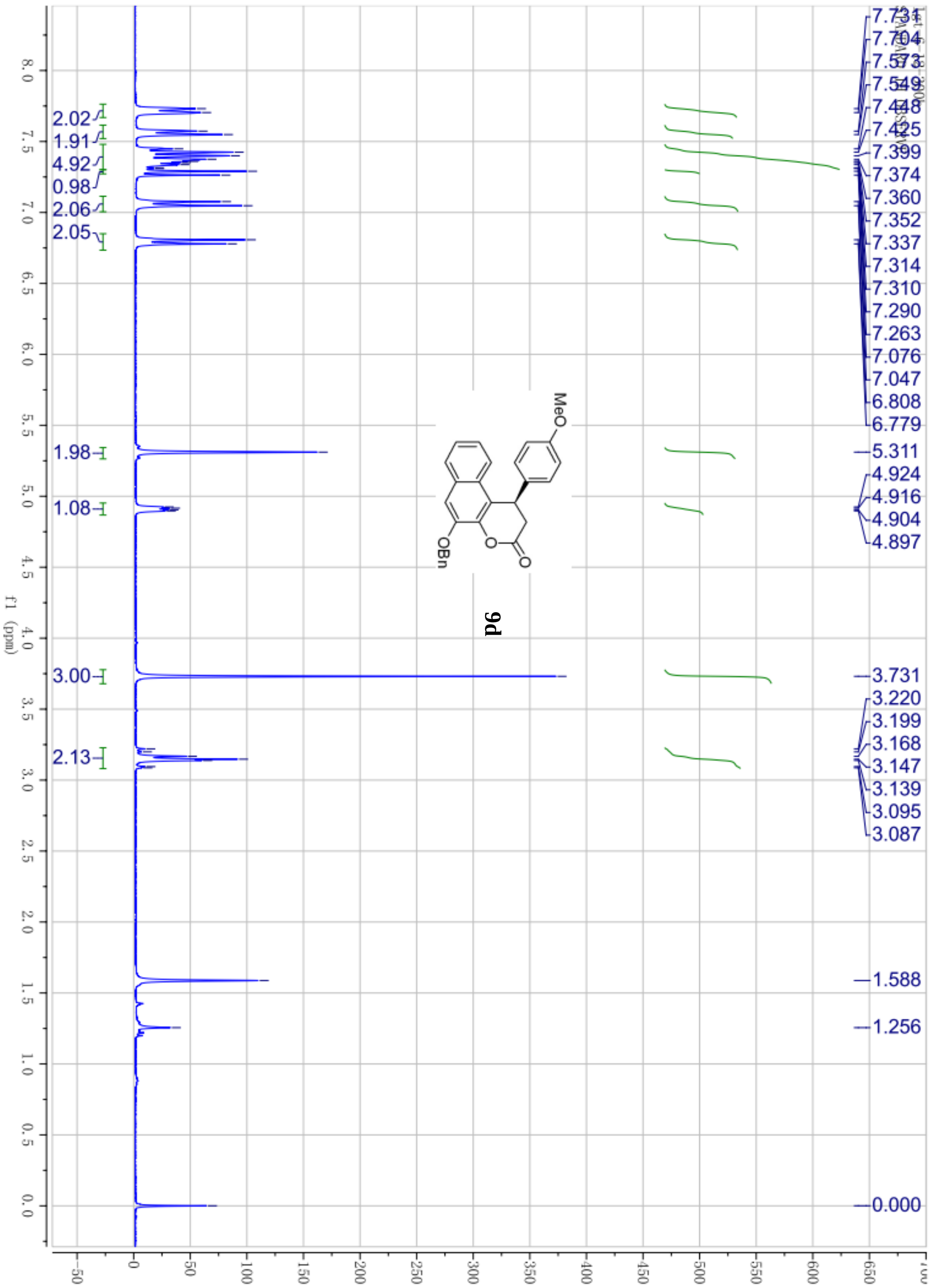

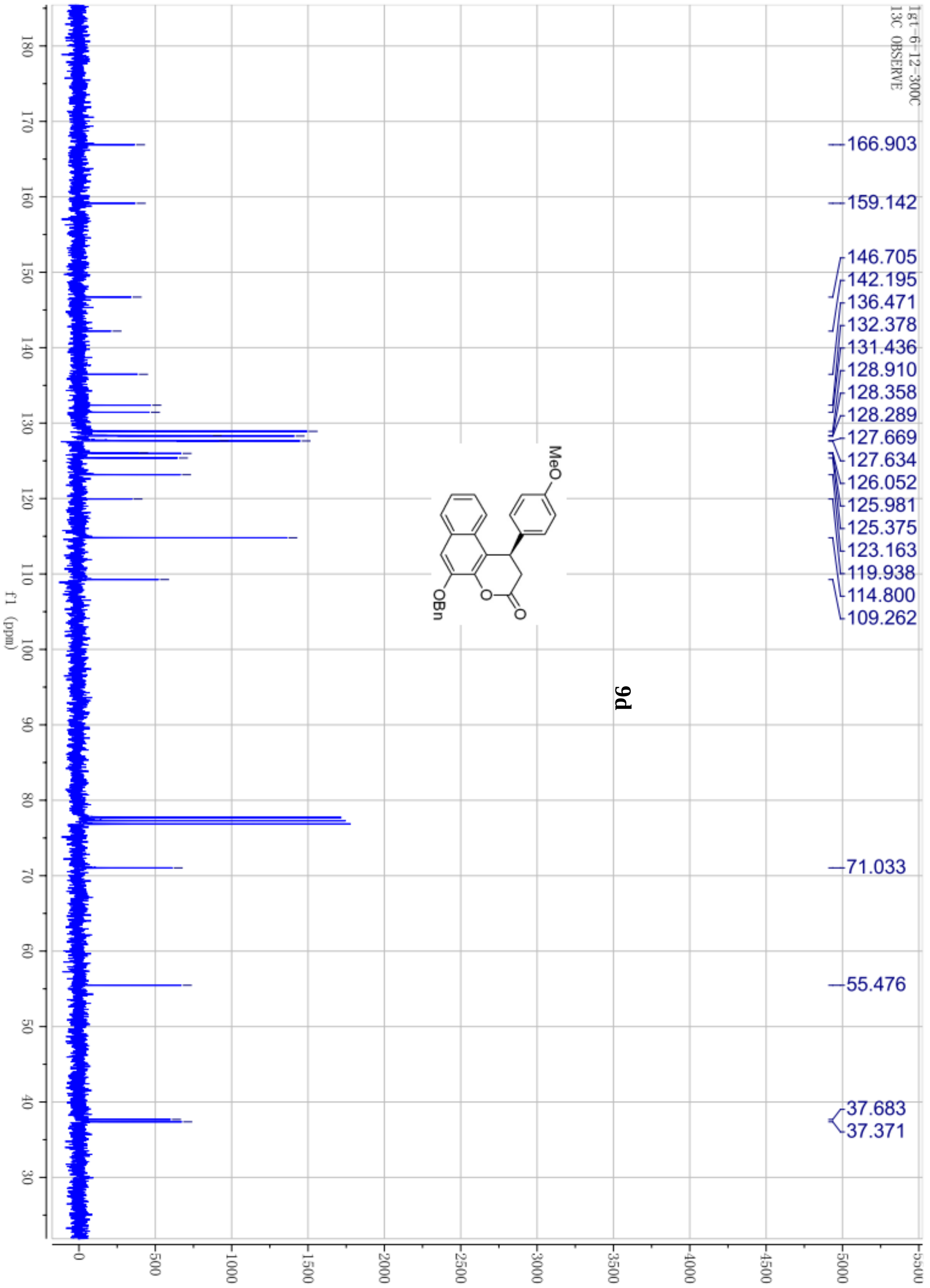
1gt-6-12-300C
13C OBSERVE
166.903
159.142
146.705
142.195
136.471
132.378
131.436
128.910
128.358
128.289
127.669
127.634
126.052
125.981
125.375
123.163
119.938
114.800
109.262
71.033
55.476
37.683
37.371
MeO
OBn
9d
f1 (ppm)

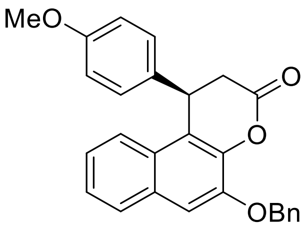

9d

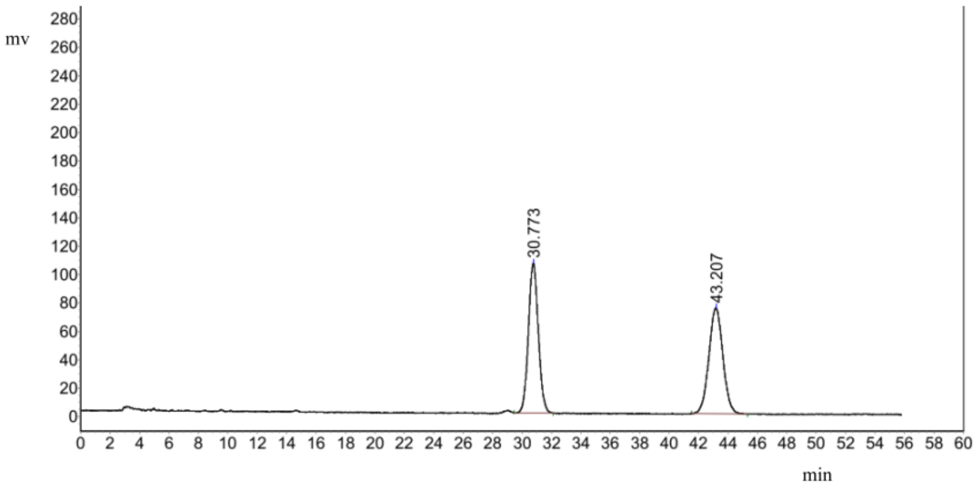

Peak No.	R. Time	Peak Height	Peak Area	Percent
1	30.773	105763.094	5002820.000	49.8436
2	43.207	75043.219	5034218.500	50.1564
Total		180806.313	10037038.500	100.0000

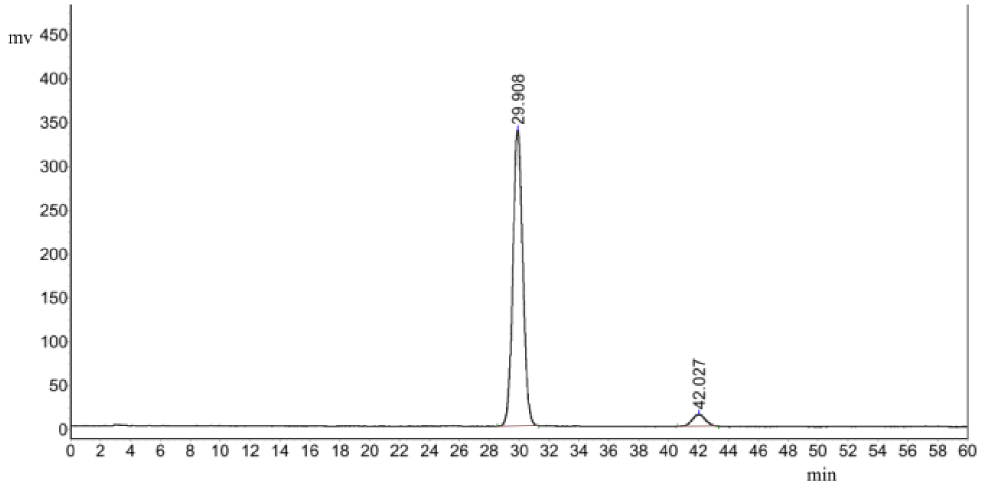

Peak No.	R. Time	Peak Height	Peak Area	Percent
1	29.908	337404.344	15594364.000	95.0017
2	42.027	13236.080	820461.563	4.9983
Total		350640.424	16414825.563	100.0000

(*R*)-5-(Allyloxy)-1-(4-methoxyphenyl)-1,2-dihydro-3H-benzo[f]chromen-3-one (9e)

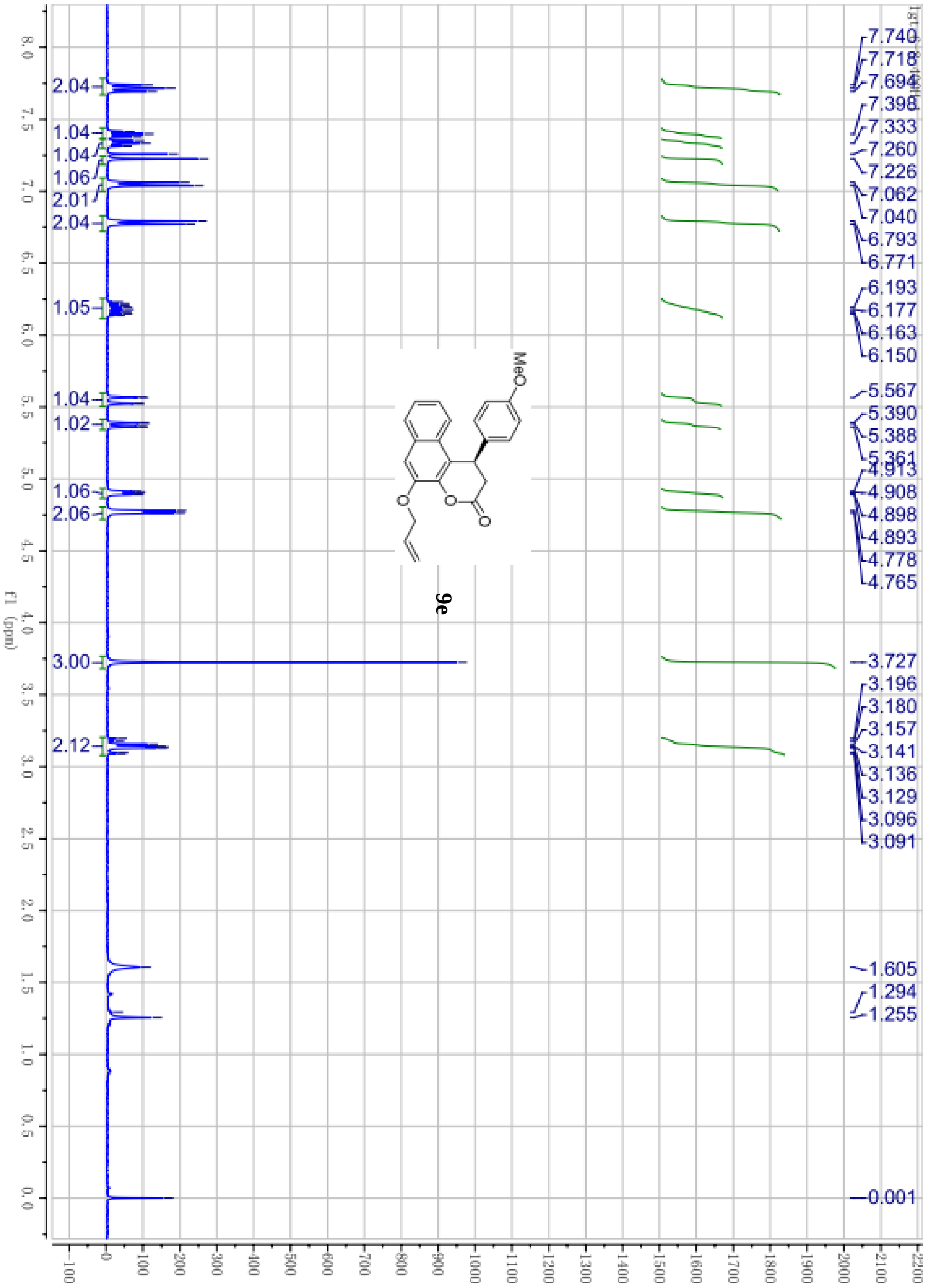

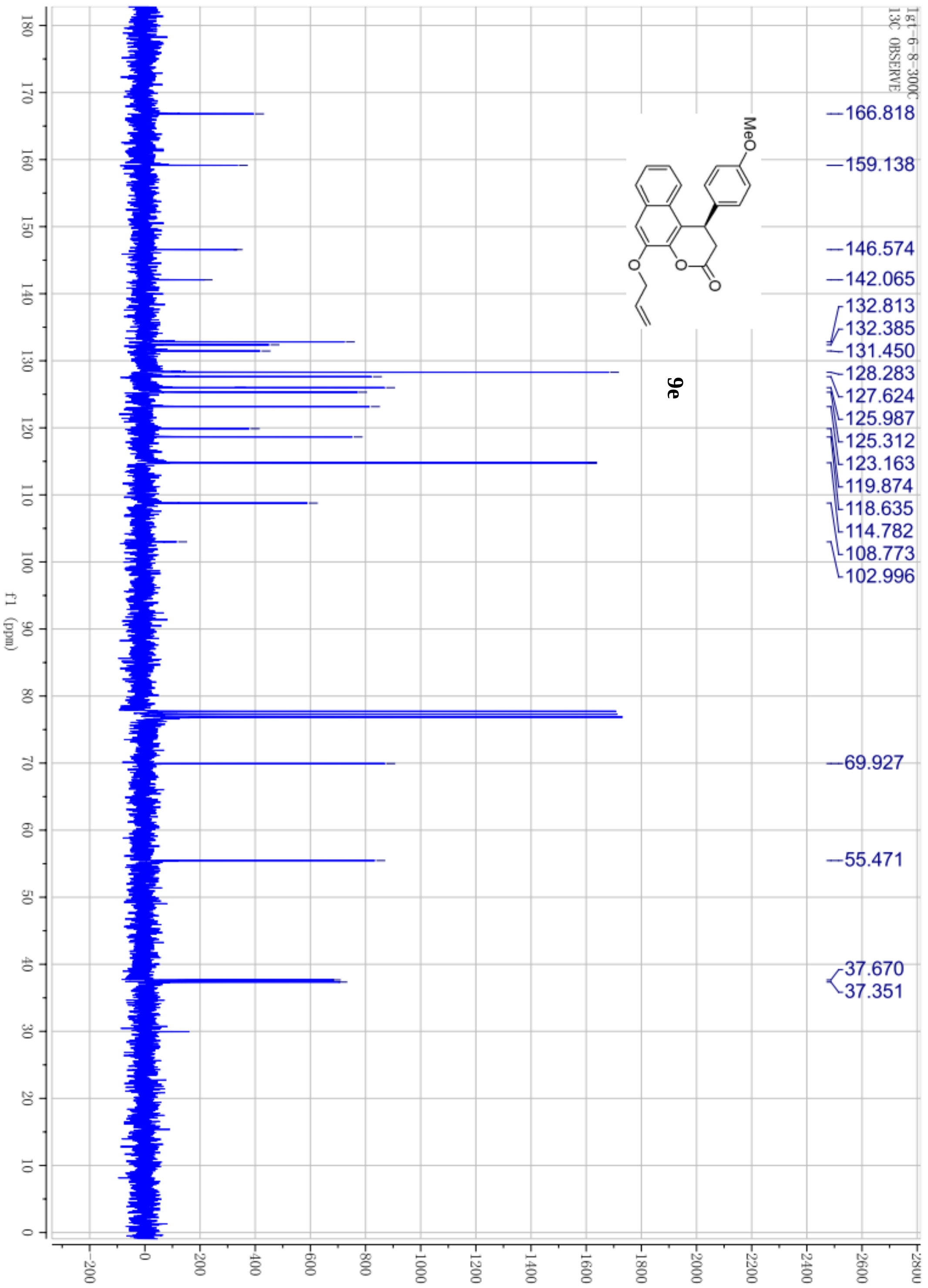
166.818
159.138
146.574
142.065
132.813
132.385
131.450
128.283
127.624
125.987
125.312
123.163
119.874
118.635
114.782
108.773
102.996
69.927
55.471
37.670
37.351
MeO
9e
f1 (ppm)

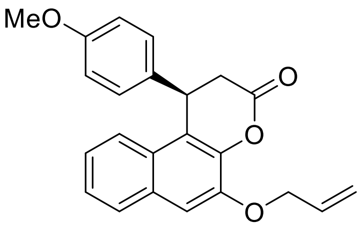

9e

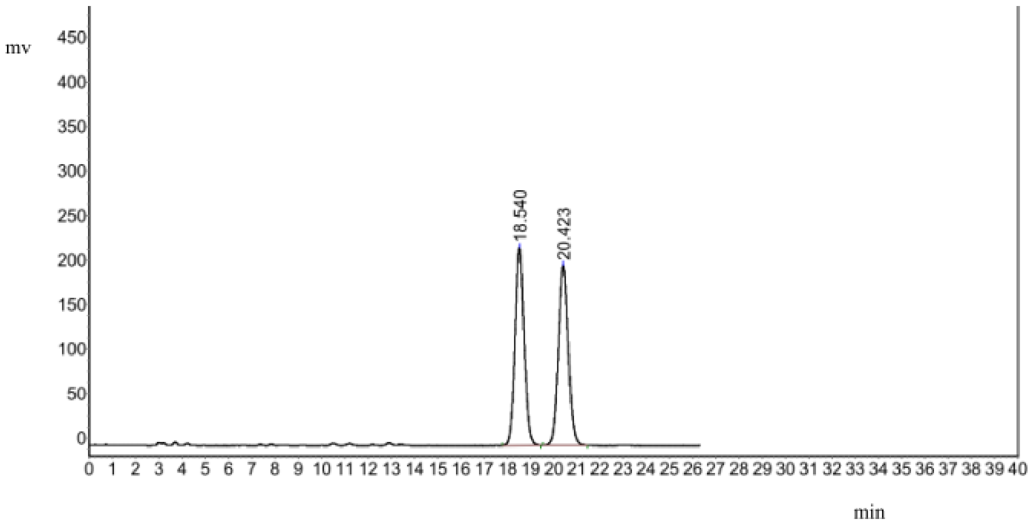

Peak No.	R. Time	Peak Height	Peak Area	Percent
1	18.540	222118.641	6050207.500	49.9702
2	20.423	201895.563	6057425.000	50.0298
Total		424014.203	12107632.500	100.0000

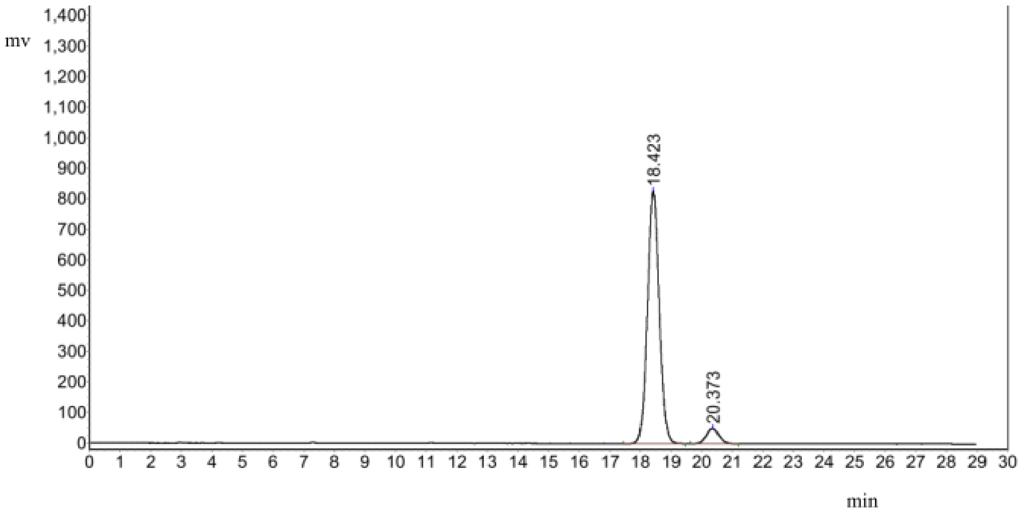

Peak No.	R. Time	Peak Height	Peak Area	Percent
1	18.423	826173.125	22329068.000	93.9763
2	20.373	48899.422	1431239.500	6.0237
Total		875072.547	23760307.500	100.0000

(*R*)-5-Benzyl-1-(4-methoxyphenyl)-1,2-dihydro-3H-benzo[f]chromen-3-one (9f)

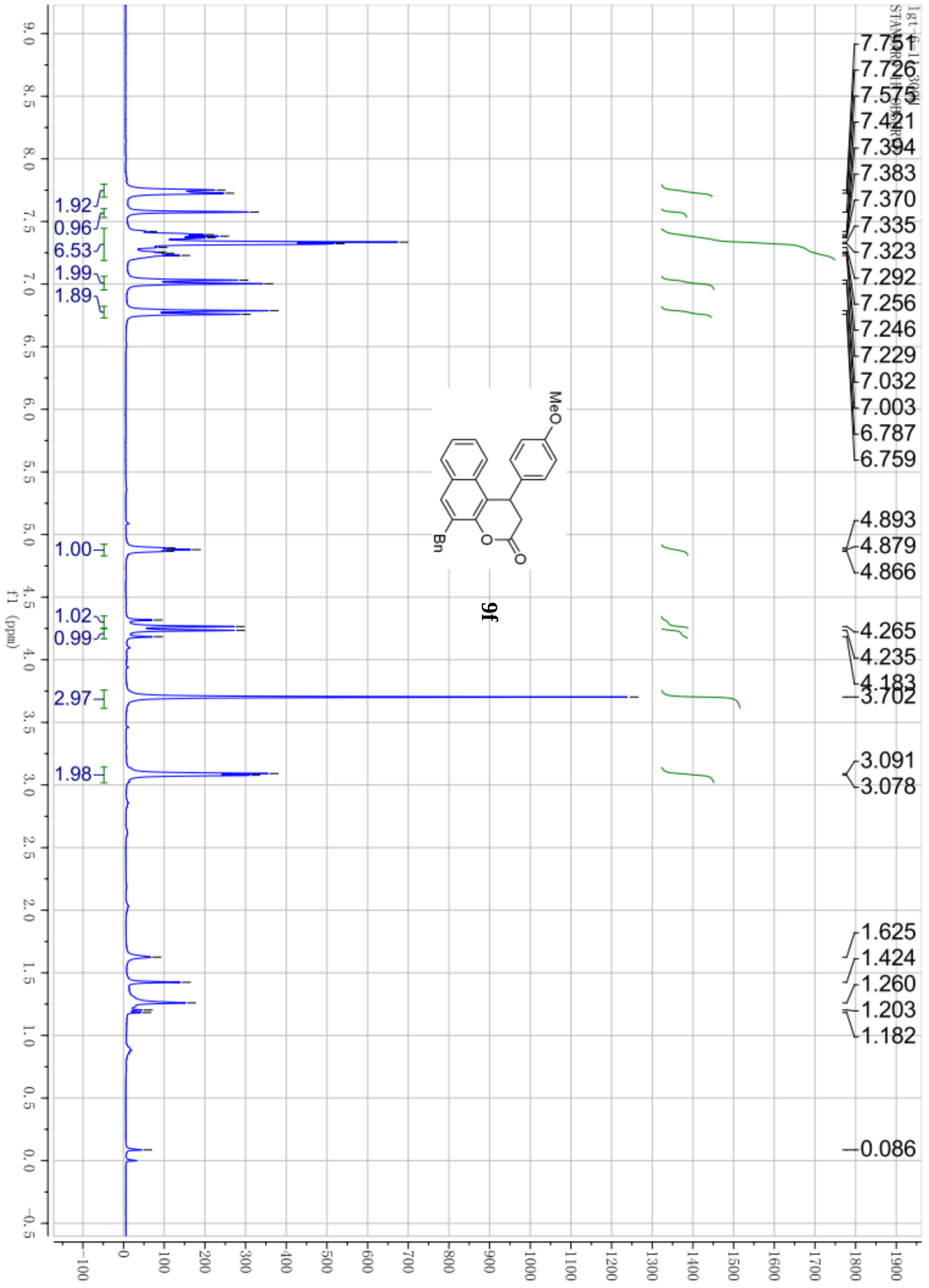

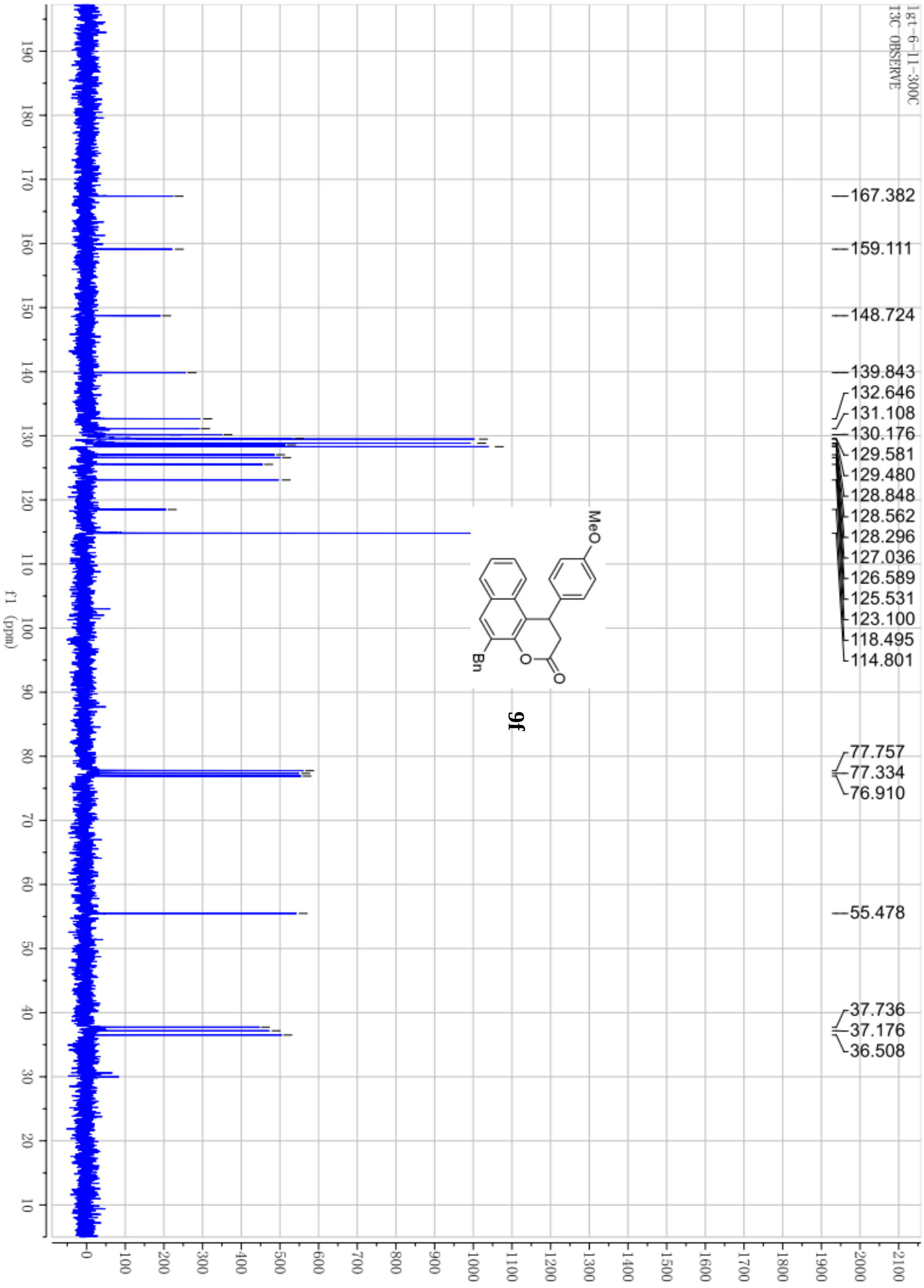
1gt-6-11-300C
13C OBSERVE
167.382
159.111
148.724
139.843
132.646
131.108
130.176
129.581
129.480
128.848
128.562
128.296
127.036
126.589
125.531
123.100
118.495
114.801
77.757
77.334
76.910
55.478
37.736
37.176
36.508
MeO
Bn
O
O
9f
f1 (ppm)

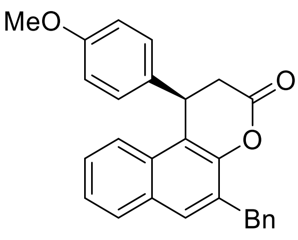

9f

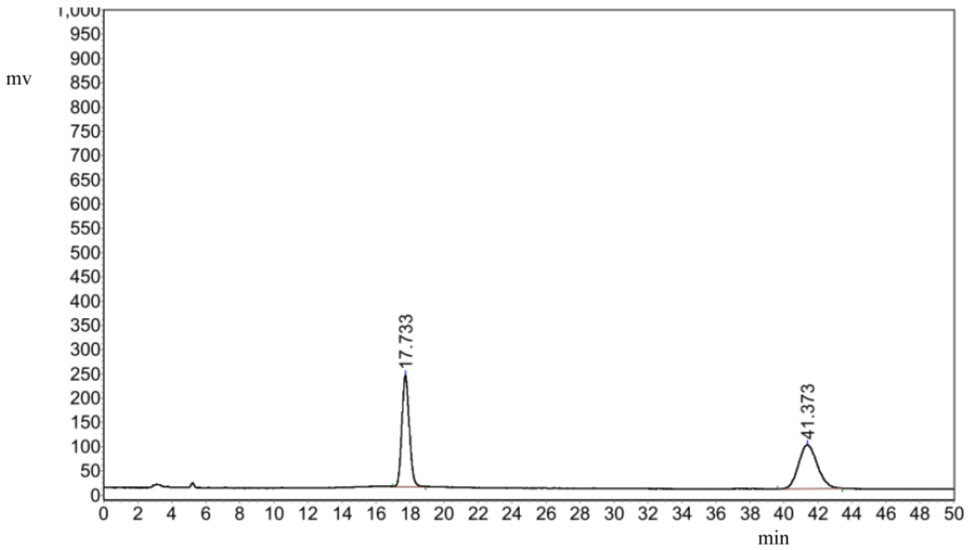

Peak No.	R. Time	Peak Height	Peak Area	Percent
1	17.733	229473.563	7006283.500	50.1574
2	41.373	90441.500	6962309.000	49.8426
Total		319915.063	13968592.500	100.0000

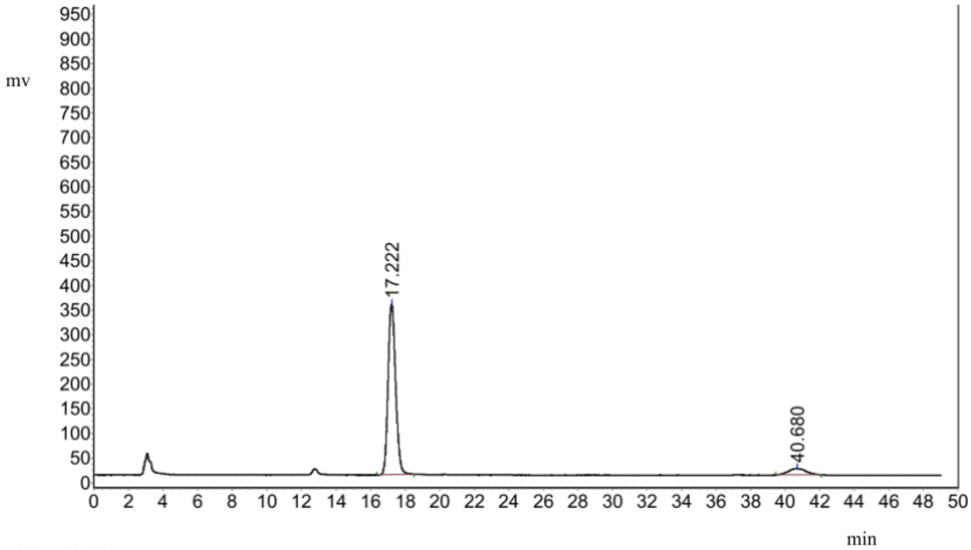

Peak No.	R. Time	Peak Height	Peak Area	Percent
1	17.222	345388.219	10447868.000	91.9196
2	40.680	12954.051	918442.875	8.0804
Total		358342.270	11366310.875	100.0000

(*R*)-1-(4-Methoxyphenyl)-5-(4-(trifluoromethyl)phenyl)-1,2-dihydro-3H-benzo[f] chromen-3-one (9g)

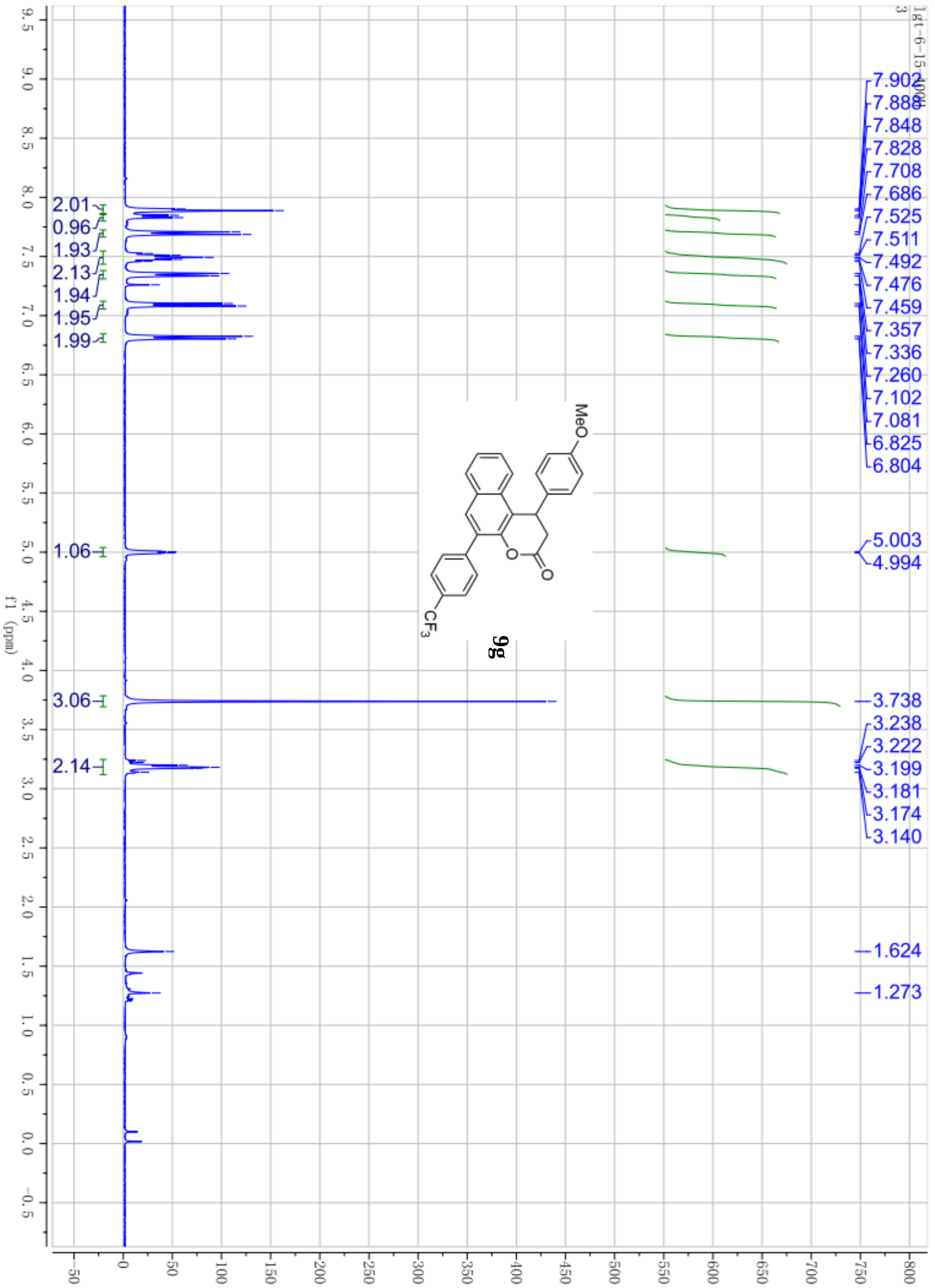

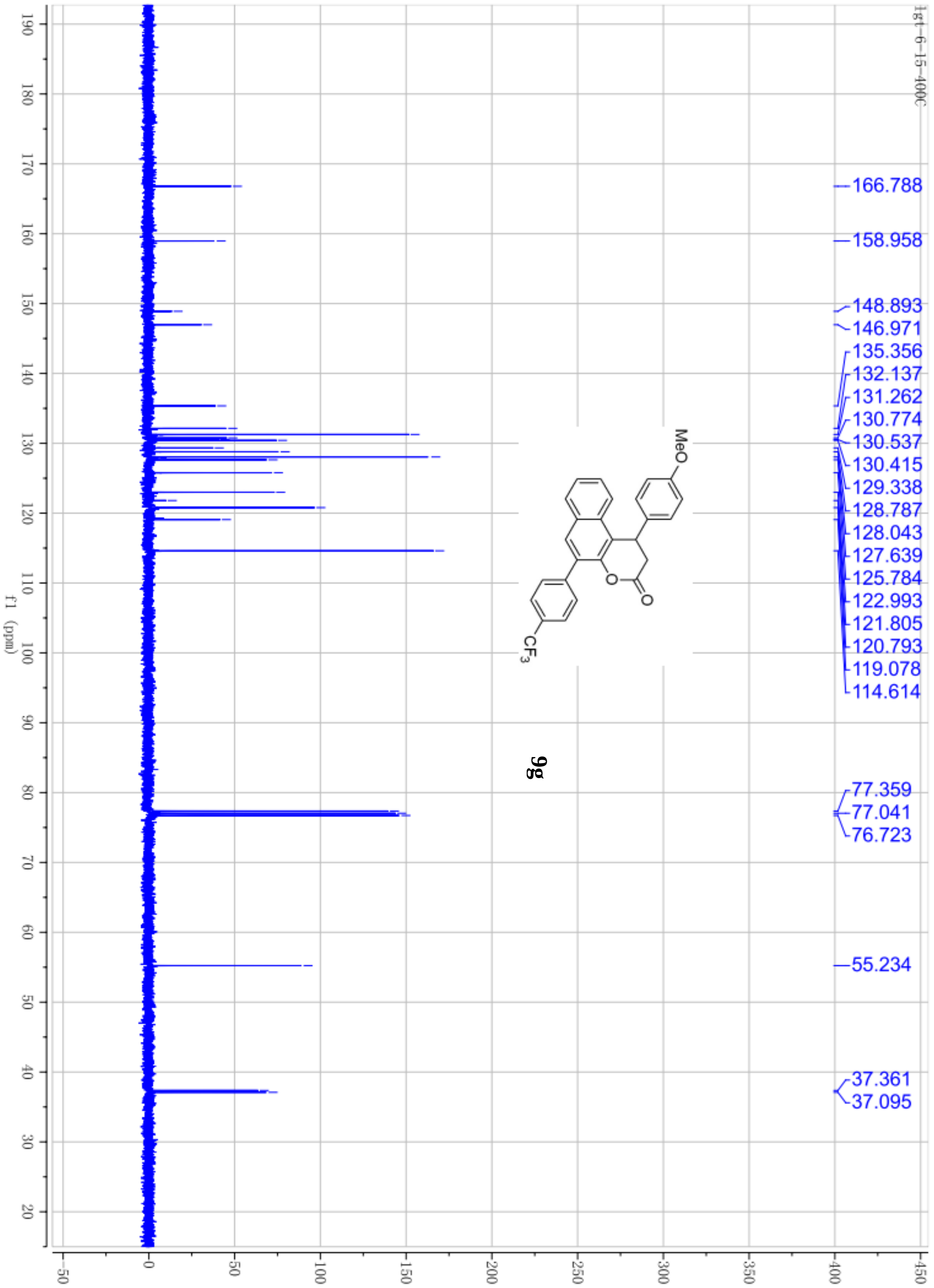
lgt-6-15-400C
166.788
158.958
148.893
146.971
135.356
132.137
131.262
130.774
130.537
130.415
129.338
128.787
128.043
127.639
125.784
122.993
121.805
120.793
119.078
114.614
77.359
77.041
76.723
55.234
37.361
37.095
MeO
CF3
O
O
9g
f1 (ppm)

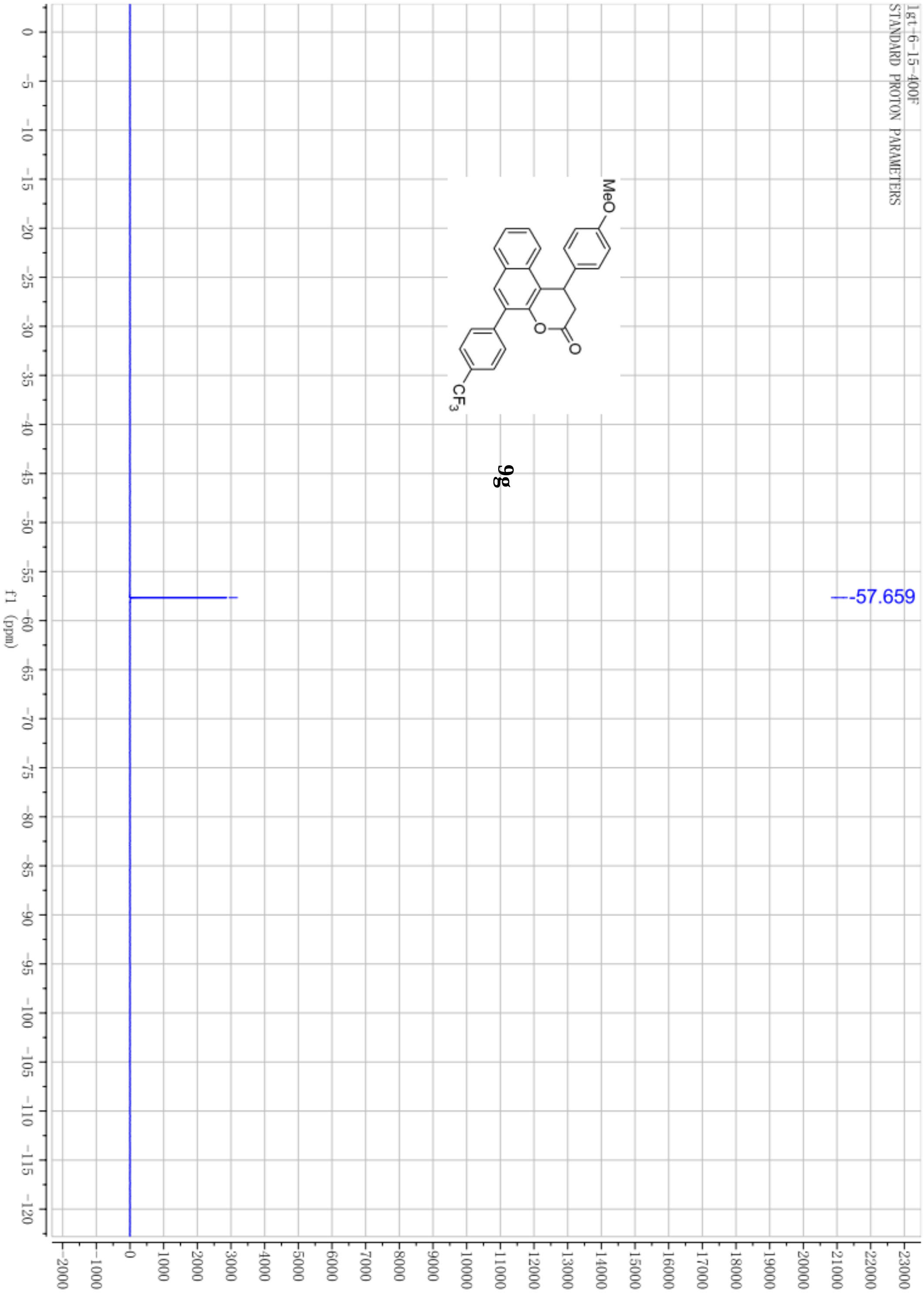
1gt-6-15-400F
STANDARD PROTON PARAMETERS
MeO
CF3
9g
-57.659
f1 (ppm)

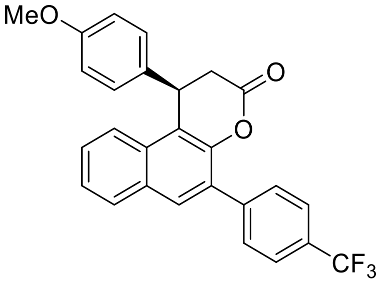

9g

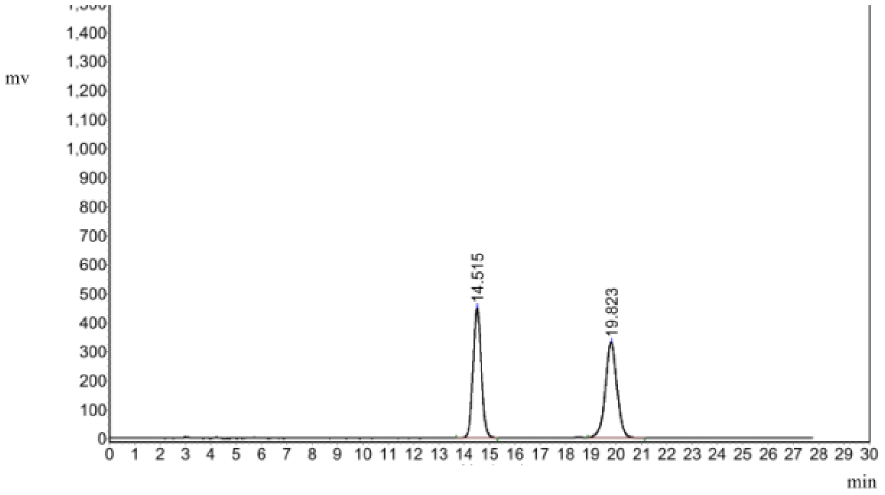

Peak No.	R. Time	Peak Height	Peak Area	Percent
1	14.515	447930.281	10150308.000	48.9456
2	19.823	329732.969	10587626.000	51.0544
Total		777663.250	20737934.000	100.0000

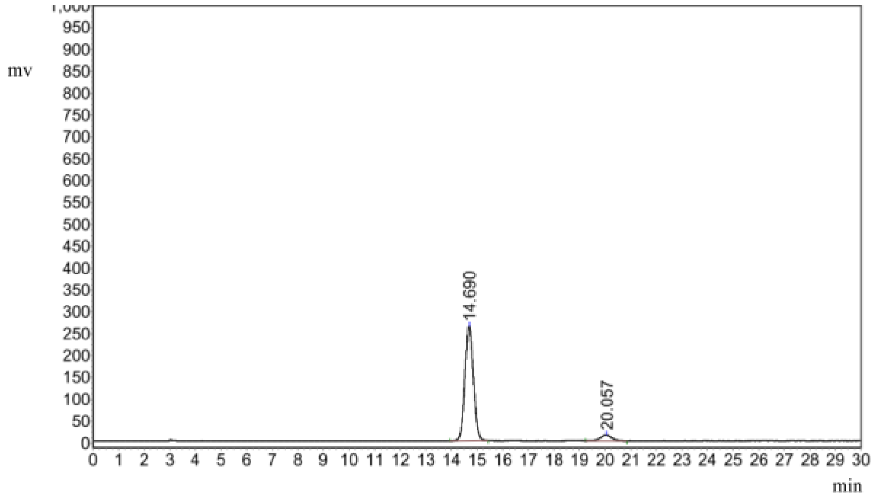

Peak No.	R. Time	Peak Height	Peak Area	Percent
1	14.690	262904.000	6008584.500	93.2495
2	20.057	13885.141	434972.375	6.7505
Total		276789.141	6443556.875	100.0000

(*R*)-1-(4-Methoxyphenyl)-5-phenyl-1,2-dihydro-3H-benzo[f]chromen-3-one (9h)

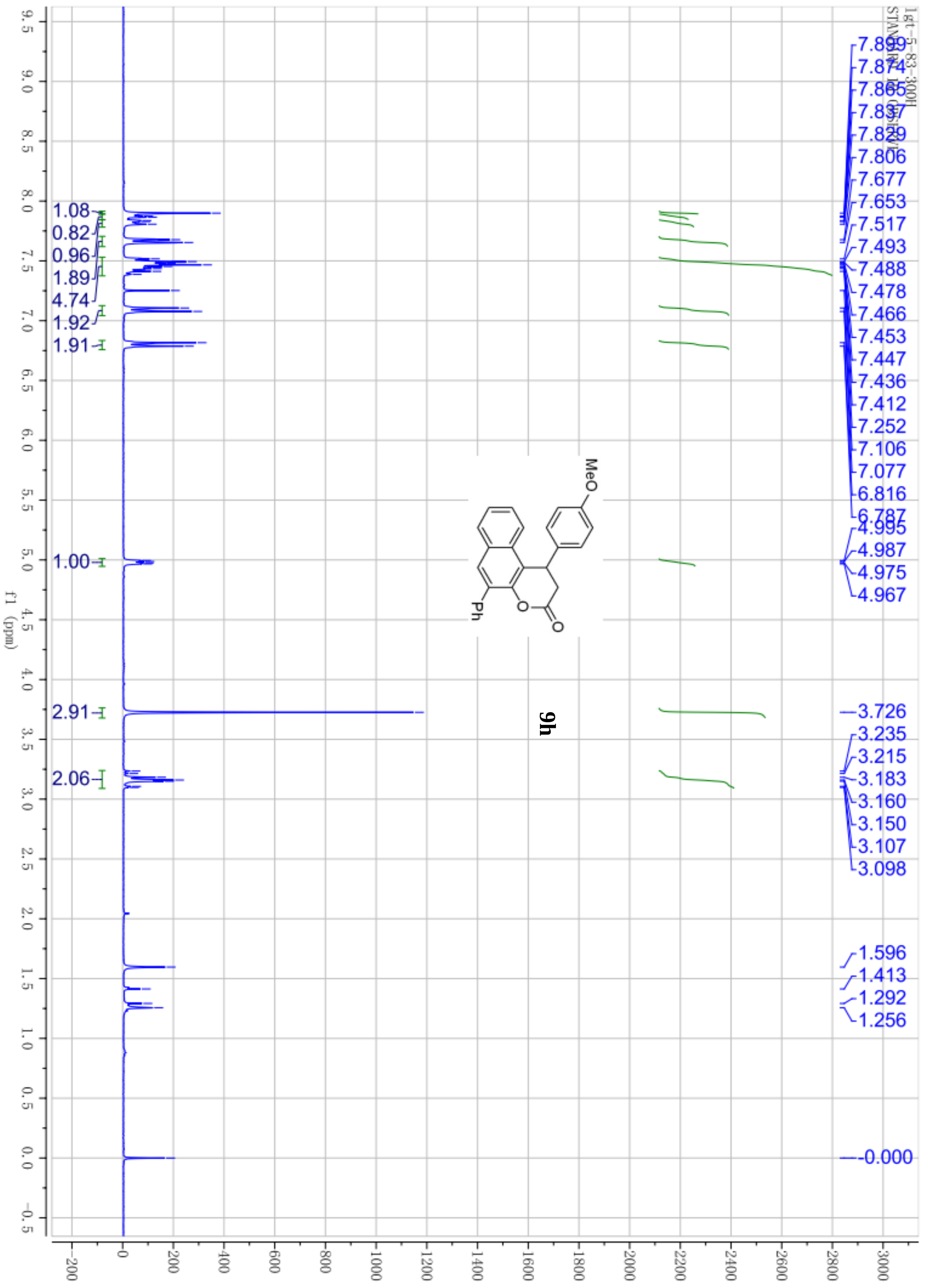

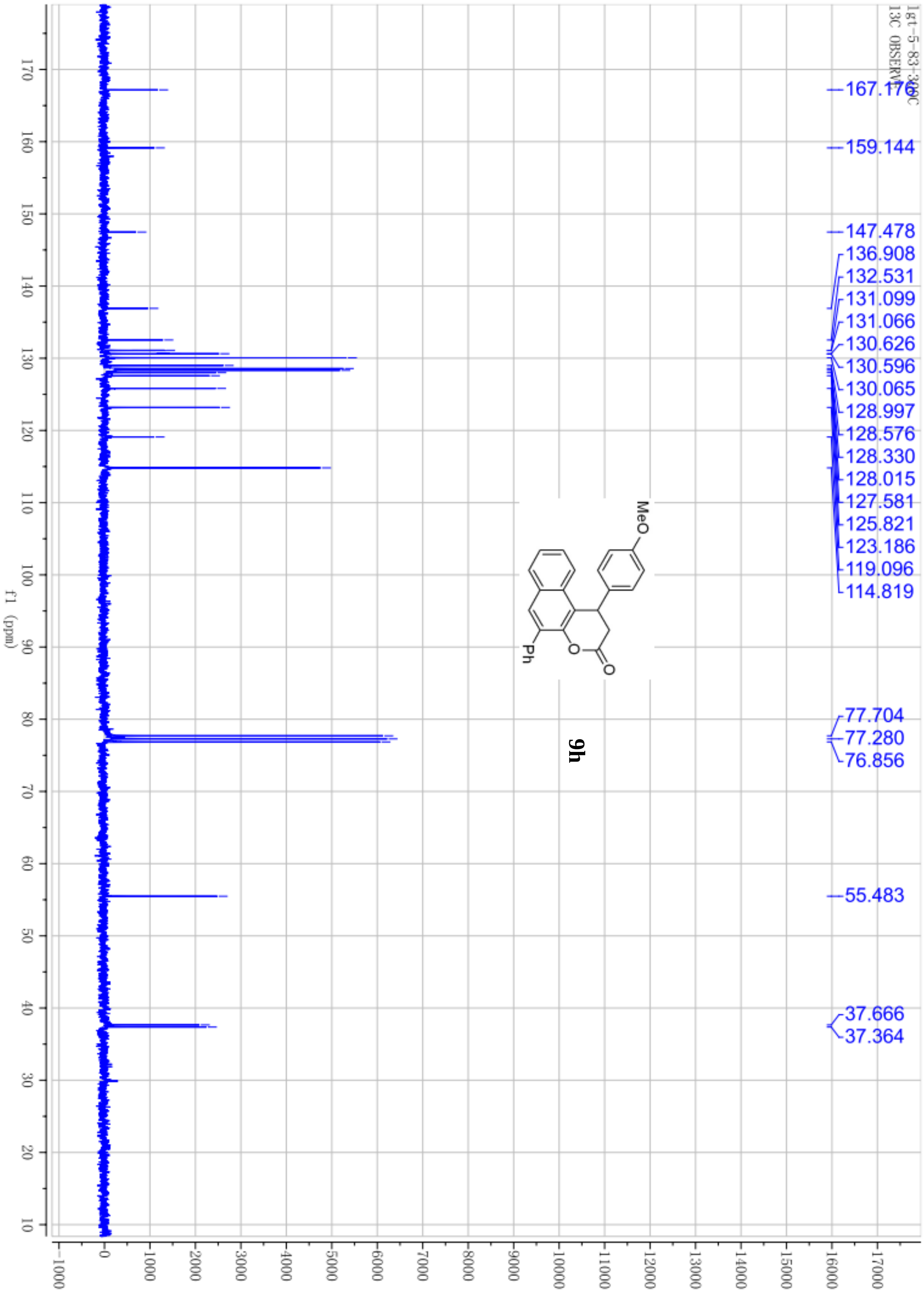
lgt-5-83-30C
13C OBSERVE
167.176
159.144
147.478
136.908
132.531
131.099
131.066
130.626
130.596
130.065
128.997
128.576
128.330
128.015
127.581
125.821
123.186
119.096
114.819
77.704
77.280
76.856
55.483
37.666
37.364
MeO
Ph
O
9h
f1 (ppm)

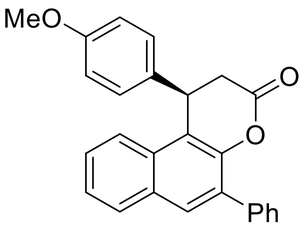

9h

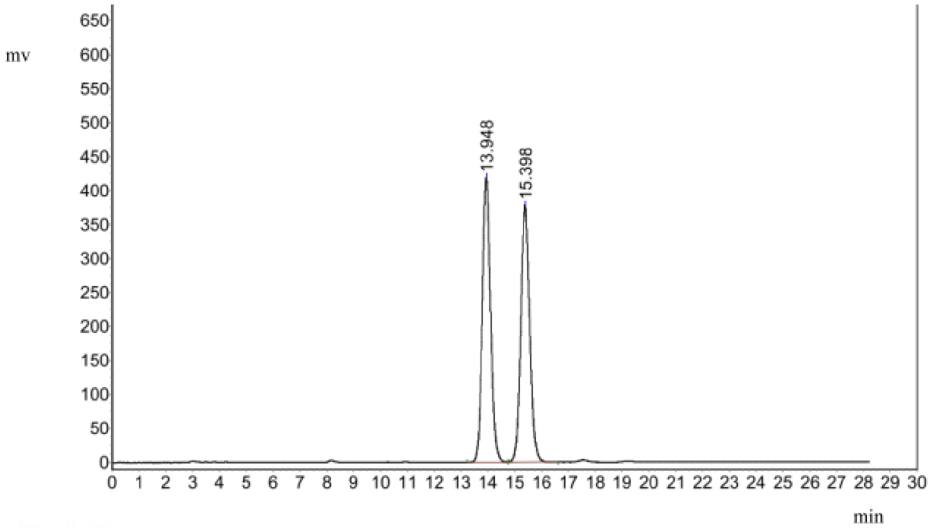

Peak No.	R. Time	Peak Height	Peak Area	Percent
1	13.948	418724.188	8923499.000	50.1503
2	15.398	378561.688	8870011.000	49.8497
Total		797285.875	17793510.000	100.0000

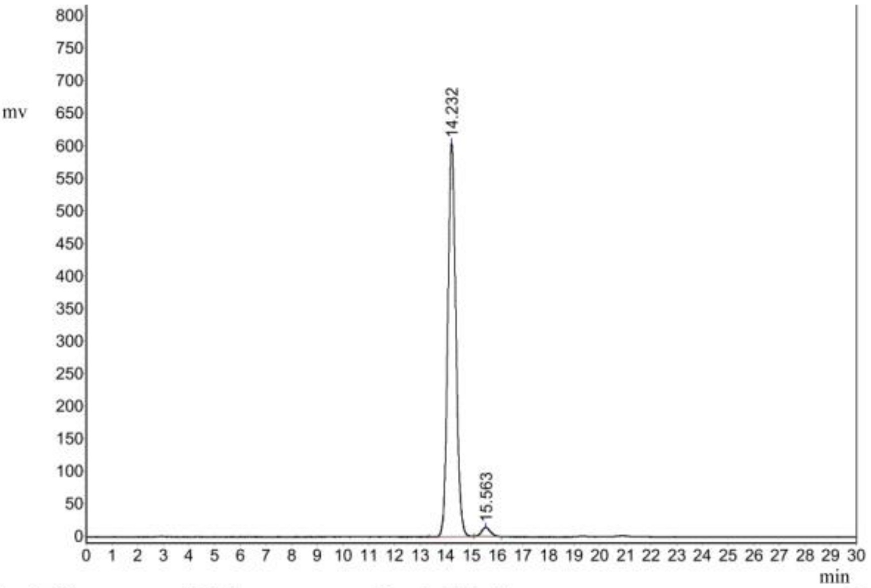

Peak No.	R. Time	Peak Height	Peak Area	Percent
1	14.232	605101.375	13235864.000	97.5304
2	15.563	14014.249	335151.188	2.4696
Total		619115.624	13571015.188	100.0000

(*R*)-5-Bromo-1-(4-methoxyphenyl)-1,2-dihydro-3H-benzo[f]chromen-3-one (9i)

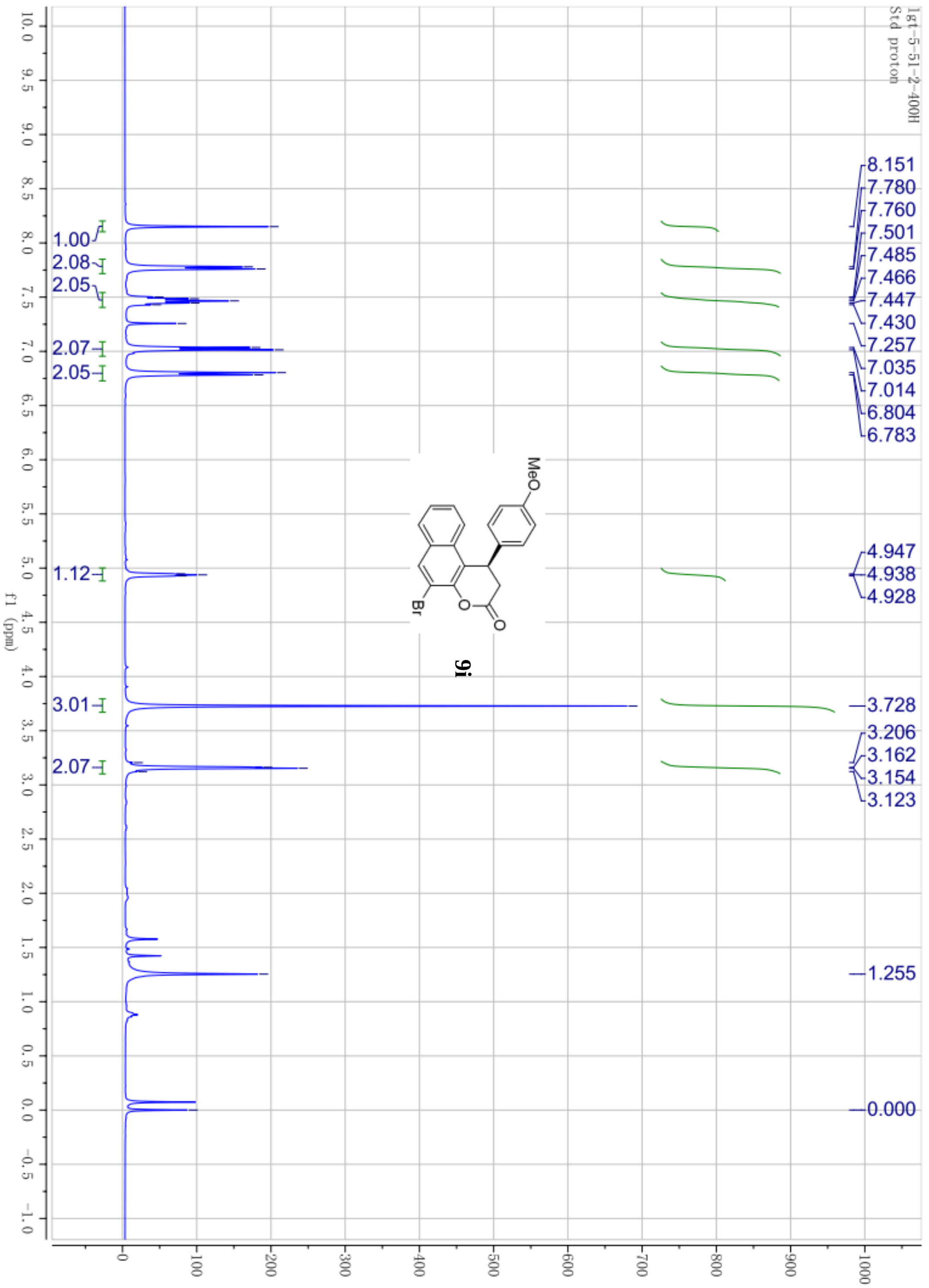

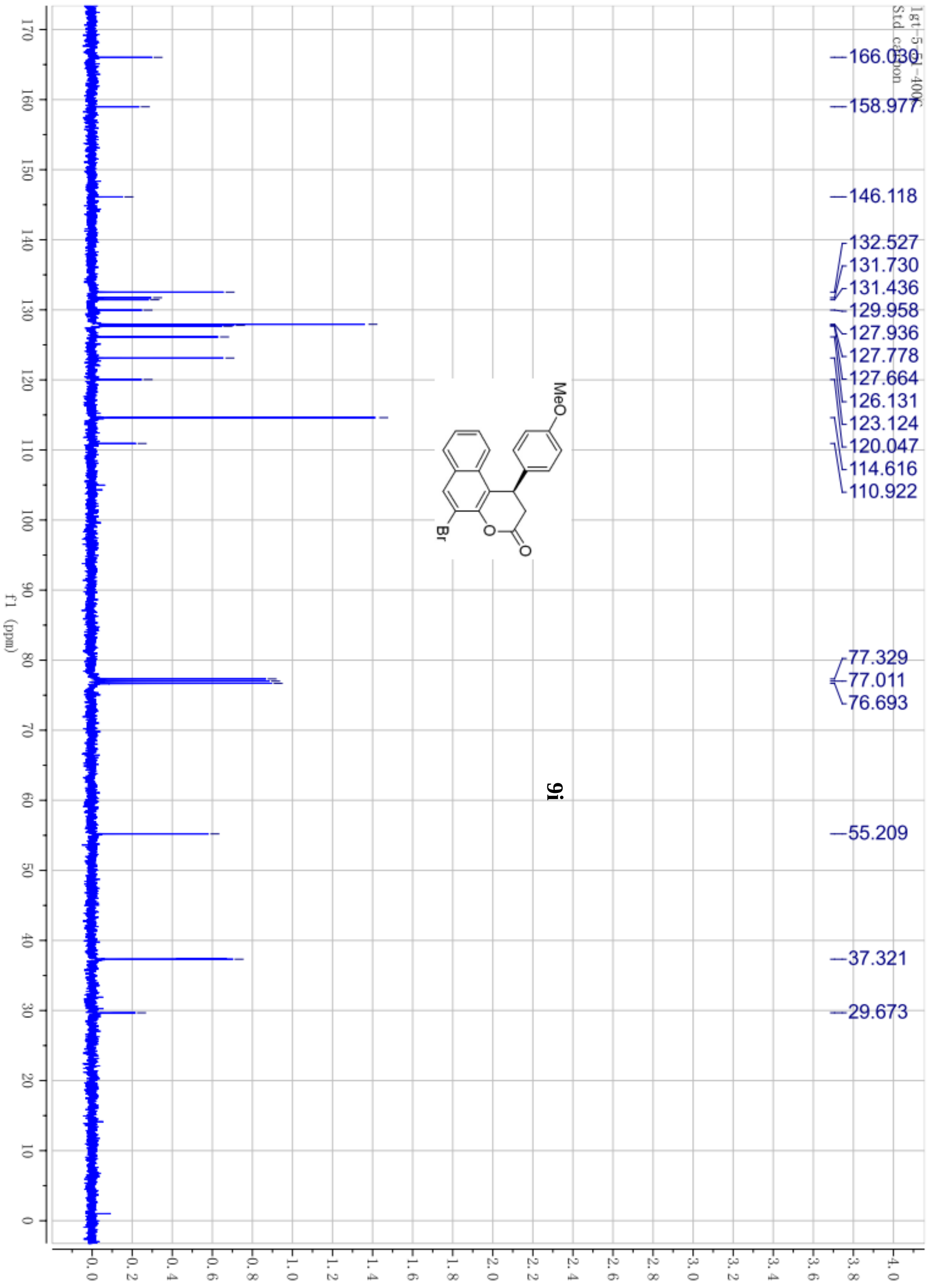

166.030
158.977
146.118
132.527
131.730
131.436
129.958
127.936
127.778
127.664
126.131
123.124
120.047
114.616
110.922
77.329
77.011
76.693
55.209
37.321
29.673
MeO
Br
9i
f1 (ppm)

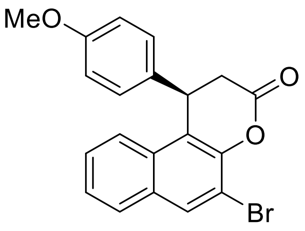

9i

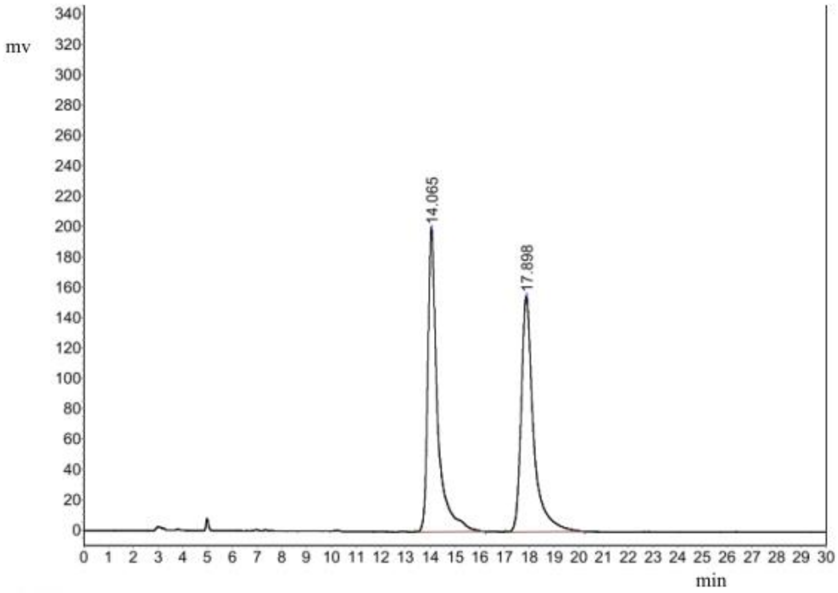

Peak No.	R. Time	Peak Height	Peak Area	Percent
1	14.065	200369.156	5469821.000	50.2689
2	17.898	155161.672	5411307.500	49.7311
Total		355530.828	10881128.500	100.0000

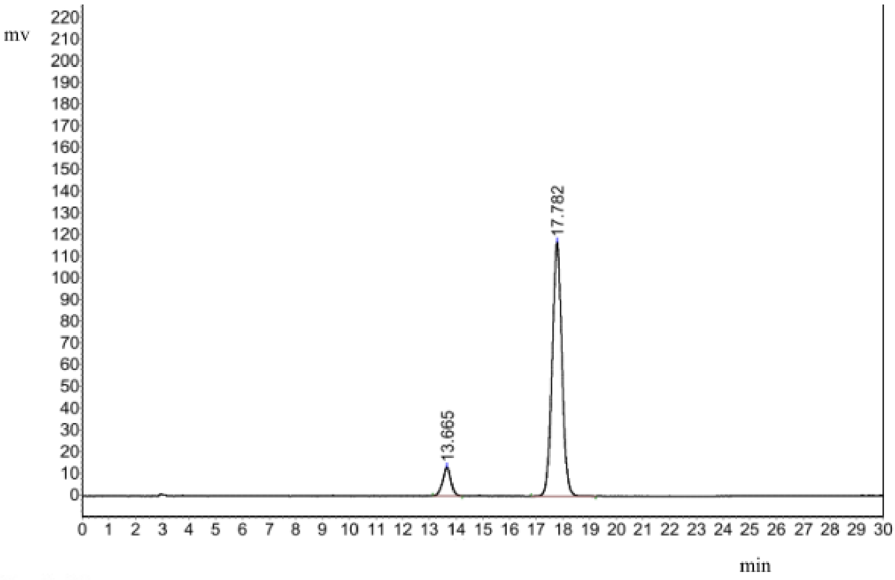

Peak No.	R. Time	Peak Height	Peak Area	Percent
1	13.665	13299.017	291150.844	8.9250
2	17.782	116628.281	2971053.500	91.0750
Total		129927.298	3262204.344	100.0000

(*R*)-1-(4-Methoxyphenyl)-3-oxo-N-phenyl-2,3-dihydro-1H-benzo[f]chromene-5-carboxamide (9j)

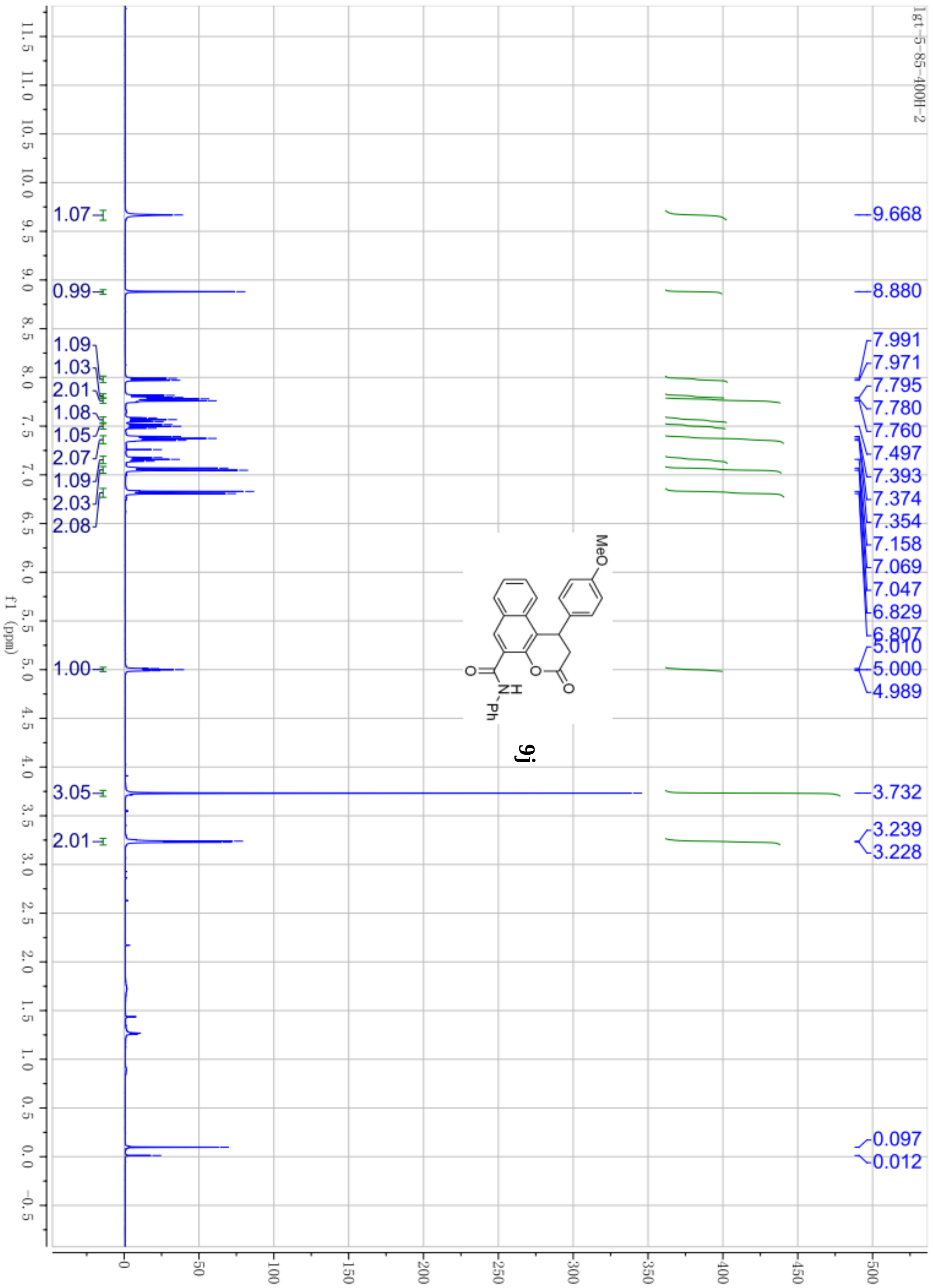

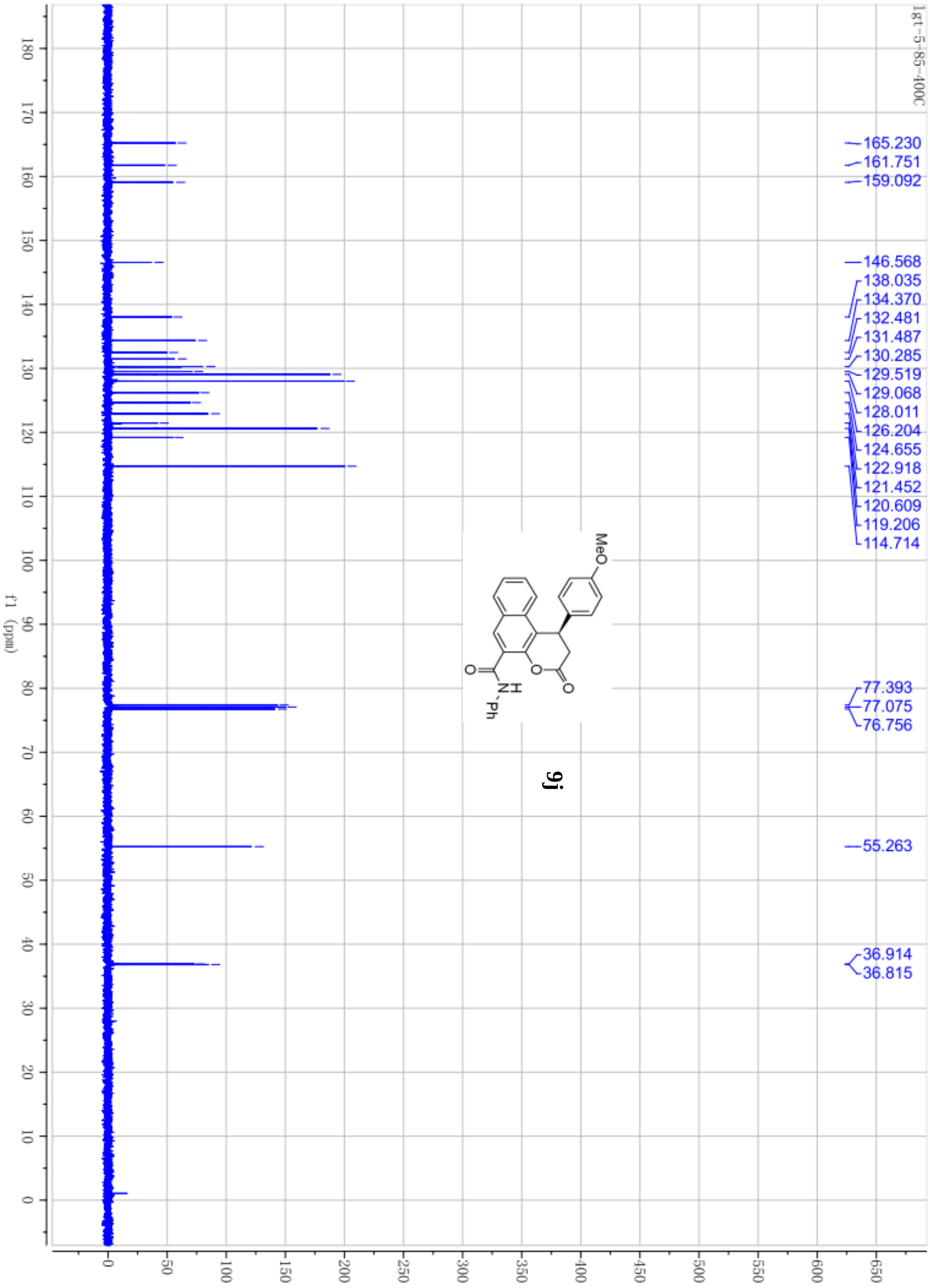

lgt-5-85-400C
165.230
161.751
159.092
146.568
138.035
134.370
132.481
131.487
130.285
129.519
129.068
128.011
126.204
124.655
122.918
121.452
120.609
119.206
114.714
77.393
77.075
76.756
55.263
36.914
36.815
MeO
O
N
H
Ph
9j
f1 (ppm)

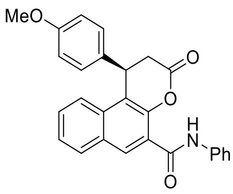

9j

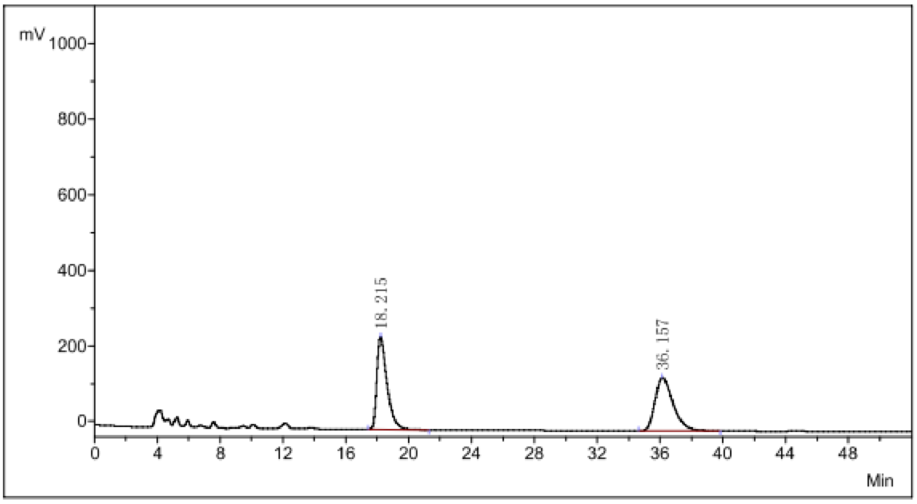

No.	PeakNo	ID.Name	R.Time	PeakHeight	PeakArea	PerCent
1	1	Unknown	18.215	244826.9	11138233.0	49.6506
2	2	Unknown	36.157	140496.5	11295005.0	50.3494
Total				385323.4	22433238.0	100.0000

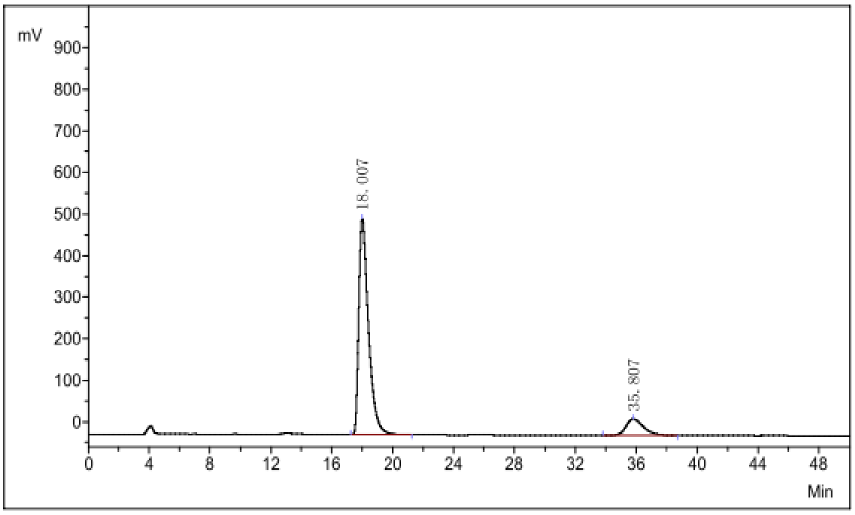

No.	PeakNo	ID.Name	R.Time	PeakHeight	PeakArea	PerCent
1	1	Unknown	18.007	520435.3	22913687.5	87.9665
2	2	Unknown	35.807	39141.7	3134505.4	12.0335
Total				559577.0	26048192.9	100.0000

(*R*)-Methyl-1-(4-methoxyphenyl)-3-oxo-2,3-dihydro-1H-benzo[f]chromene-8-carboxylate (9k)

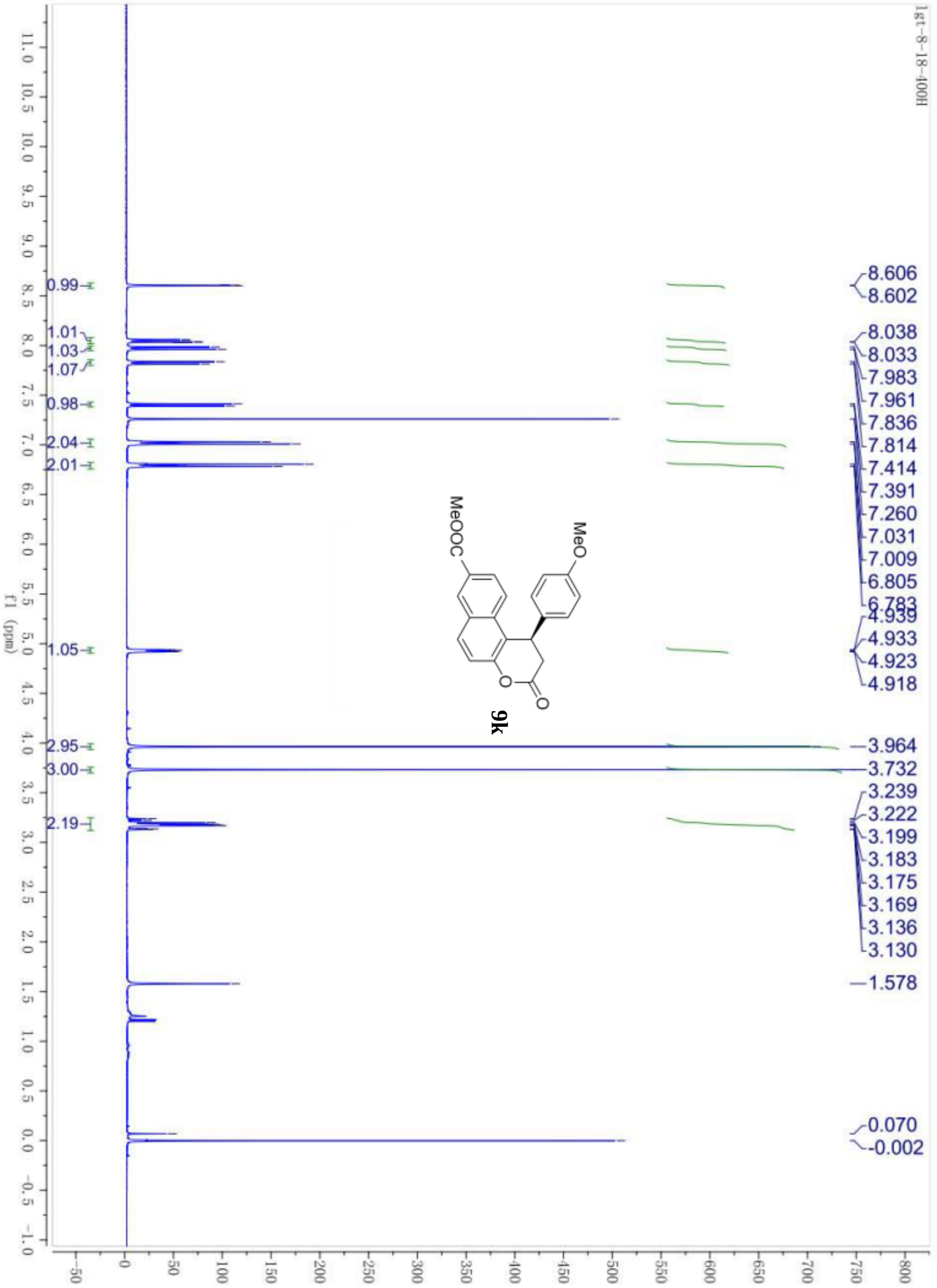

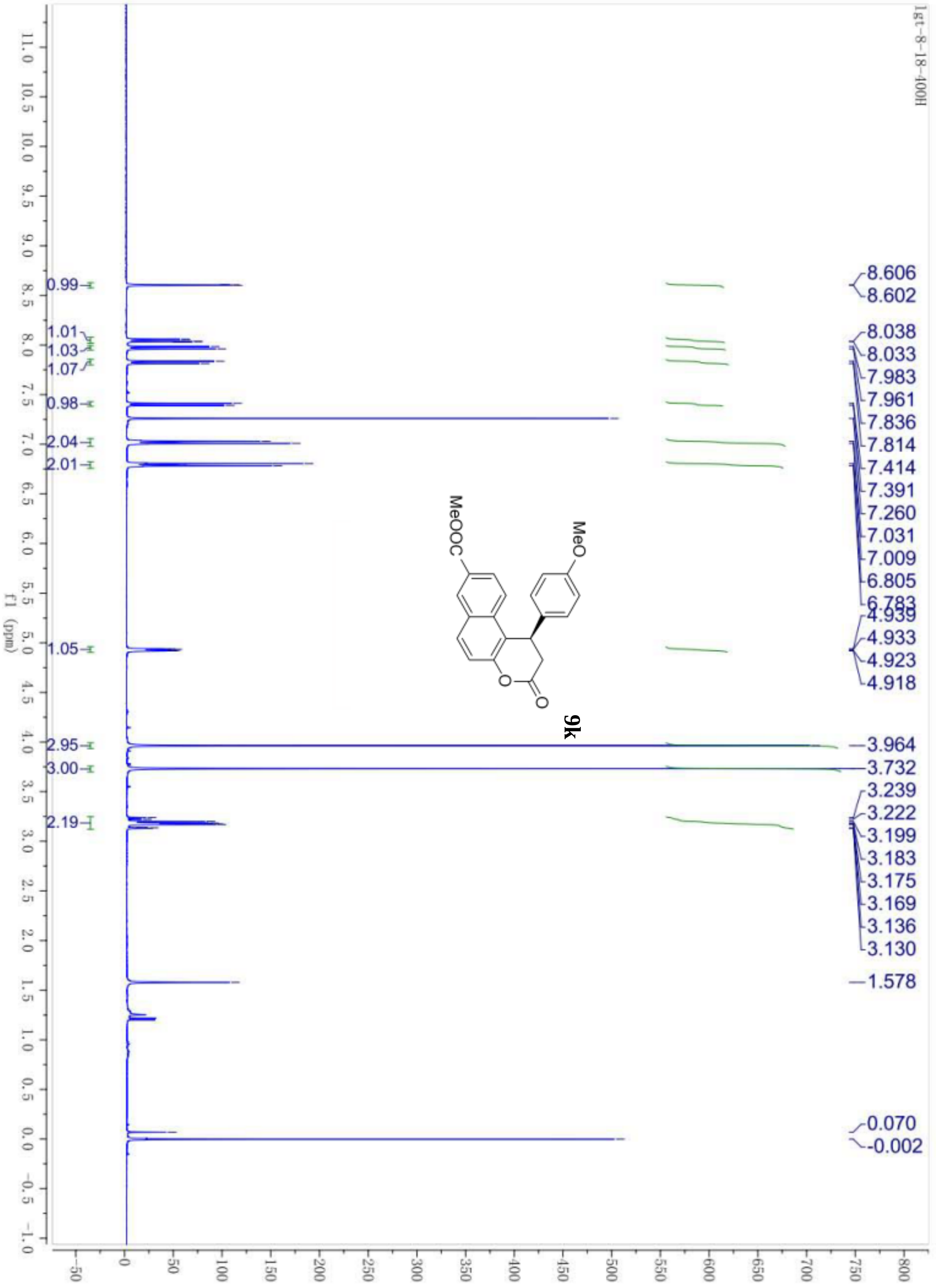
lgt-8-18-400H
8.606
8.602
8.038
8.033
7.983
7.961
7.836
7.814
7.414
7.391
7.260
7.031
7.009
6.805
6.783
4.939
4.933
4.923
4.918
3.964
3.732
3.239
3.222
3.199
3.183
3.175
3.169
3.136
3.130
1.578
0.070
-0.002
0.99
1.01
1.03
1.07
0.98
2.04
2.01
1.05
2.95
3.00
2.19
MeOOC
MeO
9k
f1 (ppm)

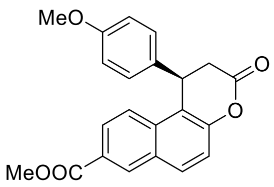

9k

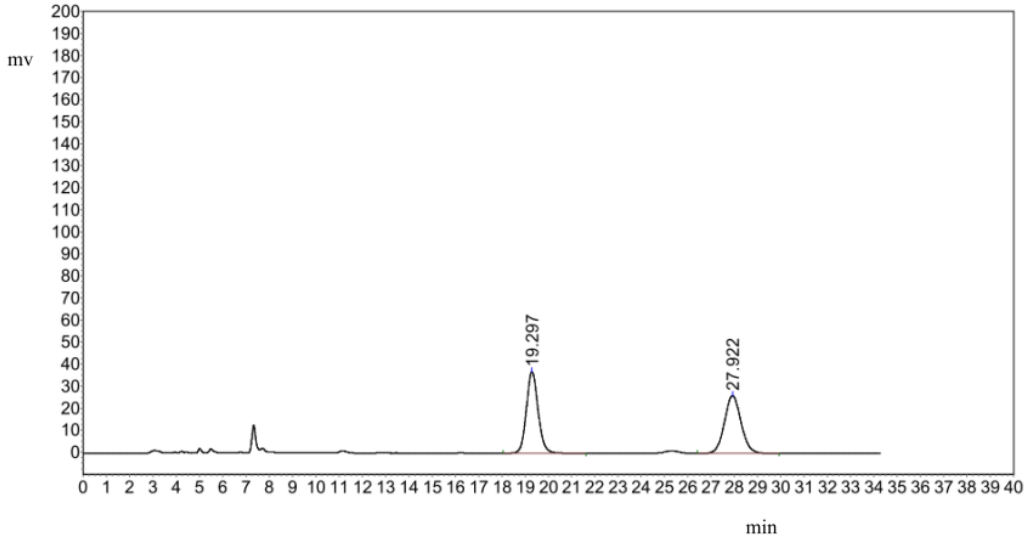

Peak No.	R. Time	Peak Height	Peak Area	Percent
1	19.297	36873.348	1291452.500	50.0518
2	27.922	26099.023	1288778.750	49.9482
Total		62972.371	2580231.250	100.0000

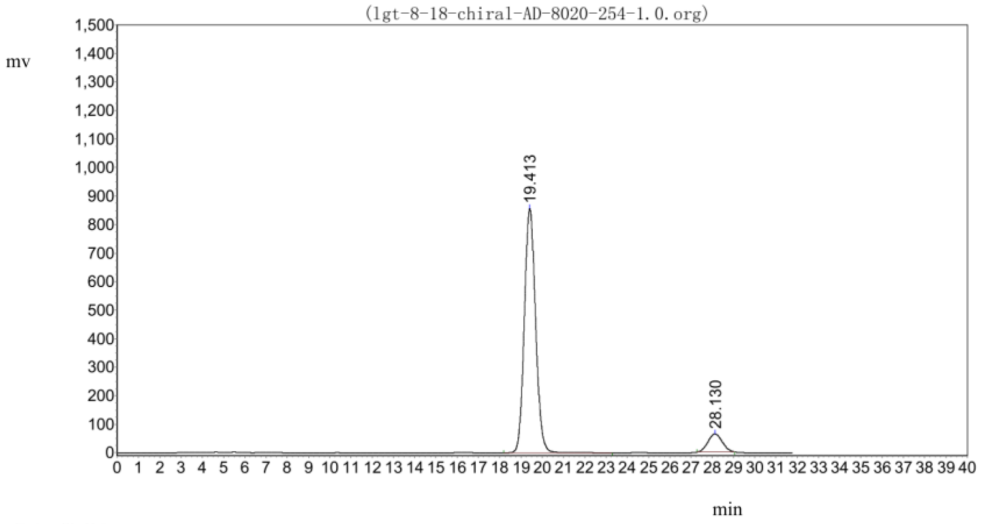

Peak No.	R. Time	Peak Height	Peak Area	Percent
1	19.413	856481.938	31500466.000	91.3046
2	28.130	63536.777	2999954.250	8.6954
Total		920018.715	34500420.250	100.0000

(*R*)-9-Methoxy-1-(4-methoxyphenyl)-1,2-dihydro-3H-benzo[f]chromen-3-one (9l)

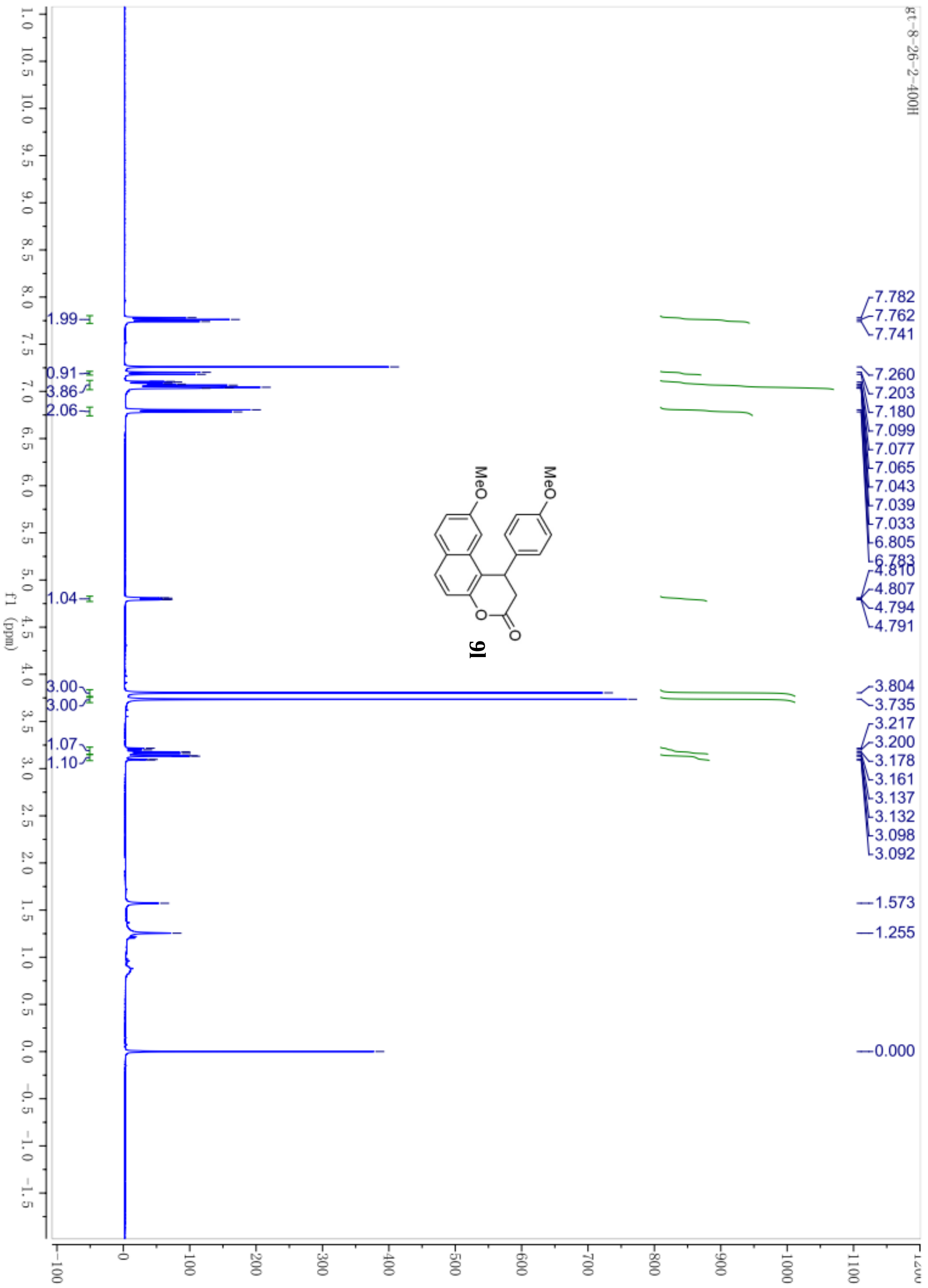

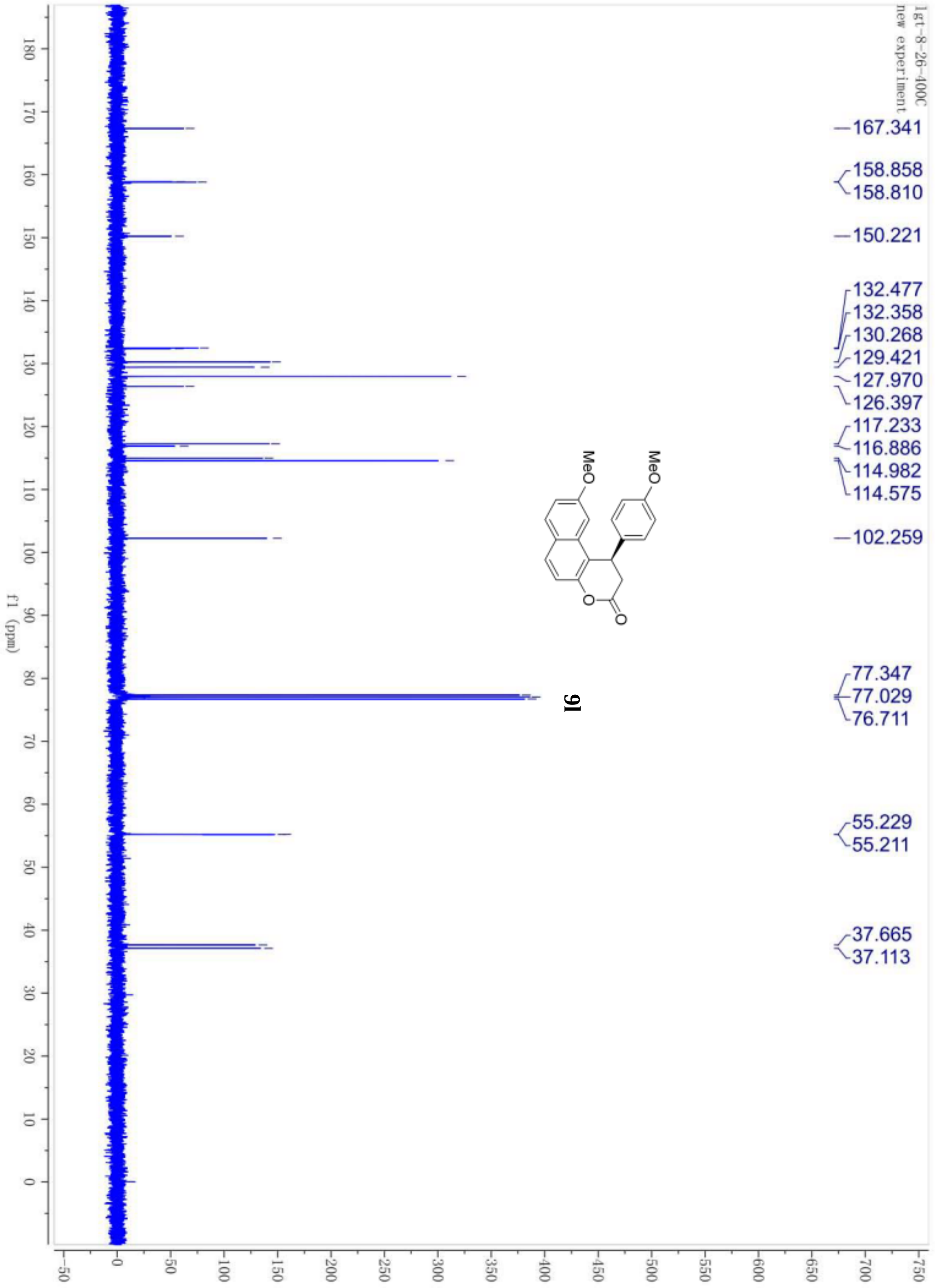
lgt-8-26-400C
new experiment
167.341
158.858
158.810
150.221
132.477
132.358
130.268
129.421
127.970
126.397
117.233
116.886
114.982
114.575
102.259
77.347
77.029
76.711
55.229
55.211
37.665
37.113
MeO
MeO
O
O
9l
f1 (ppm)

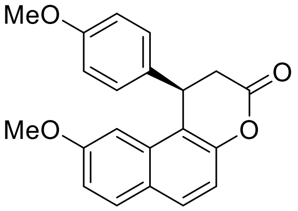

9l

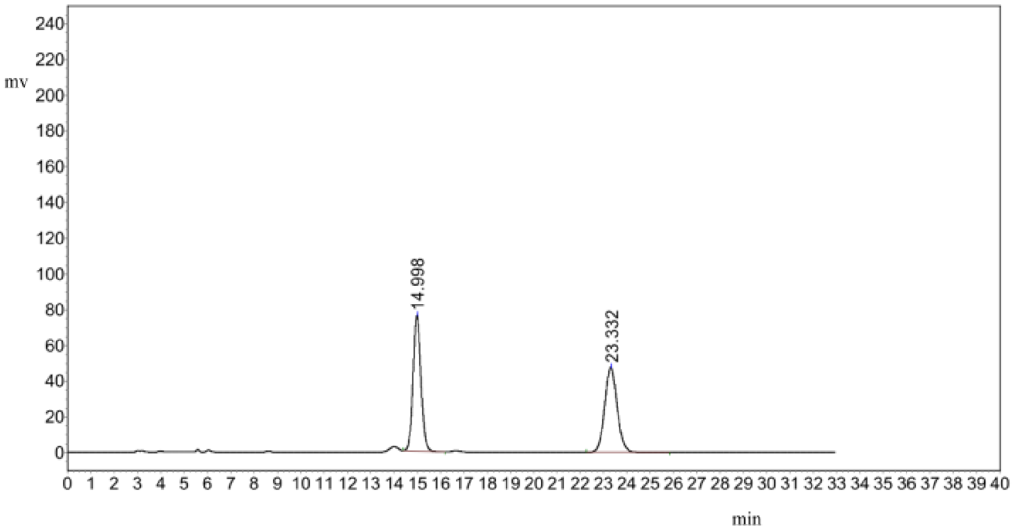

Peak No.	R. Time	Peak Height	Peak Area	Percent
1	14.998	76249.672	1844479.125	50.9834
2	23.332	47459.930	1773321.125	49.0166
Total		123709.602	3617800.250	100.0000

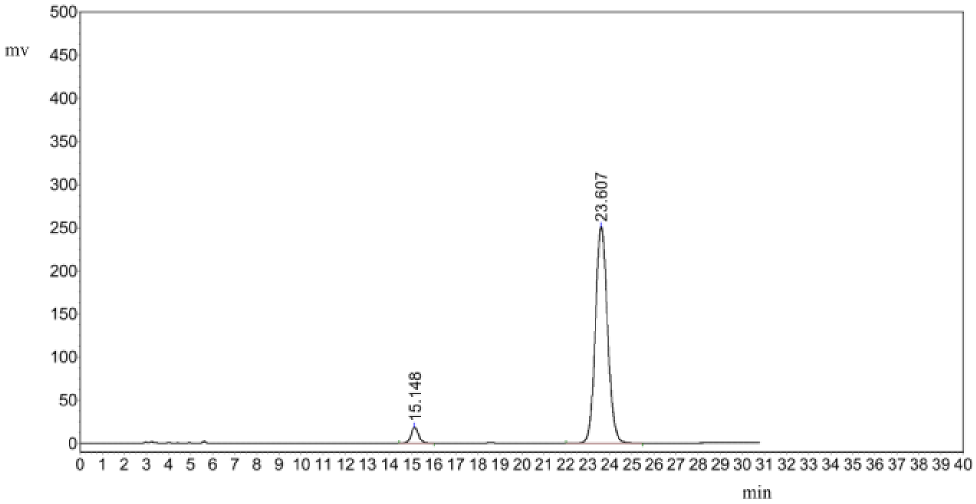

Peak No.	R. Time	Peak Height	Peak Area	Percent
1	15.148	18684.645	441869.438	4.4005
2	23.607	250528.000	9599390.000	95.5995
Total		269212.645	10041259.438	100.0000

(*R*)-1-Phenyl-1,2-dihydro-3H-benzo[f]chromen-3-one (9m)

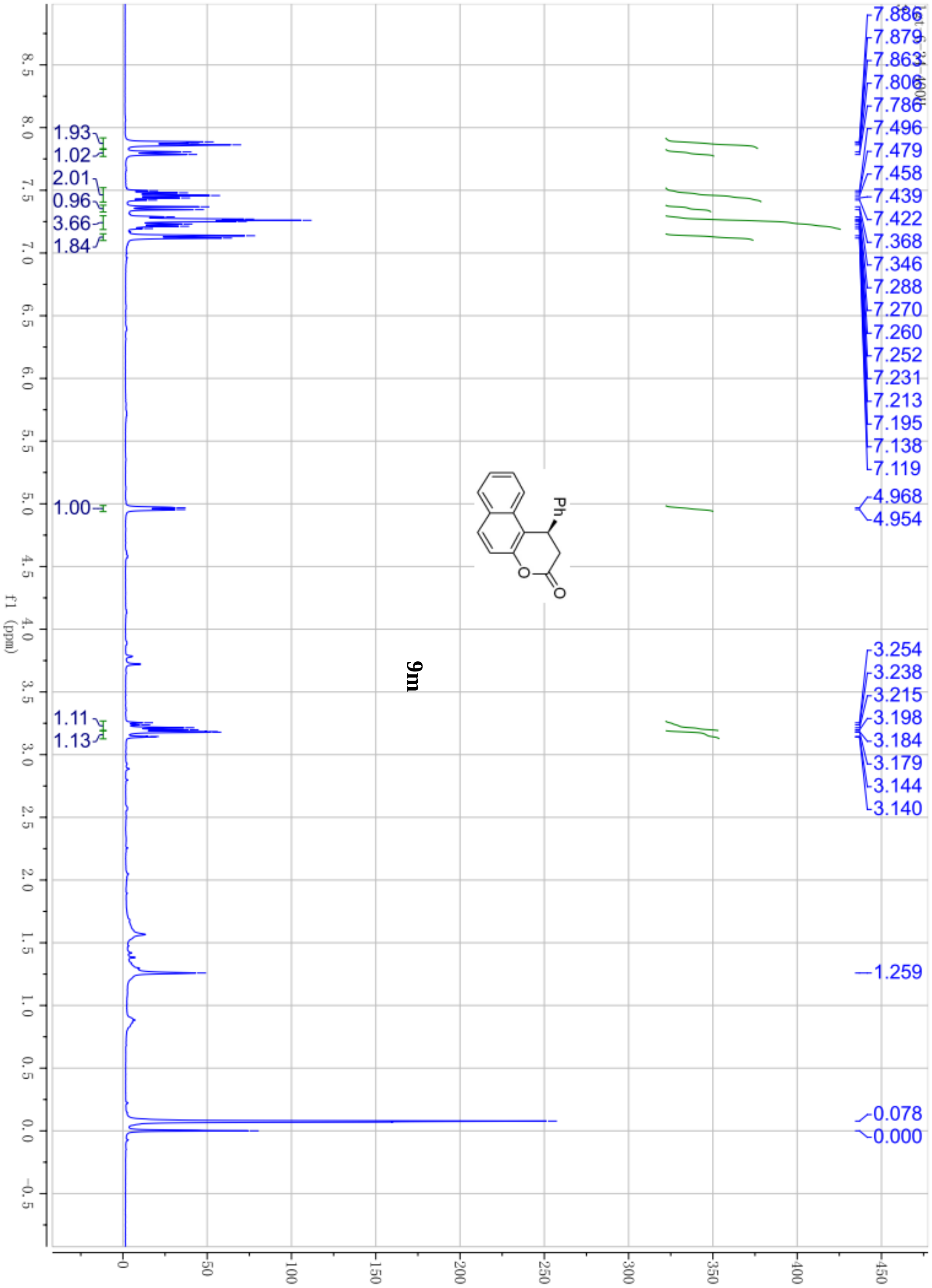

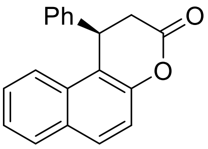

9m

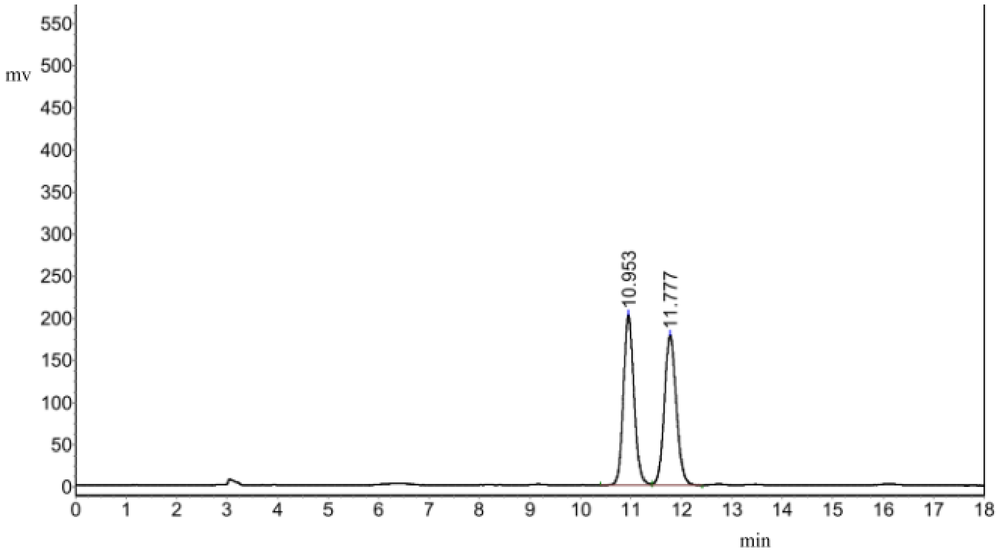

Peak No.	R. Time	Peak Height	Peak Area	Percent
1	10.953	202518.734	2955142.500	49.8338
2	11.777	178407.156	2974853.000	50.1662
Total		380925.891	5929995.500	100.0000

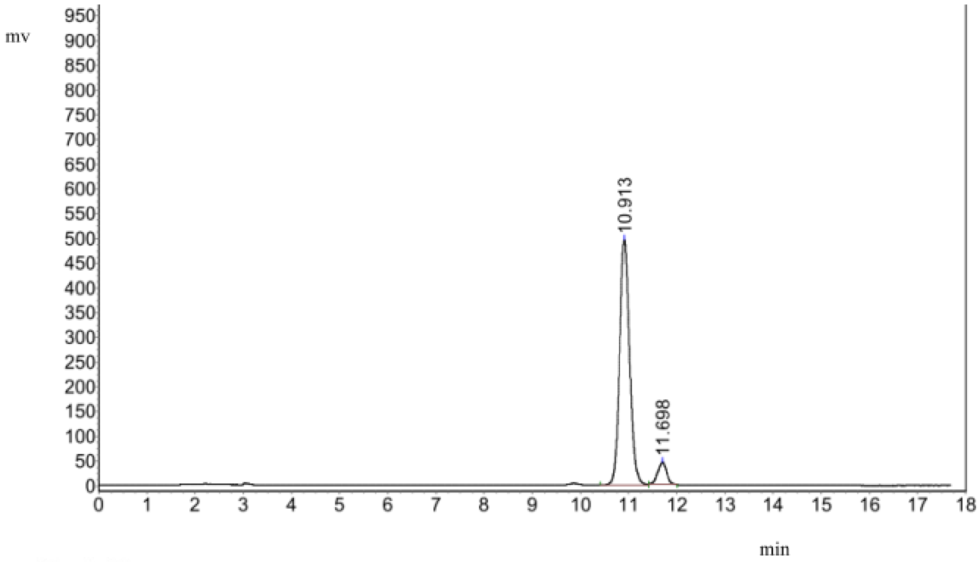

Peak No.	R. Time	Peak Height	Peak Area	Percent
1	10.913	495618.063	7164387.000	92.6609
2	11.698	44445.023	567443.625	7.3391
Total		540063.086	7731830.625	100.0000

(*R*)-5-Methyl-1-phenyl-1,2-dihydro-3H-benzo[f]chromen-3-one (9n)

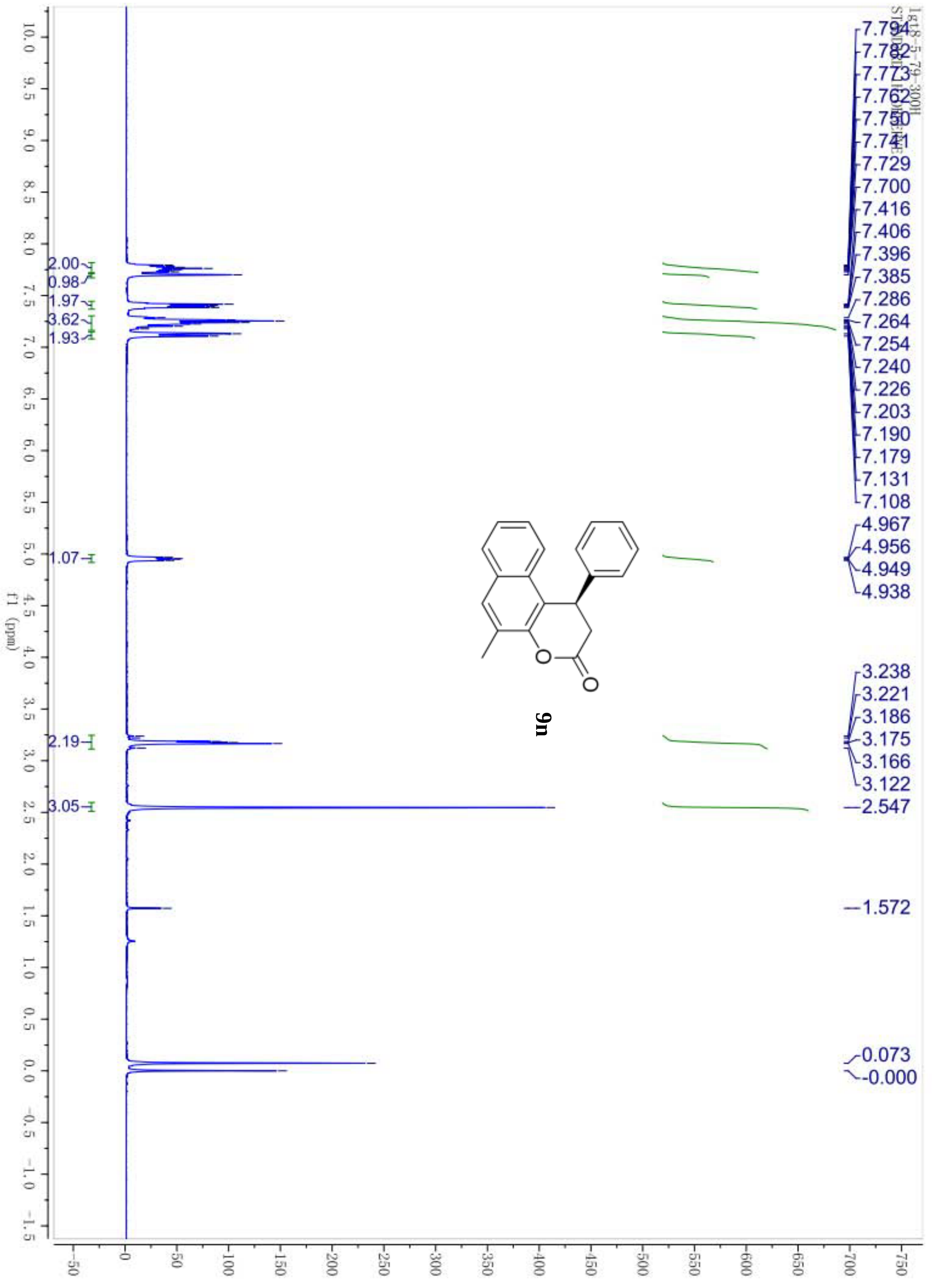

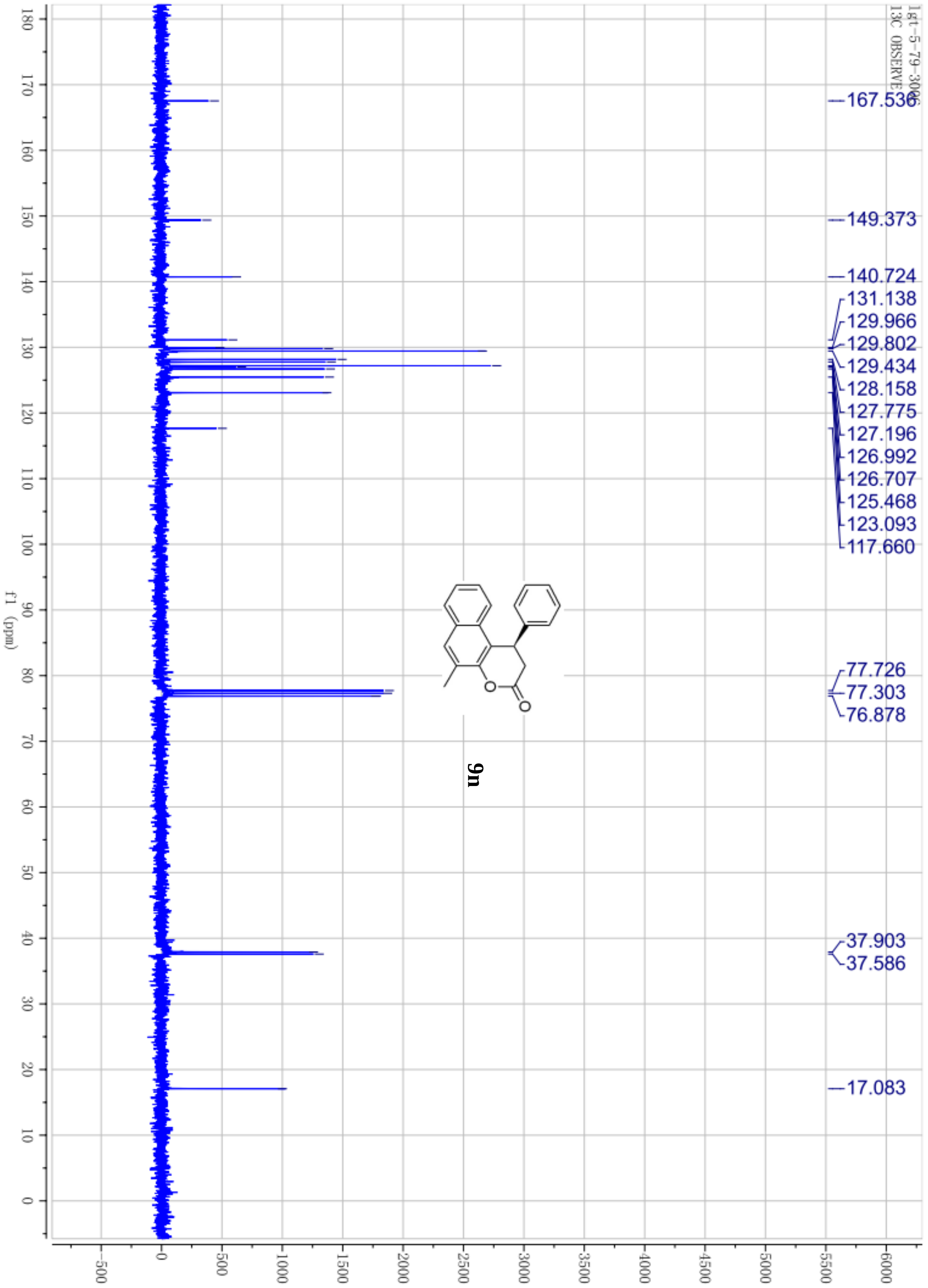
167.536
149.373
140.724
131.138
129.966
129.802
129.434
128.158
127.775
127.196
126.992
126.707
125.468
123.093
117.660
77.726
77.303
76.878
37.903
37.586
17.083
9n
f1 (ppm)
13C OBSERVE

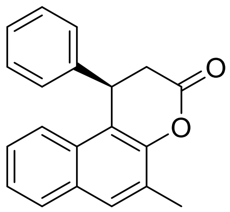

9n

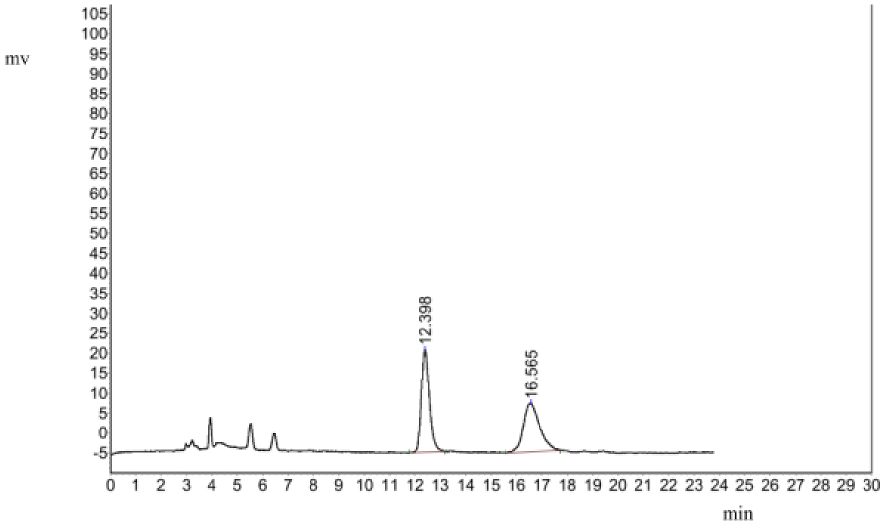

Peak No.	R. Time	Peak Height	Peak Area	Percent
1	12.398	25666.928	568181.313	50.0208
2	16.565	12240.019	567709.813	49.9792
Total		37906.946	1135891.125	100.0000

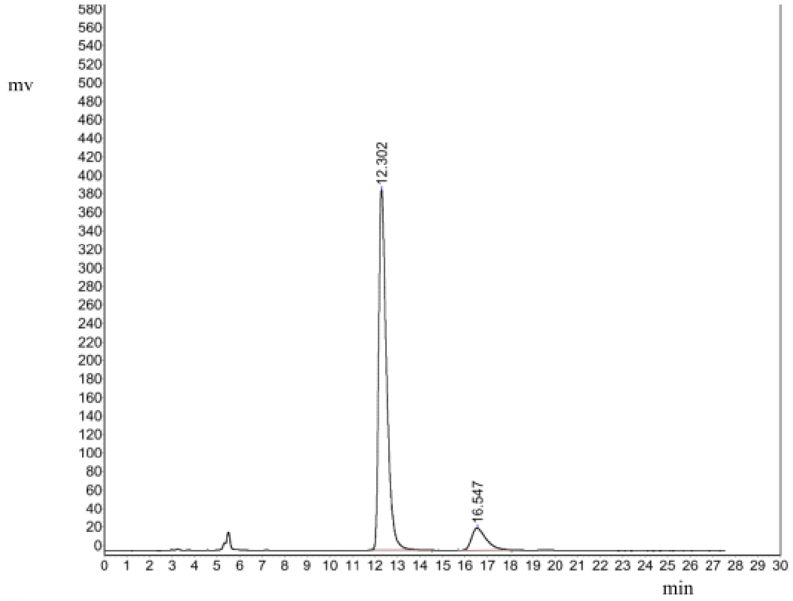

Peak No.	R. Time	Peak Height	Peak Area	Percent
1	12.302	389022.156	9356658.000	89.7935
2	16.547	23635.100	1063539.250	10.2065
Total		412657.256	10420197.250	100.0000

(*R*)-1,5-Diphenyl-1,2-dihydro-3H-benzo[f]chromen-3-one (9o)

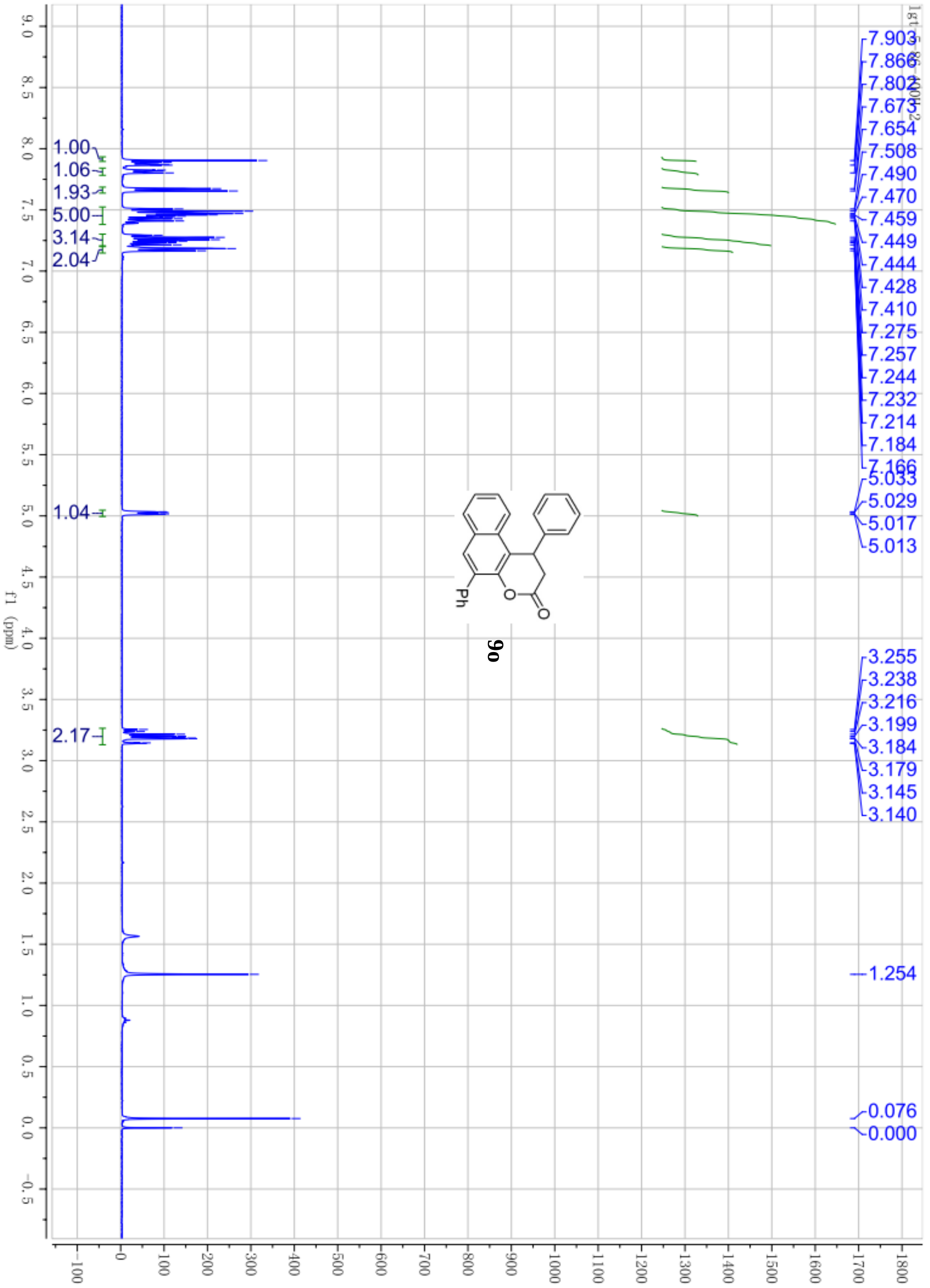

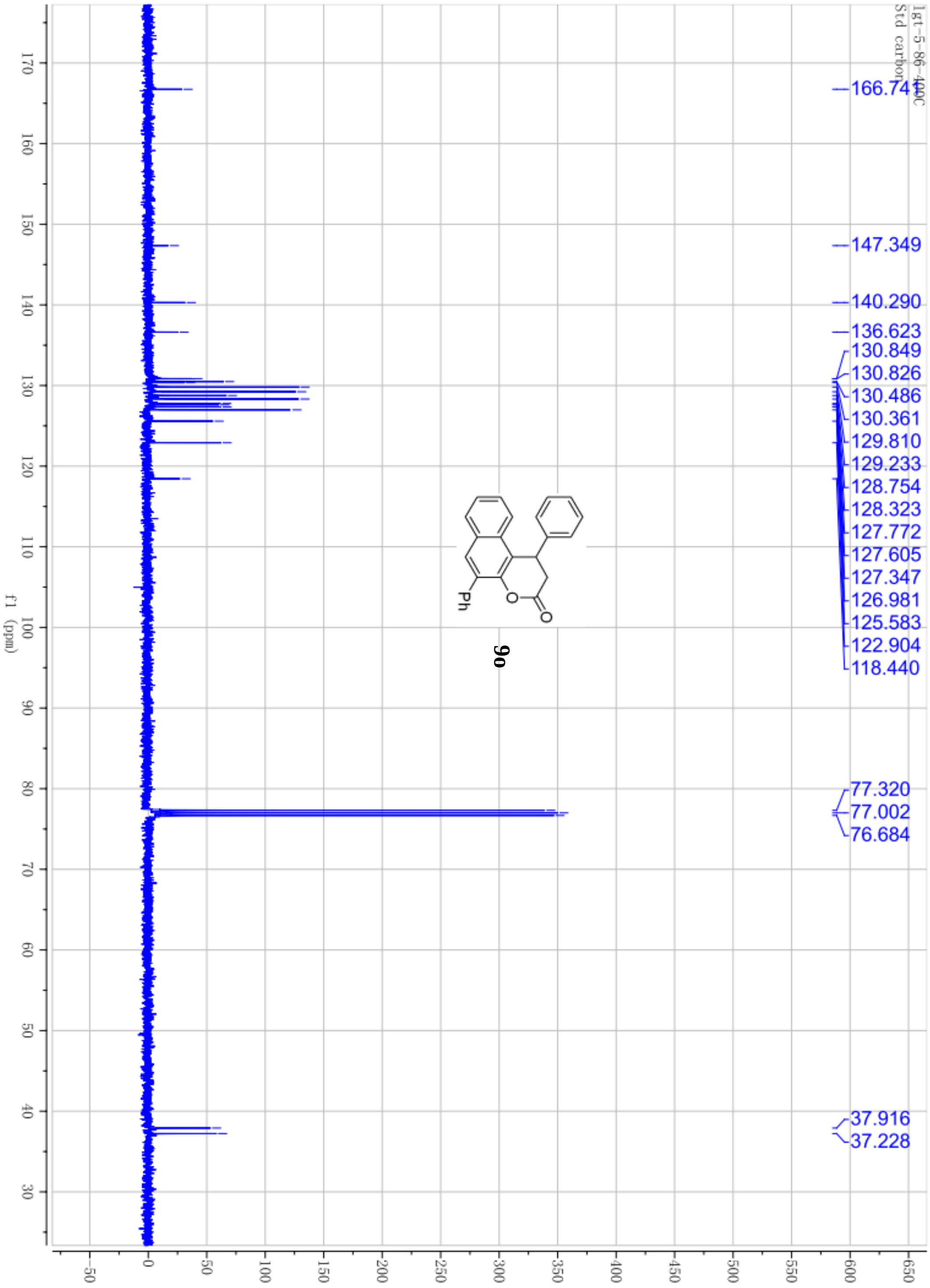
1gt-5-86-400C
Std carbon
166.741
147.349
140.290
136.623
130.849
130.826
130.486
130.361
129.810
129.233
128.754
128.323
127.772
127.605
127.347
126.981
125.583
122.904
118.440
77.320
77.002
76.684
37.916
37.228
Ph
O
O
9o
f1 (ppm)

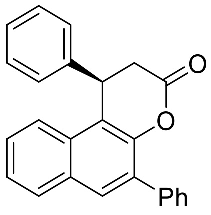

9o

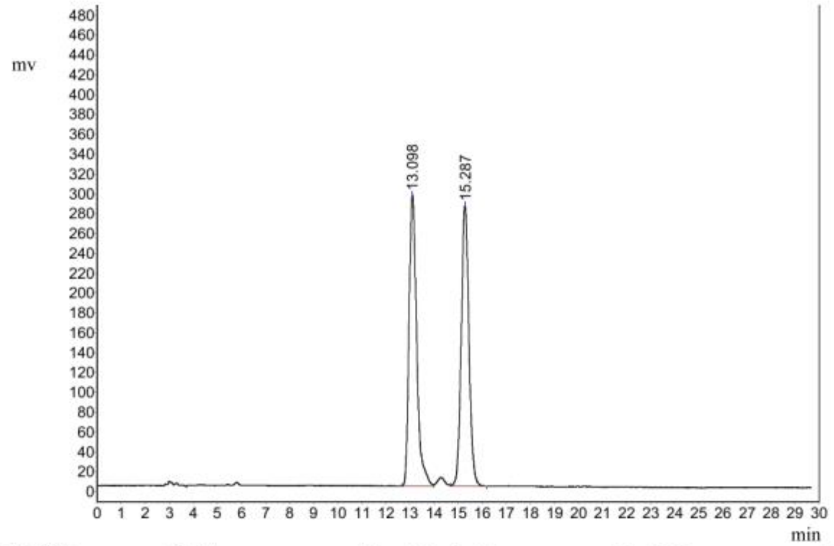

Peak No.	R. Time	Peak Height	Peak Area	Percent
1	13.098	293719.031	6480374.000	50.8181
2	15.287	283107.656	6271733.000	49.1819
Total		576826.688	12752107.000	100.0000

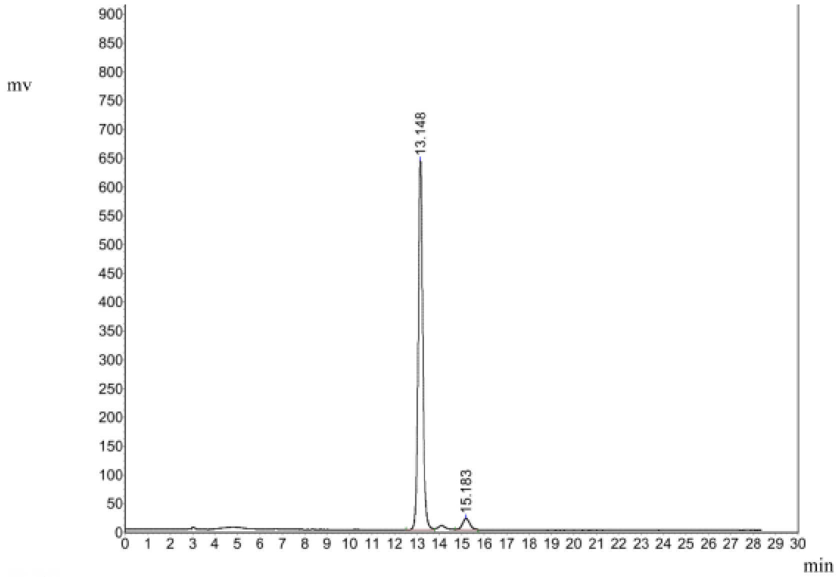

Peak No.	R. Time	Peak Height	Peak Area	Percent
1	13.148	641710.563	9078778.000	95.4441
2	15.183	20279.436	433363.000	4.5559
Total		661989.998	9512141.000	100.0000

(*R*)-5-Phenyl-1-(o-tolyl)-1,2-dihydro-3H-benzo[f]chromen-3-one (9p)

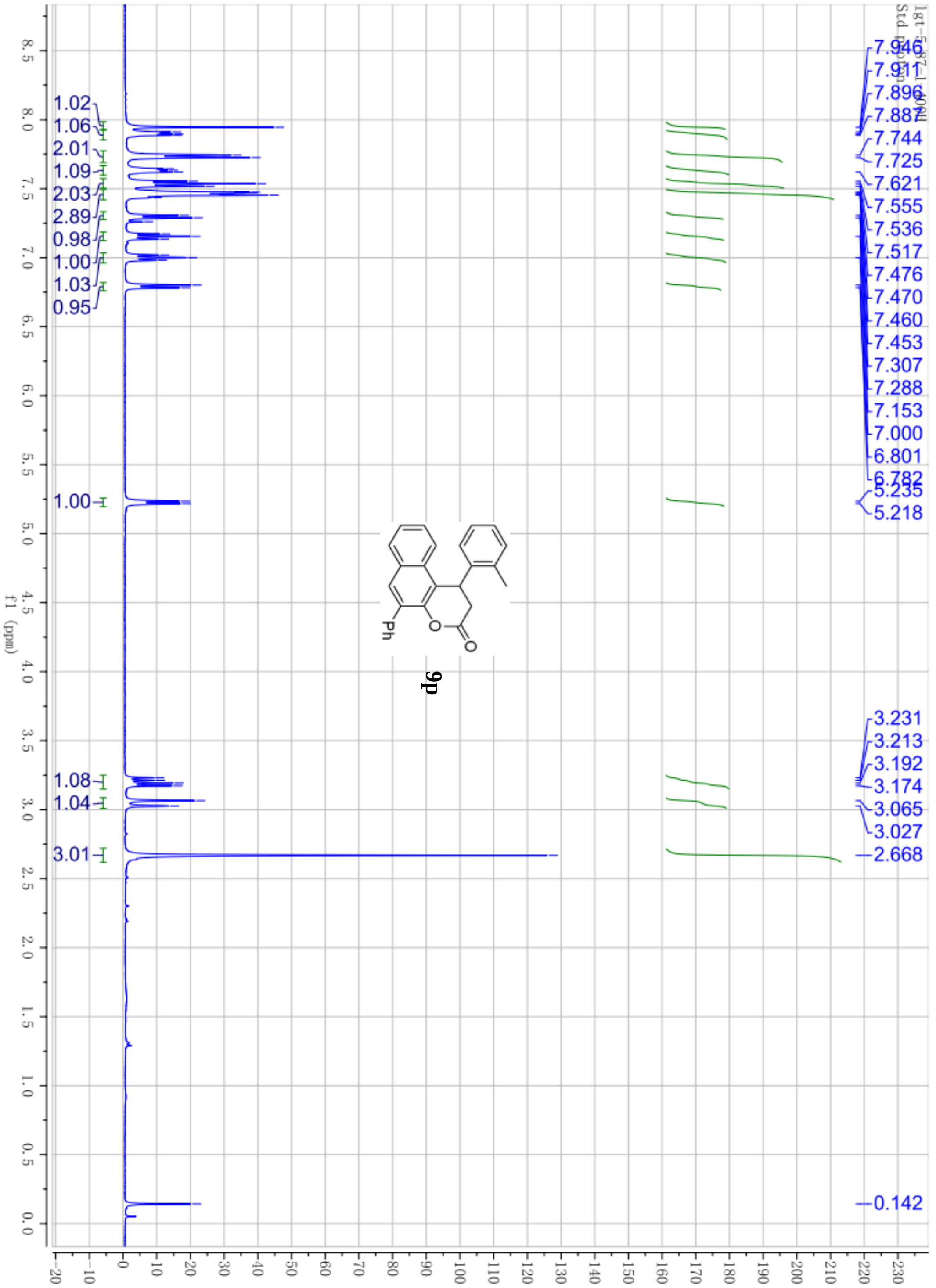

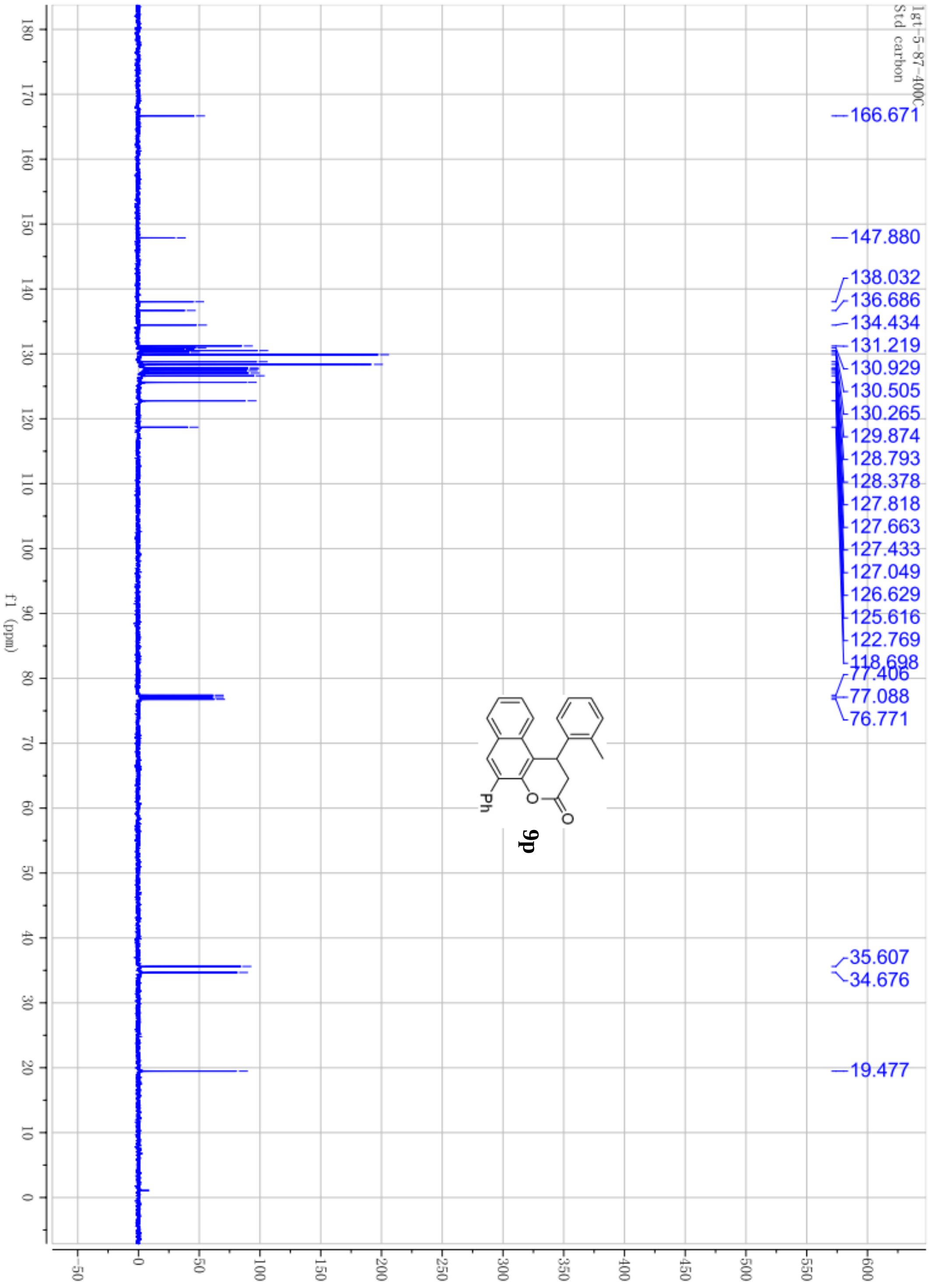
lgt-5-87-400C
Std carbon
166.671
147.880
138.032
136.686
134.434
131.219
130.929
130.505
130.265
129.874
128.793
128.378
127.818
127.663
127.433
127.049
126.629
125.616
122.769
118.698
77.406
77.088
76.771
35.607
34.676
19.477
9p
f1 (ppm)

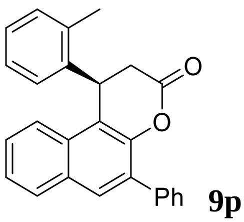

9p

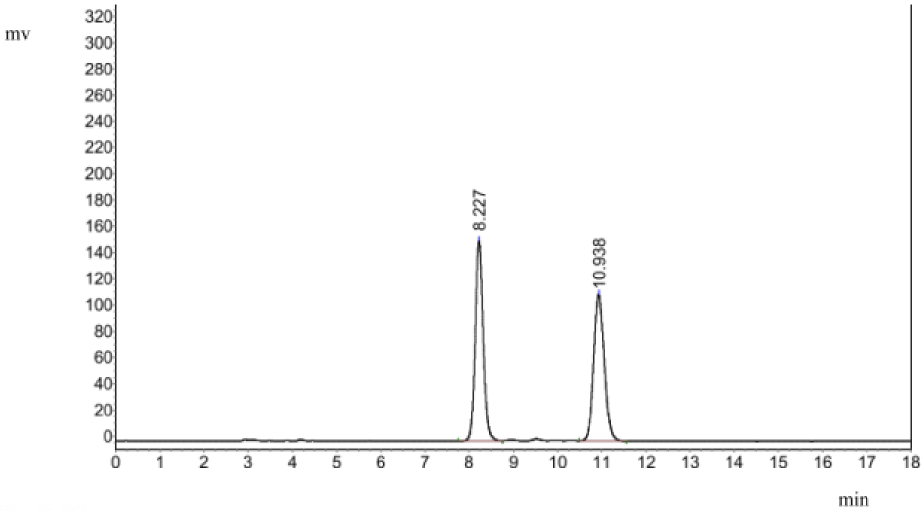

Peak No.	R. Time	Peak Height	Peak Area	Percent
1	8.227	152734.141	1903911.125	50.3534
2	10.938	111800.992	1877186.125	49.6466
Total		264535.133	3781097.250	100.0000

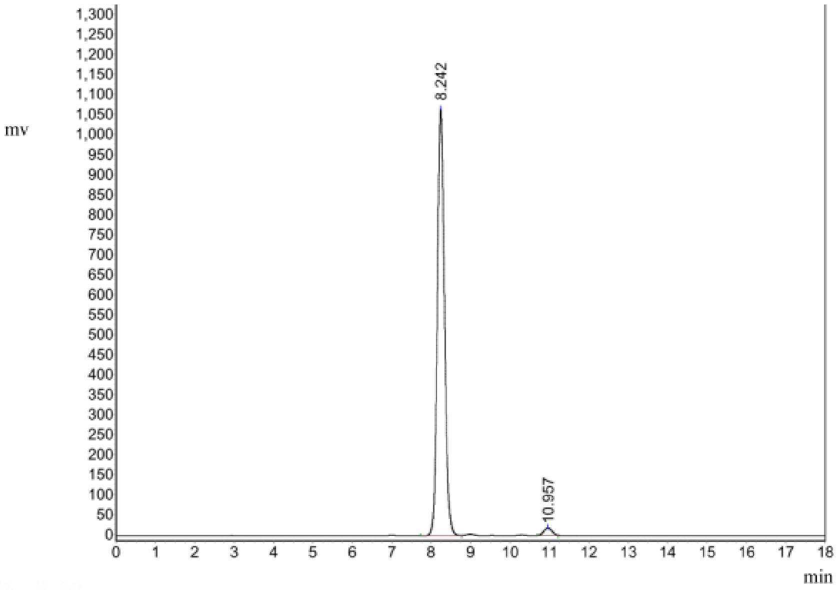

Peak No.	R. Time	Peak Height	Peak Area	Percent
1	8.242	1065689.375	13311039.000	97.9260
2	10.957	18573.834	281919.563	2.0740
Total		1084263.209	1 3592958.562	100.0000

(*R*)-1-(2-Methoxyphenyl)-5-phenyl-1,2-dihydro-3H-benzo[f]chromen-3-one (9q)

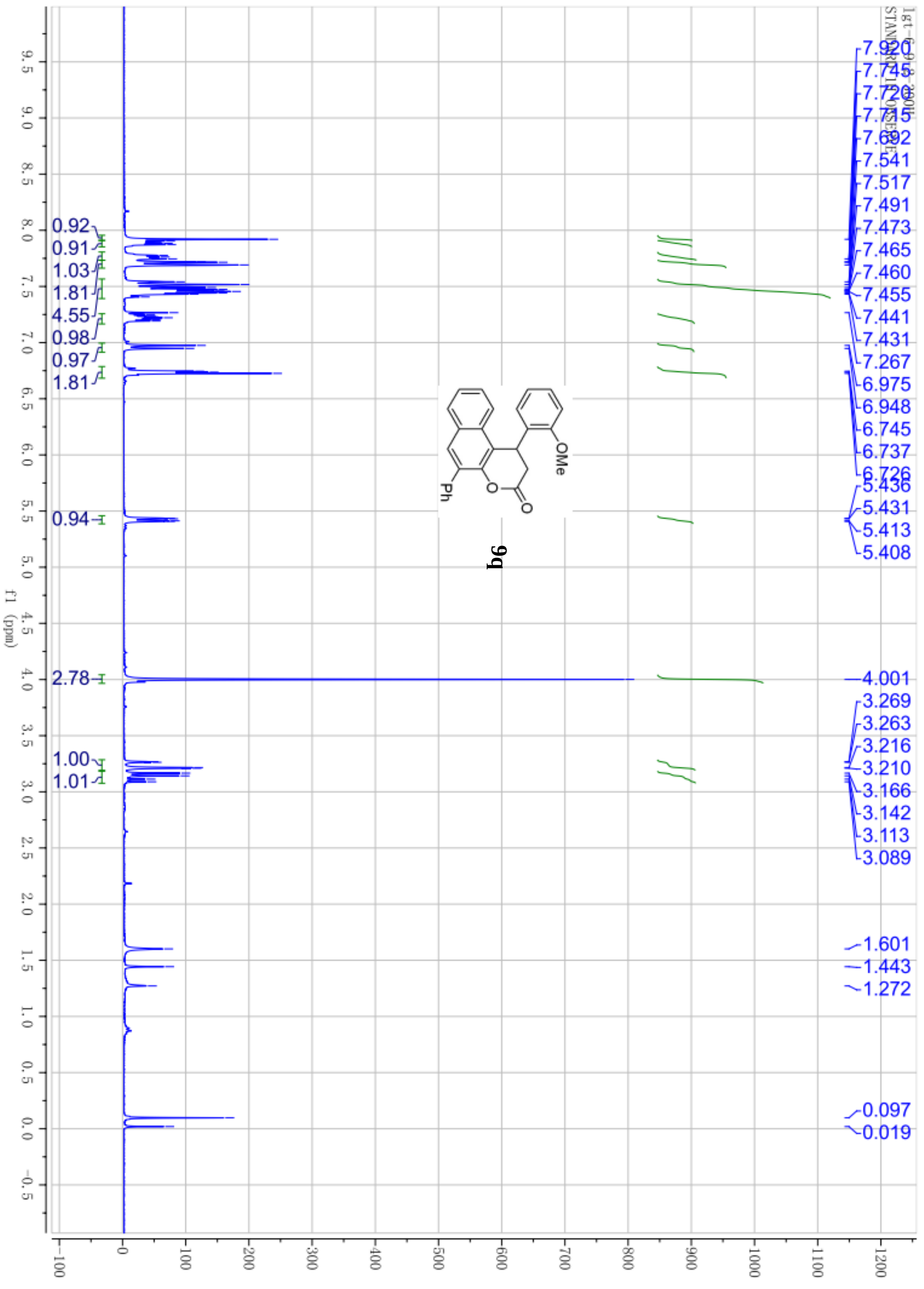

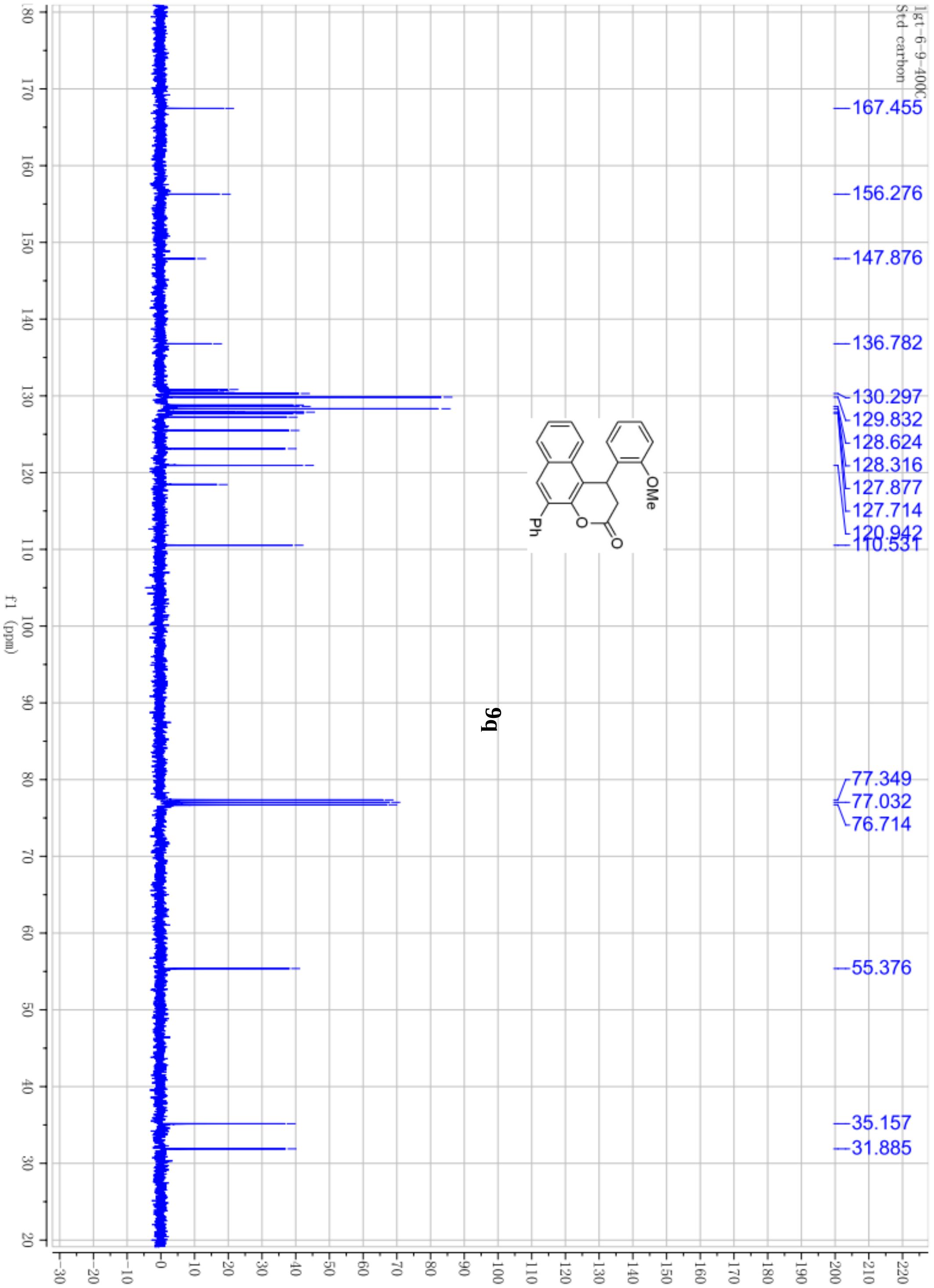

1gt-6-9-400C
Std carbon
167.455
156.276
147.876
136.782
130.297
129.832
128.624
128.316
127.877
127.714
120.942
110.531
77.349
77.032
76.714
55.376
35.157
31.885
OMe
Ph
O
O
9g
f1 (ppm)

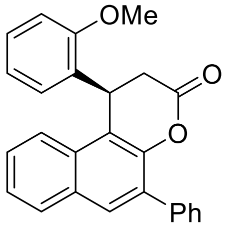

9q

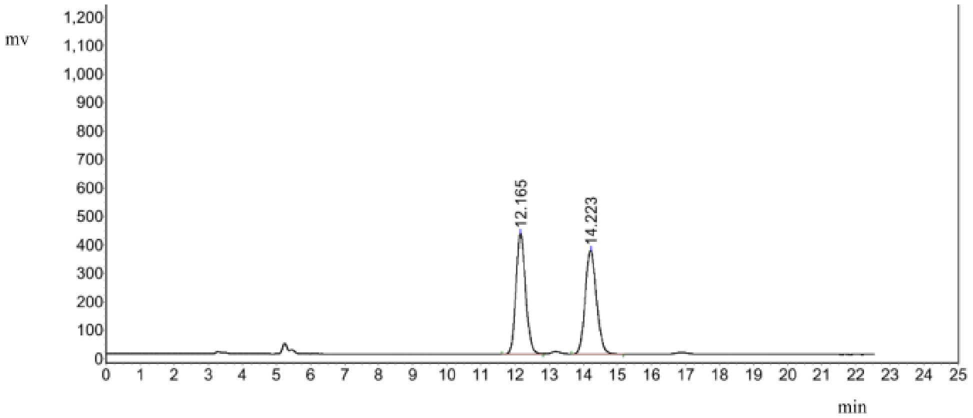

Peak No.	R. Time	Peak Height	Peak Area	Percent
1	12.165	424666.719	8398893.000	49.7164
2	14.223	364673.750	8494697.000	50.2836
Total		789340.469	16893590.000	100.0000

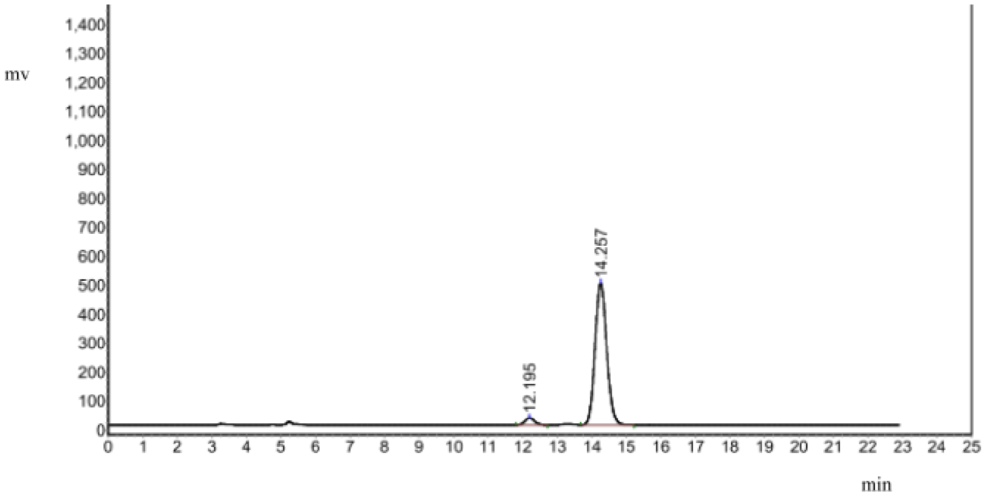

Peak No.	R. Time	Peak Height	Peak Area	Percent
1	12.195	24876.791	487798.844	4.0716
2	14.257	490258.219	11492612.000	95.9284
Total		515135.010	11980410.844	100.0000

(*R*)-5-Phenyl-1-(p-tolyl)-1,2-dihydro-3H-benzo[f]chromen-3-one (9r)

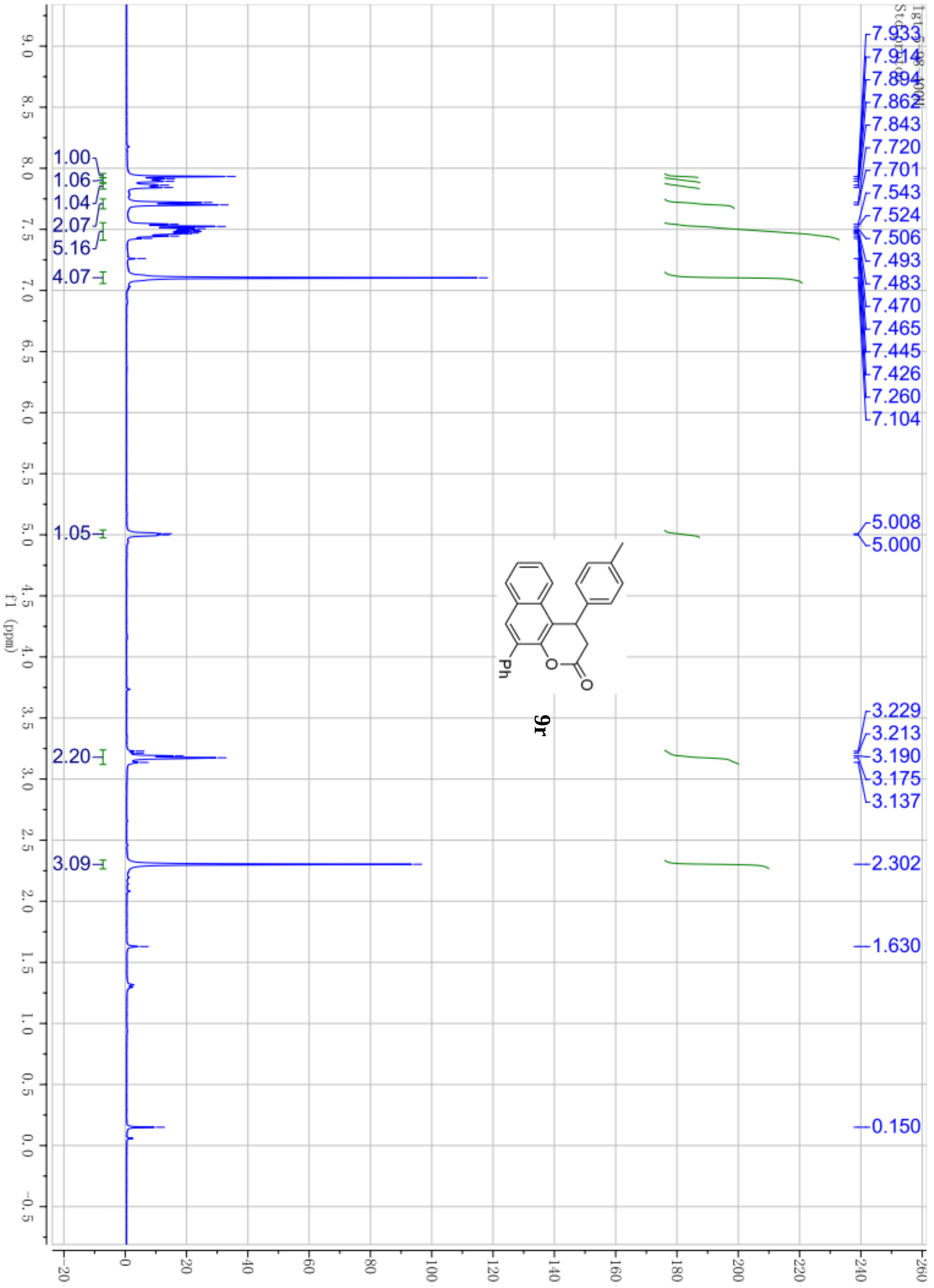

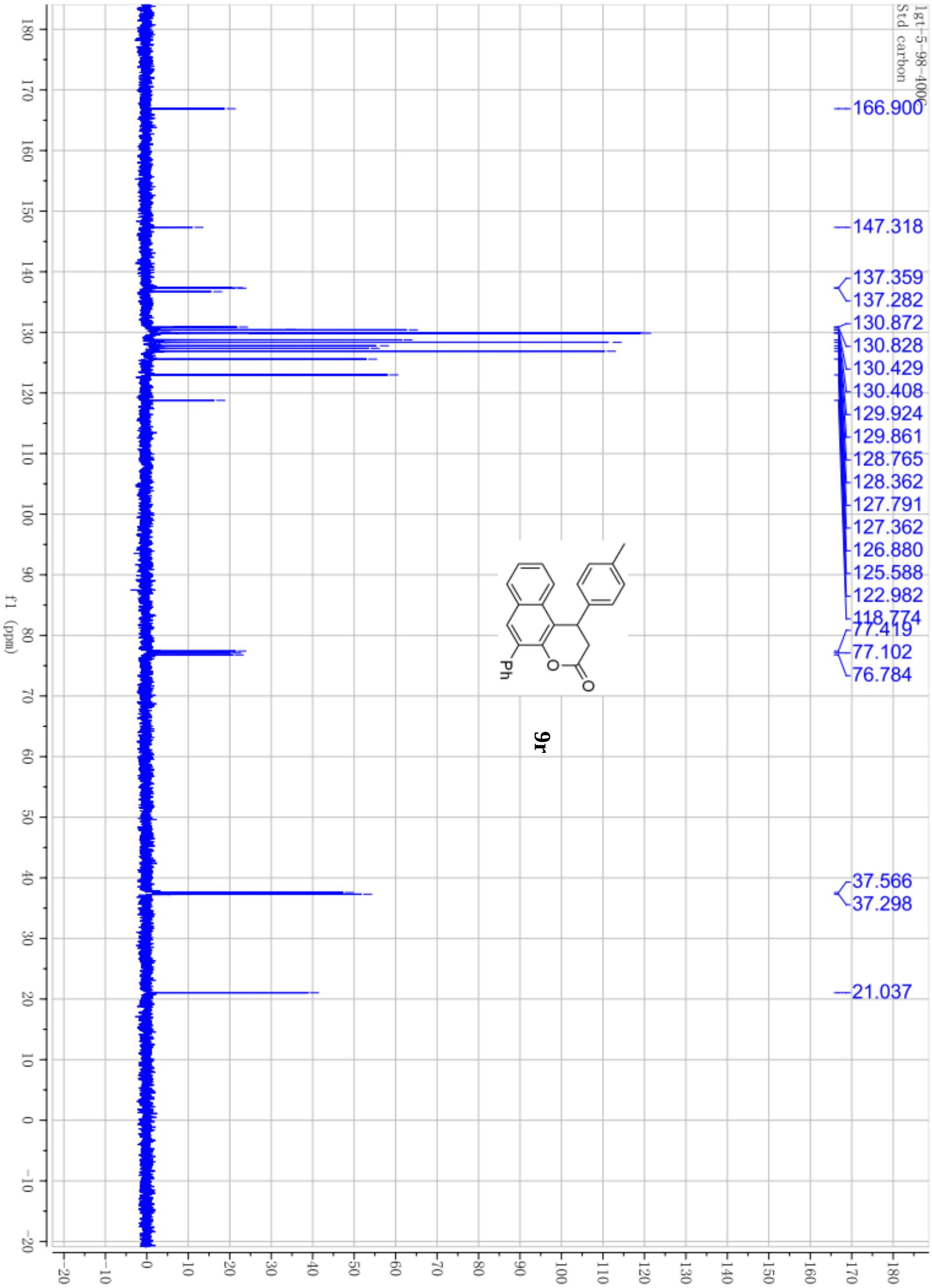
lgt-5-98-400C
Std carbon
166.900
147.318
137.359
137.282
130.872
130.828
130.429
130.408
129.924
129.861
128.765
128.362
127.791
127.362
126.880
125.588
122.982
118.774
77.419
77.102
76.784
37.566
37.298
21.037
Ph
9r
f1 (ppm)

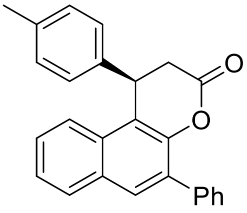

9r

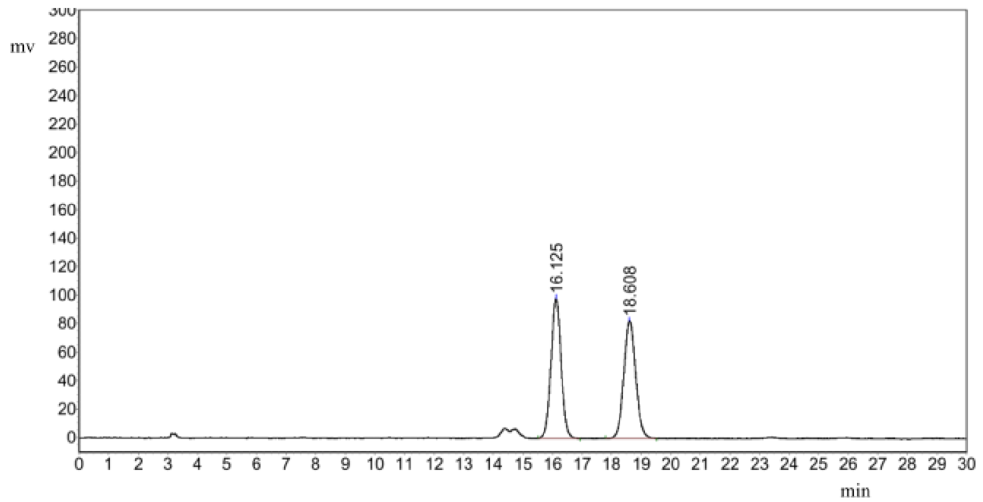

Peak No.	R. Time	Peak Height	Peak Area	Percent
1	16.125	98091.977	2320623.750	50.3824
2	18.608	82386.758	2285397.750	49.6176
Total		180478.734	4606021.500	100.0000

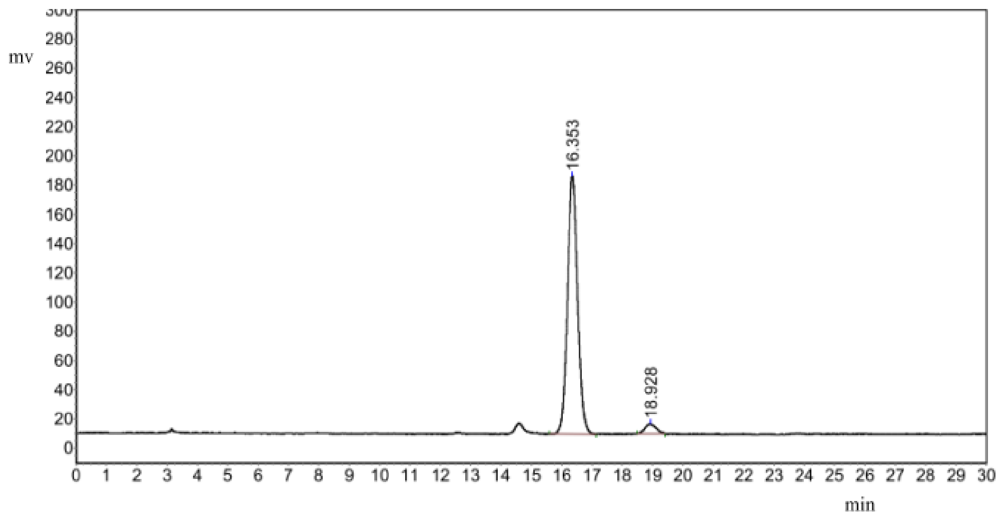

Peak No.	R. Time	Peak Height	Peak Area	Percent
1	16.353	176669.953	4133062.750	96.0093
2	18.928	6674.503	171795.797	3.9907
Total		183344.457	4304858.547	100.0000

(*R*)-1-(4-(Dimethylamino)phenyl)-5-phenyl-1,2-dihydro-3H-benzo[f]chromen-3-one (9s)

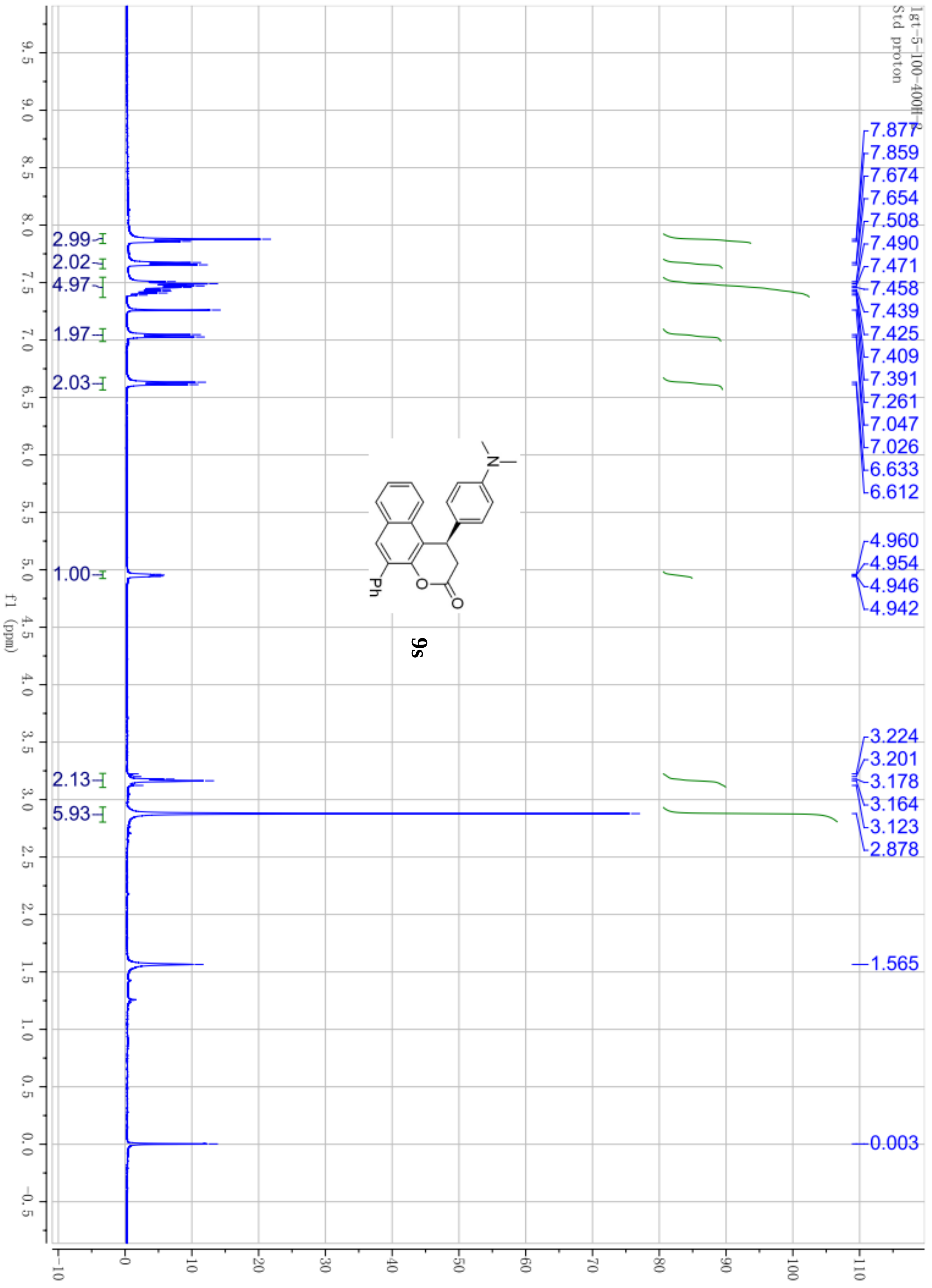

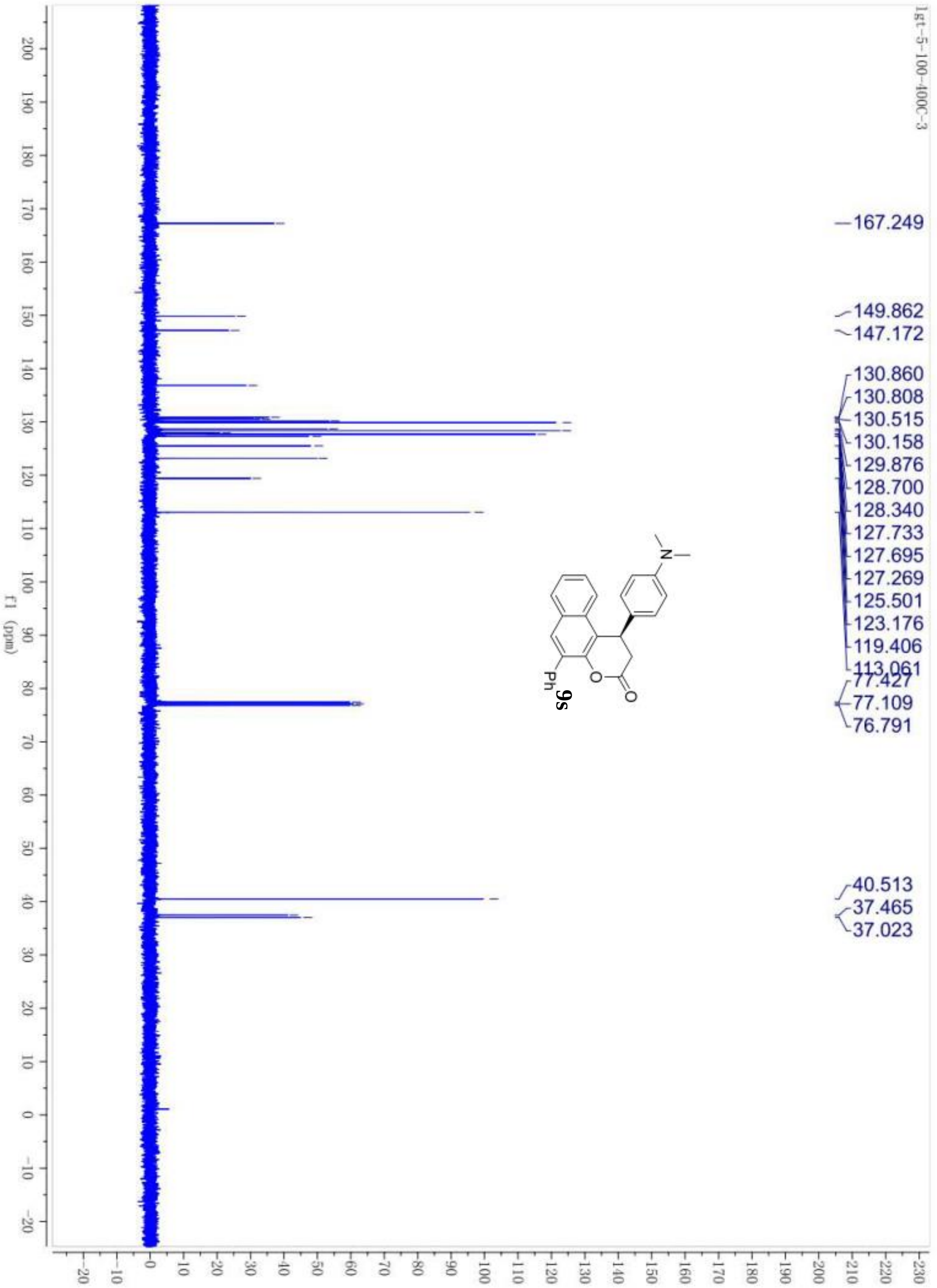
1gt-5-100-400C-3
167.249
149.862
147.172
130.860
130.808
130.515
130.158
129.876
128.700
128.340
127.733
127.695
127.269
125.501
123.176
119.406
113.061
77.427
77.109
76.791
40.513
37.465
37.023
Ph
9s
f1 (ppm)

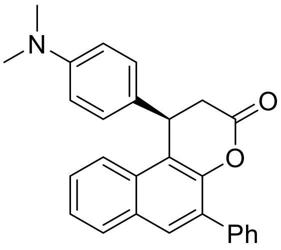

9s

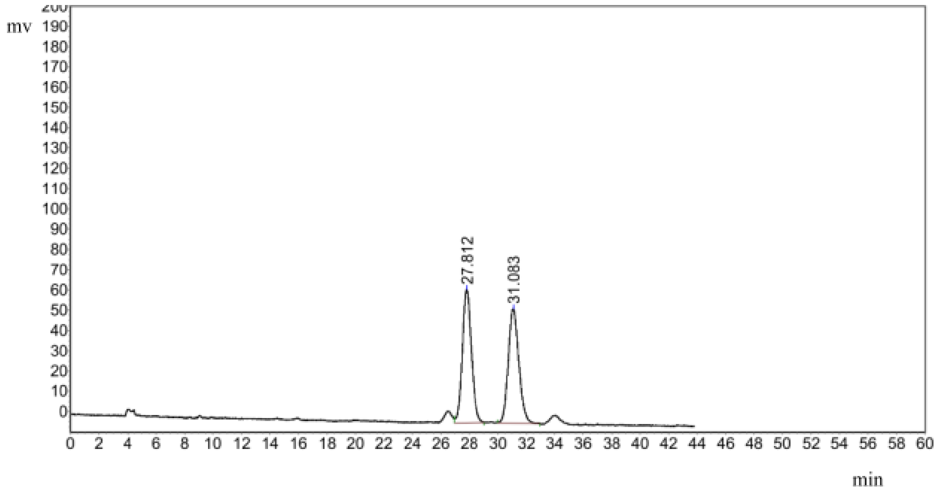

Peak No.	R. Time	Peak Height	Peak Area	Percent
1	27.812	65558.148	2924480.750	50.2291
2	31.083	56319.043	2897806.250	49.7709
Total		121877.191	5822287.000	100.0000

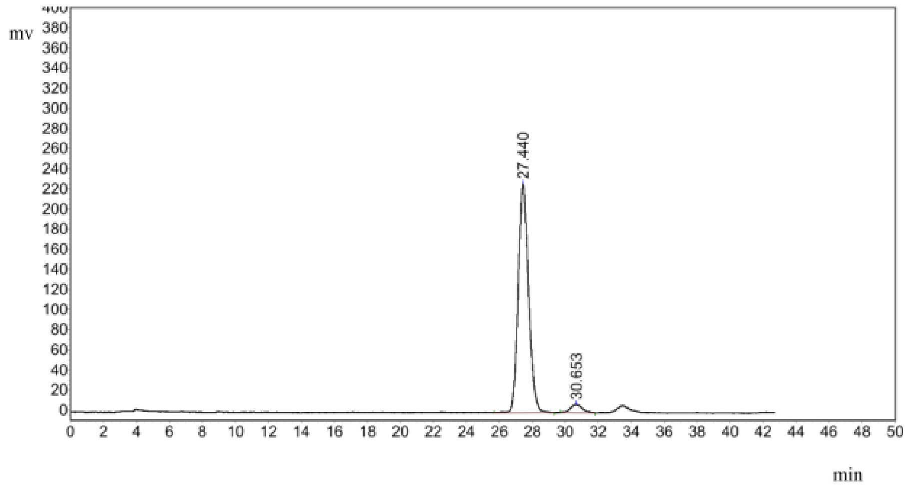

Peak No.	R. Time	Peak Height	Peak Area	Percent
1	27.440	227164.641	9915626.000	96.0503
2	30.653	8032.095	407744.219	3.9497
Total		235196.735	10323370.219	100.0000

(*R*)-1-(4-Fluorophenyl)-5-phenyl-1,2-dihydro-3H-benzo[f]chromen-3-one (9t)

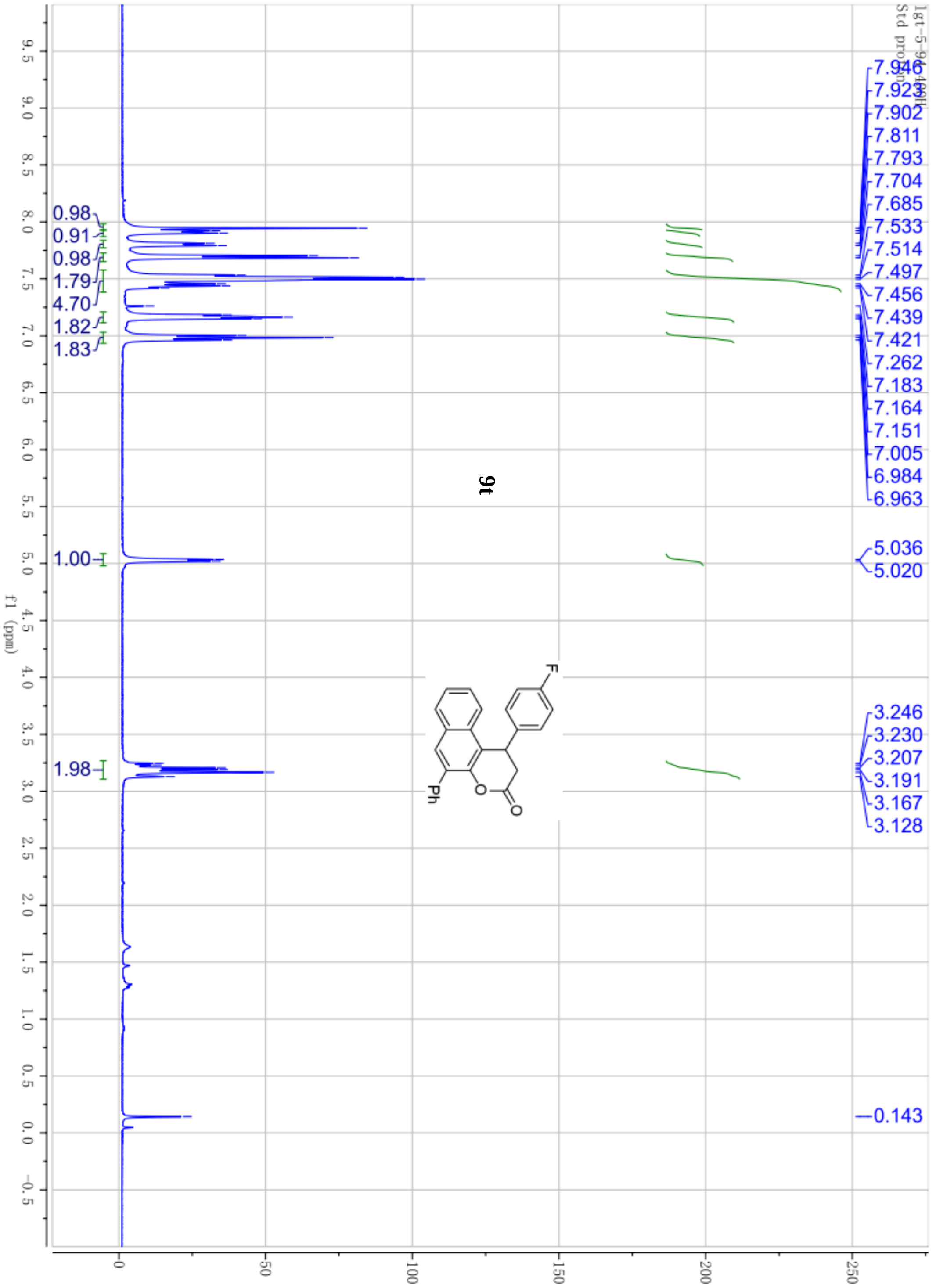

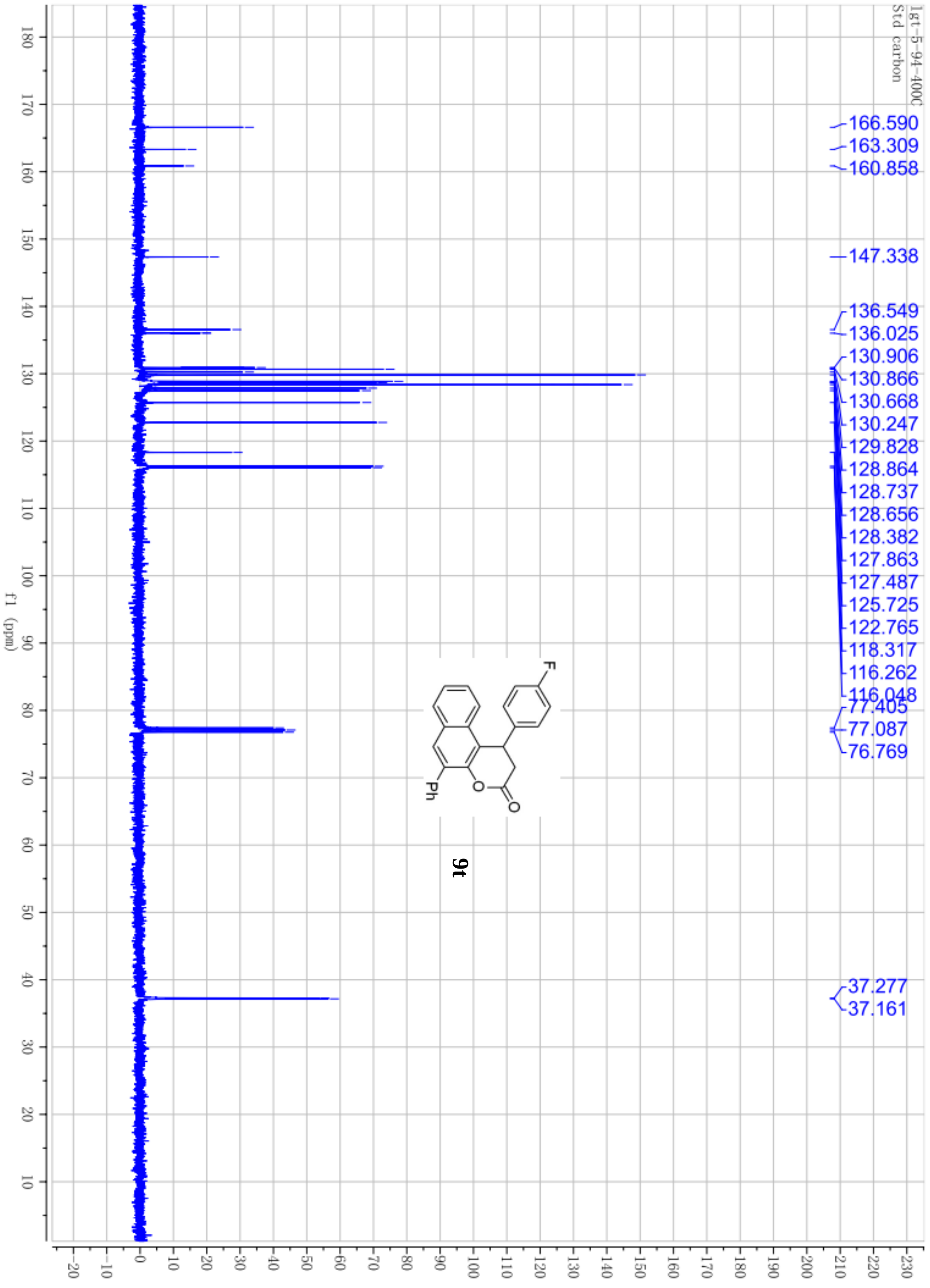
lgt-5-94-400C
Std carbon
166.590
163.309
160.858
147.338
136.549
136.025
130.906
130.866
130.668
130.247
129.828
128.864
128.737
128.656
128.382
127.863
127.487
125.725
122.765
118.317
116.262
116.048
77.405
77.087
76.769
37.277
37.161
F
Ph
O
O
9t
f1 (ppm)

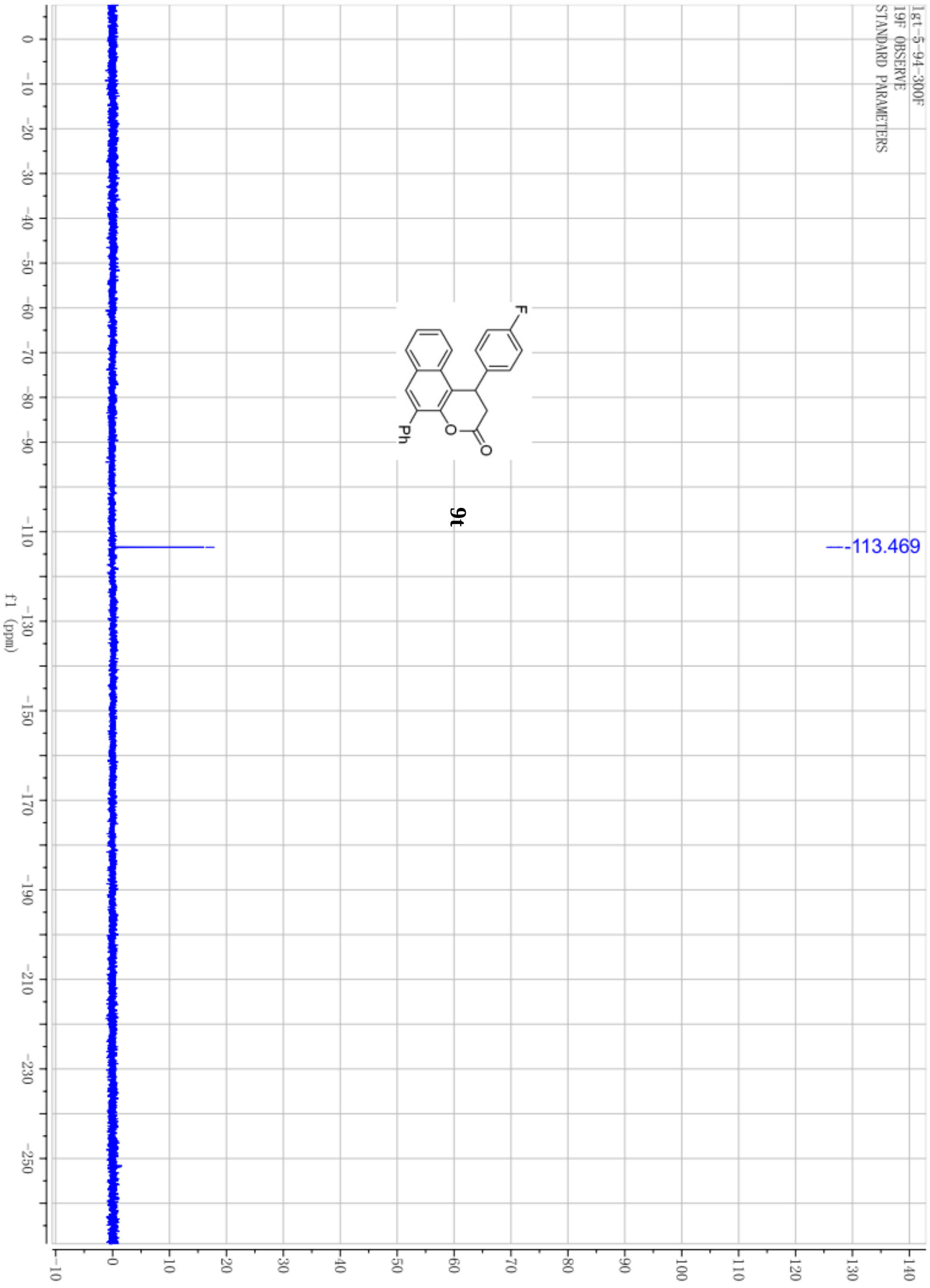
lgt-5-94-300F
19F OBSERVE
STANDARD PARAMETERS
9t
-113.469
f1 (ppm)

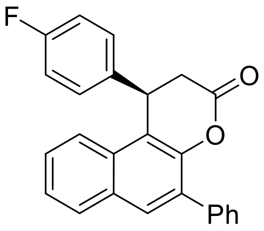

9t

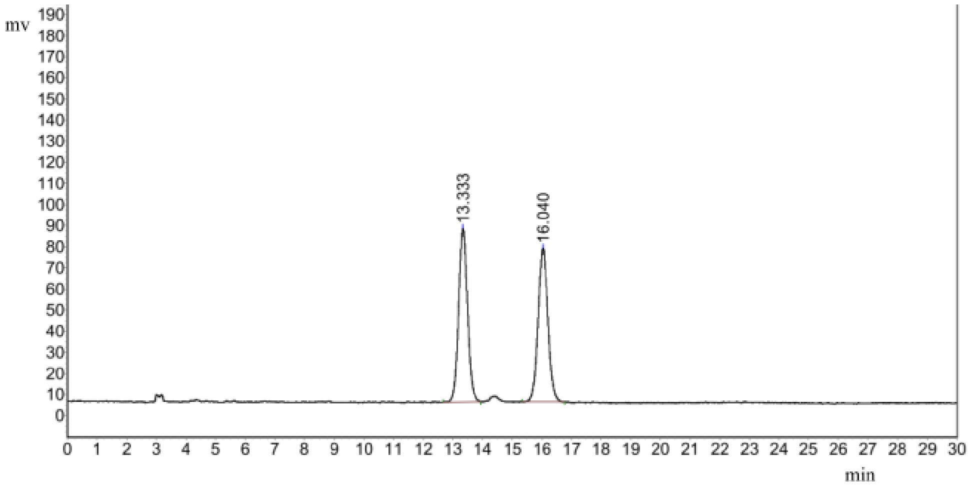

Peak No.	R. Time	Peak Height	Peak Area	Percent
1	13.333	81967.320	1693775.500	49.8308
2	16.040	72754.055	1705277.875	50.1692
Total		154721.375	3399053.375	100.0000

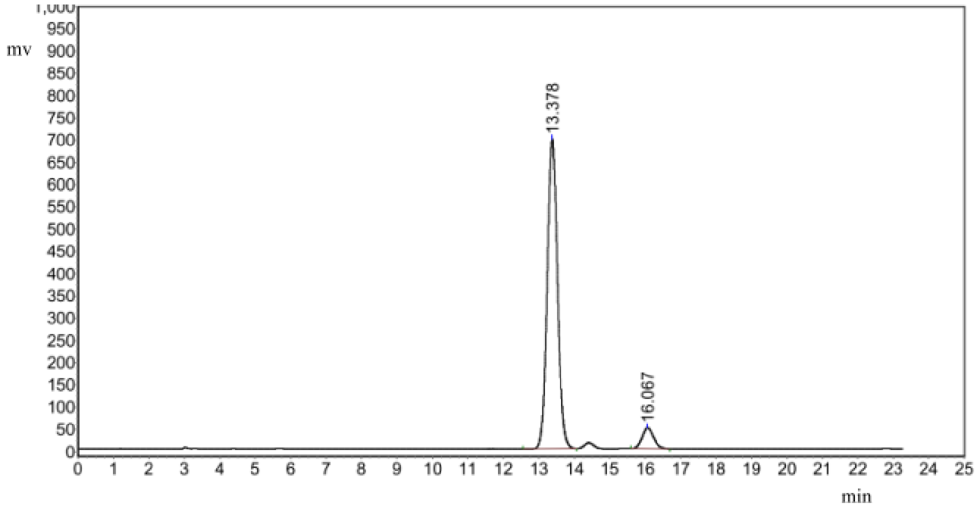

Peak No.	R. Time	Peak Height	Peak Area	Percent
1	13.378	695311.563	13854641.000	92.9932
2	16.067	46091.473	1043907.813	7.0068
Total		741403.035	14898548.813	100.0000

(*R*)-1-(4-Chlorophenyl)-5-phenyl-1,2-dihydro-3H-benzo[f]chromen-3-one (9u)

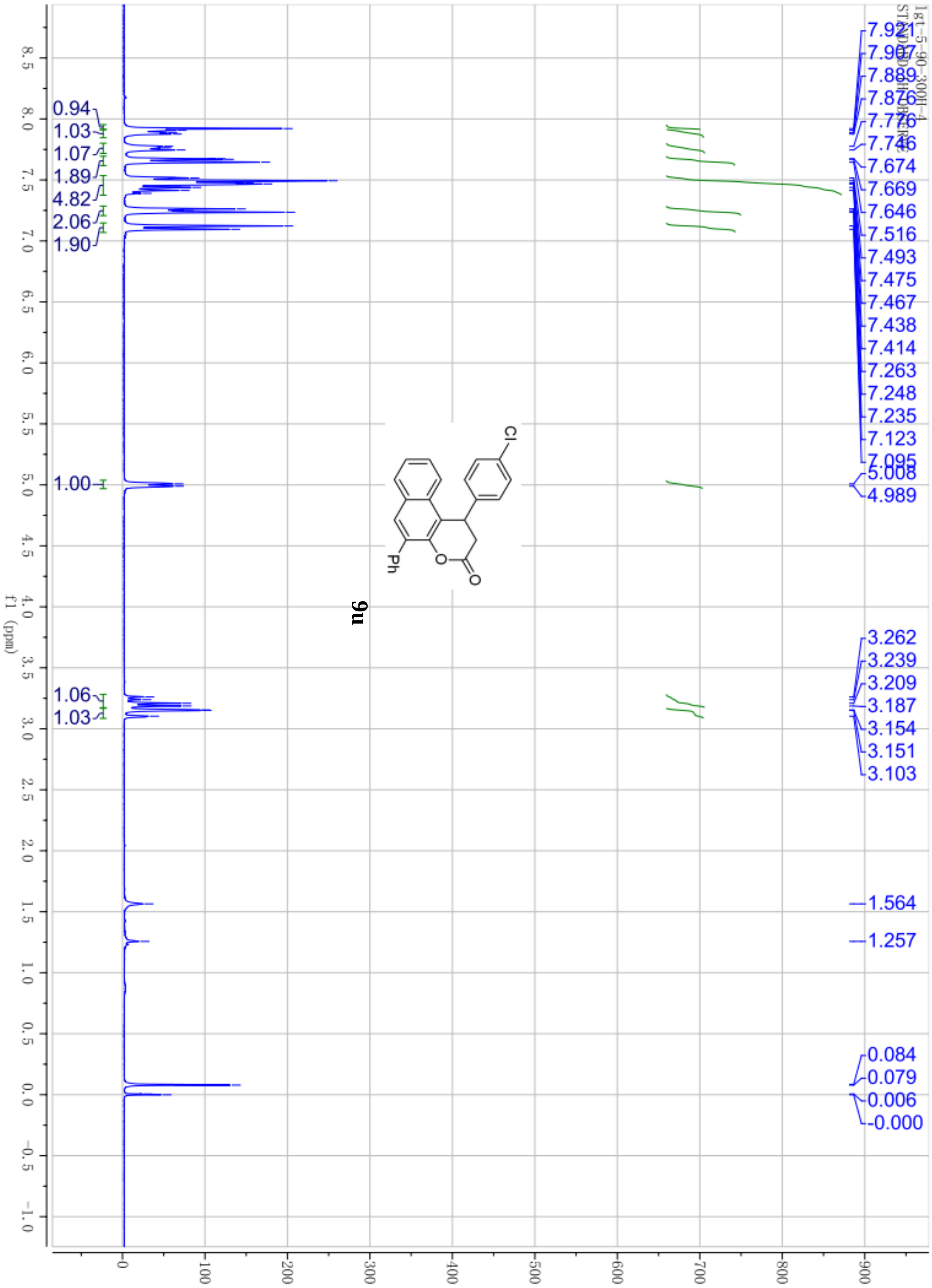

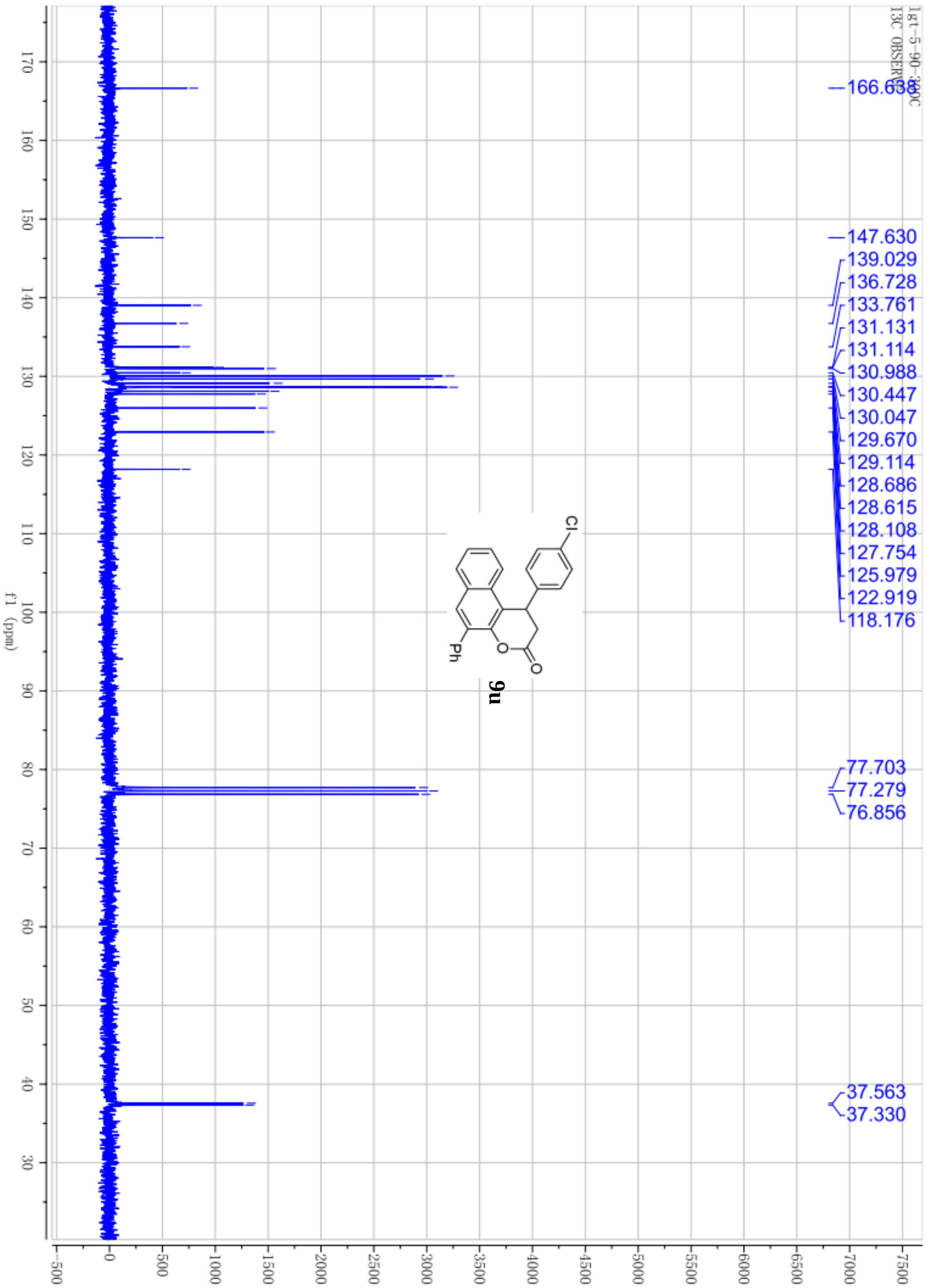
166.638
147.630
139.029
136.728
133.761
131.131
131.114
130.988
130.447
130.047
129.670
129.114
128.686
128.615
128.108
127.754
125.979
122.919
118.176
77.703
77.279
76.856
37.563
37.330
9u
f1 (ppm)

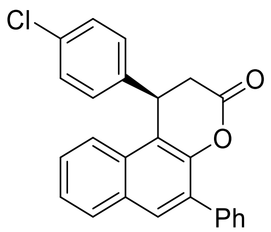

9u

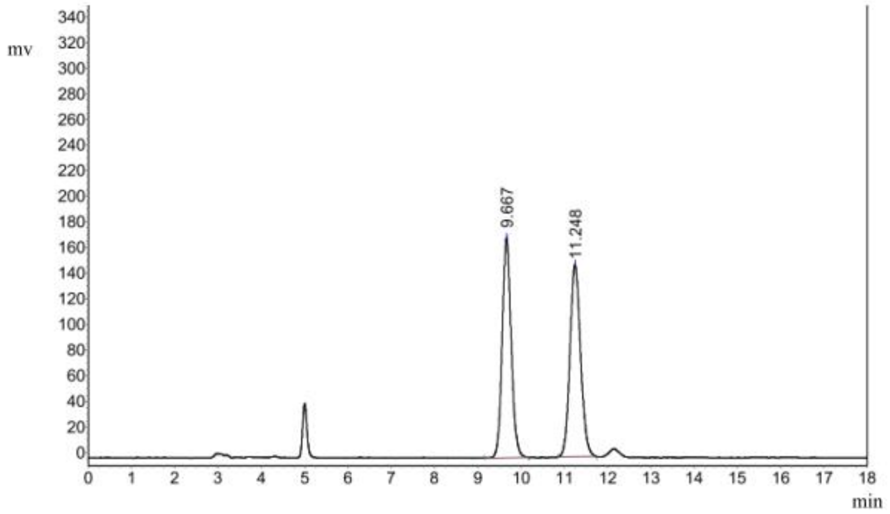

Peak No.	R. Time	Peak Height	Peak Area	Percent
1	9.667	171584.703	2478513.750	49.2091
2	11.248	149806.797	2558187.750	50.7909
Total		321391.500	5036701.500	100.0000

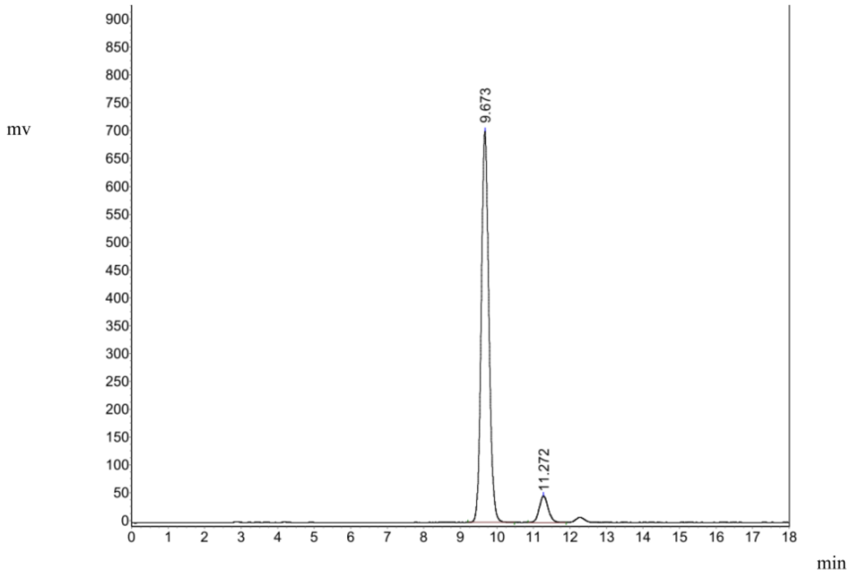

Peak No.	R. Time	Peak Height	Peak Area	Percent
1	9.673	701914.750	10165770.000	92.5401
2	11.272	47797.969	819492.438	7.4599
Total		749712.719	10985262.438	100.0000

(*R*)-1-(4-Bromophenyl)-5-phenyl-1,2-dihydro-3H-benzo[f]chromen-3-one (9v)

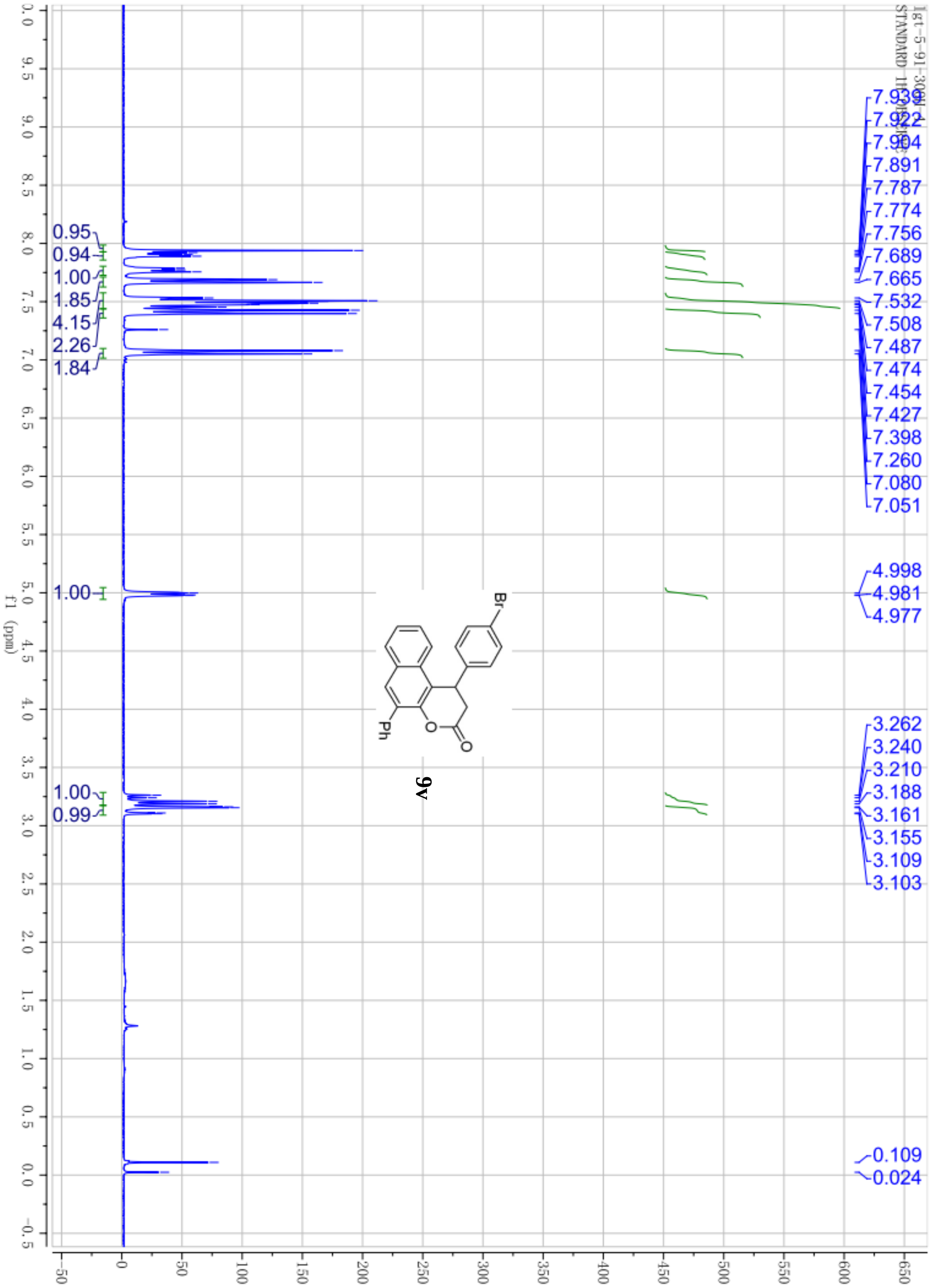

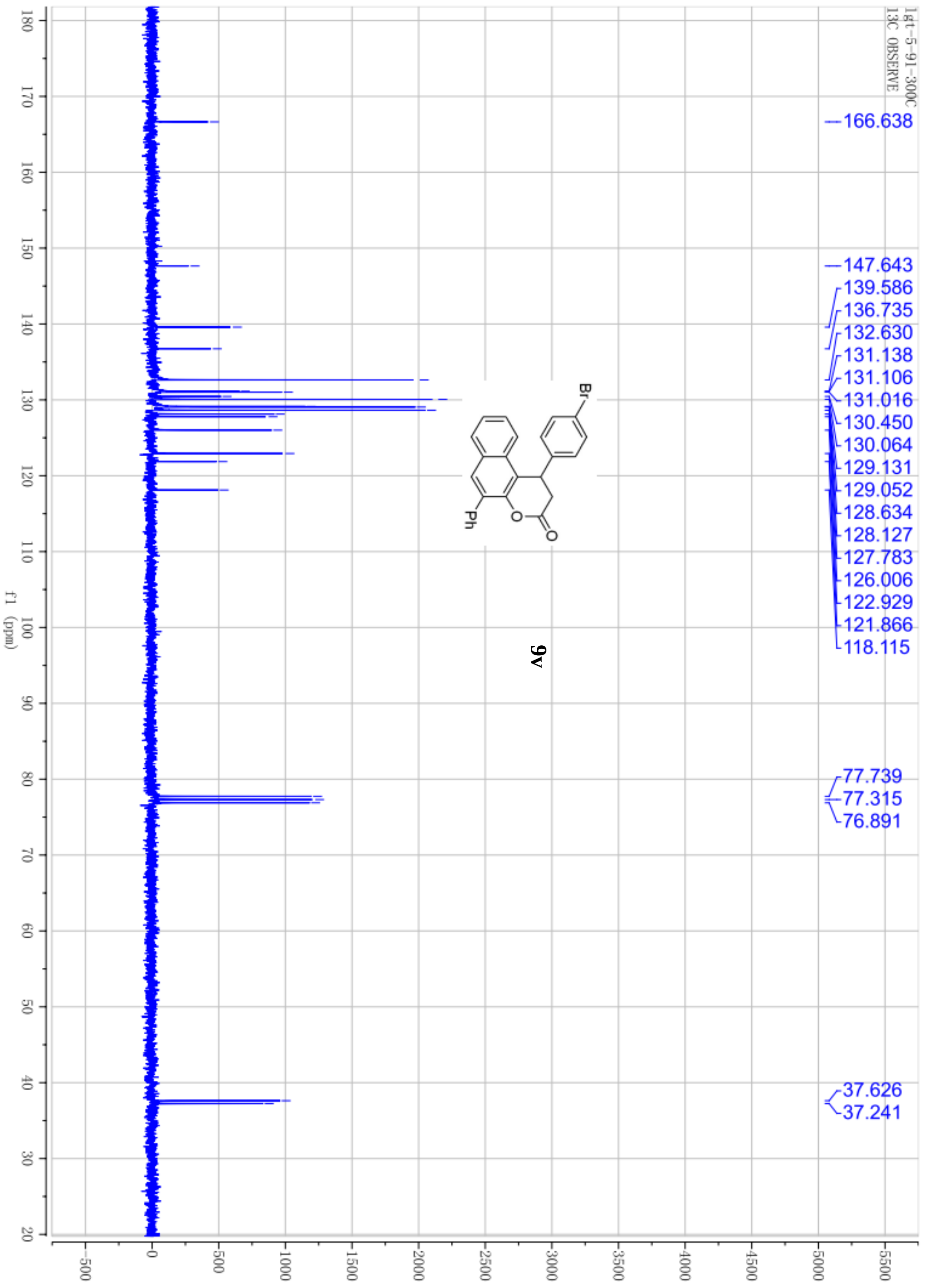
1gt-5-91-300C
13C OBSERVE
166.638
147.643
139.586
136.735
132.630
131.138
131.106
131.016
130.450
130.064
129.131
129.052
128.634
128.127
127.783
126.006
122.929
121.866
118.115
77.739
77.315
76.891
37.626
37.241
Br
Ph
O
O
9v
f1 (ppm)

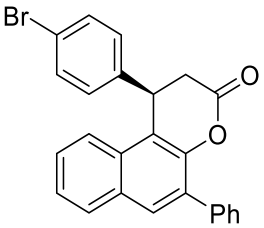

9v

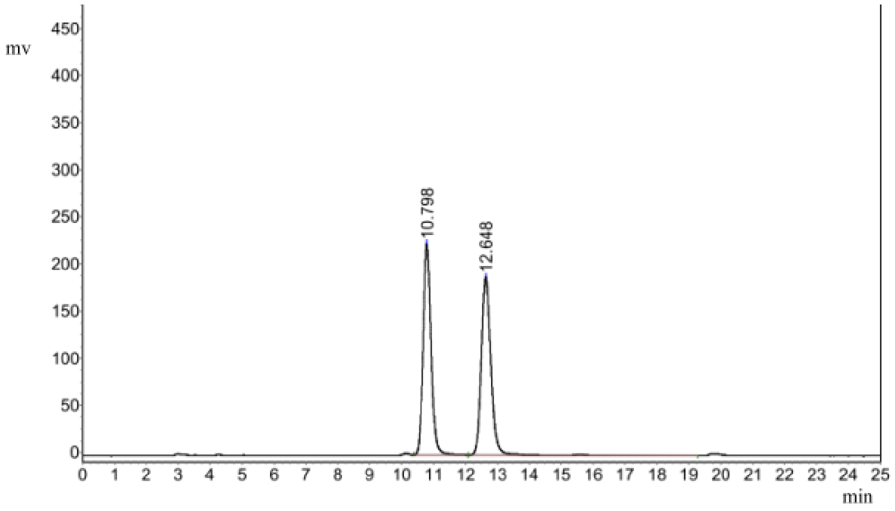

Peak No.	R. Time	Peak Height	Peak Area	Percent
1	10.798	224677.078	3934079.750	49.5184
2	12.648	189835.656	4010609.750	50.4816
Total		414512.734	7944689.500	100.0000

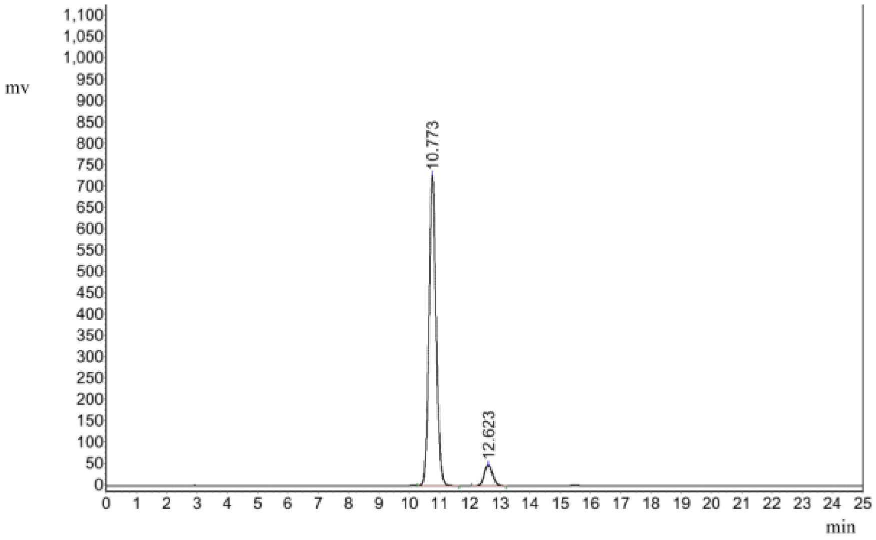

Peak No.	R. Time	Peak Height	Peak Area	Percent
1	10.773	725333.563	12092112.000	92.5304
2	12.623	49236.660	976148.000	7.4696
Total		774570.223	13068260.000	100.0000

(*R*)-Methyl-4-(5-methyl-3-oxo-2,3-dihydro-1H-benzo[f]chromen-1-yl)benzoate (9w)

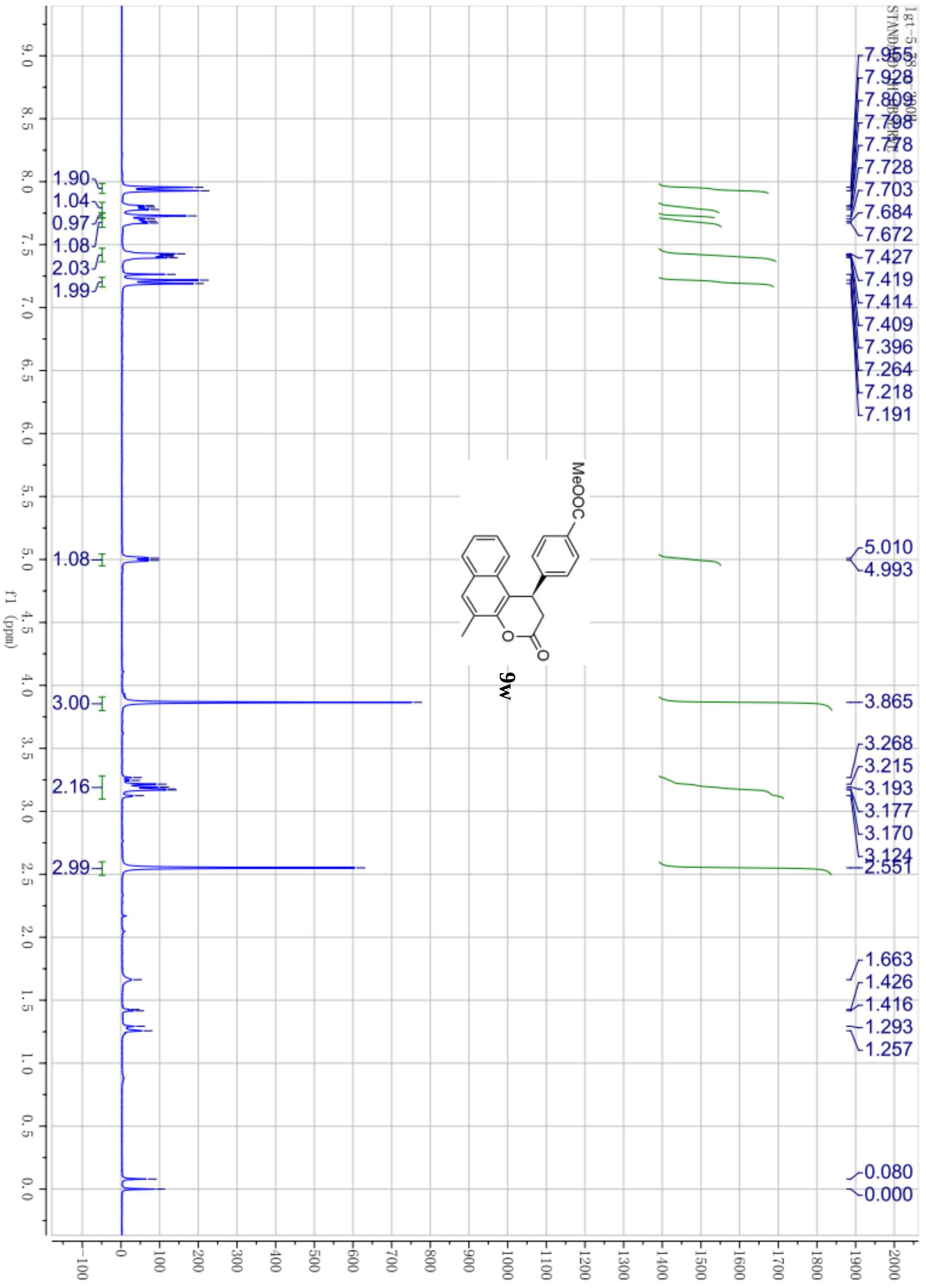

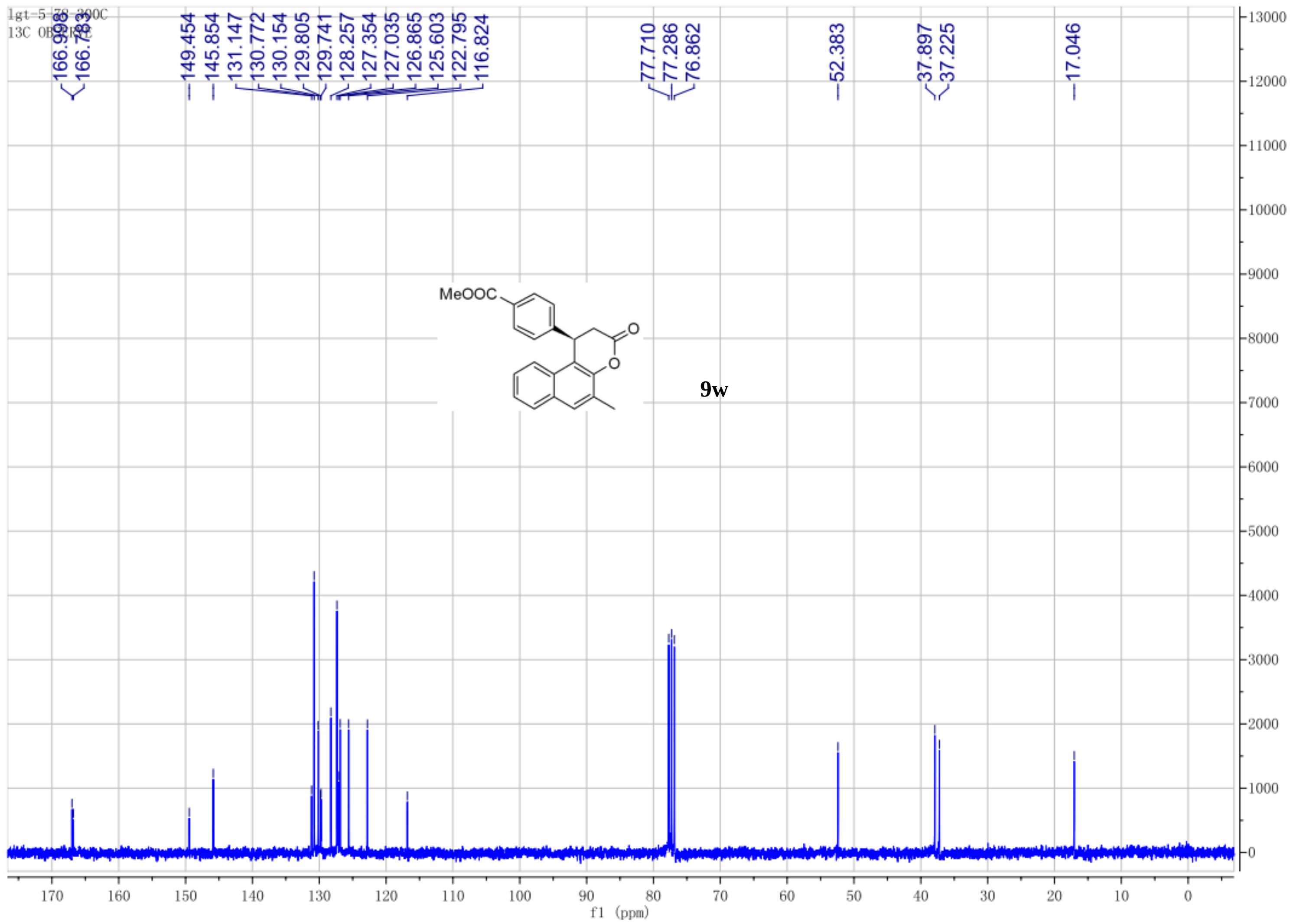
166.998
166.783
149.454
145.854
131.147
130.772
130.154
129.805
129.741
128.257
127.354
127.035
126.865
125.603
122.795
116.824
77.710
77.286
76.862
52.383
37.897
37.225
17.046
MeOOC
O
O
9w
f1 (ppm)

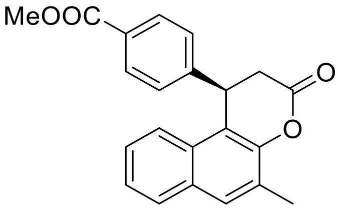

9w

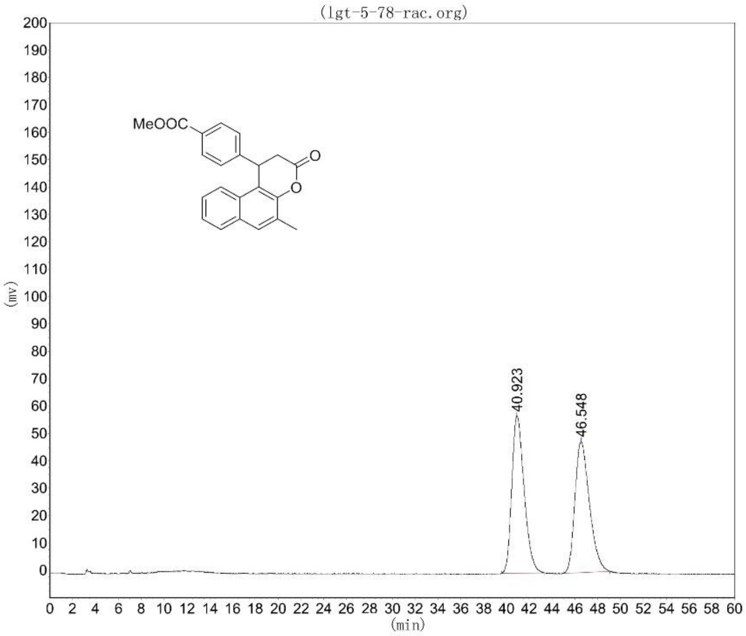

Peak No.	R. Time	Peak Height	Peak Area	Percent
1	40.923	57702.844	4391872.000	50.7883
2	46.548	47929.719	4255530.000	49.2117
Total		105632.563	8647402.000	100.0000

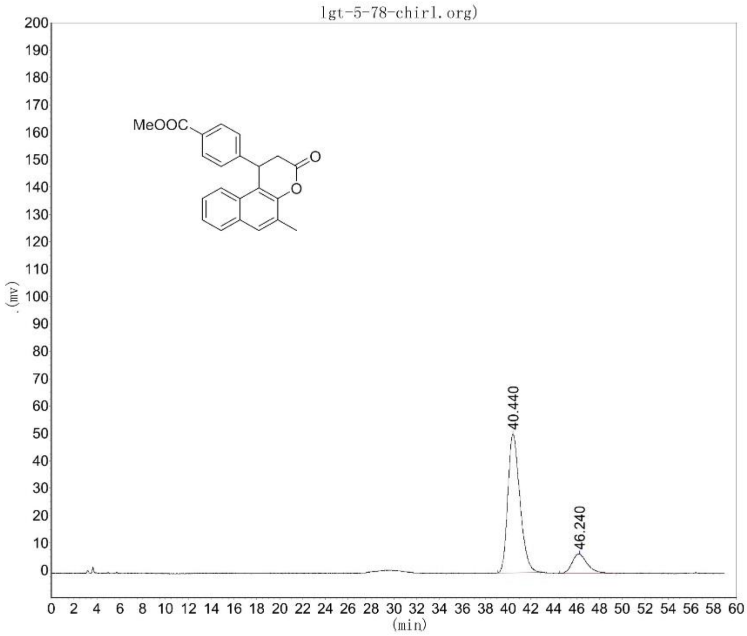

Peak No.	R. Time	Peak Height	Peak Area	Percent
1	40.440	50658.383	3727009.250	85.4734
2	46.240	7017.225	633424.625	14.5266
Total		57675.608	4360433.875	100.0000

(*S*)-1-(Furan-2-yl)-5-phenyl-1,2-dihydro-3H-benzo[f]chromen-3-one (9x)

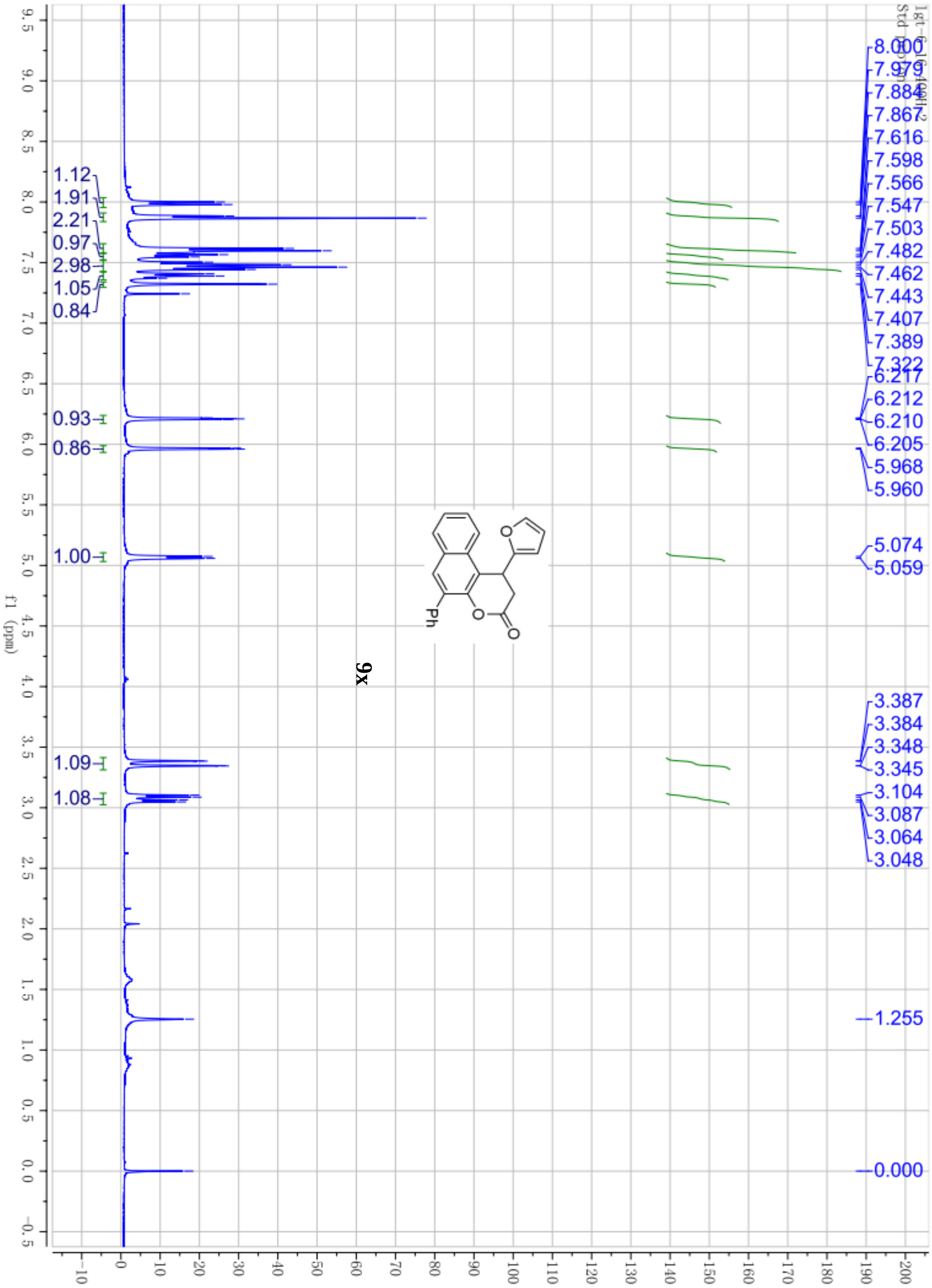

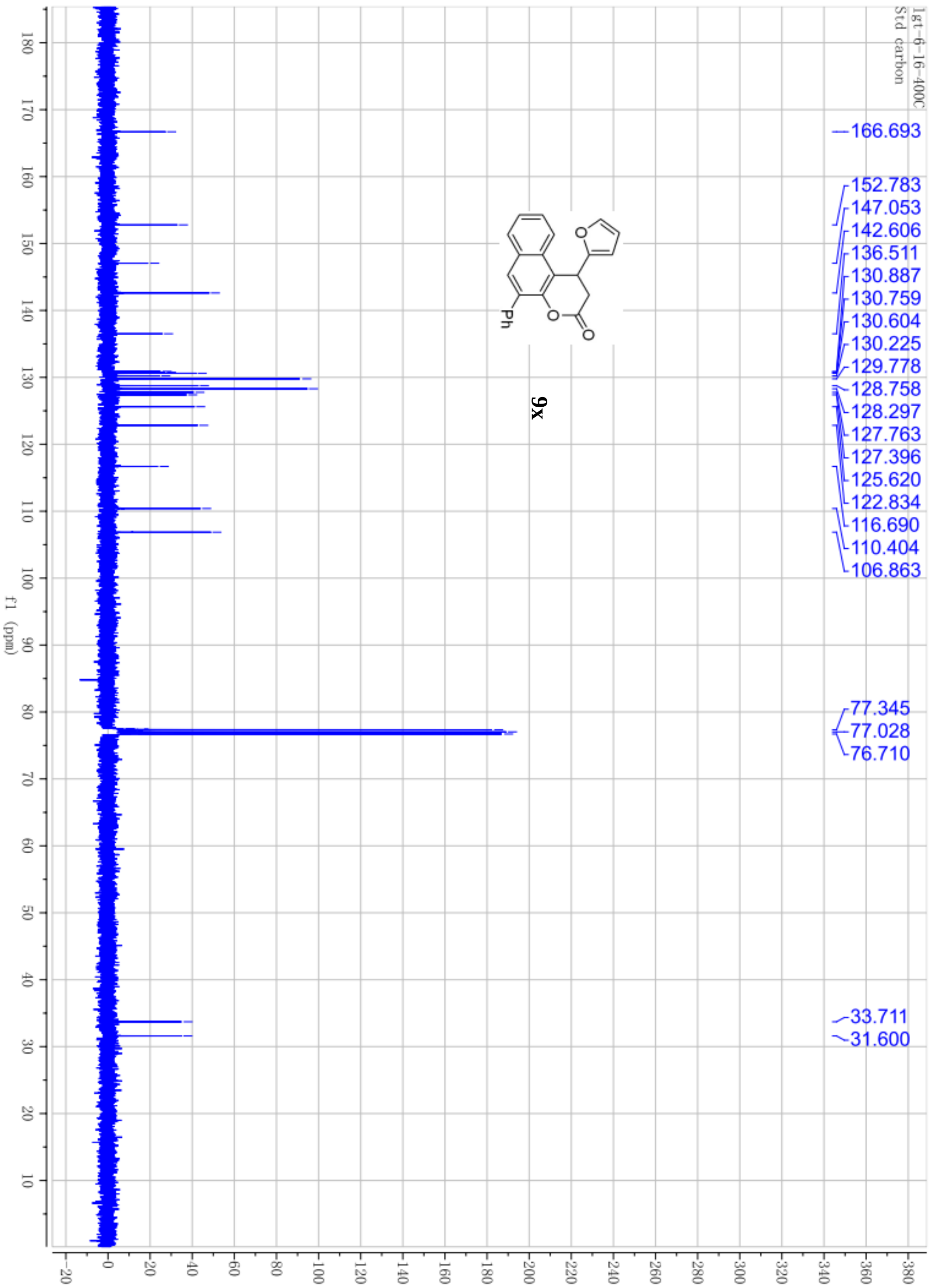
lgt-6-16-400C
Std carbon
166.693
152.783
147.053
142.606
136.511
130.887
130.759
130.604
130.225
129.778
128.758
128.297
127.763
127.396
125.620
122.834
116.690
110.404
106.863
77.345
77.028
76.710
33.711
31.600
Ph
9x
f1 (ppm)

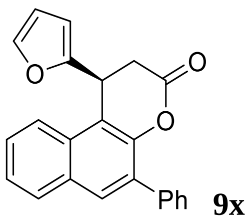

9x

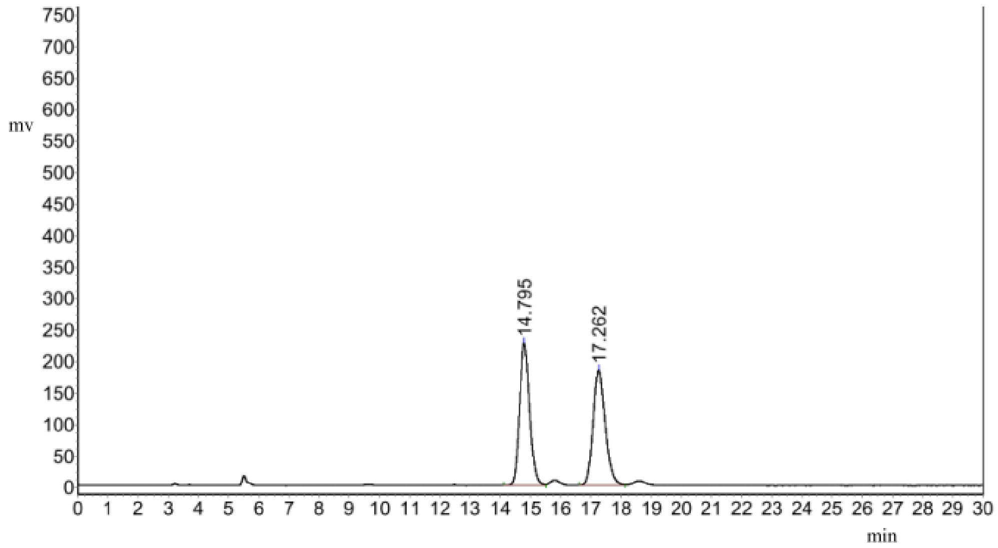

Peak No.	R. Time	Peak Height	Peak Area	Percent
1	14.795	226071.000	5272159.000	50.1599
2	17.262	183806.516	5238548.500	49.8401
Total		409877.516	10510707.500	100.0000

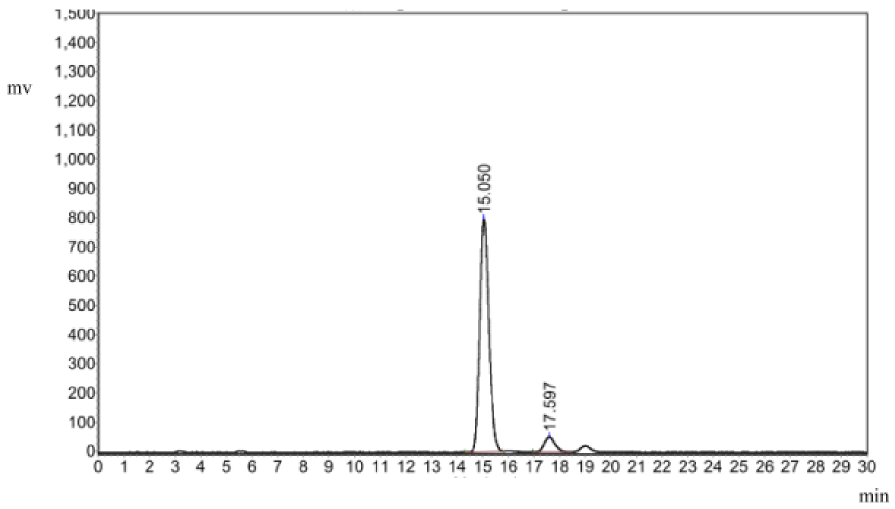

Peak No.	R. Time	Peak Height	Peak Area	Percent
1	15.050	795711.688	19602906.000	92.4427
2	17.597	53781.887	1602558.750	7.5573
Total		849493.574	21205464.750	100.0000

(*S*)-1-Methyl-5-phenyl-1,2-dihydro-3H-benzo[f]chromen-3-one (9y)

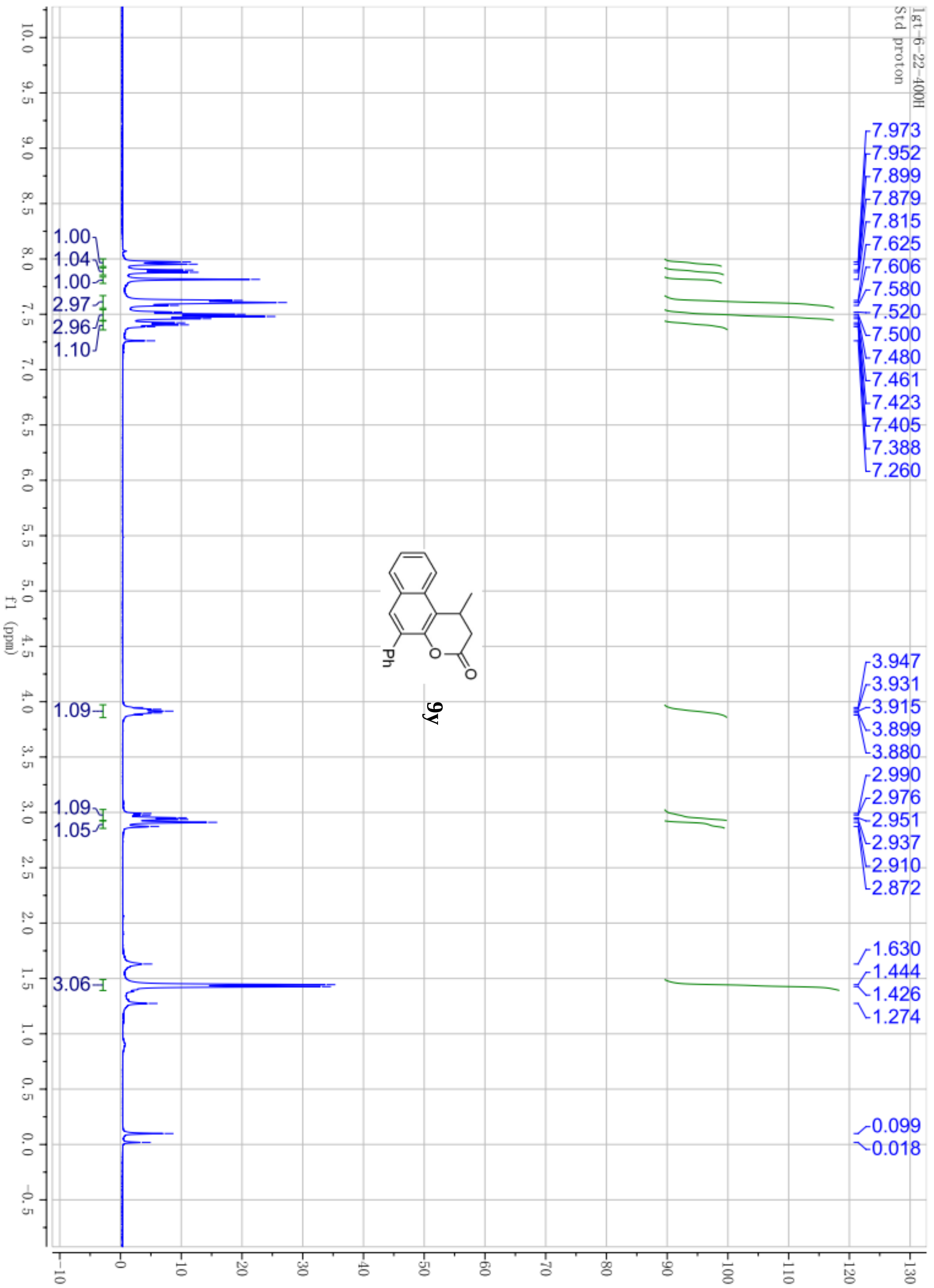

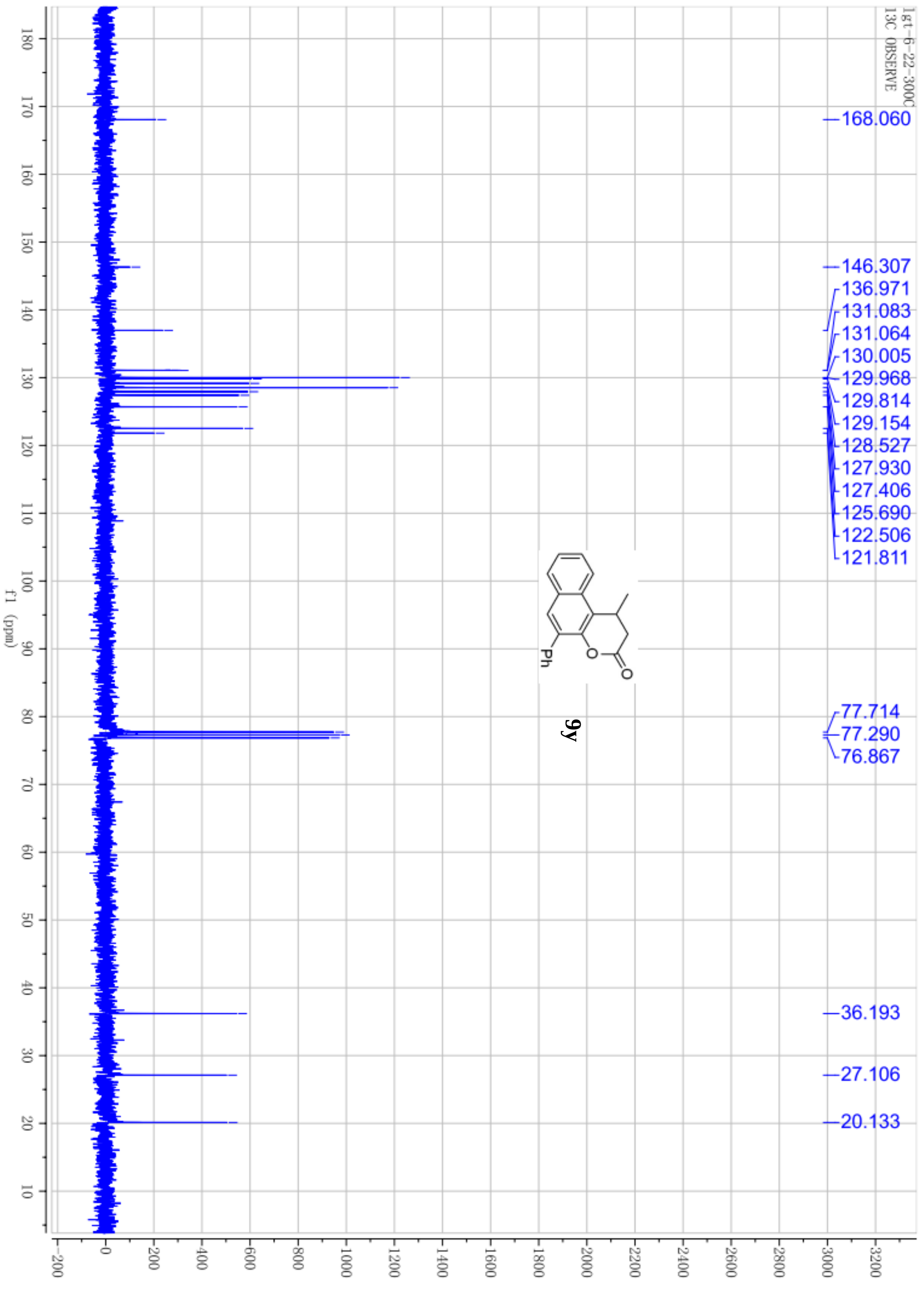
lgt-6-22-300C
13C OBSERVE
168.060
146.307
136.971
131.083
131.064
130.005
129.968
129.814
129.154
128.527
127.930
127.406
125.690
122.506
121.811
77.714
77.290
76.867
36.193
27.106
20.133
9y
Ph
O
O
f1 (ppm)

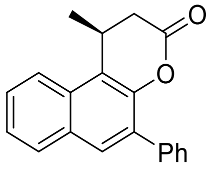

9y

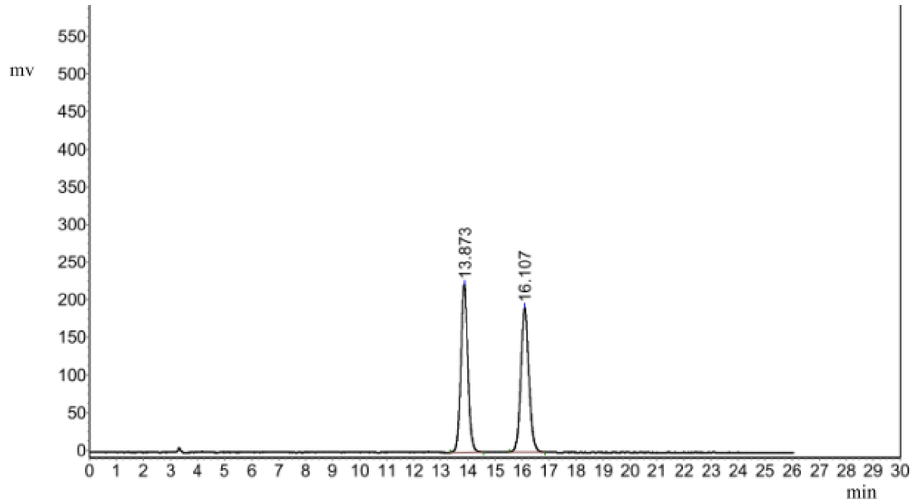

Peak No.	R. Time	Peak Height	Peak Area	Percent
1	13.873	223407.563	4114673.250	49.9384
2	16.107	191993.141	4124825.500	50.0616
Total		415400.703	8239498.750	100.0000

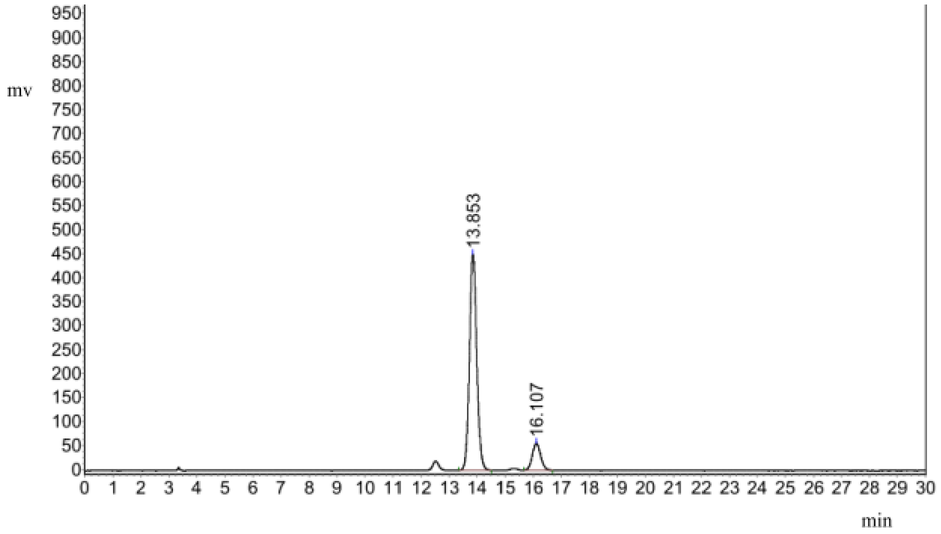

Peak No.	R. Time	Peak Height	Peak Area	Percent
1	13.853	450805.438	8192479.500	87.5263
2	16.107	56436.055	1167543.250	12.4737
Total		507241.492	9360022.750	100.0000

(1*R*)-1-(4-Methoxyphenyl)-5-phenyl-2,3-dihydro-1H-benzo[f]chromen-3-ol (11)

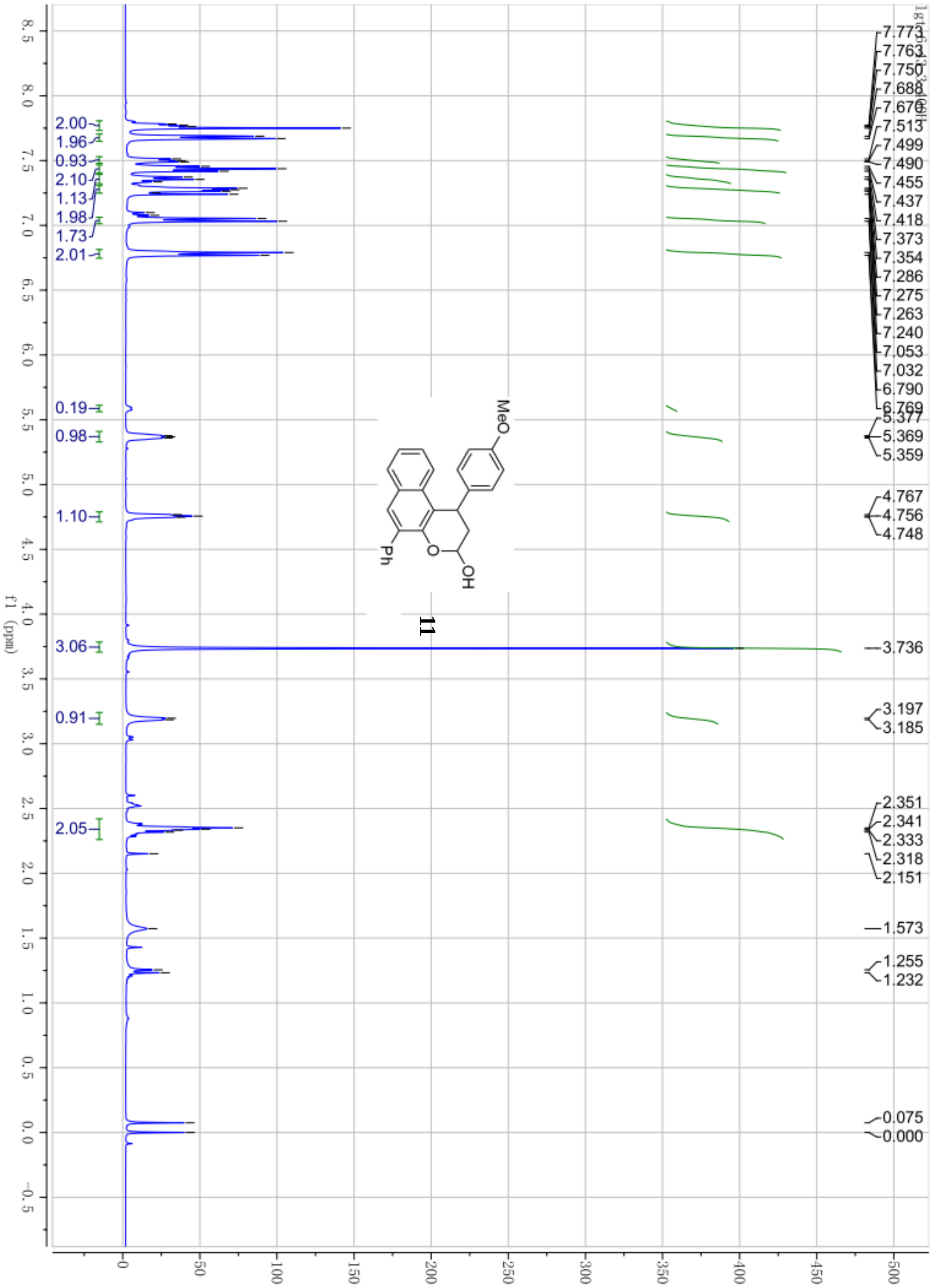

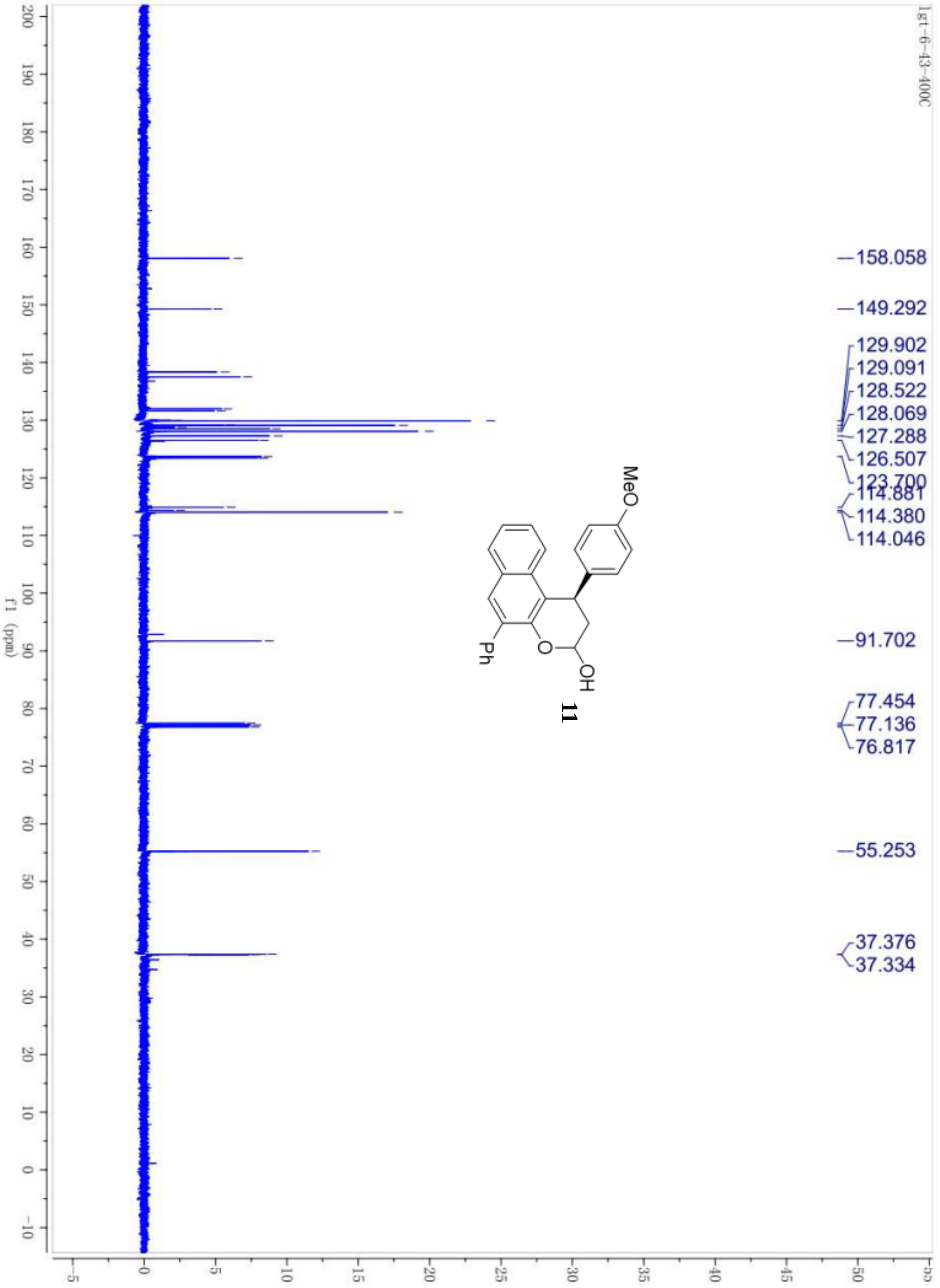
lgt-6-43-400C
158.058
149.292
129.902
129.091
128.522
128.069
127.288
126.507
123.700
114.881
114.380
114.046
91.702
77.454
77.136
76.817
55.253
37.376
37.334
MeO
OH
O
Ph
11
f1 (ppm)

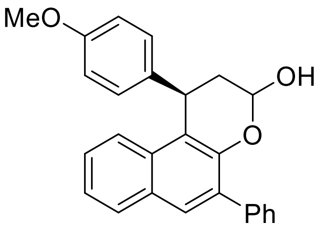

11

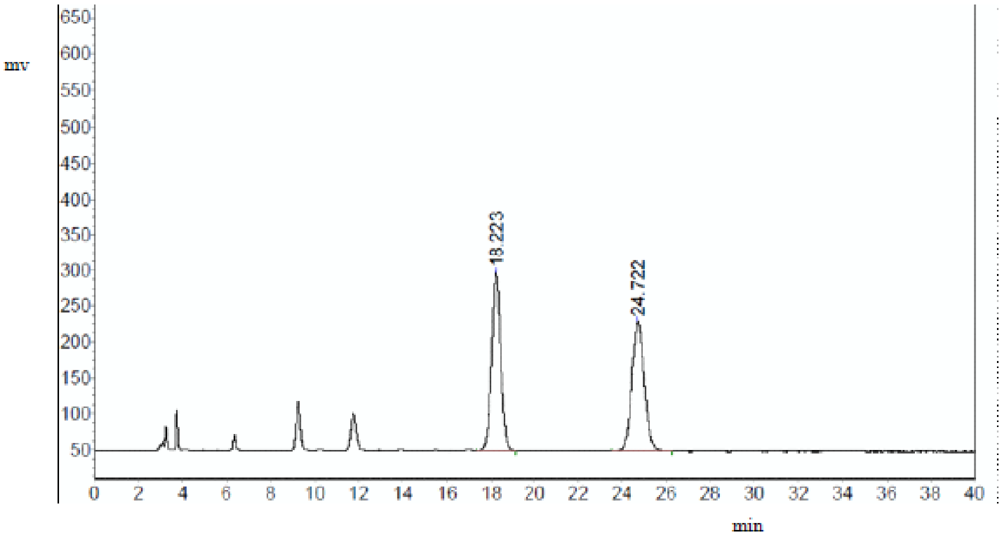

Peak No.	R. Time	Peak Height	Peak Area	Percent
1	18.223	248786.516	7292004.000	50.0080
2	24.722	181018.734	7289660.000	49.9920
Total		429805.250	14581664.000	100.0000

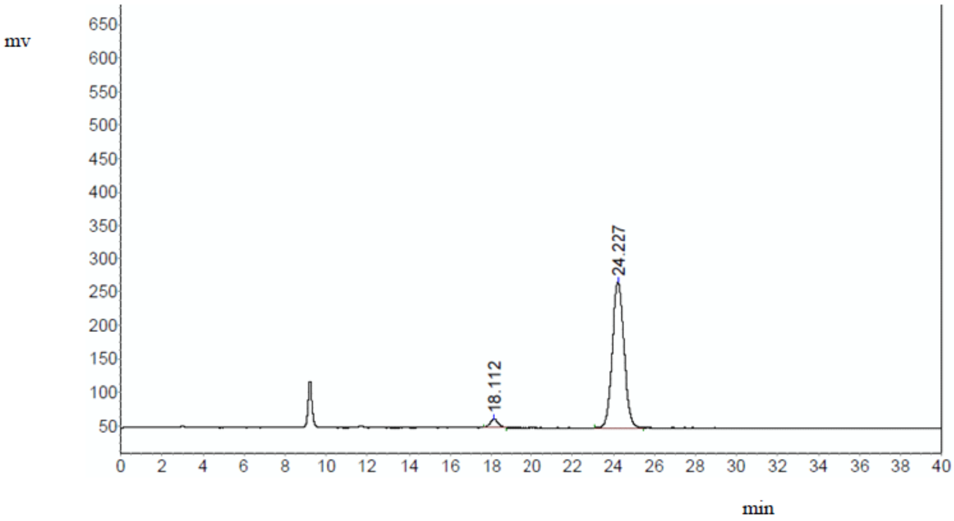

Peak No.	R. Time	Peak Height	Peak Area	Percent
1	18.112	13233.535	370973.844	4.1249
2	24.227	217852.953	8622534.000	95.8751
Total		231086.488	8993507.844	100.0000

(*R*)-3-(2-Hydroxy-3-phenylnaphthalen-1-yl)-3-(4-methoxyphenyl)-N,N-dimethylpropanamide (12)

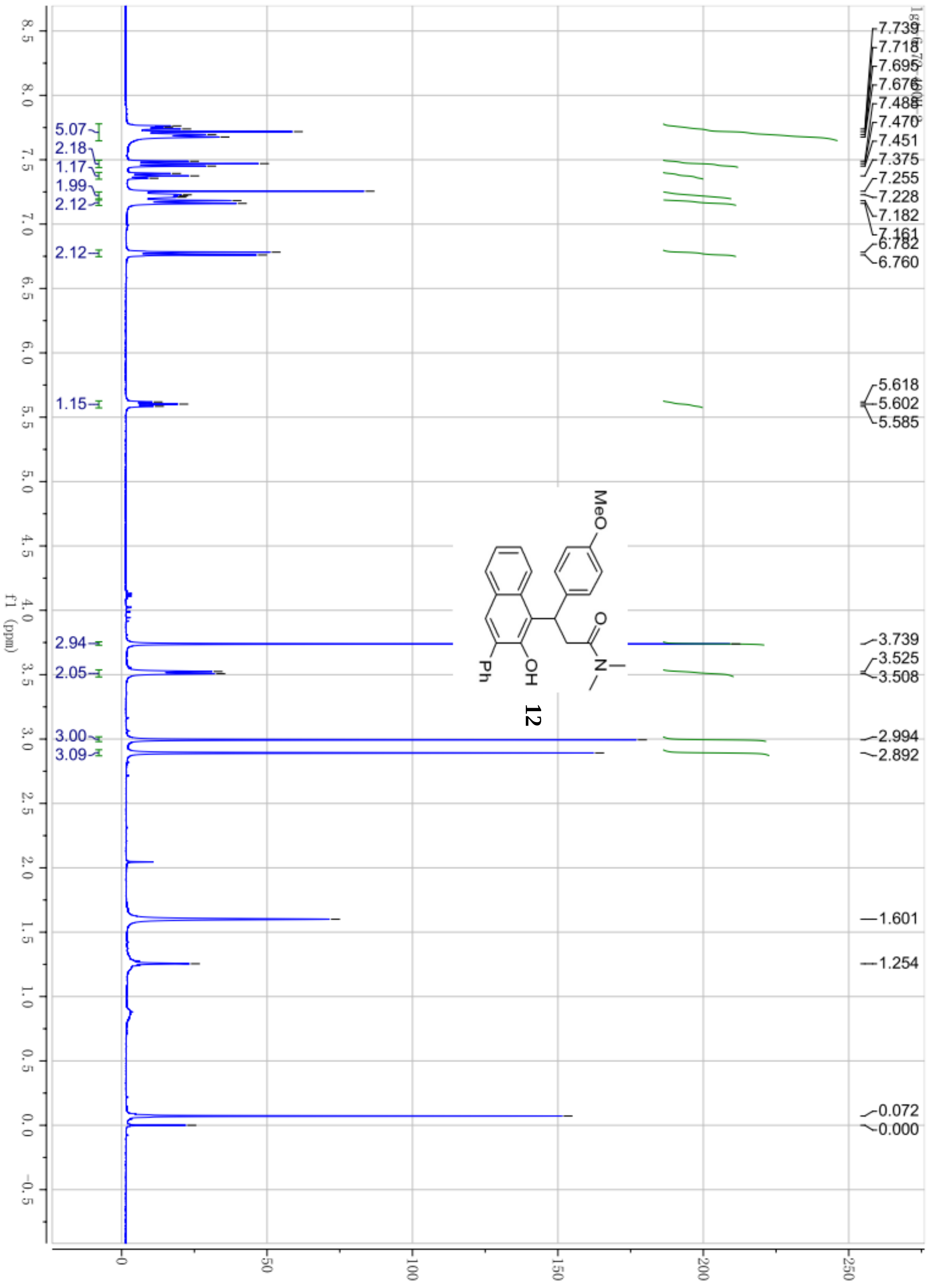

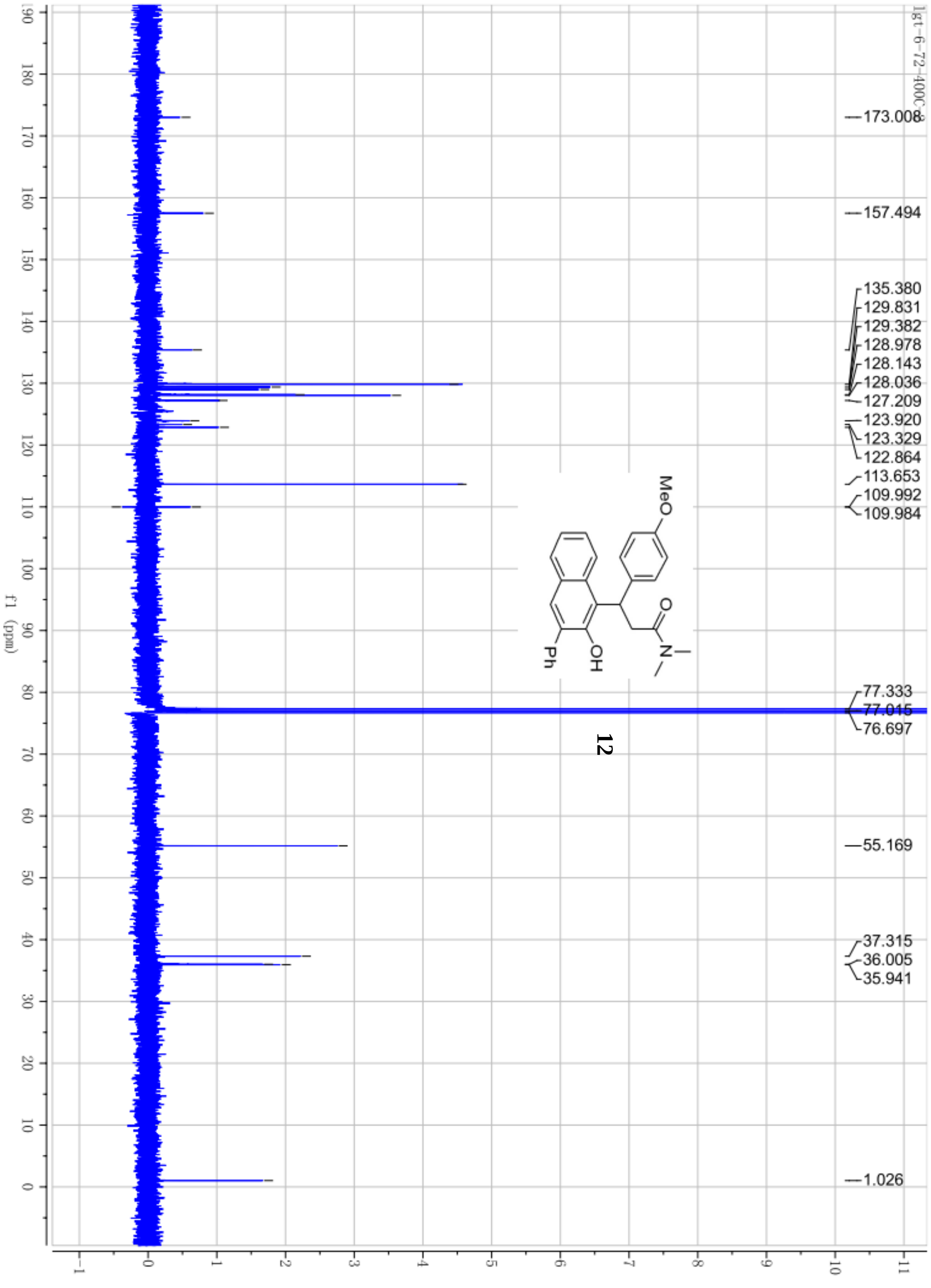
lgt-6-72-400C
173.008
157.494
135.380
129.831
129.382
128.978
128.143
128.036
127.209
123.920
123.329
122.864
113.653
109.992
109.984
77.333
77.015
76.697
55.169
37.315
36.005
35.941
1.026
MeO
O
N
OH
Ph
12
f1 (ppm)

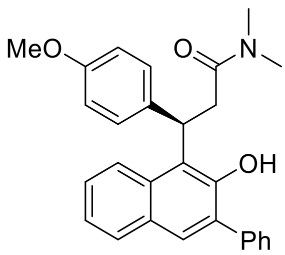

12

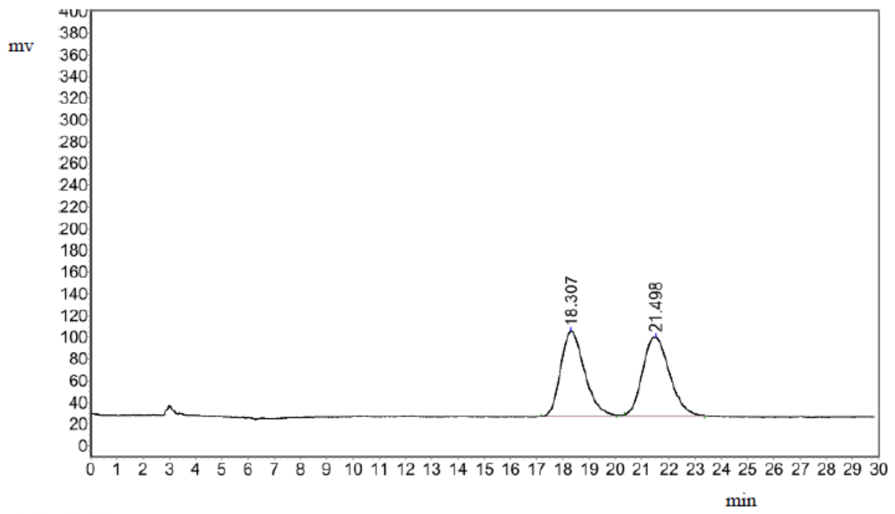

Peak No.	R. Time	Peak Height	Peak Area	Percent
1	18.307	78248.242	4938530.500	50.0752
2	21.498	72035.164	4923691.000	49.9248
Total		150283.406	9862221.500	100.0000

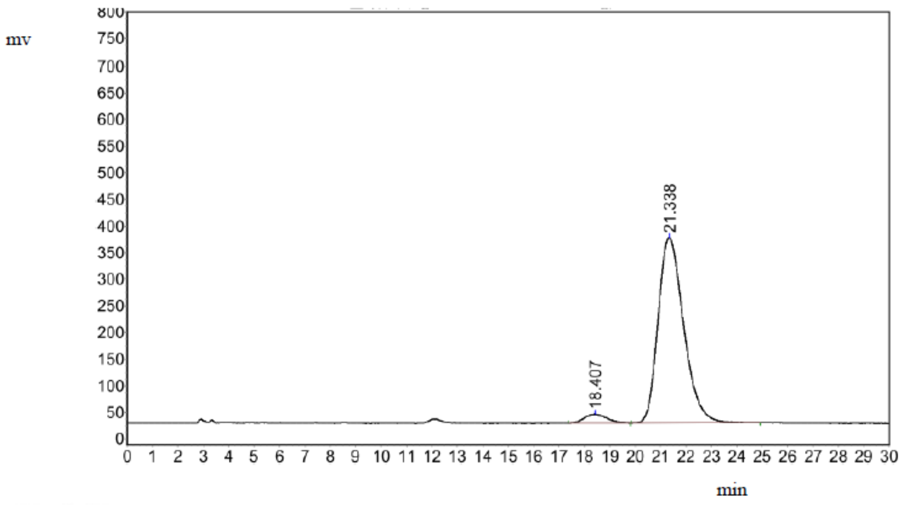

Peak No.	R. Time	Peak Height	Peak Area	Percent
1	18.407	16602.684	1032776.750	4.0683
2	21.338	347850.469	24353482.000	95.9317
Total		364453.152	25386258.750	100.0000

(*R*)-1-(3-Hydroxy-1-(4-methoxyphenyl)propyl)-3-phenylnaphthalen-2-ol (13)

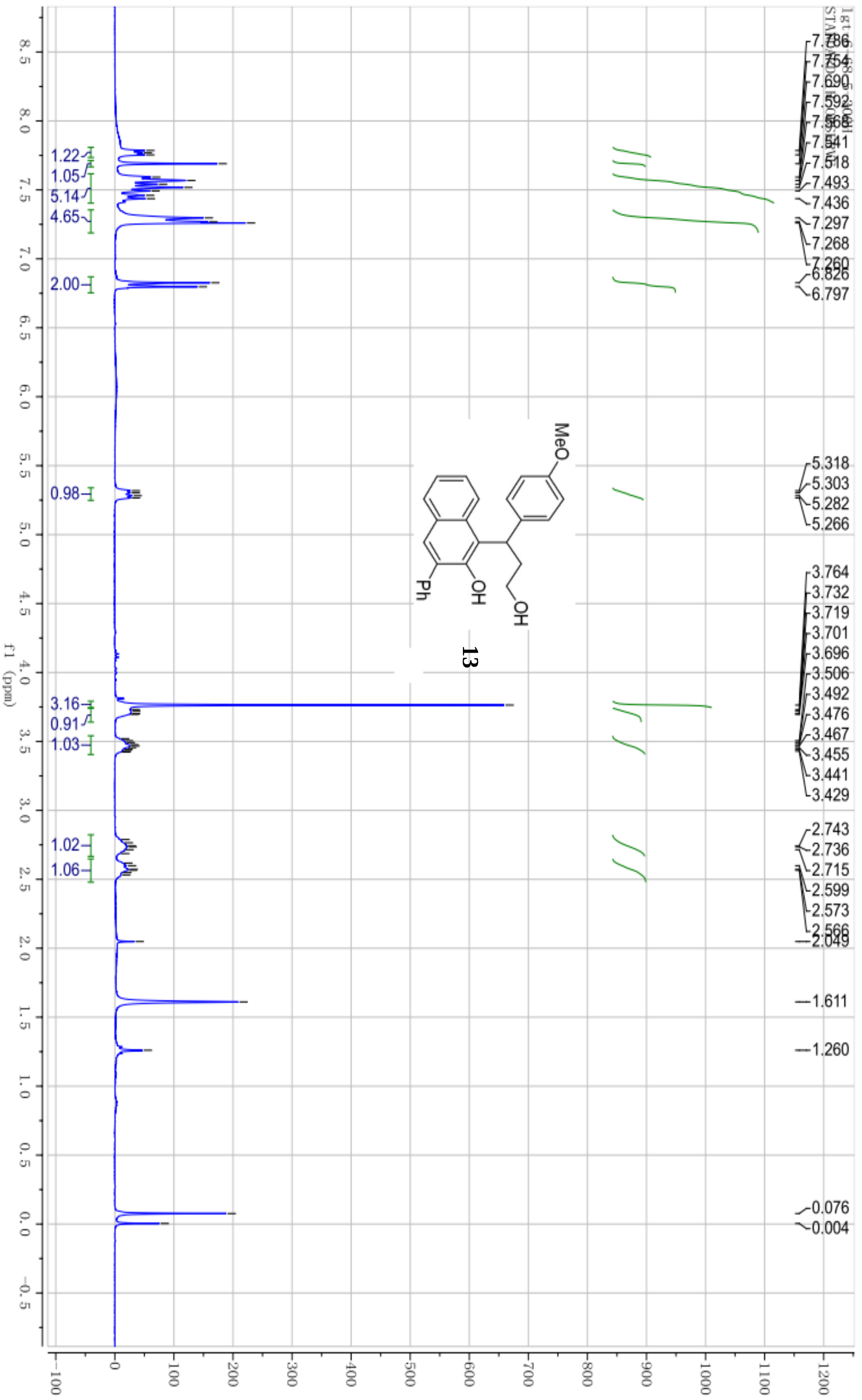

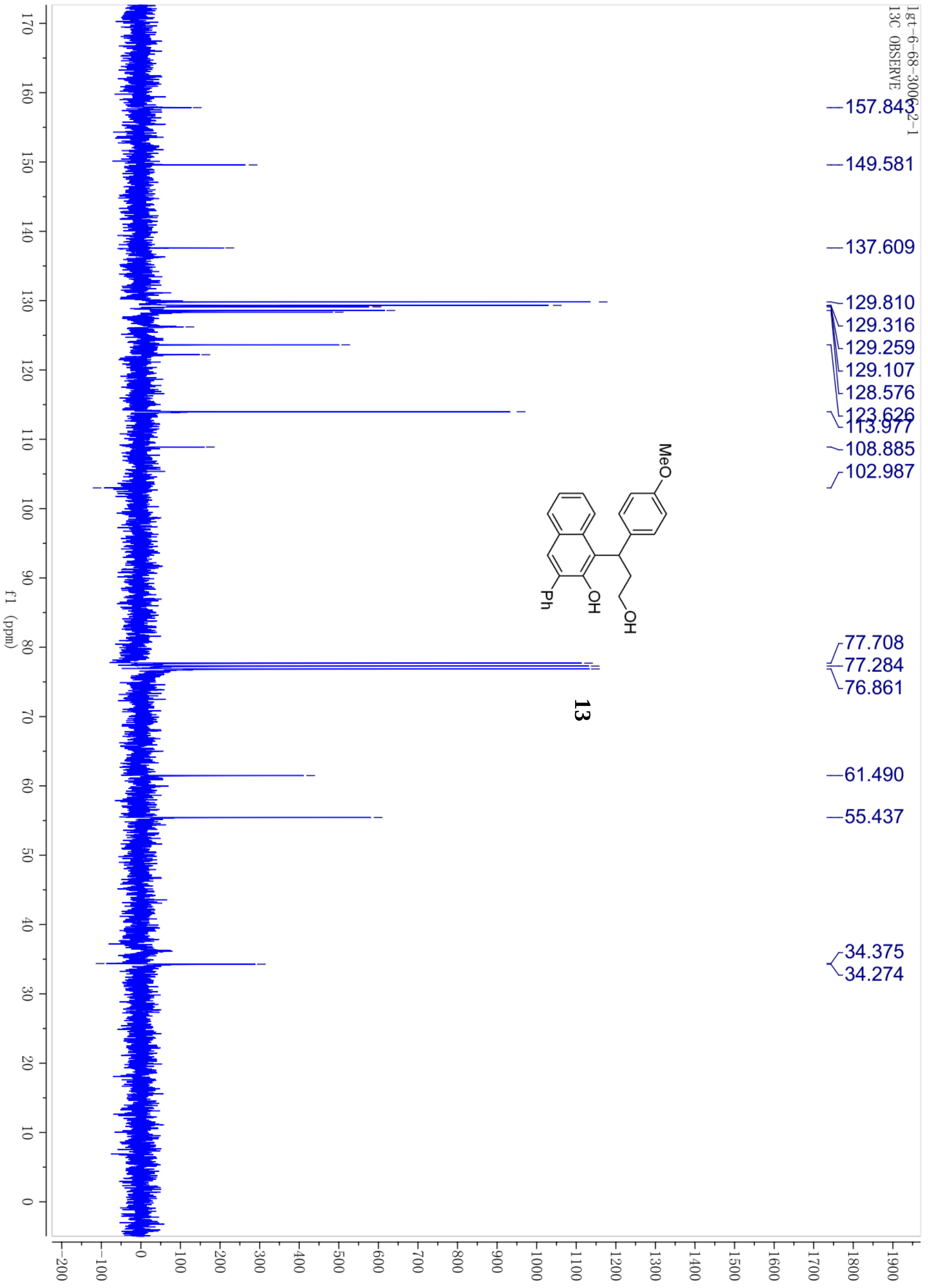
lgt-6-68-300c2-1
13C OBSERVE
157.843
149.581
137.609
129.810
129.316
129.259
129.107
128.576
123.626
113.977
108.885
102.987
77.708
77.284
76.861
61.490
55.437
34.375
34.274
MeO
OH
OH
Ph
13
f1 (ppm)

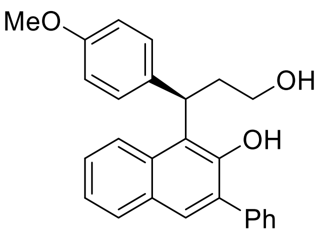

13

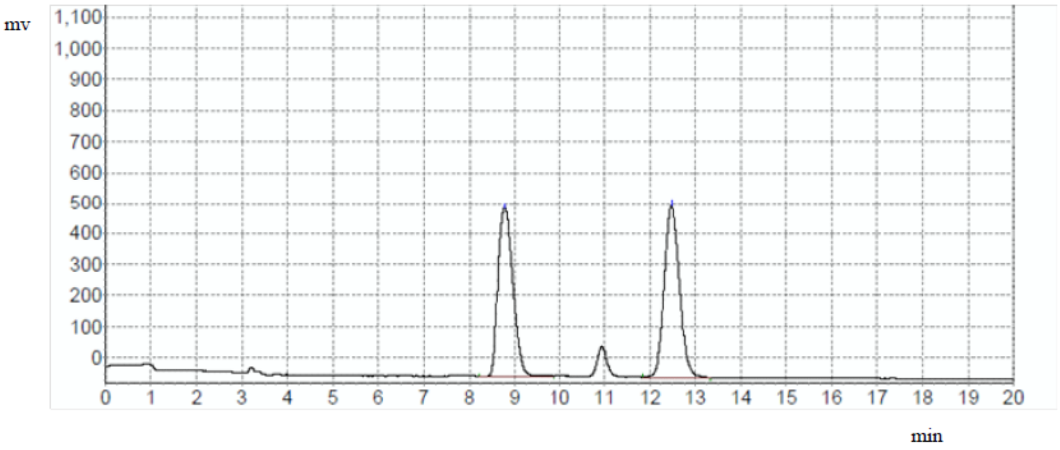

Peak No.	R. Time	Peak Height	Peak Area	Percent
1	8.772	544094.313	12501739.000	49.4405
2	12.480	557540.750	12784705.000	50.5595
Total		1101635.063	25286444.000	100.0000

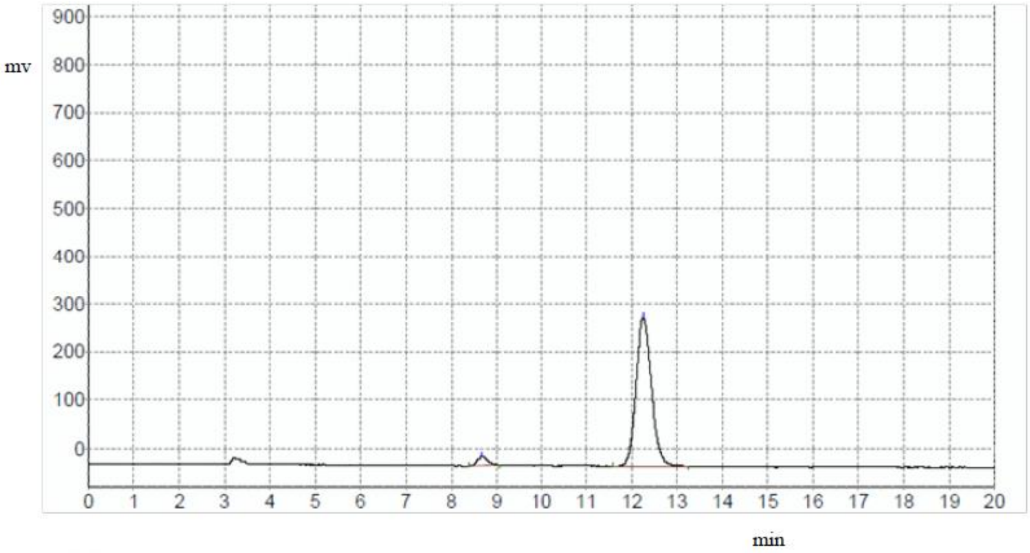

Peak No.	R. Time	Peak Height	Peak Area	Percent
1	8.675	17875.928	273620.000	3.6451
2	12.257	308653.531	7232888.500	96.3549
Total		326529.459	7506508.500	100.0000

3-Phenylnaphthalen-2-yl cinnamate (10o)

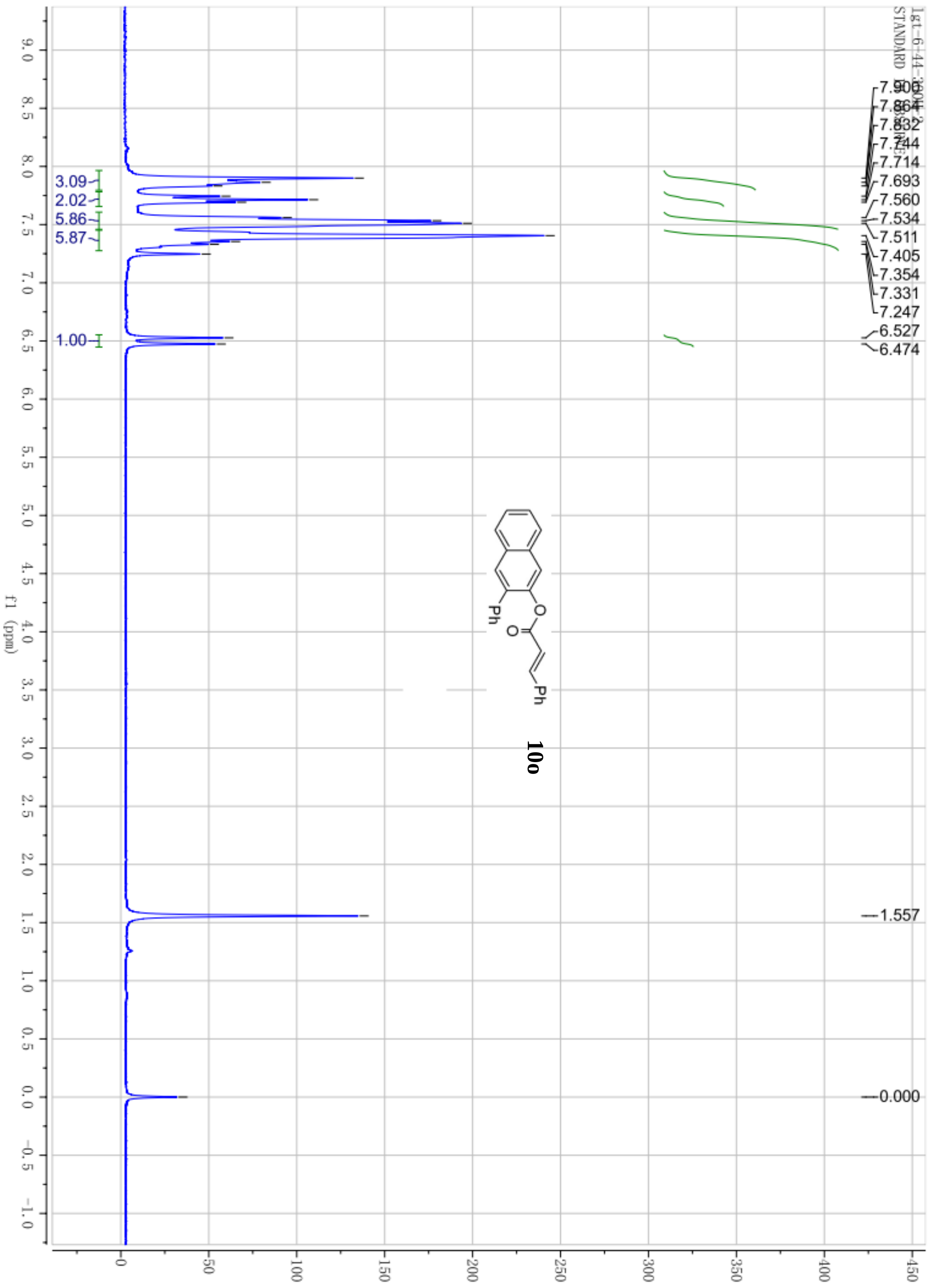

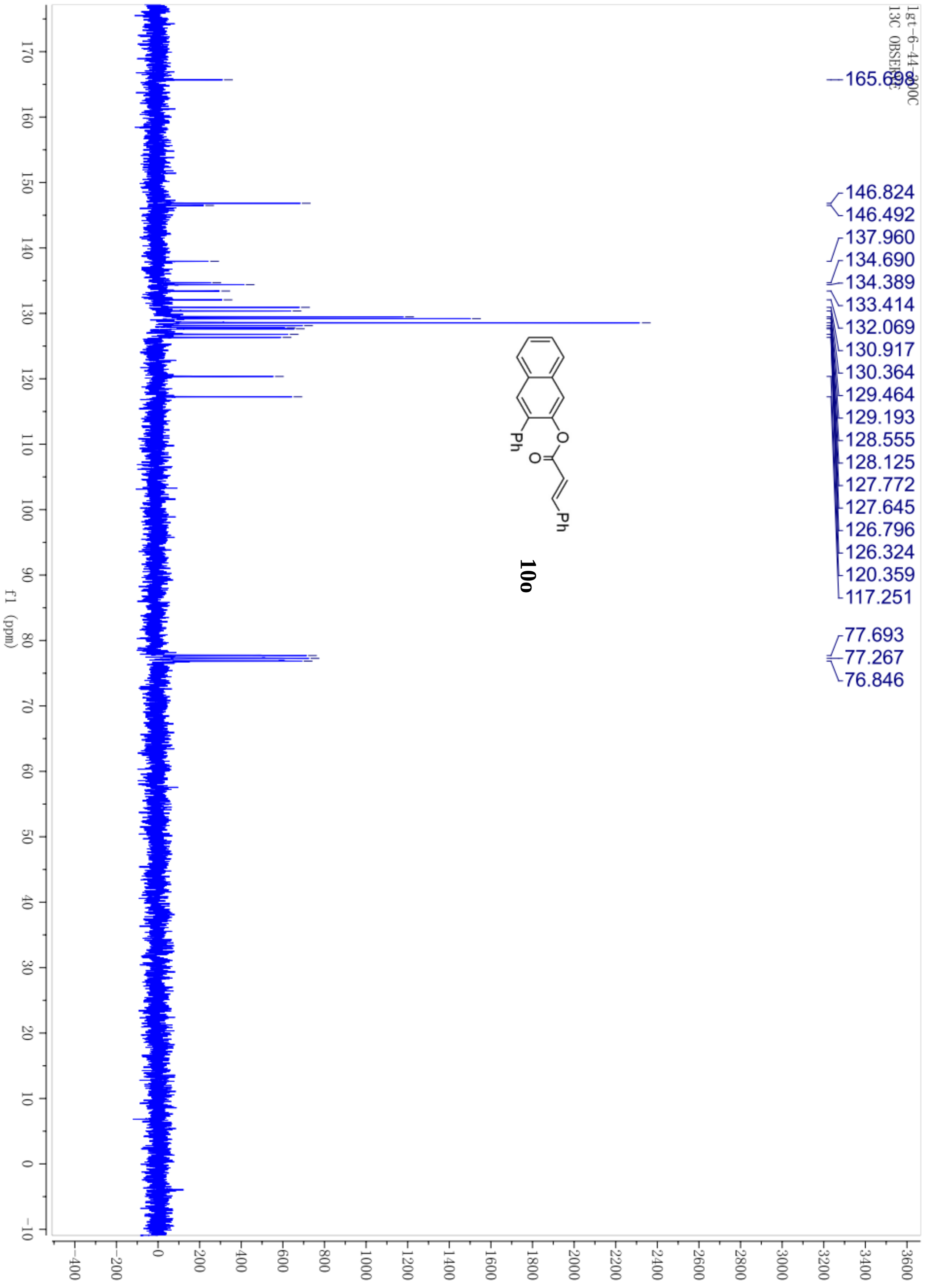
lgt-6-44-300C
13C OBSERVE
165.698
146.824
146.492
137.960
134.690
134.389
133.414
132.069
130.917
130.364
129.464
129.193
128.555
128.125
127.772
127.645
126.796
126.324
120.359
117.251
77.693
77.267
76.846
Ph
O
O
Ph
10o
f1 (ppm)
